# The influence of solid state information and descriptor selection on statistical models of temperature dependent aqueous solubility

**DOI:** 10.1186/s13321-018-0298-3

**Published:** 2018-08-29

**Authors:** Richard L. Marchese Robinson, Kevin J. Roberts, Elaine B. Martin

**Affiliations:** School of Chemical and Process Engineering, University of Leeds, Leeds, LS2 9JT UK

**Keywords:** Quantitative structure–property relationships, Solubility, Temperature dependent solubility data, Enthalpy of solution, Machine learning, Random forest, Multiple linear regression, Feature selection, Crystal structure, Lattice energy, Melting point

## Abstract

Predicting the equilibrium solubility of organic, crystalline materials at all relevant temperatures is crucial to the digital design of manufacturing unit operations in the chemical industries. The work reported in our current publication builds upon the limited number of recently published quantitative structure–property relationship studies which modelled the temperature dependence of aqueous solubility. One set of models was built to directly predict temperature dependent solubility, including for materials with no solubility data at any temperature. We propose that a modified cross-validation protocol is required to evaluate these models. Another set of models was built to predict the related enthalpy of solution term, which can be used to estimate solubility at one temperature based upon solubility data for the same material at another temperature. We investigated whether various kinds of solid state descriptors improved the models obtained with a variety of molecular descriptor combinations: lattice energies or 3D descriptors calculated from crystal structures or melting point data. We found that none of these greatly improved the best direct predictions of temperature dependent solubility or the related enthalpy of solution endpoint. This finding is surprising because the importance of the solid state contribution to both endpoints is clear. We suggest our findings may, in part, reflect limitations in the descriptors calculated from crystal structures and, more generally, the limited availability of polymorph specific data. We present curated temperature dependent solubility and enthalpy of solution datasets, integrated with molecular and crystal structures, for future investigations.

**Electronic supplementary material:**

The online version of this article (10.1186/s13321-018-0298-3) contains supplementary material, which is available to authorized users.

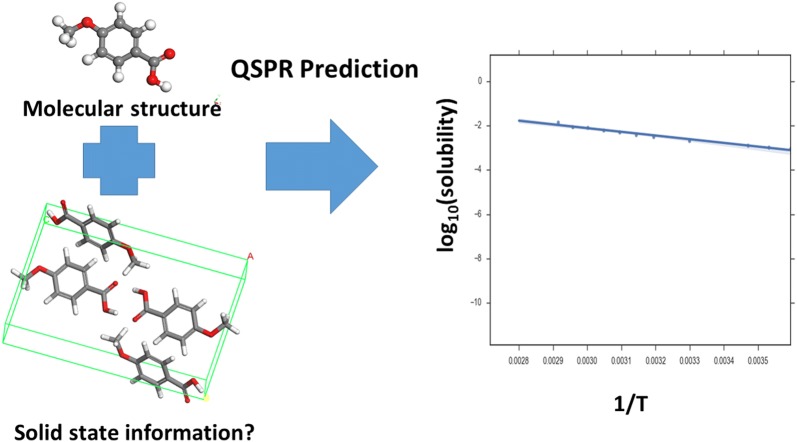

## Introduction

A plethora of computational approaches currently exist to predict the equilibrium solubility of organic chemicals, as well as related thermodynamic terms such as the free energy of solvation [1]. These approaches include data driven statistical modelling approaches, such as quantitative structure–property relationships (QSPRs), as well as various kinds of physics based models. The focus of much of this work is on the prediction of aqueous solubility at a single temperature, or a nominal single value around typical ambient temperatures, to support estimation of product performance, e.g. in terms of the bioavailability of active pharmaceutical ingredients (APIs) or the environmental fate of pollutants [1–3].

In contrast, we are interested in predicting the temperature dependence of equilibrium solubility. Predictions of the solubility of relevant organic crystalline materials, in all relevant solvents, across a range of temperatures are crucial for digital design of unit operations in pharmaceutical manufacturing. For example, they could support the design of cooling crystallization operations [4]. Determination of aqueous solubility at elevated temperatures may also be relevant to the design of wet granulation processes [5, 6].

It is important to note that various kinds of physics based approaches to modelling solution thermodynamics are capable of capturing temperature dependence, including in complex mixtures [1, 7–9]. If combined with estimations of solid state thermodynamic contributions, these might be applied to predict the temperature dependence of solubility [10–13].

However, physics based models are not necessarily more accurate and may be more computationally expensive than QSPR approaches [1, 14]. Interestingly, however, few QSPR models have been developed to capture the temperature dependence of solubility. Some QSPR models were reported to predict the solubilities of organic chemicals, across a range of temperatures, in supercritical carbon dioxide for small (less than 30 chemicals), non-diverse datasets [15, 16]. More recently, two QSPR studies sought to capture the temperature dependence of aqueous solubility for large, chemically diverse datasets [14, 17].

Specifically, Avdeef [17] developed QSPR models for the standard enthalpy of solution, for the unionized solute, in water. Under certain assumptions, the variation in solubility with temperature may be expressed in terms of the van’t Hoff relationship in Eq. (1), where *S* is the solubility (in molar concentration units), *T* is the temperature (in Kelvin), *R* is the molar gas constant and $\Delta H_{sol}^{0}$ is the standard enthalpy of solution [17–20]. If it is assumed that the standard enthalpy of solution is effectively constant over the temperature range of interest, Eq. (1) can be used to interpolate solubility values between temperatures or extrapolate solubility data from one temperature to another [17].

1 $$ \log_{10} S = \frac{{ - \Delta H_{sol}^{0} }}{{\ln \left( {10} \right)RT}} + constant $$

The models for the enthalpy of solution developed by Avdeef [17] were based on different combinations of molecular descriptors and melting point values and built using multiple linear regression (MLR) [21], recursive partition tree [22] and random forest [23, 24]. The melting points were measured or predicted from a molecular descriptors based model [25].

In contrast, Klimenko et al. [14] built a model for directly predicting aqueous solubility at a specified temperature. Their predictions were based on molecular descriptors and a descriptor derived from experimental temperature, with random forest used to train the model.

In the work reported in our current article, we extended the work of Avdeef [17] and Klimenko et al. [14] as follows. Firstly, we investigated the effect of incorporating crystallographic information, in the form of lattice energies or 3D descriptors calculated from an experimental crystal structure, into the models. Secondly, we compared models for the enthalpy of solution based on molecular descriptors with or without melting point values and examined the effect of including melting point values into direct predictions of temperature dependent solubility. In both respects, this means our work is a contribution to the wider debate in the recent literature regarding the importance of explicitly capturing solid state contributions in QSPR models of solubility and whether the availability of crystallographic or melting point information is essential to achieve this [3, 26–30]. Indeed, it has recently been suggested that the major source of error in QSPR prediction of solubility is the failure of molecular descriptors to fully capture solid state contributions [28]. Thirdly, we considered a larger variety of molecular descriptor permutations, with or without the explicit solid state contribution descriptors, including the application of a feature selection algorithm to produce parsimonious models from high dimensional descriptor sets. Finally, we introduced a novel pseudo-cross-validation protocol for evaluating direct models of temperature dependent solubility. This novel validation protocol allowed us to investigate potential optimistic bias when validating those models.

## Methods and data

For brevity, the essential points are provided below and further details are provided, under corresponding sub-headings, in Additional file 1.

### Solubility data curation

Electronic datasets were curated for two endpoints related to temperature dependent solubility: enthalpy of solution values and temperature specific solubility values. Enthalpy of solution data (in kJ/mol) were curated from the publication of Avdeef [17] and temperature dependent solubility data (log_10_[molar concentration]) were curated from the publication of Klimenko et al. [14].

Avdeef [17] reported enthalpy of solution values derived from temperature dependent intrinsic solubility values via van’t Hoff analysis. (Intrinsic solubility refers to the solubility of the unionized solute [1]. Avdeef [17] estimated the intrinsic solubility values from experimental values reported in various literature studies.) Avdeef also presented curated enthalpy of solution values obtained from direct calorimetric measurements, which were considered more reliable [17]. Here, it has been assumed that all curated enthalpy of solution values closely corresponded to the standard enthalpy of solution, such that they could be used via Eq. (1) to interpolate or extrapolate intrinsic solubility data between temperatures.

**Fig. 1:**
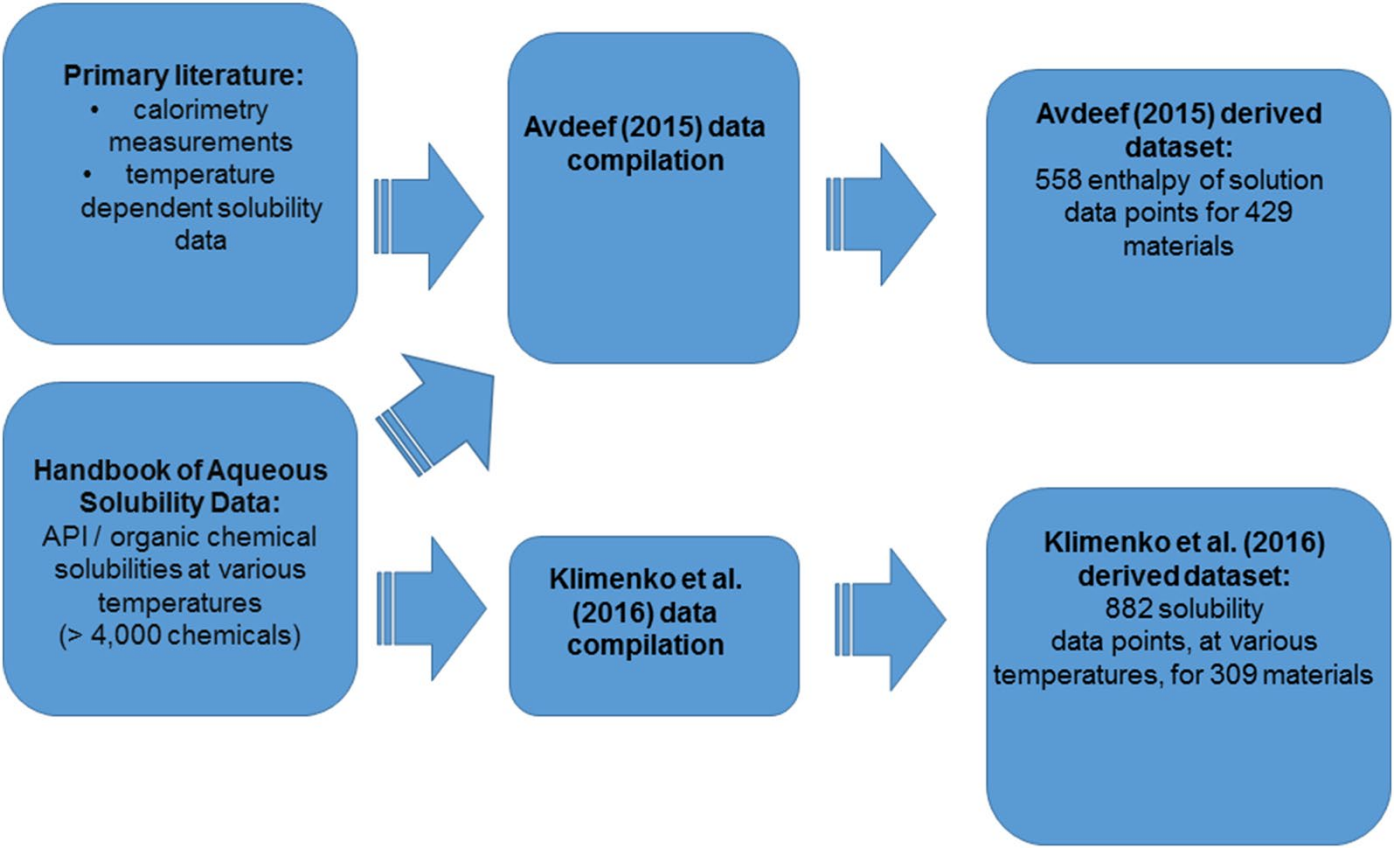
An overview of the curation of endpoint data and associated metadata for endpoints related to temperature dependent aqueous solubility which was carried out for our article. These endpoints were the enthalpy of solution and temperature specific solubility measurements for datasets curated starting from the work of Avdeef [17] and Klimenko et al. [14] respectively. The descriptions on the right hand side of this image refer to the curated datasets we prepared, starting from the information reported in these earlier studies and based on cross-referencing against other references where necessary, from which datasets for QSPR modelling were derived. Full details of the curation process, including explanations of how these curated datasets differed from those reported in the literature, are provided in Additional file 1. See the sections “Solubility data curation” and “Comparison to the literature” therein

As well as curating the endpoint values, we curated the corresponding metadata, including chemical names (or CAS numbers) identifying the molecular species and corresponding polymorph metadata, where this was reported. This included curating the data quality assessments made by Avdeef [17]. An overview of this curation process is provided in Fig. 1.

It should be noted that the “Avdeef (2015) derived dataset” and “Klimenko et al. (2016) derived dataset” labels (Fig. 1) refer to the datasets curated in this work into the electronic template, starting from the work of Avdeef [17] and Klimenko et al. respectively [14], where differences in the datasets arose during the curation process. One key difference between our versions of these datasets and those reported in these earlier studies is that we filtered dataset entries where there was no evidence that the enthalpy of solution or solubility data corresponded to dissolution from the solid state.

### Integration with molecular structures

In the first instance, SMILES representations of molecular structures were retrieved via querying the following online resources: the Chemical Identifier Resolver service [31], ChemSpider [32] and PubChem [33, 34]. For those scenarios where no, or inconsistent, molecular structures were retrieved, other references were consulted to determine the molecular structures.

### Integration with crystal structures

Where possible, Cambridge Structural Database (CSD) refcodes were obtained for each combination of molecular structure identifier and polymorph description (i.e. each material), each refcode denoting a crystal structure [35]. Only a small proportion (< 3%) of solubility or enthalpy of solution data points were associated with a description of the corresponding polymorphic form in the Klimenko et al. [14]. or Avdeef [17] derived datasets respectively, i.e. the polymorph description was typically blank. Hence, in the majority of cases, only a possible match could be determined based upon cross-referencing the molecular identifiers (names and CAS numbers) and molecular structures associated with the data points against the CSD. Nonetheless, where polymorph information was available in the dataset and CSD for provisional matches, conflicting polymorph descriptions were manually identified and the corresponding matches deleted. In keeping with literature precedence, all multiple matches remaining were filtered to only keep the putative lowest energy structure, based upon calculated lattice energy [29, 30].

### Calculation of lattice energies

Lattice energies were calculated from the available crystal structures, using the COMPASS force field [36–38], and used as a descriptor of solid state contribution to the modelled endpoints. This is justified by the fact that solubility can be related to the standard Gibbs free energy change, comprising enthalpic and entropic contributions, upon moving from the solid state to the solution phase [1, 4]. In turn, this may be decomposed into the free energy change of sublimation (breaking of the crystal lattice to form a gaseous phase) and solvation (transfer from the gas phase to the solution phase), i.e. hydration in the case of an aqueous solution [1, 26, 29]. Hence, the enthalpy of solution may be decomposed into the sublimation enthalpy and the solvation enthalpy. The lattice energy is a contribution to the sublimation enthalpy. It is defined as the energy change upon forming the crystal lattice from infinitely separated gas phase molecules [29]. Under certain assumptions, the enthalpy of sublimation may be related to the lattice energy as per Eq. (2) [29]. In Eq. (2), $\Delta H_{sub}$ represents the enthalpy of sublimation, $E_{latt}$ the lattice energy, $R$ the gas constant and $T$ the temperature in Kelvin.

2 $$ \Delta H_{sub} = - E_{latt} - 2RT $$

### Validation of lattice energies

The calculated lattice energies were compared to the experimental estimates of lattice energies, obtained from experimental sublimation enthalpies via Eq. (2) and assuming a constant temperature of 298 Kelvin, for a subset of the SUB-48 dataset from McDonagh et al. [29]. (See “Filtering of SUB-48 Dataset” in Additional file 1.) In keeping with the solubility and enthalpy of solution datasets, this dataset also comprised a set of single component crystals, was a mixture of general organic and pharmaceutical API small molecules and was filtered in keeping with the crystal structure selection criteria applied when integrating the QSPR datasets with crystal structures.

### Preparation of molecular structures for descriptor calculations

Prior to calculating 2D molecular descriptors, all molecular structures were standardized and filtered. Prior to calculating 3D molecular descriptors, from the conformer generator structure but not the crystal structure, similar standardization was applied to the structures retained for the QSPR ready datasets, yet stereochemistry was retained prior to conformer generation.

### Calculation of 2D molecular descriptors

The choice of 2D molecular descriptors was based upon the different permutations considered by Klimenko et al. [14] and Avdeef [17]. Where possible, we sought to calculate the same subsets of descriptors as per these previous studies, and to consider the same combinations of these subsets, as well as the combined pool of all molecular descriptors. Each of the different subsets is denoted by a label explained in Additional file 1: Table S1 and the combinations of 2D molecular descriptors evaluated are enumerated, therein, using these labels. These labels are also used in the file names of the versions of the QSPR ready datasets (see Table 1) provided in Additional File 2, to denote the 2D molecular descriptors incorporated into the applicable combination of the available descriptors.

### Calculation of crystal structure based 3D molecular descriptors

In addition to employing calculated lattice energies as a descriptor, the value of crystallographic information was evaluated via computing 3D molecular descriptors from the molecular structure found in the crystal. Specifically, charged partial surface area (CPSA) descriptors, representing the charge distribution at the molecular surface [39–41], were calculated using Mordred [42, 43]. These may partially capture intermolecular interactions in the solid state. Whereas the calculated lattice energies and experimental melting point data explicitly convey information about the solid state contribution, these descriptors may—in part—implicitly represent this information. However, if the solution state structures are not wholly different, they might partially capture molecular interactions associated with non-solid state contributions. Moreover, these descriptors may also be calculated for 3D molecular structures estimated from the available molecular information. Hence, in order to assess whether these descriptors added value due to their having been computed from the crystal structure, corresponding models were built using CPSA descriptors calculated from the 3D molecular structure derived from the originally curated SMILES using the ETKDG conformer generator algorithm [44, 45] and UFF force-field [46] geometry refinement. These descriptors were only calculated for those dataset entries which could be integrated with crystal structures.

### Temperature descriptor

For a given material, assuming the standard enthalpy of solution may be approximated as a constant over the relevant temperature range, as well as other assumptions, the logarithm of solubility may be linearly related to (1/T), where T is the temperature in Kelvin [c.f. Eq. (1)] [17–20]. Hence, as temperature dependent solubility in log_10_[molar concentration] units was modelled for the Klimenko et al. [14]. derived dataset, the experimental temperature values were transformed to (1/T) to use as a descriptor. N.B. It should be noted that Klimenko et al. [14] proposed a more complicated temperature descriptor. However, for simplicity, and due to the grounding of the (1/T) dependence, under certain assumptions [17–20], in fundamental thermodynamics, we chose to use (1/T) as the descriptor.

### Melting point descriptor

Experimental melting point data were used as a descriptor for all datasets. The data retrieved do not necessarily correspond to the polymorph for which enthalpy of solution or solubility data were modelled.

### QSPR ready datasets

**Fig. 2:**
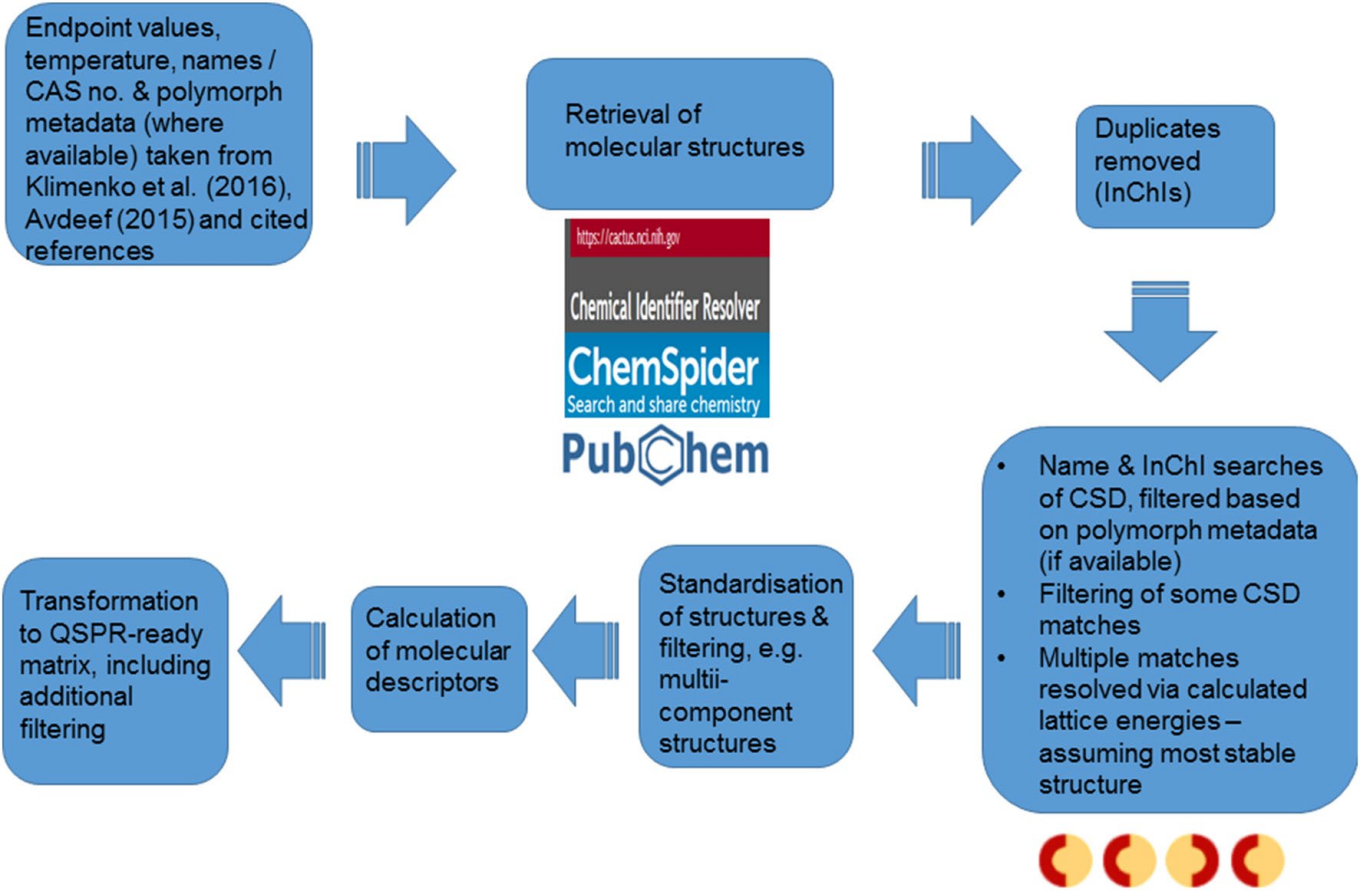
A summary of the steps taken to transform the curated experimental endpoint datasets (see Fig. 1) into the QSPR-ready datasets used for modelling studies (see Table 1). As is explained in the text of Additional file 1, some of these steps were carried out iteratively

Table 1 summarizes the QSPR ready datasets which were used for the evaluation of modelling approaches investigated in our work. A summary of the derivation of these QSPR ready datasets is provided in Fig. 2. These datasets were derived from the curated datasets summarized in Fig. 1, following integration with structural information, standardizing molecular structures, and calculating descriptors. For the enthalpy of solution datasets, data points noted to be low quality by Avdeef [17] were also filtered. The derived dataset matched instance IDs to an endpoint value and a vector of descriptors.

**Table 1:** QSPR ready datasets

Dataset name	Instance identifier^a^	Extra filtering?	No. instances	Total no. descriptors^b^	Endpoint	Source^c^	Integrated with crystal structures?
Avdeef_ExDPs_CS_False	[name]_[CAS no.]_[polymorph description]	Low quality data points removed [17]	364	4776	Enthalpy of solution	Avdeef [17] derived dataset	No
Avdeef_ExDPs_Cal_CS_False	[name]_[CAS no.]_[polymorph description]	Only calorimetry data points retained [17]	50	4776			
Klimenko_CS_False	[name]_[CAS no.]_[polymorph description]_[temperature value]	No	882	3764	Solubility at some defined temperature	Klimenko et al. [14] derived dataset	
Avdeef_ExDPs_CS_True	[name]_[CAS no.]_[refcode]	Low quality data points removed [17]	169	4820	Enthalpy of solution	Avdeef [17] derived dataset	Yes
Avdeef_ExDPs_Cal_CS_True	[name]_[CAS no.]_[refcode]	Only calorimetry data points retained [17]	30	4820			
Klimenko_CS_True	[name]_[CAS no.]_[refcode]_[temperature value]	No	530	3808	Solubility at some defined temperature	Klimenko et al. [14] derived dataset	

^a^The [name] (or [CAS no.]) and/or [polymorph description] could be “none”, denoting the absence of the relevant information

^b^This denotes the complete set of all 2D molecular, temperature (for the solubility datasets), melting point and, for crystal structure integrated datasets, lattice energy and 3D descriptors calculated for instances in this dataset. Different subsets were considered for different models, as described under “Descriptor combinations investigated”

^c^See Fig. 1

The instances in these datasets, i.e. the unique identifiers associated with endpoint values and corresponding descriptor vectors used for modelling, represent different organic crystalline materials, typically corresponding to different molecular chemicals, and—for the Klimenko et al. [14] derived QSPR datasets—different temperatures. Where multiple endpoint data points were associated with a given instance identifier, the arithmetic mean endpoint value was assigned. Hence, each instance identifier only occurred once.

### Descriptor combinations investigated

For the QSPR ready datasets which were not integrated with crystallographic information, all previously described combinations of 2D molecular descriptors were considered, with or without the melting point descriptor and in combination with the temperature descriptor for the Klimenko et al. [14] derived datasets. For the QSPR ready datasets integrated with crystallographic information, the same combinations of descriptors were considered, with or without the calculated lattice energy. In addition, a new set of descriptor combinations were evaluated for these datasets based upon the 3D descriptors calculated from the corresponding crystal structure, or the conformer generator structure. These descriptor combinations were obtained via adding the 3D descriptors, to all descriptor combinations involving the combined set of 2D molecular descriptors, or substituting the combined set of 2D molecular descriptors for the 3D descriptors. Finally, for the high dimensional descriptor combinations containing the complete set of 2D molecular descriptors, but not the 3D descriptors, feature selection was applied to yield another set of descriptor combinations. Feature selection was not applied to the sets containing the 3D descriptors, as initial results obtained with the 2D molecular descriptors were worse when feature selection was applied.

### Feature selection

The feature selection algorithm and rationale is documented in Additional file 1.

### Descriptor scaling

All descriptor values were range scaled to lie between 0 and 1, using the training set ranges, prior to modelling.

### Machine learning

Models were built using Multiple Linear Regression (MLR) [21] and the non-linear random forest regression (RFR) [23, 24] algorithms. For all RFR models, the model was built five times using a different random number generator seed and each tree was grown on a training set sample without replacement, rather than bootstrapping. All cross-validation statistics were averaged (arithmetic mean) across these seeds, as were all descriptor importance values.

### Validation statistics

Model performance was assessed in terms of the coefficient of determination (*R*^2^) and the root mean squared error (RMSE) [47]. (Definitions are provided in Additional file 1.) For the comparison of models on the same test set, these statistics are redundant. However, as *R*^2^ is a composite of the mean squared error and the variance for the test set endpoint values, propagation of errors necessarily makes it less robust. Hence, for comparisons on the same dataset, using the same cross-validation folds, the mean RMSE values were compared. However, RMSE estimates are not comparable for different endpoints or test sets where the range in endpoint values differs [47]. Hence, for comparisons across datasets, or on the same dataset using different cross-validation folds, the mean *R*^2^ values were compared.

### Cross-validation protocols

Initially, a “vanilla” cross-validation protocol was applied: R repetitions of stratified K-fold cross-validation (R = 5, K = 5). In addition, results for a novel “pseudo cross-validation” protocol are reported: the “remove temperature” protocol, labelled the “CV = rt” protocol for brevity.

**Fig. 3:**
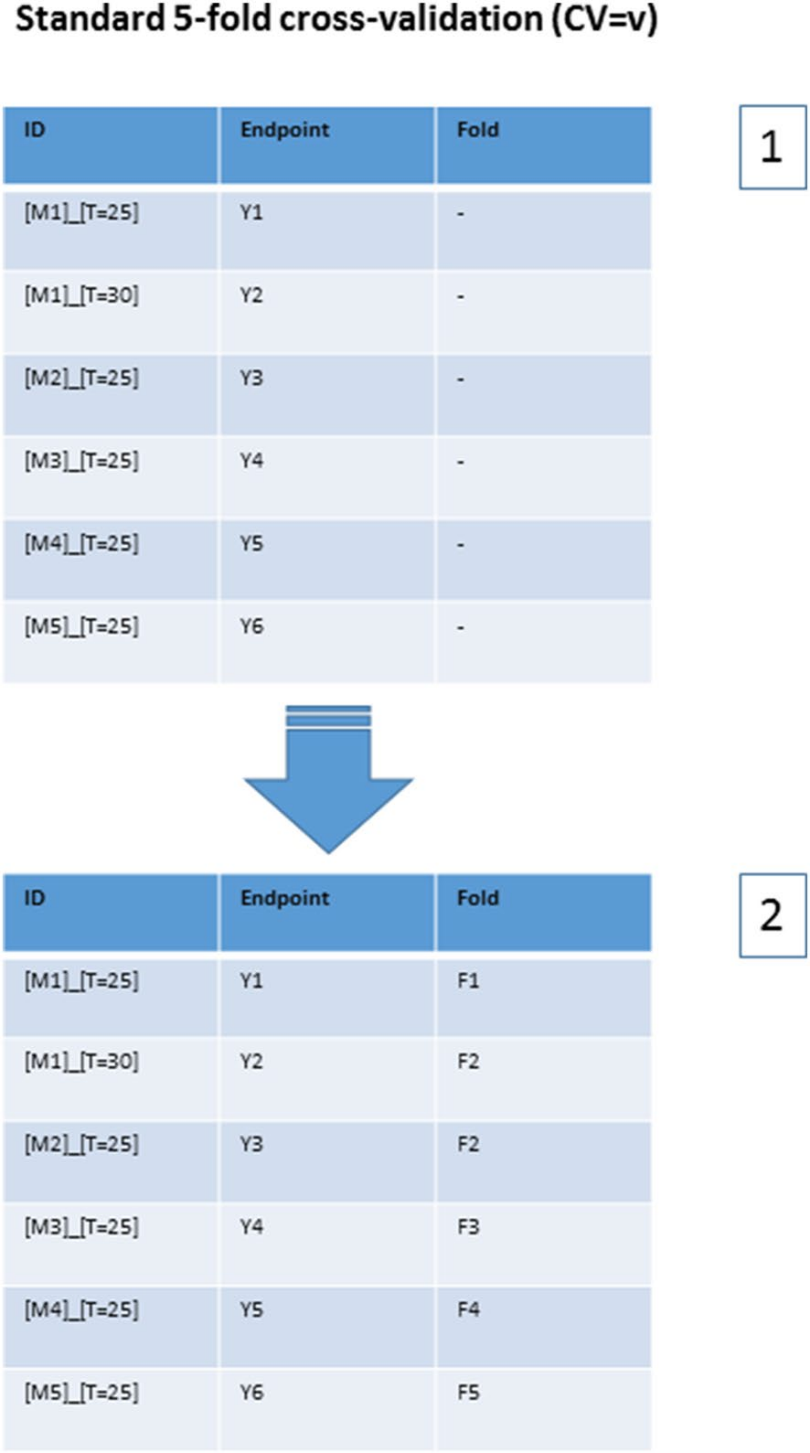
The application of a standard, or “vanilla”, cross-validation protocol (fivefold CV) to temperature dependent endpoint data, where the instance IDs comprise the [MATERIAL IDENTITY]_[TEMPERATURE]. As shown here, instances corresponding to the same material, yet with endpoint values measured at different temperatures, might be assigned to different folds. (For this hypothetical dataset, this means [M1]_[T = 25] and [M1]_[T = 30] were assigned to folds F1 and F2 respectively.) Since each fold is used, in turn, as the test set, with the remaining data being used as the training set, this allows the same material to appear in corresponding training and test sets, when the corresponding endpoint values were measured at different temperatures

**Fig. 4:**
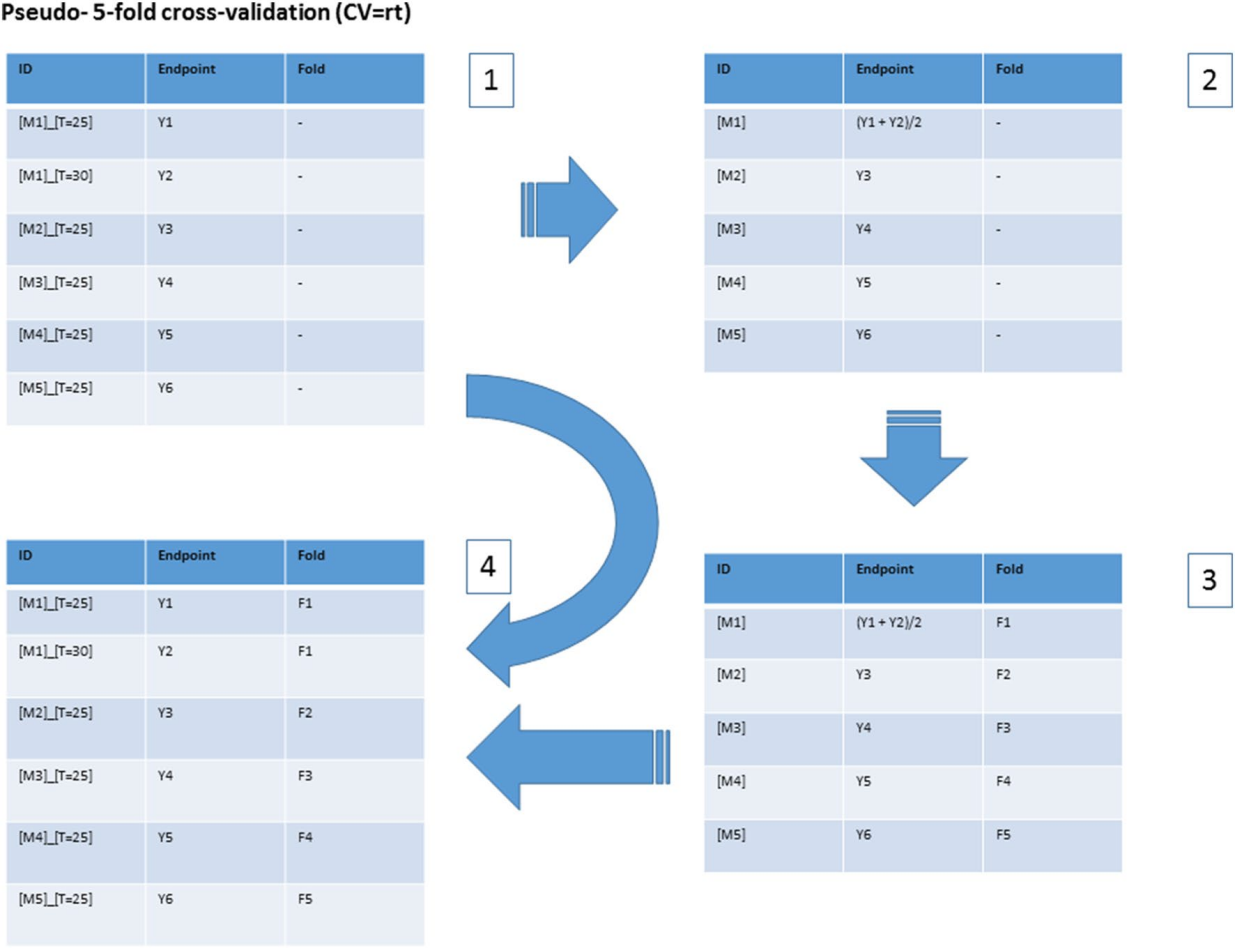
The application of the CV = rt pseudo-cross-validation protocol to the same hypothetical dataset shown in Fig. 3. The first step entails the transformation of 1 into 2, via removing the temperature [T = x] suffix from the ID, deleting all but one occurrence of each truncated ID and assigning this truncated ID the arithmetic mean endpoint value associated with all corresponding original IDs. The transformation of 2 into 3 just entails the application of the standard cross-validation protocol. (In the current case, the nominal endpoint values were required as stratified sampling, based on the distribution of endpoint values, was employed for cross-validation.) Finally, the original dataset IDs are assigned the folds associated with their truncated IDs, in 3, to give the CV = rt folds 4. This ensures that instance IDs corresponding to the same material, yet with endpoint IDs measured at different temperatures, are always assigned to the same fold. (For this hypothetical dataset, this means [M1]_[T = 25] and [M1]_[T = 30] were both assigned to fold F1.) This ensures they can never be placed in corresponding training and test sets

The application of the CV = rt protocol ensured that solubility values for the same organic material, measured at different temperatures, could not be included in the corresponding training and test set. The motivation for introducing this protocol was to assess whether simply applying a “vanilla” cross-validation protocol could give optimistically biased results, when applied to temperature dependent data—where the dataset instances could correspond to the same material, yet with the endpoint value measured at a different temperature. (Hence, this protocol was only applicable to the datasets derived from Klimenko et al. [14], where the endpoint was temperature dependent solubility.) The difference between the “vanilla” cross-validation protocol (CV = v) and the novel pseudo-cross-validation protocol (CV = rt) is illustrated by Figs. 3 and 4 respectively.

### Statistical significance of differences in cross-validated results

Pairwise differences in arithmetic mean validation statistics, from cross-validation, were evaluated for statistical significance for the key scenarios of interest. These key scenarios were pairwise comparisons of all corresponding modelling protocols, or cross-validation protocols, differing only with respect to the following: (1) whether the lattice energy descriptor was included; (2) whether the melting point descriptor was included; (3) whether the crystal structure based 3D descriptors, as opposed to the conformer generator based 3D descriptors, were used; (4) whether feature selection was applied; (5) whether the CV = v or CV = rt cross-validation protocol was applied. For scenarios (1–4), *p* values were computed based on the paired RMSE values. For scenario (5), *p* values were computed based on the *R*^2^ values.

Statistical significance was assessed via calculating approximate *p* values which were then adjusted, separately for each key scenario, to account for the multiple comparisons made. All references to statistically significant results refer to adjusted *p* values < 0.05. However, only approximate assessments of statistical significance could be made and it is possible that the applied analysis somewhat overstated the degree to which statistically significant findings were obtained. Hence, all adjusted *p* values are considered apparent indicators of statistical significance.

### Descriptor importance analysis

A final model, or set of models using five different random seeds for RFR, was built on the entirety of the relevant dataset and the corresponding descriptor importance values, or arithmetic mean values for RFR, were analyzed. For MLR, the magnitudes of the descriptor coefficients were retrieved. For RFR, the descriptor permutation based importance measure was employed [23].

### Lattice energy predictions using molecular descriptors

To get some insight into the extent to which calculating lattice energies from the crystal structures added information to the models of enthalpy of solution and temperature dependent solubility, beyond that inherent in the molecular descriptors, models for the lattice energy descriptor were built using the combined set of 2D molecular descriptors and random forest regression. For the Avdeef_ExDPs_CS_True and Avdeef_ExDPs_Cal_CS_True datasets, the same cross-validation folds were used as per the enthalpy of solution models. For the Klimenko_CS_True dataset, the CV = v cross-validation folds were used and all repeated occurrences of the same combination of lattice energy and descriptor values, due to different solubility values at different temperatures, were removed. Each set of modelling results was generated five times, using different random seeds. Descriptor importance analysis was carried out as per the models of enthalpy of solution and temperature dependent solubility.

### Computational details

Further details related to the software and hardware used to generate our results are documented in Additional file 1.

## Results and discussion

### Summary of cross-validated results

Ultimately, cross-validated modelling results were generated for the Avdeef [17] (enthalpy of solution endpoint) and Klimenko et al. [14] (temperature dependent solubility endpoint) derived datasets, according to a variety of different combinations of molecular (plus temperature, for the temperature dependent solubility endpoint) descriptors, with or without computed lattice energies and with or without melting point values, modelling algorithms (RFR or MLR), feature selection (yes or no) and cross-validation schemes. (N.B. For brevity, we refer to different modelling approaches—meaning a given combination of modelling algorithm, descriptor set and use, or not, of feature selection—as different models.) The predictive performance has been summarized, for each scenario, in terms of the arithmetic mean RMSE and R^2^ values on the validation sets. Detailed results are presented, in Excel workbooks, in Additional file 3. These detailed results include all R^2^ and RMSE values obtained from cross-validation, along with the mean of those values and, for key scenarios described under “Statistical significance of differences in cross-validated results”, pairwise differences in those mean values and the corresponding adjusted *p* values. As explained under “Methods and Data”, models were ranked on the same dataset using the mean RMSE and, with the exception of comparisons between results obtained using different cross-validation folds, *p* values were computed based on the mean RMSE values. All code and dataset files required to generate these cross-validated results are, as documented in Additional file 1, provided in Additional files 4, 5, 6, 7, 8, 9, 10.

### Choosing the most suitable cross-validation protocol

For the Klimenko et al. [14]. derived datasets, pairwise comparison of all corresponding performance estimates obtained with the same model evaluated via the CV = v and CV = rt cross-validation protocols clearly indicated that lower estimates of performance were almost always obtained using the CV = rt protocol. For 107 out of the relevant 116 scenarios, there was an apparent reduction in performance, in terms of the cross-validated mean R^2^, upon moving from the standard cross-validation (CV = v) to the pseudo-cross-validation (CV = rt) protocol. (Of the remaining nine scenarios, all of these corresponded to extreme overfitting of MLR models.) For 90 of these 107 pairwise comparisons, the mean differences appeared to be statistically significant (Additional file 3).

These results are expected: allowing data for the same material to appear in corresponding training and test sets at different temperatures is expected to lead to inflated performance. As we are most interested in predicting temperature dependent solubility profiles for untested materials, we focus on the results obtained with our novel CV = rt protocol, except when comparing our results to those obtained by Klimenko et al. [14].

### Comparison to the literature

For the modelling scenarios which were most directly comparable to the work of Avdeef [17] and Klimenko et al. [14], we typically obtained similar results. Our best results, obtained using different modelling approaches, were better or fairly similar. However, due to refinements we made to their datasets and some differences in descriptor calculations, modelling protocols and validation protocols, we do not report perfectly like-for-like comparisons. Further details are provided in Additional file 1.

### Summary of best cross-validated results

In order to assess the effect of incorporating different sets of descriptors on the predictive performance for both endpoints, we focus on the top performing results. We present the relevant top ranking and second best results in Tables 2 and 3. (Additional file 1: Table S3 includes the top ranking CV = v results for the Klimenko et al. [14] derived datasets.)

**Table 2:** Top ranked results according to various scenarios for the enthalpy of solution datasets

Dataset	Rank	Molecular descriptors	3D descriptors from crystal structure?	Melting point descriptor included	Lattice energy descriptor included	Method	R^2^	RMSE (kJ/mol)
Avdeef_ExDPs_Cal_CS_False	1st	IntegSub, SiRMSSub	False	True	False	RFR	0.63	8.56
Avdeef_ExDPs_Cal_CS_False	2nd	IntegSub, SiRMSSub	False	False	False	RFR	0.63	8.58
Avdeef_ExDPs_Cal_CS_True	1st	IntegSub, SiRMSSub	False	True	False	RFR	0.26	11.45
Avdeef_ExDPs_Cal_CS_True	2nd	IntegSub, SiRMSSub	False	True	True	RFR	0.26	11.47
Avdeef_ExDPs_CS_False	1st	Rdk	False	False	False	RFR	0.34	13.82
Avdeef_ExDPs_CS_False	2nd	Rdk	False	True	False	RFR	0.34	13.84
Avdeef_ExDPs_CS_True	1st	3D	True	False	True	RFR	0.18	14.93
Avdeef_ExDPs_CS_True	2nd	3D	True	True	True	RFR	0.18	14.95

All results were obtained without feature selection. All results are rounded to 2dp. The definitions of the 2D molecular descriptors subsets are provided in Additional file 1: Table S1. All references to R^2^ and RMSE denote arithmetic mean values obtained from cross-validation and all model rankings were generated based on the mean RMSE values

**Table 3:** Top ranked results according to various scenarios for the temperature dependent solubility datasets

Dataset	CV protocol	Rank	Molecular descriptors	3D descriptors from crystal Structure?	Melting point descriptor included	Lattice energy descriptor included	Method	R^2^	RMSE (log units)
Klimenko_CS_False	rt	1st	IntegSub, SiRMSSub, Absolv, Ind, Rdk	False	True	False	RFR	0.92	0.70
Klimenko_CS_False	rt	2nd	Rdk, Absolv	False	True	False	RFR	0.92	0.70
Klimenko_CS_True	rt	1st	3D, IntegSub, SiRMSSub, Absolv, Ind, Rdk	False	True	False	RFR	0.85	0.83
Klimenko_CS_True	rt	2nd	Rdk, Absolv	False	True	False	RFR	0.85	0.83

All results were obtained without feature selection. All results are rounded to 2dp. The definitions of 2D molecular descriptors subsets are provided in Additional file 1: Table S1. All references to R^2^ and RMSE denote arithmetic mean values obtained from cross-validation (CV = rt) and all model rankings were generated based on the mean RMSE values

### Effect of incorporating crystallographic information: lattice energy descriptor

The top performing model for enthalpy of solution, evaluated on the Avdeef_ExDPs_CS_True dataset, included calculated lattice energy as a descriptor (Table 2). However, none of the other top models for enthalpy of solution (Table 2) or direct prediction of temperature dependent solubility (Table 3) incorporated the lattice energy descriptor.

This may be partially attributed to the presence of the melting point or crystal structure based 3D descriptors acting as a partial proxy for the solid state contribution which the lattice energy descriptor is designed to capture. When only those models including neither the melting point nor crystal structure based 3D descriptors are considered (see Additional file 3), it remains the case that the top model for the Avdeef_ExDPs_CS_True dataset does and the top model for the Avdeef_ExDPs_Cal_CS_True dataset does not incorporate the lattice energy descriptor. (However, the results on the smaller Avdeef_ExDPs_Cal_CS_True dataset may be less robust.) However, for the Klimenko_CS_True dataset, the new top ranking model does incorporate the lattice energy descriptor.

**Fig. 5:**
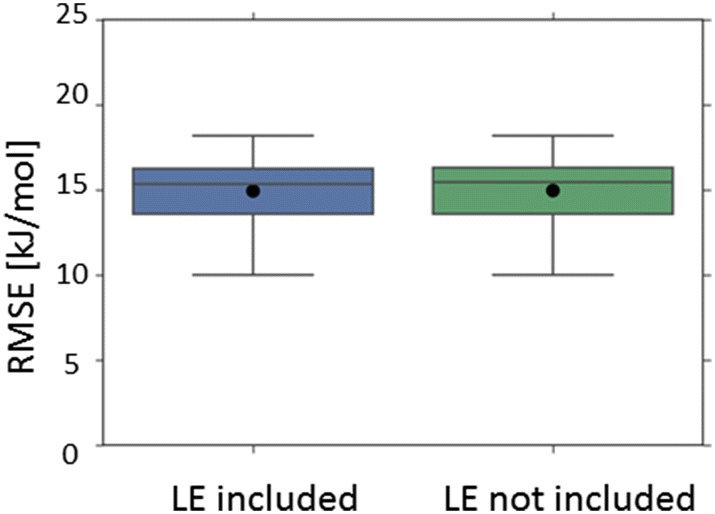
Cross-validated performance (RMSE) of the top performing model where the lattice energy (LE) descriptor was incorporated (LHS), compared to the corresponding model which didn’t include the lattice energy descriptor (RHS): dataset = Avdeef_ExDPs_CS_True. The distributions of cross-validated results are presented as a boxplot, with whiskers extending 1.5 times the interquartile range beyond the upper and lower quartiles, with the arithmetic mean superimposed as a black circle. The presence of a star denotes an apparently statistically significant difference in cross-validated mean RMSE

**Fig. 6:**
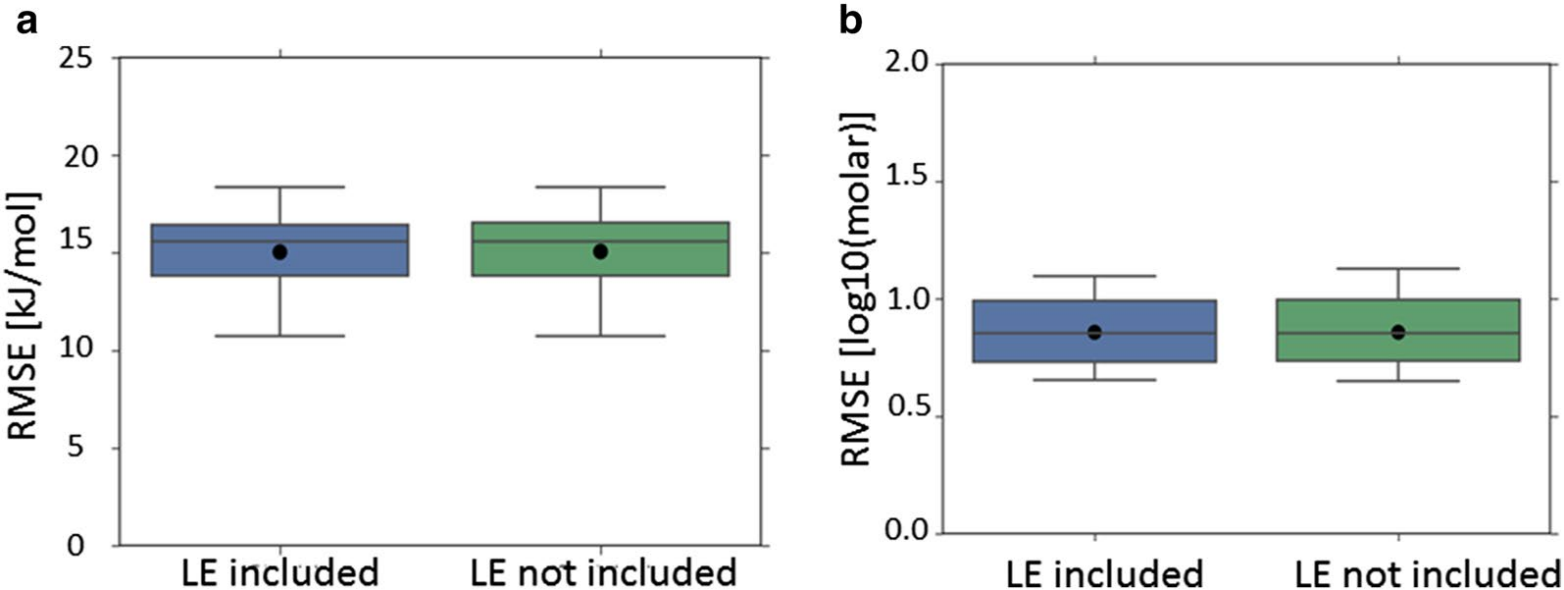
Cross-validated performance (RMSE) of the top performing models (excluding models incorporating the melting point or crystal structure based 3D descriptors) where the lattice energy (LE) descriptor was incorporated (LHS), compared to the corresponding model which didn’t include the lattice energy descriptor (RHS): **a** dataset = Avdeef_ExDPs_CS_True; **b** dataset = Klimenko_CS_True, CV = rt. All results are presented as per Fig. 5

Nonetheless, for all scenarios in which the top performing model incorporated the lattice energy descriptor, the apparent performance enhancement over the corresponding model which did not incorporate the lattice energy descriptor was negligible (Fig. 5) and was never statistically significant. This remains the case when only those models not incorporating melting point or crystal structure based 3D descriptors are considered (Fig. 6). For all such scenarios, the increases in mean RMSE, upon removing the lattice energy descriptor, were around 0.03 kJ/mol and 0.00 (2dp) log units for predictions of enthalpy of solution and temperature dependent solubility respectively. The differences in mean R^2^ were around 0.00 (2dp).

Pairwise comparison of all relevant corresponding models identified only a minority of paired results, for both enthalpy of solution datasets and direct predictions of temperature dependent solubility (CV = rt), for which the inclusion of the lattice energy descriptor appeared to result in a statistically significant reduction in mean RMSE. Further discussion of the trends across all datasets is presented in Additional file 1 and the details for all pairwise comparisons are presented in Additional file 3.

### Effect of incorporating crystallographic information: 3D descriptors based on crystal structure

Only in the case of the Avdeef_ExDPs_CS_True dataset did the top performing model include the crystal structure based 3D descriptors (Tables 2, 3). However, upon removing the potentially confounding factors of the melting point and lattice energy descriptors, the best modelling results for Avdeef_ExDPs_CS_True and Klimenko_CS_True (CV = rt) were obtained using these descriptors (Additional file 3).

**Fig. 7:**
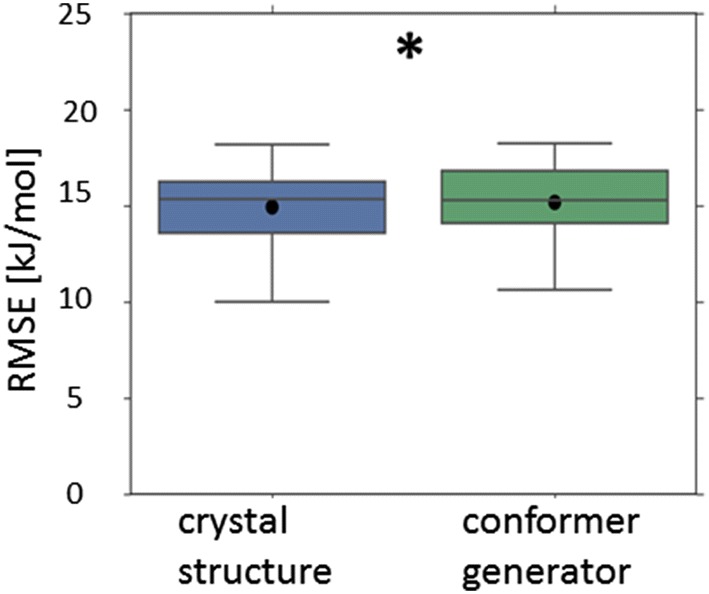
Cross-validated performance (RMSE) of the top performing model where the crystal structure based 3D descriptors were incorporated (LHS), compared to the corresponding model using the conformer generator based 3D descriptors (RHS): dataset = Avdeef_ExDPs_CS_True. All results are presented as per Fig. 5

**Fig. 8:**
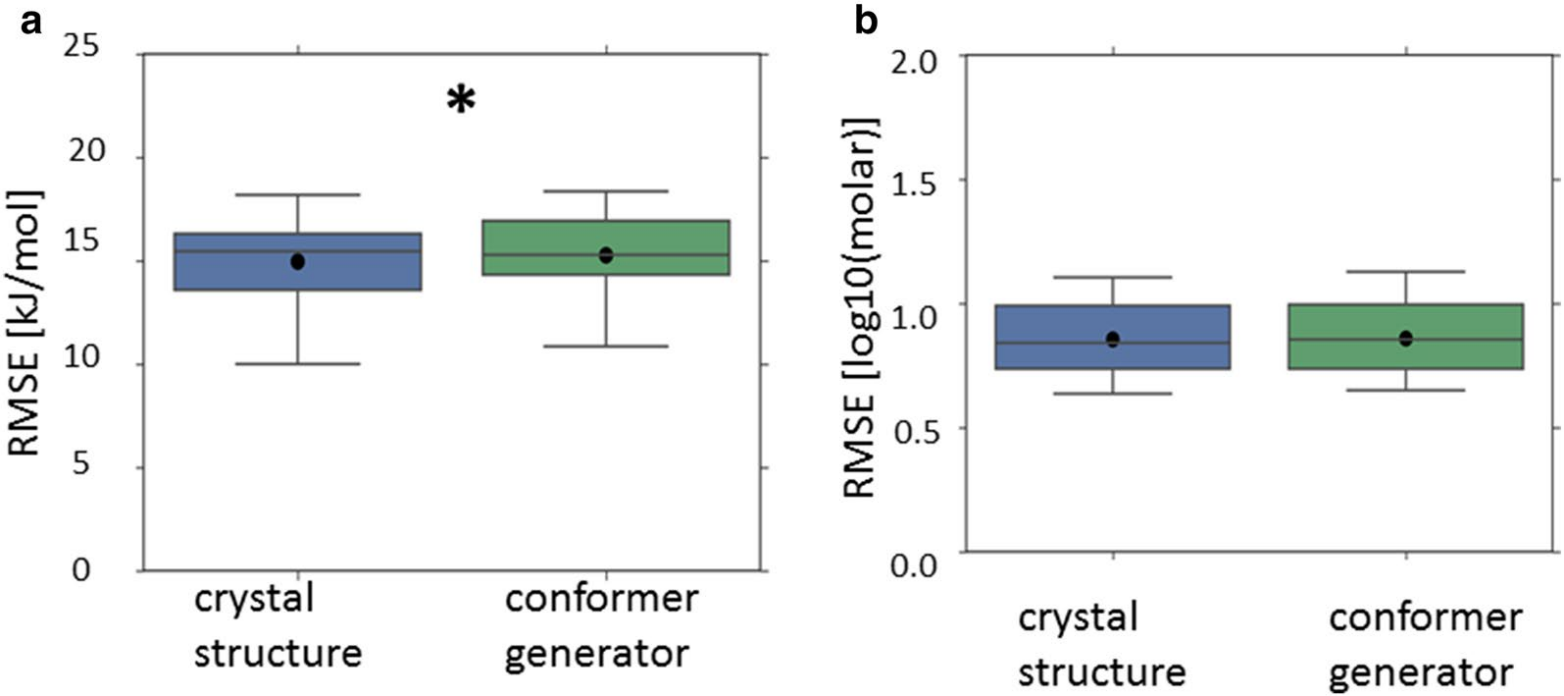
Cross-validated performance (RMSE) of the top performing models (excluding models incorporating the melting point or lattice energy descriptor) where the crystal structure based 3D descriptors were incorporated (LHS), compared to the corresponding model using the conformer generator based 3D descriptors (RHS): **a** dataset = Avdeef_ExDPs_CS_True; **b** dataset = Klimenko_CS_True, CV = rt. All results are presented as per Fig. 5

All of these results for the Avdeef_ExDPs_CS_True dataset (Figs. 7, 8) appeared statistically significantly different to the corresponding result obtained using the 3D descriptors based upon conformer generator structures. (This is in spite of the lattice energy descriptor also being incorporated into the overall top Avdeef_ExDPs_CS_True model.) This suggests the models may genuinely have benefited from the solid state information implicit in the 3D descriptors based upon the crystal structure. However, the same comparison for the top Klimenko_CS_True (CV = rt) model, after removing models with the lattice energy or melting point descriptor, found the difference in mean RMSE to the corresponding model using 3D descriptors based upon conformer generator structures appeared statistically insignificant.

Pairwise comparison identified only a minority of paired results, for one of the enthalpy of solution datasets (Avdeef_ExDPs_CS_True), for which the inclusion of crystal structure based 3D descriptors appeared to result in a statistically significant reduction in mean RMSE compared to the corresponding model using conformer generator based 3D descriptors. For the other enthalpy of solution dataset and direct prediction of temperature dependent solubility, no apparently significantly different results were obtained. Further discussion of the trends across all datasets is presented in Additional file 1 and the details for all pairwise comparisons are presented in Additional file 3.

### Effect of incorporating melting point

**Fig. 9:**
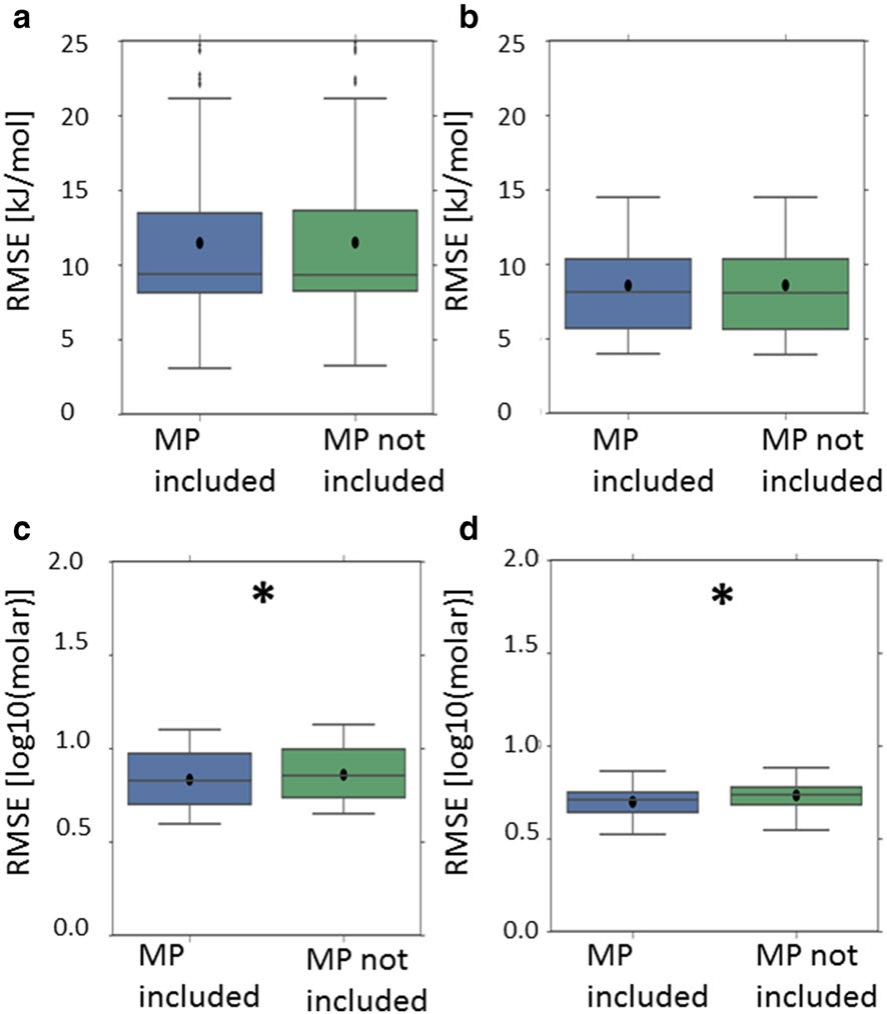
Cross-validated performance (RMSE) of the top performing models for all scenarios where they incorporated the melting point (MP) descriptor (LHS), compared to the corresponding model which didn’t include the MP descriptor (RHS): **a** dataset = Avdeef_ExDPs_Cal_CS_True; **b** dataset = Avdeef_ExDPs_Cal_CS_False; **c** dataset = Klimenko_CS_True, CV = rt; **d** Klimenko_CS_False, CV = rt. All results are presented as per Fig. 5

**Fig. 10:**
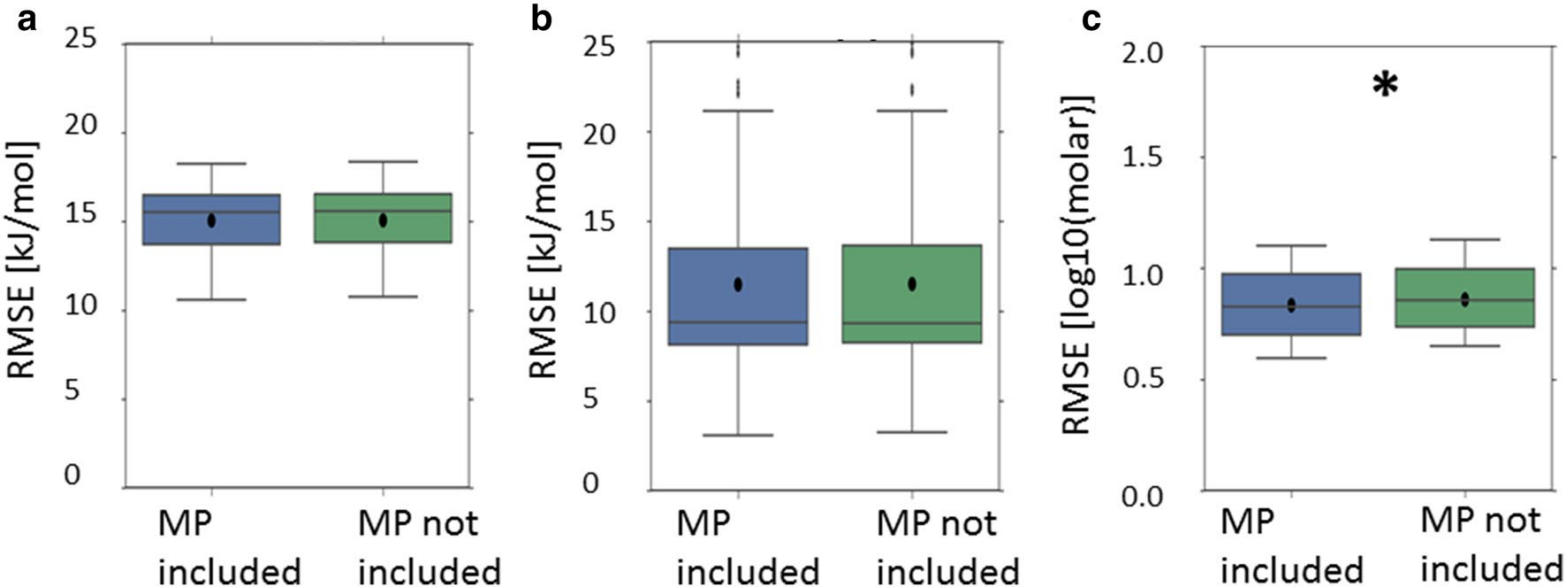
Cross-validated performance (RMSE) of the top performing models for all crystal structure integrated datasets, excluding models involving the lattice energy or crystal structure based 3D descriptors, where they incorporated the melting point (MP) descriptor (LHS), compared to the corresponding model which didn’t include the MP descriptor (RHS): **a** dataset = Avdeef_ExDPs_CS_True; **b** dataset = Avdeef_ExDPs_Cal_CS_True; **c** dataset = Klimenko_CS_True, CV = rt. All results are presented as per Fig. 5

For all relevant scenarios, save for models developed using the Avdeef_ExDPs_CS_False or Avdeef_ExDPs_CS_True datasets, the best performing models incorporated the melting point descriptor (Tables 2, 3). When models incorporating the other solid state contribution descriptors—i.e. the lattice energy and crystal structure based 3D descriptors—were excluded, the top models for all crystal structure integrated datasets incorporated the melting point descriptor. However, it should be noted that the apparent increase in best predictive performance upon incorporating the melting point descriptor was, at most, modest (Figs. 9, 10). Moreover, only the performance increases for the Klimenko_CS_False and Klimenko_CS_True datasets appeared statistically significant.

Pairwise comparison identified that, for a majority of scenarios, the inclusion of the melting point descriptor appeared to result in statistically significant enhancement in direct predictions of temperature dependent solubility. However, this was almost never observed from pairwise comparison of the models of enthalpy of solution. Further discussion of the trends across all datasets is presented in Additional file 1 and the details for all pairwise comparisons are presented in Additional file 3.

### Effect of feature selection

The application of the feature selection algorithm never yielded one of the top models as assessed according to any of the evaluation protocols. Hence, it cannot be claimed that feature selection improved the best predictive performance.

This is in keeping with the pairwise comparison of modelling protocols which differed only in terms of whether feature selection was employed. All such comparisons where feature selection appeared to improve the mean RMSE corresponded to scenarios in which MLR was applied in combination with the high dimensional combination of all 2D molecular descriptors. Indeed, all scenarios involving RFR indicated a reduction in predictive performance upon applying feature selection.

### Significance of the temperature descriptor

The importance of the (1/T) descriptor, in terms of its coefficient magnitude, was always close to the lowest for any descriptor for the evaluated MLR models. Conversely, for the evaluated models built using the non-linear RFR algorithm, the (1/T) descriptor was consistently in the top 20% of descriptors, excluding those models for which the molecular descriptors were based solely on the Absolv or Absolv and Ind (see Additional file 1: Table S1) or 3D descriptor sets.

These results can be explained by the van’t Hoff relationship (see Eq. 1), which posits that, if the standard enthalpy of solution is roughly constant over the relevant temperature (T) range, log10(solubility) should be linearly related to (1/T) for a given material, with the slope of the trend line being proportional to the standard enthalpy of solution. Hence, due to the variation in the standard enthalpy of solution across materials, a non-linear relationship will exist between (1/T) and log10(solubility) across materials. We found that the van’t Hoff relationship and the assumption that the standard enthalpy of solution is temperature independent holds well for most entries in the Klimenko et al. [14] derived datasets, for which an assessment was possible, and that the standard enthalpy of solution varied considerably across materials.

Detailed results supporting these comments are provided in Additional file 1.

### Significant molecular descriptors

Regarding the question of which sets of molecular descriptors yielded the most predictive models, it can be seen (Tables 2, 3) that the top models for enthalpy of solution or direct prediction of temperature dependent solubility, under different scenarios, were built using a variety of descriptor sets. Regarding the question of which individual molecular descriptors were found to be most important for the models, descriptor analysis suggested that no single molecular descriptor stood out as being consistently important, but the descriptors identified as most important could generally be rationalized in terms of the information they conveyed regarding the potential for specific kinds of solid state and/or solution state interactions.

Detailed results supporting these comments are provided in Additional file 1.

### Discussion of the main findings

The main findings from this work relate to the outcome of evaluating temperature dependent solubility models via a novel (CV = rt) cross-validation scheme and the effect of explicitly incorporating various kinds of solid state information into these models or models of the related enthalpy of solution endpoint. Here, we offer possible explanations for our findings and put them within the context of previous studies.

#### Standard cross-validation protocols can overestimate the performance of models of temperature dependent solubility

Our findings make clear that standard cross-validation protocols are likely to significantly overestimate the performance of models designed to estimate temperature dependent solubility for untested materials, by allowing solubility data points measured for the same material at slightly different temperatures to be placed in corresponding training and test sets. Of course, this scenario could still be a fair test for a model designed to interpolate solubility data measured for a given material at a couple of temperatures for other relevant temperatures. Nonetheless, we can recommend our modified (CV = rt) cross-validation scheme for scenarios in which the predictive performance of QSPR models for the temperature dependent solubility profile of untested materials are being evaluated.

#### Solid state descriptors based on crystallographic information or melting point data never substantially improved the best models of temperature dependent solubility or the related enthalpy of solution endpoint

Our results suggest that incorporating the lattice energy descriptor, calculated from the assigned crystal structure, may improve predictive performance for both temperature dependent solubility related endpoints under some scenarios. However, no statistically significant findings were obtained to indicate that this descriptor improves the best predictions of enthalpy of solution or the best direct predictions of temperature dependent solubility. This remained the case when only models without either of the other solid state descriptors were considered.

The inclusion of crystallographic information in the form of crystal structure based 3D descriptors may genuinely improve predictions of enthalpy of solution. However, only for one of the two enthalpy of solution datasets modelled were apparently statistically significant improvements in the best predictions, due to the incorporation of crystallographic information in this fashion, observed. These descriptors never appeared to statistically significantly enhance the best direct predictions of temperature dependent solubility. This remained the case when the other solid state descriptors were removed.

We found that the inclusion of a melting point descriptor almost never appeared to yield statistically significant improvements in predictions of enthalpy of solution. Indeed, this descriptor was never observed to result in statistically significant improvement in the best enthalpy of solution predictions. This remained the case when only those models not incorporating any other solid state descriptors were considered. Contrastingly, we found that the inclusion of the melting point descriptor appears to result in statistically significant performance enhancement for the best direct predictions of temperature dependent solubility. This was in keeping with the observation that the inclusion of the melting point descriptor often led to apparently statistically significant improvements in direct predictions of temperature dependent solubility.

However, even when apparently statistically significant improvements in the best predictions were observed, they were not substantial (Figs. 5, 6, 7 8, 9, 10). The failure of any of the solid state descriptors to substantially improve either the best direct predictions of temperature dependent solubility or the best predictions of the related enthalpy of solution endpoint, even when the other solid state descriptors were not included in the models, may be attributed to a variety of possible, non-mutually exclusive, explanations. (1) The variation in the endpoint data, for the investigated datasets, might be primarily dominated by variations in non-solid state contributions. (2) The solid state descriptors are insufficiently good at capturing the variation in solid state contributions. (3) The molecular descriptors, not including the crystal structure based 3D descriptors, implicitly capture the variation in solid state contributions to a considerable extent. Each of these possible explanations is considered in turn.

#### Do our findings reflect greater variation in non-solid state contributions for the modelled datasets?

If it were the case that non-solid state contributions to temperature dependent solubility, or the related enthalpy of solution, made a greater contribution to the variation in the solubility (or enthalpy) values for our datasets, this would suggest that being able to better capture the variations in solid state contributions would only lead to a modest improvement in predictive power for these endpoints. In practice, such a modest improvement might be sufficiently small to be deemed statistically insignificant. However, whether this could be expected to be the case for our modelled datasets is unclear. Recent experimental and computational studies have variously indicated that the variation in solubility across different public datasets was [26] and was not [48] dominated by non-solid state contributions. Moreover, we can only speculate on whether the relative importance of solid and non-solid state contributions to the variability in solubility suggested by these analyses is representative of the situation for the datasets studied in our work.

#### Do our findings reflect the limitations of the solid state descriptors?

It should be noted that there are two kinds of possible limitations to the ability of the solid state descriptors to capture solid state contributions to the modelled endpoints: (a) inherent limitations; (b) limitations arising from the possibility that the solid state descriptors were calculated or, in the case of melting point data, measured for the wrong polymorph. As discussed under “Integration with crystal structures”, a very small proportion of endpoint data points were annotated with the assessed polymorph and, typically, the predicted most stable available crystal structure was selected. It has previously been suggested that it is not essential to calculate lattice energies from the correct polymorph in order to predict solubility [49] and computational studies suggest most polymorph energies differ by less than 7 kJ/mol [50]. Nonetheless, the experimental solubilities of polymorphs may differ by around 0.60 log units (log10[molar]) [51]. (Elsewhere, higher apparent solubility differences between polymorphs are reported, although it is suggested that these differences are typically less than 1.0 log units (log10[molar]) [52].) However, given that whether the solid form corresponding to the solid state descriptors differs from the polymorph corresponding to the solubility or enthalpy of solution data modelled in the current work is typically unknown, we are unable to assess the extent to which non-inherent limitations of the solid state descriptors affect our findings.

The lattice energy descriptor obtained a Pearson’s correlation coefficient of 0.77 (one-tail *p* value = 10^−6^) with the experimentally estimated lattice energies for the 27 SUB-48 [29] dataset entries which complied with our filtering criteria. This confirms that the force-field protocol used to compute the lattice energy descriptor was a reasonable choice. If this statistic was representative of the performance of the lattice energy descriptor for the modelled datasets, the lattice energy descriptor should significantly capture the solid state contribution to the temperature dependent solubility related endpoints. Since the SUB-48 dataset was [29], like the datasets modelled in our work, a mixture of pharmaceutical APIs and general organic compounds and, as per most entries in our datasets, the calculated lowest energy crystal structure was assigned in the absence of polymorph specific information, this statistic could reasonably be expected to be representative of how the lattice energy descriptor would perform on the modelled datasets.

However, whilst experimental lattice energy estimates were not available for the modelled datasets, the correlation between the lattice energy descriptor and the available melting point data may be considered indicative of the extent to which the former captures the solid state contributions to the enthalpy of solution and temperature dependent solubility data. This can be seen from consideration of Eqs. (2), (3) [53] and (4), from which it can be expected that lattice energy and melting point are negatively correlated. (In Eqs. 3 and 4, $T_{m}$ denotes melting point, $\Delta H_{fus}$ and $\Delta S_{fus}$ the enthalpy and entropy of fusion respectively, $\Delta H_{sub}$ the sublimation enthalpy and $\Delta H_{cond}$ the condensation enthalpy.)

3 $$ T_{m} = \frac{{\Delta H_{fus} }}{{\Delta S_{fus} }} $$

4 $$ \Delta H_{fus} = \Delta H_{sub} + \Delta H_{cond} $$

**Fig. 11:**
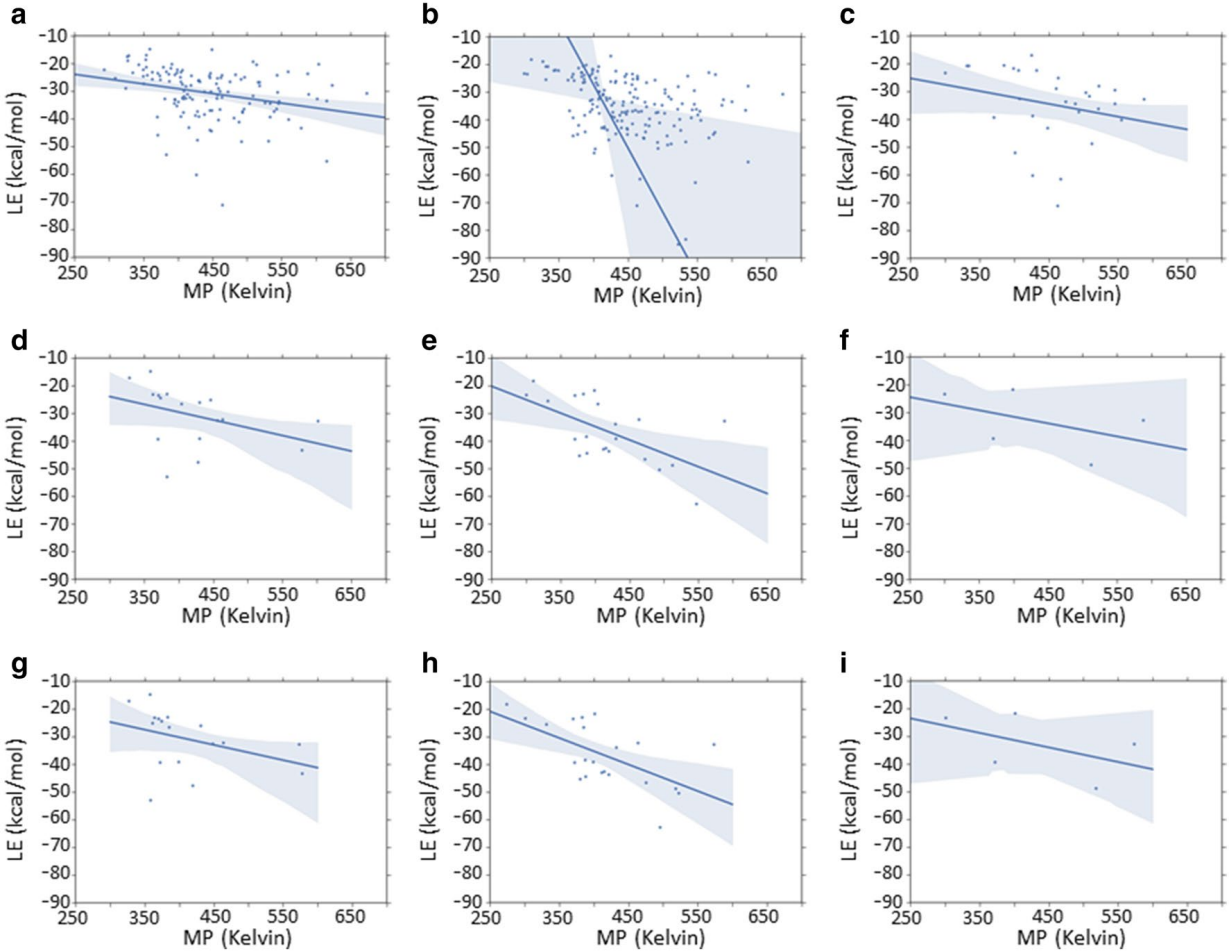
Correlation, in terms of the Pearson correlation coefficients (r) and one-tail *p* values (p), between all N corresponding pairs of lattice energy (LE) descriptor values and melting point (MP) values, from different sources, for different datasets: **a** Klimenko_CS_True dataset, melting point descriptor values (N = 129, r = − 0.29, *p* = 0.00); **b** Avdeef_ExDPs_CS_True dataset, melting point descriptor values (N = 169, r = − 0.15, *p* = 0.02); **c** Avdeef_ExDPs_Cal_CS_True dataset, melting point descriptor values (N = 30, r = − 0.24, *p* = 0.10); **d** Klimenko_CS_True subset with CSD melting point data, melting point descriptor values (N = 17, r = − 0.39, *p* = 0.06); **e** Avdeef_ExDPs_CS_True subset with CSD melting point data, melting point descriptor values (N = 22, r = − 0.61, *p* = 0.00); **f** Avdeef_ExDPs_Cal_CS_True subset with CSD melting point data, melting point descriptor values (N = 5, r = − 0.48, *p* = 0.21); **g** Klimenko_CS_True subset with CSD melting point data, CSD melting point data (N = 17, r = − 0.37, *p* = 0.07); **h** Avdeef_ExDPs_CS_True subset with CSD melting point data, CSD melting point data (N = 22, r = − 0.60, *p* = 0.00); **i** Avdeef_ExDPs_Cal_CS_True subset with CSD melting point data, CSD melting point data (N = 5, r = − 0.52, *p* = 0.19). N.B. (1) These one tail *p* values denote the probability of getting as negative a correlation coefficient as observed, by chance, given the null-hypothesis of zero correlation. (2) In order to make the plot legible, one outlier (CSD refcode TEPHTH13, calculated lattice energy − 2735.71 kcal/mol) was excluded from plot (b). (3) Where a range of melting points was retrieved for the specific crystal structure from the CSD, the mean value was used

Hence, the weak negative correlations between the lattice energy descriptor and the melting point descriptor could suggest the lattice energy descriptor did not capture solid state contributions well for the entirety of these datasets (Fig. 11a–c). Moreover, the fact that these correlations were observed to increase when only the subset of entries for which crystal structure specific melting point data were available was considered (Fig. 11d–f) could further suggest that the lattice energy descriptor was not uniformly good at capturing solid state contributions for all entries in the modelled datasets. (Here, it should be noted that around 2% of the Avdeef_ExDPs_CS_True and Klimenko_CS_True crystal structures were disordered. However, this was only observed to have caused lattice energy calculation errors for one structure in the Avdeef_ExDPs_CS_True dataset. Full details are provided in Additional file 1.) The fact that those correlations were negligibly changed when the actual crystal structure specific melting points, retrieved from the CSD (version 5.38) using the CSD Python API [54], were used (Fig. 11g–i) suggests that the poor correlations observed between the lattice energy descriptor and the melting point descriptor across the entirety of the modelled datasets did not reflect the fact that the melting point data used for the latter descriptor may not have corresponded to the polymorph for which the lattice energy descriptor was calculated.

Nonetheless, it should be noted that an imperfect correlation would still be expected between the lattice energy descriptor and the crystal structure specific melting point, even if the former corresponded perfectly to the true lattice energy and there were no experimental errors in the latter. Melting point will be imperfectly correlated with the enthalpy of fusion, due to the variation in the entropy of fusion across materials (Eq. 3). The enthalpy of fusion, in turn, will be imperfectly correlated with the enthalpy of sublimation, due to the variation in the condensation enthalpy across materials (Eq. 4), and the latter, in turn, is only linearly related to the lattice energy under certain assumptions [29] and at constant temperature (Eq. 2). Hence, also taking into account the possibility that the melting point descriptor may correspond to a different polymorph, the poor correlation of the lattice energy descriptor with the melting point descriptor across the modelled datasets may overstate the extent to which the lattice energy descriptor fails to capture the solid state contribution to the modelled endpoints.

The fact that the melting point descriptor did not necessarily correspond to the experimental melting point for the polymorph for which enthalpy of solution or temperature dependent solubility data were available may have contributed to this descriptor failing to fully capture the solid state contribution to the modelled endpoints. Consideration of the previously mentioned subset of dataset entries for which crystal structure specific melting point data were retrieved from the CSD, indicates that melting point data for the same chemical can differ significantly in some cases. (Full details are provided in Additional file 3. Here, it should also be noted that we cannot be certain that these discrepancies in melting point data from different data sources necessarily reflected melting point differences between polymorphs.) For the Klimenko_CS_True dataset, across all 17 pairs of corresponding CSD retrieved melting points and melting point descriptor values, the median absolute deviation was 1.5, the 95th percentile value was 41.4 and the maximum value was 85 degrees Kelvin. The corresponding statistics for the 22 (5) pairs of melting point values obtained for the Avdeef_ExDPs_CS_True (Avdeef_ExDPs_Cal_CS_True) dataset were as follows: median = 1.75 (2), 95th percentile = 35.9 (12.5), maximum = 52.2 (14.2) degrees Kelvin.

Finally, regarding the inherent limitations of the solid state descriptors considered in this work, the crystal structure based 3D descriptors would not be able to fully capture all information relevant to lattice interactions. These CPSA descriptors [39–41] capture information related to polar and non-polar intermolecular forces, based on electrostatic distributions at the molecular surface, and a CPSA descriptor, calculated from the molecular structure via a conformer generator, was amongst those making a significant contribution to the enthalpy of sublimation model of Salahinejad et al. [53]. However, these descriptors would have been unable to take account of dispersion forces or properly take account of localized interactions such as hydrogen bonding. Indeed, they are indicated to be weakly dependent on molecular conformation [39, 40].

Future studies should consider computing additional 3D descriptors, based upon the crystal structure, which more fully account for those interactions. Ideally, these would explicitly take account of the lattice structure. This would arguably result in them better taking account of the actual solid state intermolecular interactions than crystal structure based 3D molecular descriptors, which can only implicitly take account of that information. However, as per our current work, if 3D molecular descriptors were computed using the crystal structure, they would need to be benchmarked against the performance of the same 3D descriptors computed without knowledge of the crystal structure, using a conformer generator.

#### Do our findings reflect the ability of the molecular descriptors to implicitly capture solid state contributions?

It should be acknowledged that molecular descriptors cannot capture variations in solid state contributions arising from polymorphism. Hence, decent solid state descriptors, calculated or measured for the solid form for which the endpoint data were measured, would be expected to add additional information that molecular descriptors cannot capture. However, as previously discussed, the experimentally assessed polymorph was not typically known for the modelled datasets.

Under this scenario, molecular descriptors may be just as good at capturing solid state contributions to the enthalpy of solution or temperature dependent solubility as the solid state descriptors. The extent to which molecular descriptors can capture solid state structural information may be illustrated by the reasonable quality of models built for the lattice energy descriptor using the combined set of 2D molecular descriptors and random forest for the Klimenko_CS_True dataset. The cross-validated mean R^2^ was 0.60 ± 0.01 (standard error of the mean).

The lower performance for the Avdeef_ExDPs_Cal_CS_True dataset (mean R^2^ = 0.40 ± 0.04) may reflect the fewer data points available for training. However, the poor cross-validation results obtained for the Avdeef_ExDPs_CS_True dataset (mean R^2^ = − 172.20 ± 26.56) are surprising. This arguably reflects the presence of some problematic instances distorting the results. For three out of five cross-validation repetitions, all R^2^ values were negative whilst, for two repetitions, a single fold yielded R^2^ values between 0.70 and 0.78. Nine instances were identified with absolute predictions errors greater than 50 kcal/mol. (These instances are identified in Additional file 1. Other than the fact that one of them, CSD refcode TEPHTH13, was an extreme lattice energy outlier of − 2735.71 kcal/mol, there was no obvious reason for them being prediction outliers.) When the modelling results were generated again without these instances, a much better mean R^2^ (0.76 ± 0.01) was obtained.

Descriptor importance analysis suggested partial consistency between the most important molecular descriptors, for predictions of calculated lattice energies, across all datasets. The common most important descriptors are expected to have a close link to solid state intermolecular interactions. Further details are provided in Additional file 1.

#### How do our findings regarding the importance of solid state descriptors compare to previous modelling studies of related endpoints?

Both our findings regarding the effect of incorporating the crystal structure derived descriptors (calculated lattice energy or 3D descriptors) and/or the melting point descriptor into our models for temperature dependent solubility, and the related enthalpy of solution endpoint, should be seen in the context of the wider debate in the recent literature regarding the importance of explicitly representing solid state contributions in models of aqueous solubility and the extent to which molecular descriptors can capture solid state contributions [3, 26–30]. Emami et al. [27] found that two parameter QSPR models for aqueous solubility incorporating experimental melting point as a descriptor did not perform better than two parameter models based solely on molecular descriptors. (However, their results did suggest that a two parameter model incorporating the entropy of melting outperformed molecular descriptor models.) This is consistent with our finding that the inclusion of a melting point descriptor within molecular descriptor based models was not responsible for statistically significant improvements in the best predictions of the related enthalpy of solution endpoint. However, it is possibly at odds with our finding that melting point data did result in an apparently statistically significant improvement in the best direct predictions of temperature dependent solubility. Nonetheless, it should be remembered that even these apparently statistically significant improvements were not substantial.

In keeping with our finding, that incorporating a lattice energy descriptor did not lead to a statistically significant improvement in the best model for temperature dependent aqueous solubility or the related enthalpy of solution, Salahinejad et al. [3] found that the availability of lattice energy or sublimation enthalpy descriptors did not significantly improve models of aqueous solubility. However, whilst those authors [3] used sublimation enthalpies, converted to lattice energies as per Eq. (2), estimated from molecular structure [53], our own analysis was based on lattice energies estimated from crystal structures. The fact that we obtained similar findings might suggest their results were not an artefact of failing to incorporate crystallographic information into their models. On the other hand, the fact that we needed to assign a nominal crystal structure in many cases, due to the few data points associated with polymorph information in our dataset, might be a contributory factor to this finding.

In contrast to our findings, McDonagh et al. [30] suggested that random forest models of aqueous solubility were statistically significantly improved upon adding theoretical descriptors, calculated in part from crystal structures assigned using a similar protocol to our own, to molecular descriptors. However, it should be noted that the theoretical descriptors calculated by these authors [30] were a combination of solid state energetic contributions, calculated from crystal structures using the DMACRYS program [55], and non-solid state contributions, computed using Hartree–Fock or MO6-2X calculations, and they only reported statistically significant improvements in their models when those theoretical descriptors were calculated using MO6-2X calculations. This suggests that the theoretical descriptors capturing the non-solid state contributions may have been most important here. (It should also be noted that their assessment of statistically significant differences was not identical to the protocol employed herein.) Hence, their findings are not *necessarily* at odds with our own observation that incorporating lattice energy descriptors, calculated from crystal structures, do not statistically significantly improve the best QSPR models of temperature dependent aqueous solubility or the related enthalpy of solution.

As regards our suggestion that this finding reflects, in part, the ability of molecular descriptors to serve, to a considerable degree, as proxies for solid state contributions, various recent studies have considered the extent to which molecular descriptors can capture solid state contributions to solubility [26, 28, 29, 53]. Both Salahinejad et al. [53] and Docherty et al. [26] reported that molecular descriptor based QSPR models could capture most of the variation in enthalpy of sublimation data for diverse organic compounds, with test set R^2^ values > 0.90. However, Abramov [28] recently suggested that the failure of molecular descriptors to fully capture solid state contributions was the major limiting factor in the prediction of aqueous solubility using QSPR methods and that the good performance reported for molecular models of enthalpy of sublimation could represent their ability to capture short range molecular interactions in the solid state, as opposed to long range interactions within the crystal. Furthermore, even for enthalpy of sublimation, McDonagh et al. [29] found that QSPR models built using theoretical chemistry descriptors, calculated from crystal structures, appeared substantially more predictive than models built using molecular descriptors, albeit for the relatively small SUB-48 dataset.

Hence, these earlier studies support the hypothesis that incorporating crystallographic information should be able to capture solid state contributions to solubility and its temperature dependence better than simply using molecular descriptors. This may be reflected in the fact that our results offer some evidence that the incorporation of this information, in the form of crystal based 3D molecular descriptors, may genuinely improve the best QSPR models of the related enthalpy of solution term. However, the fact that our results do not suggest statistically significant improvement, upon incorporating calculated lattice energies, in the best predictions of either temperature dependent solubility related endpoint could well reflect, in part, the limited extent to which our lattice energy calculations capture solid state contributions above and beyond the degree to which this is captured by molecular descriptors. In part, this may reflect greater discrepancy between the polymorphs for which endpoint data were available and for which the lattice energies were calculated, compared to some previous studies, albeit McDonagh et al. [29, 30] were obliged to handle missing polymorph data in much the same way as per our current work. It may also reflect, as suggested by our correlation analyses of melting point data and calculated lattice energies (Fig. 11), variations in the performance of the lattice energy calculations for different subsets of the modelled datasets.

## Conclusions

In this work, we have built upon the few QSPR studies published to date which have explored the prediction of temperature dependent solubility. Specifically, we have extended previous work looking at modelling the enthalpy of solution, which can be related to temperature dependent solubility via the van’t Hoff relationship, and direct prediction of temperature dependent solubility for aqueous solutions. We built upon these earlier studies via investigating the following factors: (a) the incorporation of crystallographic information, in the form of lattice energies or 3D descriptors calculated from crystal structures, into the models; (b) the effect of adding versus excluding melting point data from the models; (c) a larger variety of molecular descriptor permutations; (d) the use of feature selection to produce parsimonious models; (e) a novel pseudo-cross-validation protocol.

All the different descriptors of solid state contributions (crystal structure calculated lattice energies, crystal structure based 3D descriptors, melting point data) were indicated to improve the models for at least one of the modelled endpoints for some scenarios. However, none of these descriptors was responsible for any substantial improvement in the best direct predictions of temperature dependent solubility or the best predictions of the related enthalpy of solution endpoint. This remained the case when the effect of one kind of solid state descriptors was considered in isolation. This finding is noteworthy and surprising, since the importance of the solid state contribution to both endpoints is clear from the underlying thermodynamics and, since a variety of solid state arrangements are possible for the same molecular structure, molecular descriptors are unlikely to fully capture this contribution. Indeed, it has recently been suggested that the major source of error in QSPR prediction of solubility is the failure of molecular descriptors to fully capture solid state contributions. This finding may, in part, reflect limitations in the calculated 3D descriptors and lattice energies. In the case of the lattice energies, correlation analysis with melting point data suggests that the quality of the lattice energy calculations varies across different dataset entries. Our findings may also, in part, reflect the limited availability of polymorph metadata. Both of these reasons may have contributed to molecular descriptors implicitly capturing solid state contributions to the modelled endpoints comparably to the solid state descriptors for at least some of the scenarios considered, limiting the value added by incorporating the solid state descriptors.

Our best modelling results were typically comparable to those previously reported in the literature, albeit we cannot claim to have performed a perfectly like-for-like comparison, partly due to refinements we made to the previously modelled datasets. We found that feature selection, as applied in our work, never improved the best modelling results.

Finally, we found that, for direct prediction of temperature dependent solubility data, standard cross-validation protocols tend to overestimate the performance of models designed to predict temperature dependent solubility for untested materials, by allowing solubility data points measured for the same material at slightly different temperatures to be placed in corresponding training and test sets. Hence, we recommend the use of our novel pseudo-cross-validation protocol, which avoids including data points measured for the same material at different temperatures in corresponding training and test sets.

## Additional files

**Additional file 1.** Extensions of the Methods and Data section (section A), step-by-step instructions for reproducing our results using the datasets and source code we have made available (section B) and detailed comparisons to results reported in the literature, along with further details regarding our results (Section C).

**Additional file 2.** All QSPR ready datasets, prior to feature selection, associated with all considered sets of descriptors. N.B. In the case of the temperature (T) values, these were replaced with (1/T) using additional scripts prior to both feature selection and modelling.

**Additional file 3.** Additional results files, in electronic format. These additional results are (a) SUB-48 calculated lattice energies for the complete set and filtered set of 27 crystal structures, (b) results from analysis of the correspondence between our temperature dependent solubility data and (1/T), (c) Excel workbooks documenting all R^2^ and RMSE values obtained from cross-validation, their mean values and the *p* values (both raw and adjusted) obtained from pairwise comparisons of corresponding models, (d) comparison of melting point data used for the melting point descriptor and retrieved from the CSD for linked refcodes.

**Additional file 4.** The “QSPR Data Processing Tools Suite”, used by the scripts employed to generate all QSPR results.

**Additional file 5.** An automated test suite for “QSPR Data Processing Tools Suite”.

**Additional file 6.** The “Feature Selection Tool”.

**Additional file 7.** The Perl script used for Materials Studio lattice energy calculations, along with the Python scripts used to generate CIF input files from CSD refcodes, including filtering, as well as the Python scripts used for evaluation of the lattice energy protocol on the SUB-48 dataset.

**Additional file 8.** The curated temperature dependent solubility and enthalpy of solution datasets, including the calculated lattice energies, and the SUB-48 dataset.

**Additional file 9.** All scripts used to generate QSPR input files, modelling results and perform analysis.

**Additional file 10.** SMARTS patterns used by one of the QSPR input file scripts (see Additional file 1), which were adapted from the Rdkit documentation [56].

## Abbreviations

RFR: random forest regression
MLR: multiple linear regression
API: active pharmaceutical ingredient
Python API: Python Application Programming Interface
