# Supplementary material for: The influence of solid state information and descriptor selection on statistical models of temperature dependent aqueous solubility
Source: J Cheminform. 2018 Aug 29;10:44. doi: 10.1186/s13321-018-0298-3 (PMC6115327; doi:10.1186/s13321-018-0298-3)
Supplement: Supplementary file 1 — Additional file 1. Extensions of the Methods and Data section (section A), step-by-step instructions for reproducing our results using the datasets and source code we have made available (section B) and detailed comparisons to results reported in the literature, along with further details regarding our results (Section C). [file 13321_2018_298_MOESM1_ESM.pdf]

Supporting Information for “The Influence of Solid State Information and Descriptor Selection on Statistical Models of Temperature Dependent Aqueous Solubility”

Richard L. Marchese Robinson,[a] Kevin J. Roberts,[a] Elaine B. Martin[a,*]

a. School of Chemical and Process Engineering, University of Leeds, Leeds LS2 9JT, United Kingdom

*Corresponding author. E-mail: E. Martin@leeds.ac.uk

Contents

Supporting Information for “The Influence of Solid State Information and Descriptor Selection on Statistical Models of Temperature Dependent Aqueous Solubility” 1

Section A: Extended Methods and Data 4

Solubility data curation 4

Integration with molecular structures 7

Integration with crystal structures 8

Calculation of lattice energies 11

Preparation of molecular structures for descriptor calculations 12

Calculation of 2D molecular descriptors 13

Calculation of crystal structure based 3D molecular descriptors 15

Melting point descriptor 16

Feature selection 17

Descriptor scaling 18

Machine Learning 19

Validation statistics 19

Cross-validation protocols 19

Statistical significance of differences in cross-validated results 20

Filtering of SUB-48 Dataset 24

Computational details 24

Section B: How to Reproduce Our Results 26

Evaluating the lattice energy protocol on the SUB-48 dataset 26

Calculating lattice energies for the solubility and enthalpy of solution datasets 27

Generating QSPR modelling results 28

Running analysis of modelling results and datasets 31

Section C: Extended Results and Discussion 33

Comparison to the literature 33

Additional Plots of Cross-Validated Results for the Best Models 36

Effect of incorporating the lattice energy descriptor: pairwise comparison of models 40

Effect of incorporating the 3D descriptors based on crystal structure: pairwise comparison of models 41
Effect of incorporating melting point: pairwise comparison of models 42
Significance of the temperature descriptor 43
Significant molecular descriptors 50
Lattice energy molecular descriptor models: prediction outliers 51
Lattice energy molecular descriptor models: descriptor importance analysis 52
References 53

Section A: Extended Methods and Data

All subsections below are extensions of the subsection of Methods and Data in the main text with the same sub-heading.

Solubility data curation

All data were curated according to a common Excel workbook template. This template was designed to capture data for endpoints related to temperature dependent solubility, along with relevant metadata. The solubility data template metadata fields included the following: important experimental conditions (e.g. temperature and, for total as opposed to intrinsic solubility data points,[1] pH); identifiers of solute molecular (chemical name and CAS number) structure and descriptions of crystal structure (free text descriptions of polymorph identity, where available); solvent description; experimental technique; units of measurement. Additional sheets in the workbook were designed to link the molecular identifiers and crystal structure descriptions to SMILES representations of molecular structure ("SMILES" sheet) and refcodes denoting crystal structure entries ("CrystalStructures" sheet) from the Cambridge Structural Database (CSD) [2], as is further explained under "Integration with molecular structures" and "Integration with crystal structures".

In the template, the enthalpy of solution endpoint is described as the "standard enthalpy of dissolution", referring to the enthalpy change associated with movement of solute molecules from the crystal lattice to a solution of standard concentration [3]. N.B. The terms "enthalpy of solution" and "enthalpy of dissolution" may be used interchangeably [4, 5].

Where possible, we wanted to link each solubility, or enthalpy of solution, value to a specific "material" identity, defined in terms of a unique combination of molecular identifier (name and/or CAS number) and crystal identity (polymorph description). The "material" identity

would then be linked (see "Integration with crystal structures") to a CSD refcode, where possible. The molecular identity (i.e. name and/or CAS number) would be linked to a SMILES representation of molecular structure. In order to achieve this, it was necessary to iteratively update the metadata originally curated from the publications of Avdeef [4] and Klimenko et al.[6]

Following curation of the data and metadata extracted from their publication, the solubility data points taken from Klimenko et al.[6] were then linked to names, for those cases where it was not possible to retrieve the corresponding molecular structures based on the CAS number (see "Integration with molecular structures"), via cross-referencing the CAS number taken from Klimenko et al.[6] against the Handbook of Aqueous Solubility Data (2nd edition) [7]. (N.B. Klimenko et al.[6] curated data from the 1st edition of the Handbook of Aqueous Solubility [8], whereas Avdeef [4] and ourselves referred to the 2nd edition [7].) Where multiple names were linked to the same CAS number, an arbitrary selection of a single synonym was made. The data points were then linked to polymorph descriptions, in the few cases where this was possible, via cross-referencing unique combinations of solubility value - CAS number - temperature, as reported in Klimenko et al. [6], to the corresponding combinations reported in the Handbook of Aqueous Solubility Data (2nd edition) [7]. In a few cases, we spotted that this cross-referencing failed to retrieve polymorph descriptions due to apparent conversion errors, beyond basic rounding errors, between Handbook of Aqueous Solubility Data (2nd edition) [7], where solubility was reported in grams/L, and Klimenko et al. [6], where solubility was reported in log10(molar) units. The corresponding dataset entries were deleted.

In the course of retrieving molecular structures (see "Integration with molecular structures"), it was determined that some of the CAS numbers ("121-14-3", "2588-04-07") reported by Klimenko et al.[6] were not valid, according to both the Common Chemistry^{TM} online resource [9] and their absence from the 2nd edition of the Handbook of Aqueous Solubility Data [7].

Hence, the corresponding dataset entries were deleted. In one case, it was spotted that there was a mismatch between one of the CAS number – name combinations documented in the Handbook of Aqueous Solubility Data (2nd edition) [7] and the information provided by the Common Chemistry online resource [9]. This combination was "87-78-5" and "d-Mannitol". Hence, all dataset entries corresponding to the CAS number "87-78-5" were deleted.

The initially curated SMILES (see "Integration with molecular structures") were then used, via converting them directly to InChI [10] values using Pybel [11–14], to identify names (Avdeef derived dataset) [4] or CAS numbers (Klimenko et al.[6] derived dataset) matched to identical structures. The resulting combinations of names were reviewed, partially via consulting Common Chemistry™ [9], to identify synonyms, or otherwise used to identify initially erroneous SMILES, which were corrected. The names of synonyms were merged and the merged names replaced the corresponding original names in the datasets, e.g. "3-nitrobenzoic acid_OR[SYNONYM]_Benzoic acid, 3-NO2-" replaced "3-nitrobenzoic acid" and "Benzoic acid, 3-NO2-". Other merged names were created by supplementing the name reported by Avdeef [4] with synonyms retrieved, for the purpose of identifying molecular structures, via consulting the Handbook of Aqueous Solubility Data (2nd edition) [7]. Full details of the data point specific iterative updates to the datasets are documented in the appropriate comment fields within the datasets.

Dataset entries where the SMILES retrieved (see "Integration with molecular structures") was identified as incorrect (or probably incorrect), and challenges were encountered when trying to confirm the correct structure, were deleted.

Finally, since we were concerned with the temperature dependent solubility of crystalline materials, it was necessary to exclude dataset entries where there was no evidence that the solubility data (or enthalpy of solution data) corresponded to dissolution from the solid state. (None of the data appeared to correspond to amorphous forms, based on consulting the work

of Avdeef [4], Klimenko et al.[6] and the Handbook of Solubility Data (2nd edition) [7].) Hence, as the enthalpy of solution values taken from the work of Avdeef [4] were described as corresponding to 25 °C, any dataset entries where the experimental melting points taken from Avdeef [4] were less than or equal to 25 °C were deleted. In the case of the temperature dependent solubility values taken from the work of Klimenko et al.[6], all dataset entries were deleted where the melting point (or lower limit) reported in the Handbook of Solubility Data (2nd edition) [7], for the corresponding CAS number, was less than or equal to the temperature at which solubility data were measured. Any dataset entries where an experimental melting point (or lower limit) was not obtained were also deleted.

Integration with molecular structures

In the first instance, the names and CAS numbers, retrieved from Avdeef [4] and Klimenko et al.[6] respectively, were used to retrieve SMILES representations of molecular structures via using each identifier (i.e. name or CAS number) as a query term for the following online resources, via their respective Application Programming Interfaces (APIs): the Chemical Identifier Resolver service [15] (via the CIRpy Python module) [16], ChemSpider [17] (via the ChemSpiPy Python module) [18] and PubChem [19, 20] (via the PubChemPy module) [21]. For those scenarios where no SMILES was retrieved, other references were consulted to determine the molecular structures. In some cases, the Handbook of Aqueous Solubility Data (2nd edition)[7] was consulted to determine a synonym which could be used as a query term for the previously described online resources. For the Klimenko et al.[6] derived dataset, the names retrieved as explained under "Solubility data curation" were used as query terms. In some cases, the primary literature was consulted. Full, data point specific, details are provided in the appropriate comment fields within the datasets.

For those scenarios where multiple SMILES were retrieved, for a given name or CAS number, which were not identified as the same structure via converting them to InChI [10] values using Pybel [11–14], other references were consulted to determine the correct structure. In the first instance, the Common Chemistry™ online resource [9] was used to check each of the retrieved SMILES via visualizing them using the Chemicalize™ [22] or CDK Depict [23] online tools. Other references which were consulted included the primary literature, databases such as the Protein Data Bank (PDB)[24, 25] and the CSD [2] (version 5.37), as well as chemical supplier websites. Again, full, data point specific, details are provided in the appropriate comment fields within our datasets. As is documented in those comments, stereochemical information was missing or was uncertain for a few SMILES strings. However, we checked that this did not affect integration with crystal structures (see "Integration with crystal structures"), i.e. any such SMILES strings were required to match based upon the associated names, rather than just the molecular structure.

Integration with crystal structures

The CSD (version 5.37) [2] was queried using the CSD Python API (version 1.0.0) [26], made available by the Cambridge Crystallographic Data Centre (CCDC). Prior to finding provisional matches, between CSD entries and entries in our datasets, the Python API was used to filter the CSD database entries according to the following criteria: z' <= 1;[27, 28] only organic structures; no disordered structures (a few disordered structures still passed the filter – see below); no errors; has 3D co-ordinates; no powder structure determinations; no polymeric structures; no ionic structures (other than zwitterions); R factor <= 5.0. N.B. This was achieved by explicitly filtering on *entry.z_prime* and setting up a *Search.Settings* instance with the following options: only_organic = True, no_disorder = True, no_errors = True,

has_3d_coordinates = True, no_powder = True, not_polymeric = True, not_ions = True, max_r_factor = 5.0. N.B. The following QSPR ready dataset entries, identified via their CSD refcodes, still contained disordered atoms, which were removed when generating input structures for the crystal structure based 3D descriptors, but which could have caused errors in the calculated lattice energies: Avdeef_ExDPs_CS_True (TEPHTH13, DMANTR01, PIMELA14), Klimenko_CS_True (KUSVEZ02, MPARAT01, DMANTR01). However, only one of these (TEPHTH13) was observed to have been processed improperly during calculation of lattice energies, leading to an abnormally negative calculated lattice energy.

The molecular chemical identifiers (i.e. names and CAS numbers) reported in our datasets were compared to the corresponding chemical identifiers retrieved for each CSD entry in the filtered subset. (The CSD entry chemical identifiers compared were the entry.chemical_name and list of entry.synonyms retrieved via the Python API.) For a CSD entry to be matched based on names, only one of the chemical identifiers, following conversion to lower case and, in the case of CSD chemical identifiers, plain text,[29] needed to match.

The corresponding molecular structures, recorded as SMILES, in our datasets were compared to the CSD entry molecular structure reported via the entry.molecule.to_string('sdf') string retrieved using the Python API. This comparison was achieved via converting both structural representations to InChIs [10] using Pybel [11–14].

For all scenarios in which these InChIs [10] matched, the corresponding CSD refcode was accepted as a provisional match. For scenarios in which the names matched, yet the InChIs [10] did not, the original SMILES in our dataset, as visualized via CDK Depict [23], and the molecular structure displayed upon searching the CSD (version 5.37) in the Mercury (version 3.8) GUI [30], using the refcode of the provisional match, were compared. Matches in which the only differences related to multiple copies of the same molecule being displayed via Mercury, implicit vs. explicit hydrogen atoms, tautomerism or stereochemistry were accepted.

(Tautomeric forms were standardized and stereochemical information was removed during processing of our SMILES prior to calculating 2D molecular descriptors, as described under "Preparation of molecular structures for descriptor calculations".) In other cases, it was deemed the case that the original SMILES in our dataset was incorrect and this was replaced with a SMILES based upon the structure shown in the Mercury GUI.

These provisional matches were manually filtered where the polymorphic information was contradictory, for the few cases where this information was available in our dataset and from the provisional CSD entry match, via entry.polymorph retrieved using the Python API. (For the Klimenko et al.[6] derived dataset, 9/882 data points were associated with polymorph information. For the Avdeef [4] derived dataset, this proportion was only slightly higher: 13/558.) In some cases, it was necessary to consult the literature to ascertain whether certain polymorph descriptions were consistent, e.g. to determine that "form I" and "trigonal polymorph i" referred to the same polymorph of Barbital [31]. Clearly, making these judgements is non-trivial, especially in light of the potential inconsistency in polymorph labels across the literature [32]. Full, data point specific, details are provided in the appropriate comment fields within our datasets.

Since the entry.molecule.to_string('sdf') string used for InChI based matching (see above) corresponds to the crystal chemical unit, which is the expansion of the asymmetric unit to include whole molecules, this only returns a single enantiomer in the case of racemic crystals. Hence, InChI based matching could erroneously match the SMILES for an enantiopure dataset entry and the CSD refcode for a racemic crystal. For this reason, where analysis of the provisional refcode matches using the CSD Python API (version 1.3.0) [33], querying CSD version 5.38, indicated a racemic crystal was retrieved, this match was deleted. Similarly, where the dataset notes associated with the curated SMILES indicated absolute stereochemistry

was recorded in the SMILES, even though the compound was believed to be a racemate, corresponding CSD refcode matches were deleted.

In keeping with literature precedence [34], any remaining multiple CSD refcode matches, for a given combination of compound name – CAS number – polymorph description (with the latter most commonly being blank), were filtered via selecting the structure predicted to be most stable. Here, the structure predicted to be most stable was that with the most negative calculated lattice energy. (See "Calculation of lattice energies".) CSD refcodes where the lattice energy calculation failed or where additional analysis, based on the Python API, indicated the structure did not contain at most one molecule in the asymmetric unit and, hence, the lattice energy calculation protocol was not applied (see "Calculation of lattice energies") were filtered prior to this ranking. Finally, for a given CSD refcode provisional match associated with polymorph information in the original dataset, any additional occurences of this CSD refcode, with the same combination of compound name and CAS number where the polymorph metadata field was blank, were deleted, as were any refcodes associated with positive lattice energies. Hence, only unique refcode occurrences were retained.

Calculation of lattice energies

All calculations were performed within Materials Studio [35], using a Perl script ran via the "Run on Server" command from the GUI. Since we restricted our consideration to single component molecular crystals, with at most a single molecule in the asymmetric unit [28], the lattice energy was calculated as per equation (i). In equation (i), E_{latt} denotes the lattice energy in kcal/mol (per mole of gas phase molecule), $E_{crystal}$ denotes the potential energy of the static crystal structure in kcal/mol (per mole of unit cell), E_{gas} denotes the potential energy of a

single, static gas phase molecule in kcal/mol (per mole of gas phase molecule) and N denotes the number of molecules in the unit cell.

$$E_{latt} = \frac{E_{crystal}}{N} - E_{gas} \text{ (i)}$$

Crystal structures were generated, in CIF file format [36], as input for these calculations from the available CSD refcode, via querying the CSD (version 5.37) using the CSD Python API (version 1.3.0) [33]. Since our calculations relied on the assumption of a single component crystal with at most a single molecule in the asymmetric unit, refcodes were filtered, using the Python API [33] if the corresponding CSD entry did not meet the following criteria: z' <= 1[28] (crystal.z_prime <= 1) and the SMILES representing the crystal chemical unit, which is the expansion of the asymmetric unit to include whole molecules (crystal.molecule.smiles), should not report more than one molecular species, i.e. should not contain a "." [37].

The applied force-field protocol employed the COMPASS force field [38–40], full geometry optimization of the crystal structure (molecule positions, internal molecule co-ordinates and the unit cell were all relaxed), without further geometry optimization of the gas phase molecule extracted from the relaxed crystal structure. The force-field protocol was applied via the Forcite module of Materials Studio [35], using Ewald summation for periodic electrostatics with force-field assigned charges and the quality setting set to "medium". These choices were informed via analysis of a preliminary version of a large dataset of curated sublimation data linked to crystal structures, for which the revised dataset and in-depth analysis will be reported in a forthcoming publication.

Preparation of molecular structures for descriptor calculations

Standardization employed the rdkit [41] and standardizer [42, 43] Python modules and entailed the following steps: (1) load the SMILES as an rdkit molecule object

(Chem.MolFromSmiles(…,sanitize=True)); (2) break bonds to Group I or II metals; (3) neutralize, via adding or removing protons; (4) rule based conversion of tautomeric and resonance forms;[44, 45] (5) neutralize again, following application of aforementioned rules. (Transformation to the neutral species is arguably most appropriate if modelling the intrinsic solubility, or corresponding enthalpy of solution values, since intrinsic solubility values refer to the solubility of the unionized solute [1, 4].) Compounds comprising multiple molecular species, molecules containing any inorganic components and SMILES which could not be parsed were filtered. The standardized SMILES, for which the selected rdkit output options also removed stereochemical information, were then converted to SDF format [46], using the rdkit [41]. Finally, all SDF structures were updated to include explicit hydrogen atoms, as is required for correct calculation of "integral" descriptors (see "Calculation of 2D molecular descriptors") using the rdkit [41].

Calculation of 2D molecular descriptors

The calculations we performed were based on consulting their publications [4, 6] and private correspondence with Dr. Alex Avdeef and Dr. Kyrylo Klimenko. In some cases, we were unable to perform *exactly* the same calculations due to the lack of the same software / software versions. A summary of the descriptor subsets calculated is provided in **Table S1**.

The following combinations of subsets were considered, denoted by their labels from **Table S1**: (1) Absolv and Ind; (2) Absolv; (3) Rdk and Absolv; (4) Rdk; (5) IntegSub and SiRMSSub; (6) IntegSub, SiRMSSub, Absolv, Ind and Rdk. Descriptor combinations (1 – 5) correspond, in some cases approximately, to combinations investigated by either Avdeef [4] (combinations 1 – 4) or Klimenko et al.[6] (combination 5). (Avdeef [4] always considered subsets of

molecular descriptors in combination with melting point values.) The final combination (6) was based on combining all subsets of descriptors.

Table S1. Subsets of 2D molecular descriptors calculated for our work. N.B. (1) The footnotes explain differences between our calculations and those of Avdeef [4] and Klimenko et al. [6] (2) For calculations carried out using the Percepta [47] and HiT-QSAR software [48], full details of the steps which need to be performed in the relevant GUIs are provided under Section B: How to Reproduce Our Results.

Label	Description	Calculation protocol
Absolv	Detailed explanations are presented in Abraham [49]. The abbreviated names from Avdeef [4] are used.<br><br>A = solute H-bond total acidity, representing H-bond donor ability[4, 49]<br><br>B = solute H-bond total basicity, representing H-bond acceptor ability[4, 49]<br><br>S = solute dipolarity-polarizability, representing the potential for dipole-induced dipole interactions[4, 49]<br><br>E = solute excess molar refraction, representing the potential for dispersion interactions[4, 49]<br><br>V = McGowan volume, representing the volume of the cavity formed by the solute in the solvent [50]<br><br>A × B = product representing the potential for solute - solute hydrogen bonding, e.g. in the solid state [51]	All descriptors, except A × B which was obtained via a script, were calculated using the Absolv component of the Percepta software program [47].
Ind	These are indicator variables: IA, IB, IAB, IN – equal to one (or zero) if the calculation indicates the molecule is (not) an acid, base, ampholyte or neutral respectively.	The presence of “acidic” and “basic” groups was detected using the rdkit [41], based on minor modifications of SMARTS patterns reported in the online documentation [52]. The presence of only “acidic” or “basic” groups set IA = 1 or IB = 1 respectively. The presence of both or none set IAB = 1 or IN = 1 respectively.[a]
Rdk	All 196 molecular descriptors from the rdkit [41] as documented online [53], were calculated. These include estimates of logP, substructure counts, topological indices and descriptors representing atomic charges.	The rdkit [41] was used for all calculations.[b]
IntegSub	The following “integral” descriptors were calculated.[c]<br><br>I.XLogP = logP estimate	These were a subset of the complete set of “integral” descriptors calculated using the HiT-QSAR software [48].

	I.EN = electronegativity of the molecule [54]<br><br>I.Rf = molecular refraction [54]<br><br>I.AW = molecular mass	
SiRMSSub	This refers to 2D Simplex Representation of Molecular Structure (SiRMS) descriptors, which are occurrence counts of all unique molecular substructures found in the dataset which conform to a specified set of topological graphs and atomic labels [55, 56]. Here, the atomic labels were defined according to the following properties: lipophilicity (XlogP contribution) [6], atomic refraction, atomic charge, Sybyl atom type, hydrogen bond donor/acceptor status, atomic contribution to van der Waals attraction (or, separately, repulsion) according to the UFF [57] forcefield.[c]	These were calculated using the HiT-QSAR software [48].

a. Conversely, Avdeef calculated these indicator variables using Algorithm Builder software, based on the estimated degree of ionization at pH 7.4 using computed pK_a values (private correspondence with Dr. Alex Avdeef).

b. Avdeef employed a set of 193 descriptors described as the "RDK" set [4], obtained via merging the descriptors calculated using an earlier version of the rdkit and logP and logD estimates calculated using pDISOL-X software (private correspondence with Dr. Alex Avdeef), for which we did not have access.

c. These were understood to correspond to the descriptors employed by Klimenko et al., based upon consulting their publication [6] and private correspondence with Dr. Kyrylo Klimenko.

Calculation of crystal structure based 3D molecular descriptors

In order to generate the SDF files used as input for the crystal structure based 3D descriptors, an initial SDF structure was obtained via parsing the CSD (version 5.38) crystal structure, for the corresponding refcode, using the CSD Python API (version 1.3.0). Parsing of this crystal structure included the addition of missing hydrogens, bonding information and removal of disordered atoms, as well as retrieval of 3D co-ordinates. Hence, these structures represented

the specific tautomer and conformer found in the crystal structure. The post-processing of those CSD derived SDF structures, including basic sanitization of resonance forms but not the extensive standardization described under “Preparation of molecular structures for descriptor calculations”, was carried out using the rdkit (version 2017.03.1). The rdkit (version 2017.03.1) was also employed, as part of a workflow including the same standardization routine described under “Preparation of molecular structures for descriptor calculations”, except for the retention of stereochemical information in this case, to obtain the structures used as input for the rdkit (version 2017.03.1) conformer generator. Finally, CPSA descriptors were calculated using Mordred (version 1.0.0) [58].

N.B. For one entry in the Avdeef_ExDPs_CS_True dataset (Carvedilol), the originally curated SMILES did not contain any stereochemical information and the conformer generator created a 3D structure for the opposite enantiomer to that retrieved from the crystal structure (CSD refcode GIVJUQ). However, CPSA descriptors are identical for enantiomers, as long as the internal distances are unchanged.

Melting point descriptor

We only used the experimental melting point values provided for the Avdeef [4] derived datasets as a descriptor. In a few cases, the same melting point was documented for different polymorphs, in the absence of polymorph specific data. Where a given occurrence of an instance identifier (as defined in Table 1 in the main text) was matched to multiple melting point values, the arithmetic mean value was assigned as the descriptor. For the Klimenko et al. [6] derived datasets, the experimental melting points values used as a descriptor were typically the value (or lower limit of the available values) associated with the corresponding CAS number in the Handbook of Aqueous Solubility [7]. Hence, these values may not correspond

to the (often unknown) polymorph for which solubility data were modelled. In one case, the Handbook of Aqueous Solubility [7] provided a melting point value for the specific polymorph for which solubility data were modelled: CAS number 72-14-0, form II. In that case, the polymorph specific melting point was used.

Feature selection

Feature selection was applied based on the model training set, either the cross-validation training set (see "Cross-validation protocols") or the entire dataset (see "Descriptor importance analysis"), to avoid model selection bias [59, 60]. Here, the objective was to reduce high dimensional descriptor vectors, for which the descriptors were expected to be significantly correlated, to a subset of 10 descriptors (an arbitrary small number). The motivation was twofold: (1) to avoid severe overfitting, in the case of Multiple Linear Regression [61, 62]; (2) to facilitate model interpretation. Feature selection was carried out on the basis of the following principles: the selected descriptors should be weakly correlated with each other, yet highly correlated with the endpoint to be predicted [63–65]. N.B. This is a heuristic, as descriptors which are individually poorly correlated with the endpoint might still be predictive in combination with others in a non-linear model (e.g. Random Forest) and [66] highly correlated descriptors might still add information in combination.

Specifically, we applied the following approach. (1) All descriptors were ranked according to their "correlation" (see below) with the endpoint. (2) The top 200 descriptors – an arbitrary number – were selected. (3) The pairwise distances (see below) between these descriptors were calculated. (4) These pairwise distances were used to cluster the descriptors, according to a hierarchical, agglomerative clustering approach employing "complete" linkage, as implemented in SciPy [67]. (5) The dendrogram [68] was then cut at the point at which only

10 clusters were formed or, in case this was not possible due to distances of zero being calculated between sufficiently similar descriptors, the largest number of clusters was selected which did not exceed 10. (6) For each cluster, the descriptor which was maximally "correlated" with the endpoint was selected.

N.B. Steps (1) and (2) were implemented to enable a clustering based approach, to reduce redundancy, to be applied in a computationally efficient manner to the very high dimensional descriptor vectors [63] considered here (see Table 1 in the main text).

In this approach, all references to "correlation" refer to the association between two variables measured in terms of their estimated Mutual Information [69, 70], as computed using SciKit-Learn [71, 72], and the distance metric used for clustering was calculated as per equation (ii), with all terms estimated via discretizing the variables based on 400 equally spaced bins using a combination of NumPy [73], Pandas [74], SciPy [75] and SciKit-Learn [71, 72]. N.B. The distance metric in equation (ii) is equivalent to the metric attributed to Cover and Thomas [76, 77].

$d(X, Y) = H(X) + H(Y) - 2I(X, Y)$ (ii)

In equation (ii), $d(X, Y)$ refers to the distance metric between two variables X and Y, i.e. two descriptors in the current context, $I(X, Y)$ refers to their Mutual Information and $H(X)$ refers to the Shannon entropy [76, 78].

Descriptor scaling

This was necessary for Multiple Linear Regression, in order to derive sensible coefficients. N.B. The derivation of sensible coefficients for Multiple Linear Regression is also contingent upon the descriptors being weakly correlated [62].

Machine Learning

Models were built using Multiple Linear Regression (MLR)[61] and the non-linear Random Forest regression (RFR)[79, 80] algorithms, as implemented in the randomForest package [81, 82] and lm(…) function of the stats package [83] in the R Statistical Computing Language. No hyperparameters were optimized, i.e. the randomForest defaults were employed for RFR. The use of the randomForest package default hyperparameters [81, 82] entailed growing 500 trees using the CART algorithm, with tree depth restricted only by setting the minimum number of terminal node instances to five – i.e. not by pruning, whilst the number of descriptors considered at each node was set to one third of the number of descriptors [79, 80]. However, each of the independently, randomly sampled subsets of the training data selected for growing the trees was selected without replacement, rather than the default [81, 82] bootstrap sampling [79, 80] (randomForest(…,replace=FALSE)).

Validation statistics

In equations (iii-iv), the summations and averages are computed over the N validation set molecules, whilst y_i, p_i, $\bar{y}$ and $\bar{p}$ denote the experimental, predicted, arithmetic mean experimental and arithmetic mean predicted endpoint values respectively.

$$R^2 = 1 - \frac{\sum_i^N (y_i - p_i)^2}{\sum_i^N (y_i - \bar{y})^2} \text{ (iii)}$$

$$RMSE = \sqrt{\frac{\sum_i^N (y_i - p_i)^2}{N}} \text{ (iv)}$$

Cross-validation protocols

The “vanilla” protocol was applied using the createFolds(….) function of the caret package [84, 85] for the R Statistical Programming Language.

Statistical significance of differences in cross-validated results

Pairwise differences in arithmetic mean validation statistics, as obtained from cross-validation, were evaluated for statistical significance for the key scenarios of interest. These key scenarios were pairwise comparisons of all corresponding modelling protocols, or cross-validation protocols, differing only with respect to the following: (1) whether the lattice energy descriptor was included; (2) whether the melting point descriptor was included; (3) whether the crystal structure based 3D descriptors or the conformer generator based 3D descriptors were used; (4) whether feature selection was applied; (5) whether the CV=v or CV=rt cross-validation protocol was applied.

For scenarios (1 – 4), two-tail p-values were calculated based on the paired cross-validation RMSE values, using an approximation, based on subsampling the permutations [86], of the paired permutation test proposed elsewhere [87, 88]. However, for scenario (5), the use of different cross-validation protocols meant two-tail p-values were generated using an approximate version of an unpaired permutation test [86]. (As RMSE estimates are not directly comparable for different validation sets, the R^2 values were compared.) Since a large number of pairwise comparisons were made for each scenario, all p-values obtained for a given scenario were treated as a family and were adjusted to control the false-discovery rate [89] using the approach of Benjamini and Yekutieli [90]. Unless noted otherwise, all references to statistically significant differences were based on the adjusted p-values and p-values less than 0.05 were considered statistically significant. Finally, it was assumed that a statistically significant difference meant that the observed sign of the difference in means, indicating one approach gave better performance on average than another, could be considered statistically significant.

The paired permutation test is designed to evaluate whether the arithmetic mean difference in cross-validation results obtained using approaches A and B, for identical training/validation partitions of the same dataset and using the same set of random number generator seeds to account for the inherent random nature of Random Forest results [79, 91], is statistically significantly different to zero [87, 88]. Here, the null-hypothesis is that the mean difference across the population, of which the obtained results are a sample, of all possible corresponding validation results (i.e. the difference in test set results obtained when A and B are applied to the same training and test set and, for Random Forest, use the same seed), for A and B applied to training and validation data for which the available dataset is representative, is zero. The alternative hypothesis is that this mean is non-zero. The exact permutation test, for paired cross-validation results, is applied as follows.

1. For each pair of corresponding validation set results, calculate the difference (D_i) in the value for the validation statistic of interest (RMSE) obtained with approach A (S_{iA}) and B (S_{iB}), as per equation (v).
2. Sum these differences, and compute the absolute magnitude of this sum (T_0), as per equation (vi). In equation (vi), I denotes the total number of paired validation set results.
3. Generate a random permutation of the labels A and B, by swapping the labels for a randomly selected set of pairs, and repeat steps (1) and (2), giving T_p as per equation (vii), where $p_i = \pm 1$. In equation (vii), whether a plus or minus sign is used before each original difference term (D_i) depends upon whether the labels were swapped for the ith pair of validation set results.
4. Repeat steps (3-4) until all possible 2^I permutations, including the original set of difference terms, have been generated.
5. For all 2^I permutations, determine if $T_P \geq T_0$.

6. Calculate the p-value (P) as per equation (viii), where n is the number of times $T_P \geq T_0$, including for the original permutation where $T_P = T_0$, and N is the total number of permutations ($N = 2^I$).

$$D_i = S_{iA} - S_{iB} \text{ (v)}$$

$$T_0 = \left|\sum_i^I D_i\right| \text{ (vi)}$$

$$T_p = \left|\sum_i^I p_i D_i\right| \text{ (vii)}$$

$$P = \frac{n}{N} \text{ (viii)}$$

Since we generated up to 125 cross-validation results for a given modelling protocol, cross-validation protocol and dataset (five repetitions of five-fold cross-validation, with all results for a given training/validation set generated five times with different random seeds for Random Forest), considering all 2^{125} permutations [87, 88] was not computationally tractable and 10,000 permutations were randomly subsampled, as advocated elsewhere [86]. Otherwise, the p-value was calculated as per the exact permutation test, for paired cross-validation results, as described above.

The unpaired permutation test [86] is designed to evaluate whether the mean difference in cross-validation results, generated on different training/validation partitions of the same dataset using the same modelling approach – including the same set of random number generator seeds to account for the inherent random nature of Random Forest [79, 91], is statistically significantly different to zero. Since different training/validation partitions are used, as a result of different cross-validation protocols in our case, the validation set results cannot be paired and a greater number of permutations of the labels A and B originally applied to the results is possible, as compared to the paired permutation test. Here, these labels denote whether the results were obtained with the cross-validation protocol CV=v or CV=rt. The total number of possible permutations (N) is given by equation (ix) [86], where I is the total number of cross-validation results in each of the two sets of results being compared. As explained above, I is up

to 125 for our work. The magnitude of the difference in the sum of results labelled A and B is computed for each permutation, as per equation (x), with T_0 denoting the value of T_p calculated for the original set of results, i.e. the original permutation. Then, as per the paired permutation test, the p-value is calculated using equation (viii), where n is the number of times $T_P \geq T_0$. For the unpaired permutation test, a random sample of 10,000 of the total possible permutations was also selected to estimate the p-value.

$$N = \frac{(2I)!}{I!I!} \text{ (ix)}$$

$$T_p = \left|\sum_i^I S_{iA} - \sum_i^I S_{iB}\right| \text{ (x)}$$

Finally, the exact permutation test has been proposed as a more suitable alternative to the commonly employed t-test, which is known to suffer from elevated Type 1 error, i.e. to generate too small p-values, for evaluating differences in mean, paired cross-validation results [88, 92, 93]. However, in practice, the approximate version of the permutation test employed here may yield p-values which are smaller than those obtained from the t-test. For example, consider the 176 pairwise comparisons between mean RMSE values obtained using corresponding models with and without the lattice energy descriptor. For 144 of these comparisons, it was possible to calculate a paired t-test two-tail p-value using the statsmodels Python module [94]. (Due to the calculation failures here, it was not possible to calculate adjusted p-values from the raw t-test p-values.) Out of those 144 comparisons, the paired t-test p-value was larger than the approximate permutation test paired p-value in 81 cases. However, in only six of those cases was the approximate permutation p-value less than 0.05 when the t-test p-value was not. None of these comparisons involved the best performing models and, since statistical significance was ultimately assessed via comparing the adjusted approximate permutation p-values to 0.05, these findings do not directly affect our conclusions. Nonetheless, this does suggest that the findings regarding statistically significant differences in the current work should be considered approximate findings of statistical significance. N.B.

The t-test results were generated via running "summarize_cv_results_v16_ttest_mod.py" provided in Additional File 9.

Filtering of SUB-48 Dataset

The same filtering criteria were applied, using the CSD API, as per "Integration with crystal structures", albeit on another machine with the updated version 1.3.0 of the CSD Python API installed [33] and CSD version 5.37. The 27 retained entries are identified in Additional File 3.

Computational details

Starting from the curated and integrated solubility and enthalpy of solution datasets made available in Additional File 8, all calculations, save for generating the CIF files required for lattice energy calculations, were performed on a computer with the following setup. (The CIF files were generated on another machine with the updated version 1.3.0 of the CSD Python API installed [33], working with CSD version 5.37.) Operating System: Windows 7 Enterprise Service Pack 1 (64-bit). Processor: Intel(R) Core™ i5-6300U CPU @ 2.40GHz 2.40 GHz. Installed memory (RAM): 8GB (7.41 GB usable). All R Statistical Programming scripts were run using R version 3.3.2 (64-bit). The versions of relevant packages are provided in their citations or were otherwise the default installations. All Python scripts were run using Anaconda (Python 2.7.12, 32-bit, installed using Anaconda2-4.2.0-Windows-x86.exe). The versions of relevant modules are provided in their citations, and/or are explicitly noted in the applicable subsection of "Section A: Extended Methods and Data", or were otherwise the default installations, except for the following modules: numba (version 0.33.0), mkl (version

1.1.2), numexpr (version 2.6.2), conda (version 4.3.23 – updated to version 4.3.30 prior to generating 3D SDF structures), llvmlite (0.18.0), requests (version 2.14.2), libpng (version 1.6.28), tqdm (version 4.23.0).

Section B: How to Reproduce Our Results

This section describes how to reproduce our results, starting from the curated datasets we have made available, using the scripts we have made available.

Evaluating the lattice energy protocol on the SUB-48 dataset

See "Section A: Extended Methods and Data: Computational details" and "Section A: Extended Methods and Data: Calculation of lattice energies" for details of the software and hardware versions used.

1. Download the following set of scripts: "lattice_energy_scripts.zip" (Additional File 7).
2. Download "curated_datasets.zip" (Additional File 8).
3. Extract the SUB-48 dataset file: "SUB-48_ds_fromAuthors_prep.xlsx".
4. Extract the corresponding SUB-48 dataset file, containing the experimental estimates of lattice energy: "SUB-48_ExperimentalLatticeEnergyEstimates.csv".
5. Copy the CSD refcodes from "SUB-48_ds_fromAuthors_prep.xlsx" into a plain text file, with one refcode per line: "refcodes.txt". (The precise name does not matter.)
6. Run the following script, as follows: "python csd_refcodes_to_zvalue_labelled_cifs_post_filter.py –i refcodes.txt". This should generate the 48 CIF files required.
7. Edit "MaterialsStudio_LE_script_AqModelsPaper.pl" so that '$inputFilesFolder =' refers to the directory containing the CIF files and '$outputFilesFolder =' refers to the directory into which the calculated lattice energies will be written, in CSV format: "Results.std.csv". N.B The version of this file we generated is available from Additional File 3.

8. Filter the resulting file ("Results.std.csv") so that only the 27 crystal structures documented in "Results.std_Filtered.csv" (from Additional File 3, generated as described under "Filtering of SUB-48 Dataset" above) remain, in order to generate a new version of "Results.std_Filtered.csv".
9. Run the following script, as follows: "python updated_analysis_script.py -f Results.std_Filtered.csv –e SUB-48_ExperimentalLatticeEnergyEstimates.csv -o PerformanceStatistics.csv". This will generate a set of performance statistics for the force-field lattice energy calculation protocol. N.B. "rev_MS_CD_calc_pred_perf_metrics.py" must be kept in the same directory as this script.

Calculating lattice energies for the solubility and enthalpy of solution datasets

The lattice energies for these datasets are stored in the curated datasets, in the "CrystalStructures_Precursor" worksheet. The same workflow, using the same scripts and same software and hardware versions, to go from the CSD refcodes to calculated lattice energies for the SUB-48 dataset (described above) was used to go from the refcodes documented in this workbook to the calculated lattice energies. The lattice energies were first calculated for the provisional CSD refcode matches documented in the "CrystalStructures_Precursor" worksheet. Subsequently, the CSD refcodes were filtered to give the final matches documented in the "CrystalStructures" worksheet as documented under "Section A: Extended Methods and Data: Integration with crystal structures". In order to rank the refcode matches, for a given Name: CAS number: Polymorph description combination, based on the calculated lattice energies, the following script, part of the

“QSPR_and_curation_utilities.zip” (Additional File 4) set of utilities, needs to be downloaded and employed: “select_lowest_energy_refcode.py”.

Generating QSPR modelling results

See “Section A: Extended Methods and Data: Computational details”, “Section A: Extended Methods and Data: Preparation of molecular structures for descriptor calculations”, “Section A: Extended Methods and Data: Calculation of 2D molecular descriptors”, “Section A: Extended Methods and Data: Calculation of crystal structure based 3D molecular descriptors”, “Section A: Extended Methods and Data: Feature selection”, “Section A: Extended Methods and Data: Machine Learning” and “Section A: Extended Methods and Data: Cross-validation protocols” for details of the software and hardware versions used.

1. Download the following set of utilities: “QSPR_and_curation_utilities.zip” (Additional File 4), “feature_selection_tool.zip” (Additional File 6). It is important that the directory structure of these archives is not changed.
2. Download the following collection of scripts: “QSPR_scripts.zip” (Additional File 9). It is important that the directory structure of this archive is not changed.
3. Download “SMARTS_patterns.zip” (Additional File 10) and copy “mod_rdk_acid_base_SMARTS_file.txt” into the “calc_rdk_and_ind_descs” subdirectory of “QSPR_scripts”.
4. Download “curated_datasets.zip” (Additional File 8) and extract the curated temperature dependent solubility and enthalpy of solution datasets: “ADDoPT_Data_Template_2.9.16_Klimenko2016_rev.5.xlsx” and “ADDoPT_Data_Template_2.9.16_Avdeef2015_rev.5.xlsx”.

5. Place these curated datasets into a common directory: [YOUR CURATED DATASET DIRECTORY].
6. Replace all occurrences of r' C:\UoLeeds_work.PC\ADDoPT\data\template_data_sets\rev.3models_rev.5_Klimenko Avdeef', in the files contained within "QSPR_scripts", with [YOUR CURATED DATASET DIRECTORY].
7. Place the directory "v1.2" from "feature_selection_tool", along with all its contents, into the "feature_selection" subdirectory of "QSPR_scripts".
8. Extract the following subdirectory from "QSPR_and_curation_utilities" and place it under a directory of your choosing, called [YOUR QSPR UTILITIES DIRECTORY]: "Leeds_ADDoPT.WP3_tools-0.17".
9. Replace all occurrences of r'C:\UoLeeds_work.PC\ADDoPT\code\releases\Leeds_ADDoPT.WP3_tools-0.17', in the files contained within "QSPR_scripts", with "[YOUR QSPR UTILITIES DIRECTORY]\Leeds_ADDoPT.WP3_tools-0.17". N.B. The forward slash assumes you are running the code under Windows.
10. (i) Go to QSPR_scripts\prep_for_descs_calc; (ii) Run KlimenkoAvdeef_rev.5_prep_for_descs_calc.py
11. (i) Go to QSPR_scripts\calc_Absolv_desc; (ii) Separately, import both Avdeef_desc_calc_input_CD_False.sdf and Klimenko_desc_calc_input_CD_False.sdf from QSPR_scripts\prep_for_descs_calc into the Percepta software [47]; (iii) For both SDFs, import all fields; (iv) For both SDFs, calculate the Absolv descriptors [Calculate -> Properties -> select AlphaH2 (A), BetaH2 (B), Pi2 (S), R2 (E), McGowan Volume (V)]; (v) For both SDFs, export the results (including all fields) as SDFs, named as [input SDF suffix]_Absolv.sdf, e.g. Avdeef_desc_calc_input_CD_False_Absolv.sdf, into QSPR_scripts\calc_Absolv_desc;(vi) Run KlimenkoAvdeef_rev_Absolv_descs_csvs.py

12. (i) Go to QSPR_scripts\calc_HIT.QSAR_descs;(ii) Separately, import both Avdeef_desc_calc_input_CD_False.sdf and Klimenko_desc_calc_input_CD_False.sdf from QSPR_scripts\prep_for_descs_calc into the LSM.exe module of the HiT-QSAR software [48]; (iii) For each SDF separately, perform the following sequence of steps, with both the LSM.exe and mda1.exe GUI windows being closed between SDFs:(a) In the LSM.exe GUI, select Descriptors -> Integral;(b) In the LSM.exe GUI, select Simplex -> Calculate simplex & fragmentary descriptors;(c) In the LSM.exe GUI, under the “Fragmentary descriptors dialog”: “Fragments” panel, make sure the "Generate 2D & 3D parameters" box is ticked (not the “Generate 1D Parameters” box), with "Atoms count from 2 to 4", and click "Select connected";(d) In the LSM.exe GUI, under the “Fragmentary descriptors dialog”: “Differentiation” panel, select all boxes and keep the default ranges;(e) In the LSM.exe GUI, under the “Fragmentary descriptors dialog”: “Other” panel, make sure the "Only 2D descriptors" option is selected;(f) In the mda1.exe GUI, for each .dat file generated in turn by the LSM.exe GUI, perform the following sequence of steps:(I) Import the .dat file;(II) Save in the Dragon v1-4 format, with the following names assigned to the appropriate file generated from the corresponding .dat file:Integral_Dragon.txt, Simplex_att_Dragon.txt, Simplex_chg_Dragon.txt, Simplex_da_Dragon.txt, Simplex_elm_Dragon.txt, Simplex_en_Dragon.txt, Simplex_lip_Dragon.txt, Simplex_none_Dragon.txt, Simplex_rep_Dragon.txt, Simplex_rf_Dragon.txt, Simplex_type_Dragon.txt;(g)Run “mv_HITQSAR_out.py -i [ABSOLUTE NAME OF DIRECTORY INTO WHICH YOU HAVE DOWNLOADED QSPR_scripts]\QSPR_scripts\prep_for_descs_calc -o [ABSOLUTE NAME OF DIRECTORY INTO WHICH YOU HAVE DOWNLOADED QSPR_scripts]\QSPR_scripts\calc_HIT.QSAR_descs -s [name of original SDF] –C”.
13. (i) Go to QSPR_scripts; (ii) Run run_all.py

14. At this point, update the installation of the rdkit Python module. All of the preceding results were generated, where applicable, using rdkit version 2016.03.1 [41]. All further calculations employed rkdit version 2017.03.1 [95], which provided access to the ETKDG conformer generator algorithm [96]. For all subsequent calculations, the previously calculated descriptors are employed, with the only new steps dependent upon the updated rdkit version being the preparation of 3D structures for calculating the crystal structure based and conformer generator based 3D descriptors.
15. (i) Go to QSPR_scripts\extra_3D_sdfs_and_descs; (ii) run extra_3D_sdfs.py; (iii) run get_all_3D_descs.py
16. (i) Go to QSPR_scripts\extra_get_Klimenko_MP_descs; (ii) run get_Klimenko_MP_descs.py
17. (i) Go to QSPR_scripts\extra_qspr_input; (ii) run extra_qspr_input.py
18. (i) Go to QSPR_scripts; (ii) run run_extra_modelling.py
19. (i) Go to QSPR_scripts\extra_LE_models_input; (ii) run extra_LE_models_input.py
20. (i) Go to QSPR_scripts\extra_LE_models; (ii) run extra_run_LE_models.R.py
21. (i) Go to QSPR_scripts\extra_LE_models_no_outliers\extra_LE_models_input; (ii) run extra_LE_models_input_no_outliers.py
22. (i) Go to QSPR_scripts\extra_LE_models_no_outliers\extra_LE_models; (ii) run extra_run_LE_models.R_no_outliers.py

Running analysis of modelling results and datasets

1. (i) Go to QSPR_scripts\analysis; (ii) Run summarize_cv_results_v16.py; (iii) Run find_consistently_important_descriptors.py; (iv) Run "analyse_descriptor_importance.py –d [name of descriptor of interest] –m [name of

method of interest] –D [prefix of datasets of interest]”, e.g. “analyse_descriptor_importance.py –d D_Temperature_Pneg1_C1_O0 –m MLR –D Klimenko” N.B. The temperature descriptor name, for the feature selection reduced datasets, was “D_Temperature_Value_Pneg1_C1_O0”; (v) Run “assess_vant_Hoff_for_Klimenko_datasets.py”

2. (i) Go to QSPR_scripts\analysis\mps_analysis; (ii) Run find_LE_Tm_correlations.py; (iii) Run find_LE_Tm_correlations_WITH_Tm_FROM_DS_cf_CSD_Tm.py; (iv) Run find_LE_Tm_correlations_WITH_Tm_FROM_CSD.py

Section C: Extended Results and Discussion

Comparison to the literature

The results we obtained which are most directly comparable to the results reported in the works of Avdeef [4] and Klimenko et al.[6] are presented in Table S2. It can be seen that the estimated performance of the models is generally, but not always, similar.

Nonetheless, it should be noted that these results cannot be expected to be identical, due to important differences in our modelling and validation approaches. Firstly, the exact datasets modelled are different. Avdeef [4] modelled 571 data points, with each data point treated as a separate instance. Conversely, our nearest equivalent QSPR ready dataset (Table 1 in the main text), Avdeef_ExDPs_CS_False, corresponded to 364 instances, each instance corresponding to all unique [name]_[CAS number (blank)]_[polymorph description (typically blank)] values derived after merging identifiers for duplicate molecules, with multiple enthalpy of solution values averaged prior to modeling. Whilst we filtered low quality data points as per Avdeef [4], we also filtered molecular structures with multiple components and data for which other problems were identified. Similarly, Klimenko et al.[6] modelled 1484 aqueous solubility data points, each matched to a unique combination of CAS number and temperature value. Conversely, our closest equivalent QSPR ready dataset (Klimenko_CS_False) comprised 882 instances, each instance corresponding to a unique combination of [name (typically blank)]_[CAS number]_[polymorph description (typically blank)], after filtering structures for which problems were identified. Critically, we also filtered dataset entries where there was no evidence that the material being dissolved was in the solid state (see "Solubility data curation"). Secondly, we were not able to calculate all molecular descriptors in exactly the same fashion, as explained in **Table S1**. Thirdly, for the datasets derived from the work of Avdeef [4], we

only used experimental melting point data as a melting point descriptor. Fourthly, we also used a less complex functional form for the temperature descriptor than Klimenko et al.[6] Finally, there were some differences in the Machine Learning and validation protocols. The differences in the validation protocols are indicated in Table S2. In addition, we used the randomForest default number of trees (500) [81, 82], whereas Klimenko et al.[6] used 200 trees for Random Forest Regression (RFR) modelling, and we built the RFR models using sampling of the training set without replacement for each tree.

Regarding comparison between the best results reported in the literature (Table S2) and the corresponding best performing results obtained here (Table 2 in the main text), it can be seen that our best result on the Avdeef_ExDPs_CS_False dataset is fairly similar and our best result on the Avdeef_ExDPs_Cal_False dataset is somewhat better than the best results reported on the corresponding datasets by Avdeef [4]. Consideration of Table S2 and Table S3 illustrates that our best result on the Klimenko_CS_False dataset (CV=v) is substantially higher in terms of the mean R^2 (0.97 vs. 0.78) than the closest comparable result reported by Klimenko et al.[6], although the mean RMSE values are quite similar. Nonetheless, it should be reiterated that we cannot claim to have performed an entirely like for like comparison to either literature study, as explained in the preceding paragraph.

Table S2. Summary of comparable results obtained herein and in the cited studies. As noted in the preceding text, these are not perfectly like for like comparisons. Corresponding results are reported on consecutive rows: (1) literature result; (2) our corresponding result.

Source	Dataset[a]	Descriptors[b]	Validation	Algorithm	R^2 (average)	RMSE (average)[c]
Avdeef (2015) [4]	Avdeef_ExDPs_Cal_CS_False (Avdeef 2015)	MP, Absolv & RDK (rdkit + logD/logP)	validated on random selection of 30% of the data	RFR	0.61	11.70
Our work	Avdeef_ExDPs_Cal_CS_False	MP, Absolv & rdkit	5 x 5-fold CV (repeated with 5 seeds)	RFR	0.49	10.15
Avdeef [4]	Avdeef_ExDPs_CS_False (Avdeef 2015)	MP, Absolv & Ind	100 x leave-20%-out	MLR	0.27	Not available

Our work	Avdeef_ExDPs_CS_False	MP, Absolv & Ind	5 x 5-fold CV	MLR	0.23	14.90
Avdeef [4]	Avdeef_ExDPs_CS_False (Avdeef 2015)	MP & Absolv	validated on random selection of 30% of the data	RFR	0.25	15.10
Our work	Avdeef_ExDPs_CS_False	MP & Absolv	5 x 5-fold CV (repeated with 5 seeds)	RFR	0.25	14.70
Avdeef [4]	Avdeef_ExDPs_CS_False (Avdeef 2015)	MP, Absolv & RDK (rdkit + logD/logP)	validated on random selection of 30% of the data	RFR	0.35	13.90
Our work	Avdeef_ExDPs_CS_False	MP, Absolv & rdkit	5 x 5-fold CV (repeated with 5 seeds)	RFR	0.33	13.88
Avdeef [4]	Avdeef_ExDPs_CS_False (Avdeef 2015)	MP & RDK (rdkit + logD/logP)	validated on random selection of 30% of the data	RFR	0.32	14.40
Our work	Avdeef_ExDPs_CS_False	MP & rdkit	5 x 5-fold CV (repeated with 5 seeds)	RFR	0.34	13.84
Klimenko et al.[6]	Klimenko_CS_False (Klimenko et al. 2016)	SiRMsSub, IntegSub & Temperature Descriptor (1)	5-fold CV	RFR	0.78	0.38
Our work	Klimenko_CS_False	SiRMsSub, IntegSub & Temperature Descriptor (2)	5 x 5-fold CV (repeated with 5 seeds); CV=v	RFR	0.97	0.45

a. For ease of comparison, the datasets modelled in the work of Avdeef[4] and Klimenko et al.[6] have been labelled similarly to the closest corresponding QSPR ready dataset (see Table 2 in the main text) modelled in our work. However, as explained above, these datasets are not exactly equivalent.
b. As explained under "Calculation of 2D molecular descriptors" (above) and "Temperature descriptor" (in the main text), there were some differences in the molecular descriptor calculations for our work and a different functional form for the temperature descriptor was employed for the Klimenko et al.[6] derived datasets.
c. RMSE is in kJ/mol for the Avdeef [4] derived datasets and log units for the Klimenko et al. [6] derived datasets.

Table S3. Top ranked results according to various scenarios for the temperature dependent solubility datasets. All results were obtained without feature selection. All results are rounded to 2dp. The definitions of 2D molecular descriptors subsets are provided in **Table S1**. All references to R^2 and RMSE denote arithmetic mean values obtained from cross-validation and all model rankings were generated based on the mean RMSE values.

Dataset	CV protocol	Rank	Molecular Descriptors	3D Descriptors from Crystal Structure?	Melting Point Descriptor Included	Lattice Energy Descriptor Included	Method	R^2	RMSE (log units)
Klimenko_CS_False	rt	1st	IntegSub, SiRMSSub, Absolv, Ind, Rdk	FALSE	TRUE	FALSE	RFR	0.92	0.70
Klimenko_CS_False	rt	2nd	Rdk, Absolv	FALSE	TRUE	FALSE	RFR	0.92	0.70
Klimenko_CS_False	v	1st	Rdk, Absolv	FALSE	TRUE	FALSE	RFR	0.97	0.41
Klimenko_CS_False	v	2nd	Rdk	FALSE	TRUE	FALSE	RFR	0.97	0.41
Klimenko_CS_True	rt	1st	3D, IntegSub, SiRMSSub, Absolv, Ind, Rdk	FALSE	TRUE	FALSE	RFR	0.85	0.83
Klimenko_CS_True	rt	2nd	Rdk, Absolv	FALSE	TRUE	FALSE	RFR	0.85	0.83
Klimenko_CS_True	v	1st	Rdk, Absolv	FALSE	TRUE	FALSE	RFR	0.98	0.35
Klimenko_CS_True	v	2nd	Rdk	FALSE	TRUE	FALSE	RFR	0.98	0.35

Additional Plots of Cross-Validated Results for the Best Models

The following figures (Figure S1 - Figure S6) present the cross-validated R^2 values obtained for the same models presented in Figures 5 – 10 in the main text.

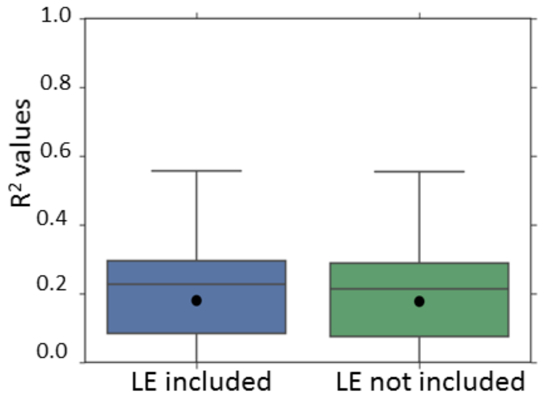

Figure S1. Cross-validated performance (R^2) of the top performing model where the lattice energy (LE) descriptor was incorporated (LHS), compared to the corresponding model which didn't include the lattice energy descriptor (RHS): dataset = Avdeef_ExDPs_CS_True. The distributions of cross-validated results are presented as a boxplot, with whiskers extending 1.5 times the interquartile range beyond the upper and lower quartiles, with the arithmetic mean superimposed as a black circle.

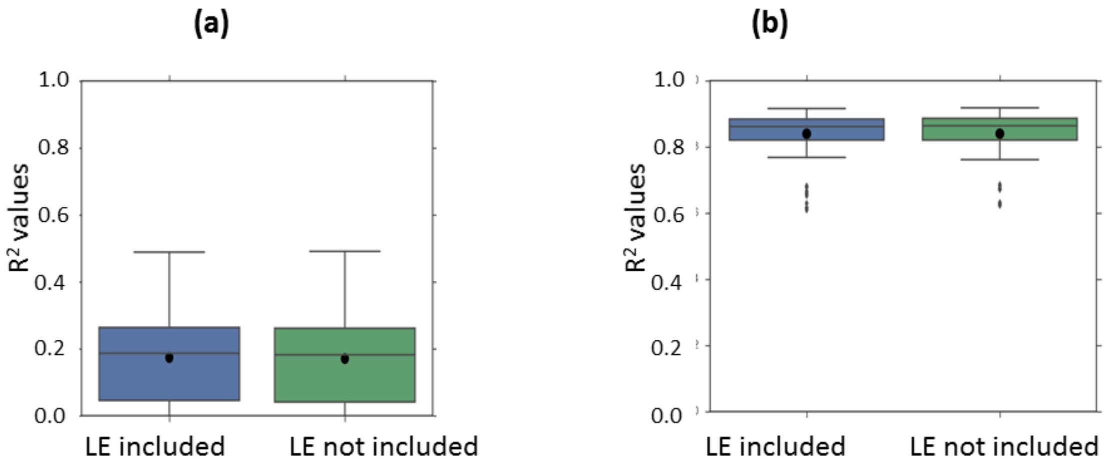

Figure S2. Cross-validated performance (R^2) of the top performing model (excluding models incorporating the melting point or crystal structure based 3D descriptors) where the lattice energy (LE) descriptor was incorporated (LHS), compared to the corresponding model which didn't include the lattice energy descriptor (RHS): (a) dataset = Avdeef_ExDPs_CS_True; (b) dataset = Klimenko_CS_True, CV=rt. The distributions of cross-validated results are presented as a boxplot, with whiskers extending 1.5 times the interquartile range beyond the upper and lower quartiles, with the arithmetic mean superimposed as a black circle.

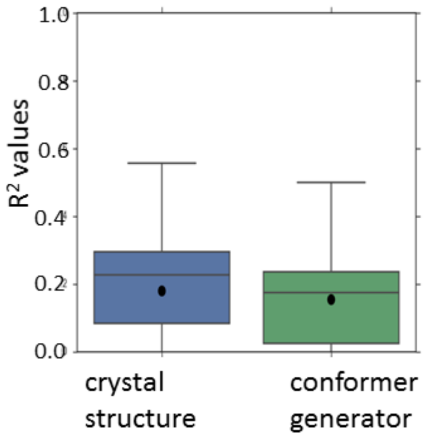

Figure S3. Cross-validated performance (R^2) of the top performing model where the crystal structure based 3D descriptors were incorporated (LHS), compared to the corresponding model using the conformer generator based 3D descriptors (RHS): dataset = Avdeef_ExDPs_CS_True.

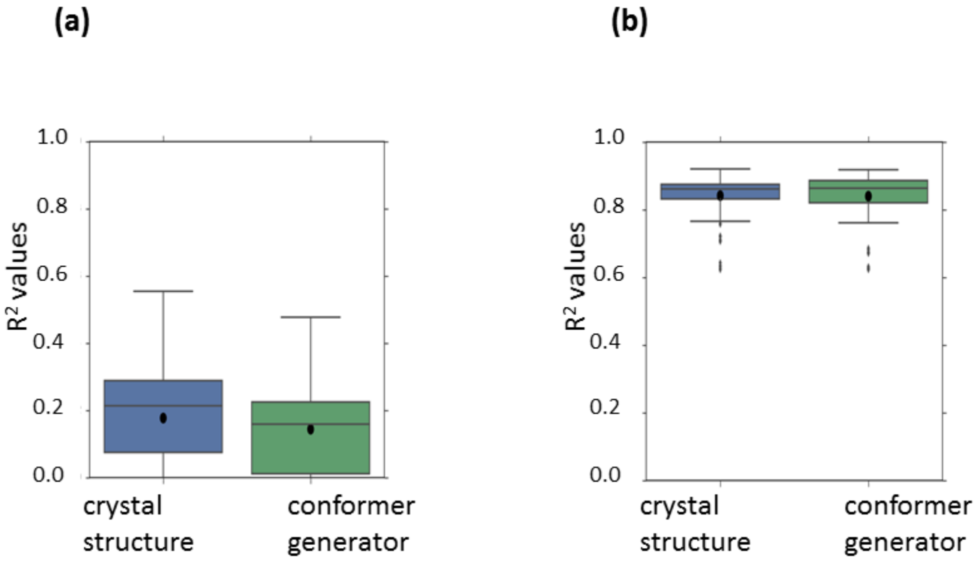

Figure S4. Cross-validated performance (R^2) of the top performing models (excluding models incorporating the melting point or lattice energy descriptor) where the crystal structure based 3D descriptors were incorporated (LHS), compared to the corresponding model using the conformer generator based 3D descriptors (RHS): (a) dataset = Avdeef_ExDPs_CS_True; (b) dataset = Klimenko_CS_True, CV=rt.

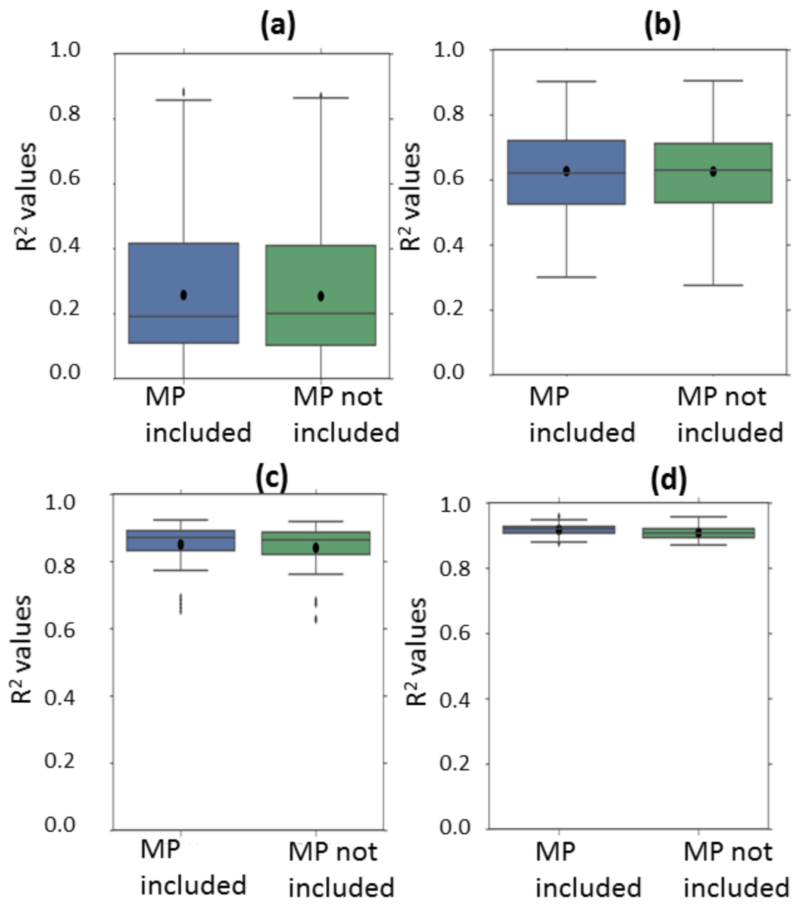

Figure S5. Cross-validated performance (R^2) of the top performing models for all scenarios where they incorporated the melting point (MP) descriptor (LHS), compared to the corresponding model which didn't include the MP descriptor (RHS): (a) dataset = Avdeef_ExDPs_Cal_CS_True; (b) dataset = Avdeef_ExDPs_Cal_CS_False; (c) dataset = Klimenko_CS_True, CV=rt; (d) Klimenko_CS_False, CV=rt.

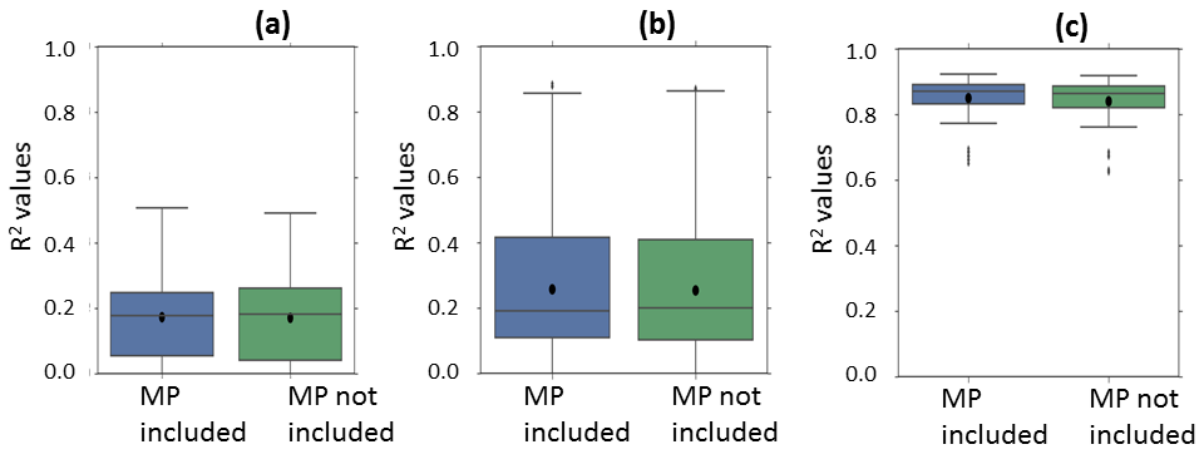

Figure S6. Cross-validated performance (R^2) of the top performing models for all crystal structure integrated datasets, excluding models involving the lattice energy or crystal structure based 3D descriptors, where they incorporated the melting point (MP) descriptor (LHS), compared to the corresponding model which didn't include the MP descriptor (RHS): (a) dataset = Avdeef_ExDPs_CS_True; (b) dataset = Avdeef_ExDPs_Cal_CS_True; (c) dataset = Klimenko_CS_True, CV=rt.

Effect of incorporating the lattice energy descriptor: pairwise comparison of models

Pairwise analysis of the corresponding results on the Avdeef_ExDPs_CS_True dataset suggested possible enhanced performance due to the inclusion of the lattice energy descriptor for only 22 out of the 40 relevant scenarios. (Since the lattice energy descriptor was never retained following feature selection, for any dataset, only those results generated without feature selection are considered.) Moreover, only eight of those scenarios appeared to correspond to statistically significant differences in mean RMSE. These eight scenarios corresponded to models generated using RFR and a variety of 2D or 3D molecular descriptors – not including 3D descriptors based on the crystal structure – with or without the melting point descriptor. Of these eight scenarios, the largest decrease in mean RMSE was 0.45 kJ/mol, with a corresponding increase in mean R^2 of 0.06, when only Absolv molecular descriptors were used without the melting point descriptor.

For the Avdeef_ExDPs_Cal_CS_True dataset, an apparent improvement enhancement upon including the lattice energy descriptor only occurred for nine out of 40 scenarios, only four of which appeared to correspond to statistically significant differences in mean RMSE. These four scenarios were based on the Absolv, or Absolv and Ind, molecular descriptors, with or without the melting point descriptor, no 3D descriptors and RFR. Of these four scenarios, the inclusion of the lattice energy descriptor lead to a maximum mean increase in R^2 of 0.05 and a maximum mean reduction in RMSE of 0.42 kJ/mol.

Pairwise analysis of the results obtained for the Klimenko_CS_True dataset also revealed few scenarios where the inclusion of the lattice energy descriptor appeared to genuinely enhance predictive performance. Restricting consideration to those results obtained when the modified

cross-validation protocol (CV=rt) was used, 25 out of 40 scenarios showed an apparent improvement upon incorporating the lattice energy descriptor. Of these 25, 10 appeared to correspond to statistically significant differences in mean RMSE. These 10 scenarios all corresponded to the use of various molecular descriptors, sometimes including 3D descriptors computed from the crystal structure or the conformer generator molecular structure, with models built using RFR or MLR with or without the melting point descriptor. Of those 10 pairwise comparisons, the largest decrease in mean RMSE was 0.34 log units, with a corresponding increase in mean R^2 of 0.40. That result was obtained using MLR with 3D molecular descriptors (not computed from the crystal structure) only.

Restricting consideration to those scenarios not involving the melting point descriptor or 3D descriptors computed from crystal structure allows an assessment of whether the proportion of pairwise scenarios where an apparent improvement enhancement occurred upon adding the lattice energy descriptor was skewed by the inclusion of these additional descriptors of the solid state contribution to the modelled endpoints. The proportion of apparently statistically significant enhancements in mean RMSE was not very different when consideration was restricted to these pairwise comparisons without additional solid state contribution descriptors. For the Avdeef_ExDPs_CS_True dataset, nine out of 16 such comparisons showed an apparent improvement enhancement and four of these appeared to correspond to statistically significant differences in mean RMSE. For the Avdeef_ExDPs_Cal_CS_True dataset and the Klimenko_CS_True dataset (CV=rt), apparent (statistically significant) improvement enhancements were observed in three (two) and 12 (five) out of 16 pairwise comparisons respectively.

Effect of incorporating the 3D descriptors based on crystal structure: pairwise comparison of models

Pairwise analysis of the Avdeef_ExDPs_CS_True dataset shows that the models using crystal structure based 3D descriptors outperformed the corresponding models using conformer generated 3D descriptors in 14 out of 16 cases. However, the differences in mean RMSE only appeared to be statistically significant in six of those cases. In all six cases, only the 3D descriptors were employed – with or without the lattice energy or melting point descriptor – with either RFR or MLR used to build the model. However, in both cases, the MLR models corresponded to extreme overfitting (mean $R^2 < 0$). Of the remaining four cases, the largest reduction in mean RMSE was 0.30 kJ/mol, with a corresponding increase in mean R^2 of 0.03. This was obtained without the lattice energy or melting point descriptor. Contrastingly, for the Avdeef_ExDPs_Cal_CS_True dataset, only three out of 16 such pairwise comparisons showed increased performance due to the use of 3D descriptors based on the crystal structure. None of those three cases appeared to reflect statistically significant differences. Similarly, for the Klimenko_CS_True (CV=rt) results, 10 out of 16 pairwise comparisons indicated the

crystal structure based 3D descriptors improved performance, but none of the differences in mean RMSE appeared statistically significant.

Removing the potential confounding influence of the lattice energy and melting point descriptors resulted in all four remaining pairwise comparisons for the Avdeef_ExDPs_CS_True dataset appearing to correspond to enhanced performance upon using crystal structure based 3D descriptors. However, only one such case – the RFR model built using only 3D descriptors - appeared to reflect statistically significant differences in mean RMSE. However, for the Avdeef_ExDPs_Cal_CS_True dataset, only one of the four remaining pairwise comparisons appeared to correspond to enhanced performance upon using crystal structure based 3D descriptors and the reduction in mean RMSE did not appear statistically significant. Indeed, for the model built using only 3D descriptors and RFR, the mean RMSE appeared statistically significantly worse when crystal structure based 3D descriptors were used. However, this could just be an artefact of the small dataset size. For the corresponding Klimenko_CS_True (CV=rt) results, three out of four scenarios also indicated improved performance upon using crystal structure based 3D descriptors, yet none of these appear to correspond to statistically significant differences.

Effect of incorporating melting point: pairwise comparison of models

Pairwise analysis of the corresponding results on the Avdeef_ExDPs_CS_False and Avdeef_ExDPs_Cal_CS_False datasets suggested possible enhanced performance due to the inclusion of the melting point descriptor for five and four out of the 12 relevant scenarios respectively. (Since the melting point descriptor was never retained following feature selection, for any dataset, only those results generated without feature selection are considered.) However, none of those scenarios appeared to correspond to statistically significant differences in mean RMSE.

Contrastingly, for the corresponding Klimenko_CS_False (CV=rt) results, 10 out of 12 scenarios suggested performance enhancement due to the inclusion of the melting point descriptor. Eight of those scenarios appeared to correspond to statistically significant differences and were obtained using RFR or MLR and a variety of molecular descriptors. Of those eight scenarios, the largest reduction in mean RMSE was 0.14 with a corresponding increase in mean R^2 of 0.04, upon adding the melting point descriptor to the MLR model built using Absolv descriptors.

For the Avdeef_ExDPs_CS_True dataset, 17 out of 40 relevant pairwise comparisons suggested improved performance upon including the melting point descriptor, yet only one of those appeared to correspond to a statistically significant difference in mean RMSE. This result was obtained using RFR and conformer generator based 3D molecular descriptors, without the lattice energy descriptor, and corresponded to an increase in mean R^2 of 0.01 and a reduction in mean RMSE of 0.08. Similarly, for the Avdeef_ExDPs_Cal_CS_True dataset, 21 out of 40 relevant comparisons suggested the melting point descriptor improved

performance and none of those appeared to correspond to statistically significant differences in mean RMSE.

Contrastingly, for the relevant comparisons of the Klimenko_CS_True (CV=rt) results, the melting point descriptor appeared to enhance performance in 31 out of 40 cases and 23 of those cases appeared to correspond to statistically significant differences in mean RMSE. These 23 cases were obtained using RFR or MLR and a variety of other descriptors, including some cases with the crystal structure based 3D descriptors and/or the lattice energy descriptor. Of those 23 cases, the largest reduction in mean RMSE was 0.31 log units, with a corresponding increase in mean R^2 of 0.17, obtained using MLR, Absolv molecular descriptors and the lattice energy descriptor.

For the crystal structure integrated datasets, the proportions of apparently statistically significant improvements in mean RMSE, upon adding the melting point descriptor, were typically similar when consideration was restricted to those pairwise comparisons without additional solid state contribution descriptors (lattice energy or crystal structure based 3D descriptors). For the Avdeef_ExDPs_CS_True dataset, nine out of 16 comparisons showed an apparent improvement enhancement and one of these appeared to correspond to statistically significant differences in mean RMSE. For the Avdeef_ExDPs_Cal_CS_True dataset, ten out of 16 comparisons suggested enhanced performance upon adding the melting point descriptor, yet none of those appeared to correspond to statistically significant differences in mean RMSE. For the Klimenko_CS_True (CV=rt) results, 12 out of 16 comparisons suggested adding the melting point descriptor improved performance and nine of those corresponded to apparently statistically significant differences in mean RMSE.

Significance of the temperature descriptor

The importance of the (1/T) descriptor, in terms of its coefficient magnitude, was always close to the lowest for any descriptor for the evaluated MLR models (Table S4). (The temperature descriptor was never retained following feature selection, so analysis is only presented for models generated without feature selection.) Conversely, for the evaluated models built using the non-linear RFR algorithm (Table S5), the (1/T) descriptor was consistently in the top 20% of descriptors, excluding those models for which the molecular descriptors were based solely on the Absolv or Absolv and Ind (see **Table S1**) or 3D descriptor sets. However, to put this into context, the (1/T) descriptor was often ranked as less important than the lattice energy descriptor for the relevant subset of these models (Table S5).

These results can be explained by the van't Hoff relationship (see equation 1 in the main text), which posits that, if the standard enthalpy of solution is roughly constant over the relevant temperature (T) range, log10(solubility) should be linearly related to (1/T) for a given material, with the slope of the trend line being proportional to the standard enthalpy of solution. Hence, due to the variation in the standard enthalpy of solution across materials, a non-linear relationship will exist between (1/T) and log10(solubility) across materials.

It was possible to test whether these assumptions held for all materials in the Klimenko et al. [6] for which solubility data were available for more than two temperatures. Across the Klimenko_CS_False dataset, the magnitude of Pearson correlation coefficients for log10(solubility) against (1/T) varied between 1.00 (2dp) to 0.74, with a median value of 0.99 and 95% of all 121 values being greater than 0.95. Furthermore, for the 95% of materials with correlation magnitude greater than 0.95, the gradients of the best fit line vary from 445 to -3489 log10[molar concentration] K^{-1}. These results suggest considerable variation in the standard enthalpy of solution across materials. Full details are presented as part of Additional File 3.

Table S4. Importance (coefficient magnitude) of temperature descriptor for all of the MLR models built on the entirety of the Klimenko_CS_False and Klimenko_CS_True datasets, excluding those models built following feature selection. N.B. A lower rank means a more important descriptor and ties were assigned the lowest possible rank.

Dataset Name	Molecular Descriptors Combination	3D Descriptors Based on the Crystal Structure Included	Melting Point Descriptor Included	Lattice Energy Descriptor Included	Total no. descriptors	Coefficient	Rank	Rank / Total
Klimenko_CS_False	IntegSub, SiRMSSub, Absolv, Ind, Rdk	FALSE	FALSE	FALSE	3763	-1.16	3763	1.00
Klimenko_CS_False	IntegSub, SiRMSSub	FALSE	FALSE	FALSE	3557	-1.15	3540	1.00
Klimenko_CS_False	Rdk, Absolv	FALSE	FALSE	FALSE	203	-1.14	182	0.90

Klimenko_CS_False	Rdk	FALSE	FALSE	FALSE	197	-1.13	176	0.89
Klimenko_CS_False	Absolv, Ind	FALSE	FALSE	FALSE	11	-1.29	8	0.73
Klimenko_CS_False	Absolv	FALSE	FALSE	FALSE	7	-1.25	6	0.86
Klimenko_CS_False	IntegSub, SiRMSSub, Absolv, Ind, Rdk	FALSE	TRUE	FALSE	3764	-1.16	3763	1.00
Klimenko_CS_False	IntegSub, SiRMSSub	FALSE	TRUE	FALSE	3558	-1.17	3541	1.00
Klimenko_CS_False	Rdk, Absolv	FALSE	TRUE	FALSE	204	-1.17	176	0.86
Klimenko_CS_False	Rdk	FALSE	TRUE	FALSE	198	-1.15	174	0.88
Klimenko_CS_False	Absolv, Ind	FALSE	TRUE	FALSE	12	-1.42	8	0.67
Klimenko_CS_False	Absolv	FALSE	TRUE	FALSE	8	-1.43	7	0.88
Klimenko_CS_True	3D, IntegSub, SiRMSSub, Absolv, Ind, Rdk	FALSE	FALSE	FALSE	3806	-1.23	3806	1.00
Klimenko_CS_True	IntegSub, SiRMSSub, Absolv, Ind, Rdk	FALSE	FALSE	FALSE	3763	-1.23	3763	1.00
Klimenko_CS_True	IntegSub, SiRMSSub	FALSE	FALSE	FALSE	3557	-1.23	3553	1.00
Klimenko_CS_True	Rdk, Absolv	FALSE	FALSE	FALSE	203	-1.22	203	1.00
Klimenko_CS_True	Rdk	FALSE	FALSE	FALSE	197	-1.22	197	1.00
Klimenko_CS_True	3D	FALSE	FALSE	FALSE	44	-1.20	40	0.91
Klimenko_CS_True	Absolv, Ind	FALSE	FALSE	FALSE	11	-1.44	8	0.73
Klimenko_CS_True	Absolv	FALSE	FALSE	FALSE	7	-1.45	6	0.86
Klimenko_CS_True	3D, IntegSub, SiRMSSub, Absolv, Ind, Rdk	FALSE	FALSE	TRUE	3807	-1.23	3807	1.00
Klimenko_CS_True	IntegSub, SiRMSSub, Absolv, Ind, Rdk	FALSE	FALSE	TRUE	3764	-1.23	3764	1.00
Klimenko_CS_True	IntegSub, SiRMSSub	FALSE	FALSE	TRUE	3558	-1.23	3553	1.00
Klimenko_CS_True	Rdk, Absolv	FALSE	FALSE	TRUE	204	-1.22	204	1.00
Klimenko_CS_True	Rdk	FALSE	FALSE	TRUE	198	-1.22	198	1.00
Klimenko_CS_True	3D	FALSE	FALSE	TRUE	45	-1.22	41	0.91
Klimenko_CS_True	Absolv, Ind	FALSE	FALSE	TRUE	12	-1.44	9	0.75
Klimenko_CS_True	Absolv	FALSE	FALSE	TRUE	8	-1.46	7	0.88
Klimenko_CS_True	3D, IntegSub,	FALSE	TRUE	FALSE	3807	-1.23	3807	1.00

	SiRMSSub, Absolv, Ind, Rdk							
Klimenko_CS_True	IntegSub, SiRMSSub, Absolv, Ind, Rdk	FALSE	TRUE	FALSE	3764	-1.23	3764	1.00
Klimenko_CS_True	IntegSub, SiRMSSub	FALSE	TRUE	FALSE	3558	-1.22	3557	1.00
Klimenko_CS_True	Rdk, Absolv	FALSE	TRUE	FALSE	204	-1.22	204	1.00
Klimenko_CS_True	Rdk	FALSE	TRUE	FALSE	198	-1.22	198	1.00
Klimenko_CS_True	3D	FALSE	TRUE	FALSE	45	-1.21	41	0.91
Klimenko_CS_True	Absolv, Ind	FALSE	TRUE	FALSE	12	-1.51	8	0.67
Klimenko_CS_True	Absolv	FALSE	TRUE	FALSE	8	-1.50	7	0.88
Klimenko_CS_True	3D, IntegSub, SiRMSSub, Absolv, Ind, Rdk	FALSE	TRUE	TRUE	3808	-1.23	3808	1.00
Klimenko_CS_True	IntegSub, SiRMSSub, Absolv, Ind, Rdk	FALSE	TRUE	TRUE	3765	-1.23	3765	1.00
Klimenko_CS_True	IntegSub, SiRMSSub	FALSE	TRUE	TRUE	3559	-1.22	3555	1.00
Klimenko_CS_True	Rdk, Absolv	FALSE	TRUE	TRUE	205	-1.22	205	1.00
Klimenko_CS_True	Rdk	FALSE	TRUE	TRUE	199	-1.22	199	1.00
Klimenko_CS_True	3D	FALSE	TRUE	TRUE	46	-1.23	41	0.89
Klimenko_CS_True	Absolv, Ind	FALSE	TRUE	TRUE	13	-1.51	9	0.69
Klimenko_CS_True	Absolv	FALSE	TRUE	TRUE	9	-1.50	8	0.89
Klimenko_CS_True	3D, IntegSub, SiRMSSub, Absolv, Ind, Rdk	TRUE	FALSE	FALSE	3806	-1.23	3806	1.00
Klimenko_CS_True	3D	TRUE	FALSE	FALSE	44	-1.18	42	0.95
Klimenko_CS_True	3D, IntegSub, SiRMSSub, Absolv, Ind, Rdk	TRUE	FALSE	TRUE	3807	-1.23	3807	1.00
Klimenko_CS_True	3D	TRUE	FALSE	TRUE	45	-1.22	41	0.91
Klimenko_CS_True	3D, IntegSub, SiRMSSub, Absolv, Ind, Rdk	TRUE	TRUE	FALSE	3807	-1.23	3807	1.00
Klimenko_CS_True	3D	TRUE	TRUE	FALSE	45	-1.15	43	0.96
Klimenko_CS_True	3D, IntegSub,	TRUE	TRUE	TRUE	3808	-1.23	3808	1.00

	SiRMSSub, Absolv, Ind, Rdk							
Klimenko_CS_True	3D	TRUE	TRUE	TRUE	46	-1.18	41	0.89

Table S5. Importance of temperature descriptor for all of the RFR models built on the entirety of the Klimenko_CS_False and Klimenko_CS_True datasets, excluding those models built following feature selection, compared to the importance of the lattice energy descriptor. N.B. A lower rank means a more important descriptor and ties were assigned the lowest possible rank.

Dataset	Molecular Descriptors Combination	3D Descriptors Based on the Crystal Structure Included	Melting Point Descriptor Included	Lattice Energy Descriptor Included	Total no. Descriptors	Temperature Descriptor			Lattice Energy Descriptor		
						Importance	Rank	Rank / Total	Importance	Rank	Rank / Total
Klimenko_CS_False	Absolv	FALSE	FALSE	FALSE	7	0.13	7	1.00	N/A	N/A	N/A
Klimenko_CS_False	Absolv	FALSE	TRUE	FALSE	8	0.14	8	1.00	N/A	N/A	N/A
Klimenko_CS_False	Absolv, Ind	FALSE	FALSE	FALSE	11	0.12	8	0.73	N/A	N/A	N/A
Klimenko_CS_False	Absolv, Ind	FALSE	TRUE	FALSE	12	0.12	9	0.75	N/A	N/A	N/A
Klimenko_CS_False	IntegSub, SiRMSSub	FALSE	FALSE	FALSE	3557	0.06	25	0.01	N/A	N/A	N/A
Klimenko_CS_False	IntegSub, SiRMSSub	FALSE	TRUE	FALSE	3558	0.07	26	0.01	N/A	N/A	N/A
Klimenko_CS_False	IntegSub, SiRMSSub, Absolv, Ind, Rdk	FALSE	FALSE	FALSE	3763	0.06	18	0.00	N/A	N/A	N/A
Klimenko_CS_False	IntegSub, SiRMSSub, Absolv, Ind, Rdk	FALSE	TRUE	FALSE	3764	0.06	17	0.00	N/A	N/A	N/A
Klimenko_CS_False	Rdk	FALSE	FALSE	FALSE	197	0.06	27	0.14	N/A	N/A	N/A
Klimenko_CS_False	Rdk	FALSE	TRUE	FALSE	198	0.06	25	0.13	N/A	N/A	N/A
Klimenko_CS_False	Rdk, Absolv	FALSE	FALSE	FALSE	203	0.06	27	0.13	N/A	N/A	N/A
Klimenko_CS_False	Rdk, Absolv	FALSE	TRUE	FALSE	204	0.06	27	0.13	N/A	N/A	N/A
Klimenko_CS_True	3D	FALSE	FALSE	FALSE	44	0.08	26	0.59	N/A	N/A	N/A

Klimenko_CS_True	3D	FALSE	FALSE	TRUE	45	0.09	23	0.51	0.11	16	0.36
Klimenko_CS_True	3D	FALSE	TRUE	FALSE	45	0.09	19	0.42	N/A	N/A	N/A
Klimenko_CS_True	3D	FALSE	TRUE	TRUE	46	0.08	22	0.48	0.10	18	0.39
Klimenko_CS_True	3D	TRUE	FALSE	FALSE	44	0.08	23	0.52	N/A	N/A	N/A
Klimenko_CS_True	3D	TRUE	FALSE	TRUE	45	0.08	21	0.47	0.15	13	0.29
Klimenko_CS_True	3D	TRUE	TRUE	FALSE	45	0.08	22	0.49	N/A	N/A	N/A
Klimenko_CS_True	3D	TRUE	TRUE	TRUE	46	0.08	21	0.46	0.15	14	0.30
Klimenko_CS_True	3D, IntegSub, SiRMSSub, Absolv, Ind, Rdk	FALSE	FALSE	FALSE	3806	0.08	12	0.00	N/A	N/A	N/A
Klimenko_CS_True	3D, IntegSub, SiRMSSub, Absolv, Ind, Rdk	FALSE	FALSE	TRUE	3807	0.08	12	0.00	0.02	37	0.01
Klimenko_CS_True	3D, IntegSub, SiRMSSub, Absolv, Ind, Rdk	FALSE	TRUE	FALSE	3807	0.08	13	0.00	N/A	N/A	N/A
Klimenko_CS_True	3D, IntegSub, SiRMSSub, Absolv, Ind, Rdk	FALSE	TRUE	TRUE	3808	0.08	13	0.00	0.02	39	0.01
Klimenko_CS_True	3D, IntegSub, SiRMSSub, Absolv, Ind, Rdk	TRUE	FALSE	FALSE	3806	0.08	12	0.00	N/A	N/A	N/A
Klimenko_CS_True	3D, IntegSub, SiRMSSub, Absolv, Ind, Rdk	TRUE	FALSE	TRUE	3807	0.08	12	0.00	0.02	34	0.01
Klimenko_CS_True	3D, IntegSub, SiRMSSub, Absolv, Ind, Rdk	TRUE	TRUE	FALSE	3807	0.08	13	0.00	N/A	N/A	N/A
Klimenko_CS_True	3D, IntegSub, SiRMSSub, Absolv, Ind, Rdk	TRUE	TRUE	TRUE	3808	0.08	12	0.00	0.02	44	0.01
Klimenko_CS_True	Absolv	FALSE	FALSE	FALSE	7	0.12	7	1.00	N/A	N/A	N/A
Klimenko_CS_True	Absolv	FALSE	FALSE	TRUE	8	0.11	8	1.00	0.91	6	0.75
Klimenko_CS_True	Absolv	FALSE	TRUE	FALSE	8	0.11	8	1.00	N/A	N/A	N/A
Klimenko_CS_True	Absolv	FALSE	TRUE	TRUE	9	0.10	9	1.00	0.76	6	0.67

Klimenko_CS_True	Absolv, Ind	FALSE	FALSE	FALSE	11	0.11	8	0.73	N/A	N/A	N/A
Klimenko_CS_True	Absolv, Ind	FALSE	FALSE	TRUE	12	0.11	8	0.67	0.88	6	0.50
Klimenko_CS_True	Absolv, Ind	FALSE	TRUE	FALSE	12	0.11	8	0.67	N/A	N/A	N/A
Klimenko_CS_True	Absolv, Ind	FALSE	TRUE	TRUE	13	0.10	10	0.77	0.75	6	0.46
Klimenko_CS_True	IntegSub, SiRMSSub	FALSE	FALSE	FALSE	3557	0.09	18	0.01	N/A	N/A	N/A
Klimenko_CS_True	IntegSub, SiRMSSub	FALSE	FALSE	TRUE	3558	0.09	19	0.01	0.09	18	0.01
Klimenko_CS_True	IntegSub, SiRMSSub	FALSE	TRUE	FALSE	3558	0.09	20	0.01	N/A	N/A	N/A
Klimenko_CS_True	IntegSub, SiRMSSub	FALSE	TRUE	TRUE	3559	0.09	19	0.01	0.08	21	0.01
Klimenko_CS_True	IntegSub, SiRMSSub, Absolv, Ind, Rdk	FALSE	FALSE	FALSE	3763	0.08	11	0.00	N/A	N/A	N/A
Klimenko_CS_True	IntegSub, SiRMSSub, Absolv, Ind, Rdk	FALSE	FALSE	TRUE	3764	0.08	11	0.00	0.06	19	0.01
Klimenko_CS_True	IntegSub, SiRMSSub, Absolv, Ind, Rdk	FALSE	TRUE	FALSE	3764	0.08	12	0.00	N/A	N/A	N/A
Klimenko_CS_True	IntegSub, SiRMSSub, Absolv, Ind, Rdk	FALSE	TRUE	TRUE	3765	0.08	14	0.00	0.05	24	0.01
Klimenko_CS_True	Rdk	FALSE	FALSE	FALSE	197	0.08	19	0.10	N/A	N/A	N/A
Klimenko_CS_True	Rdk	FALSE	FALSE	TRUE	198	0.08	19	0.10	0.11	12	0.06
Klimenko_CS_True	Rdk	FALSE	TRUE	FALSE	198	0.08	19	0.10	N/A	N/A	N/A
Klimenko_CS_True	Rdk	FALSE	TRUE	TRUE	199	0.08	20	0.10	0.11	14	0.07
Klimenko_CS_True	Rdk, Absolv	FALSE	FALSE	FALSE	203	0.08	19	0.09	N/A	N/A	N/A
Klimenko_CS_True	Rdk, Absolv	FALSE	FALSE	TRUE	204	0.08	20	0.10	0.12	12	0.06
Klimenko_CS_True	Rdk, Absolv	FALSE	TRUE	FALSE	204	0.08	17	0.08	N/A	N/A	N/A
Klimenko_CS_True	Rdk, Absolv	FALSE	TRUE	TRUE	205	0.08	20	0.10	0.11	15	0.07

Significant molecular descriptors

The models built on the entirety of the datasets were evaluated (for each dataset, method, feature selection combination) to see which descriptors were consistently found to be the most important and, otherwise, which were found to be consistently in the top 10% of descriptors. The only scenarios in which any descriptors were consistently in the top 10% across models were a subset of those involving feature selection. For these scenarios, the descriptors in the top 10% were the top ranking descriptor – as, at most, only 10 descriptors were retained following feature selection. The consistently top ranking descriptors for these scenarios are reported in Table S6. It can be seen that these are all molecular descriptors, albeit no single molecular descriptor is consistently reported as the most important across all relevant scenarios.

Table S6. Descriptors found to be consistently the best across all models built, following feature selection, for each of these combinations of dataset and modelling method.

Dataset	Method	Consistent Best[a]	Description
Avdeef_ExDPs_CS_True	RFR	N/A	N/A
Avdeef_ExDPs_CS_True	MLR	N/A	N/A
Avdeef_ExDPs_CS_False	RFR	N/A	N/A
Avdeef_ExDPs_CS_False	MLR	Fr2(rf)/A_B/1_2s/	This is a SiRMS descriptor, denoting the occurrence count of a molecular substructure defined in terms of atomic labels based on atomic refraction [55, 56]. Since molar refraction is related to dispersion interactions [97] and the enthalpy of solution for water, a polar protic solvent, is being modelled, this probably reflects the importance of dispersion interactions in the solid state.
Avdeef_ExDPs_Cal_CS_True	RFR	N/A	N/A
Avdeef_ExDPs_Cal_CS_True	MLR	N/A	N/A
Avdeef_ExDPs_Cal_CS_False	RFR	Fr3(d_a)/A_I_I/1_3d,2_3s/	This is a SiRMS descriptor, denoting the occurrence count of a molecular substructure defined in terms of atomic labels based on hydrogen bond donor / acceptor status [55, 56]. Hence, it would be related to both solid state and non-solid state contributions to the enthalpy of solution.

Avdeef_ExDPs_Cal_CS_False	MLR	S_A(rep)/A_A_B_B/1_4s,2_4s,3_4s/5	This is a SiRMS descriptor, denoting the occurrence count of a molecular substructure defined in terms of atomic labels based on van der Waals repulsion [55, 56] according to the UFF [57] forcefield.. Since enthalpy of solution for water, a polar protic solvent, is being modelled and van der Waals repulsion is likely to be significant as an offset for attractive dispersion forces [57], this probably reflects the importance of dispersion interactions in the solid state.
Klimenko_CS_True	RFR	MolMR	Rdk descriptor [53], denoting an estimator of molar refractivity (a synonym for molar refraction [98]) according to the approach of Wildman and Crippen [99]. This is related to the ability of the solute to form dispersion interactions [97]. Given that solubility in water, a polar protic solvent, is being modelled, this probably reflects the importance of dispersion interactions in the solid state..
Klimenko_CS_True	MLR	MolMR	
Klimenko_CS_False	RFR	MolLogP	Rdk descriptor [53], denoting an estimator of logP according to the approach of Wildman and Crippen [99]. This quantifies the degree to which solute molecules would be distributed, at equilibrium, between octanol and water. Hence it reflects, in part, the degree to which thermodynamically favourable interactions can be formed with the aqueous solvent (non-solid state contribution).
Klimenko_CS_False	MLR	NumValenceElectrons	Rdk descriptor [53], denoting the number of valence electrons. This may be a proxy for a number of different solid state and non-solid state contributions to solubility.

a. The original name is provided. In the course of preparing the QSPR input files, "D_" was added as a prefix, colons and brackets were replaced with underscores ("_"), commas (",") were replaced with ".comma.", spaces were contracted, plus and minus symbols were replaced with literal "_plus_" and "_minus_" statements respectively and any dash symbol ("-") followed by a lower case letter was replaced with that lower case letter. Subsequently, the modelling workflow further transformed these names, e.g. replacing "/" with ".".

Lattice energy molecular descriptor models: prediction outliers

The nine prediction outliers, from cross-validating the molecular descriptor model on the Avdeef_ExDPs_CS_True dataset, corresponded to the following CSD refcodes: PHTHAC06, TEPHTH13, BENZDC01, DAPSUO05, FPAMCA, DHXBZP, CAXMUJ, PCPTZA, PTOLIC01.

Lattice energy molecular descriptor models: descriptor importance analysis

Six of the top 10 important descriptors were the same for the lattice energy models built on the Avdeef_ExDPs_CS_True dataset, before and after removing the outliers. Four of these descriptors were also amongst the 10 most important for the model built based on the Klimenko_CS_True dataset: Kappa1 (molecular shape index) [100, 101], Chi0 (molecular connectivity index, which may be related to the degree of branching) [100, 101], TPSA (total polar surface area) [102], AxB (product of Absolv descriptors, representing the potential for solute - solute hydrogen bonding) [4, 51]. All of these descriptors may be related to the ability to form close contacts in the solid state or the potential for hydrogen bonding or dipole – based intermolecular interactions in the solid state. Curiously, only the topological indices are also amongst the top 10 descriptors when this analysis is applied to the Avdeef_ExDPs_Cal_CS_True dataset. Again, this may simply reflect the smaller size of that dataset.

References

1. Skyner RE, McDonagh JL, Groom CR, et al (2015) A review of methods for the calculation of solution free energies and the modelling of systems in solution. Phys Chem Chem Phys 17:6174–6191 . doi: 10.1039/C5CP00288E

2. Groom CR, Bruno IJ, Lightfoot MP, Ward SC (2016) The Cambridge Structural Database. Acta Cryst B, Acta Cryst Sect B, Acta Crystallogr B, Acta Crystallogr Sect B, Acta Crystallogr B Struct Crystallogr Cryst Chem, Acta Crystallogr Sect B Struct Crystallogr Cryst Chem 72:171–179 . doi: 10.1107/S2052520616003954

3. Prankerd RJ, McKeown RH (1990) Physico-chemical properties of barbituric acid derivatives Part I. Solubility-temperature dependence for 5,5-disubstituted barbituric acids in aqueous solutions. International Journal of Pharmaceutics 62:37–52 . doi: 10.1016/0378-5173(90)90029-4

4. Avdeef A (2015) Solubility Temperature Dependence Predicted from 2D Structure. ADMET & DMPK 3: . doi: 10.5599/admet.3.4.259

5. Rosbottom I, Ma CY, Turner TD, et al (2017) Influence of Solvent Composition on the Crystal Morphology and Structure of p-Aminobenzoic Acid Crystallized from Mixed Ethanol and Nitromethane Solutions. Crystal Growth & Design 17:4151–4161 . doi: 10.1021/acs.cgd.7b00425

6. Klimenko K, Kuz'min V, Ognichenko L, et al (2016) Novel enhanced applications of QSPR models: Temperature dependence of aqueous solubility. J Comput Chem 37:2045–2051 . doi: 10.1002/jcc.24424

7. Yalkowsky SH, He Y, Jain P (2010) Handbook of Aqueous Solubility Data, Second Edition, 2nd ed. CRC Press

8. Yalkowsky SH (2003) Handbook of Aqueous Solubility Data, 1st ed. CRC Press

9. Common Chemistry - Search Chemical Names and CAS Registry Numbers. http://www.commonchemistry.org/. Accessed 21 Jul 2017

10. Heller SR, McNaught A, Pletnev I, et al (2015) InChI, the IUPAC International Chemical Identifier. Journal of Cheminformatics 7:23 . doi: 10.1186/s13321-015-0068-4

11. Pybel (version 1.8) Download Link from SourceForge.net. https://sourceforge.net/projects/openbabel/files/openbabel-python/1.8/openbabel-python-1.8.py27.exe/download. Accessed 30 Sep 2013

12. Open Babel (version 2.32a) Download Link from SourceForge.net. https://sourceforge.net/projects/openbabel/files/openbabel/2.3.2/OpenBabel2.3.2a_Windows_Installer.exe/download. Accessed 27 Oct 2013

13. O'Boyle N, Morley C, Hutchison G (2008) Pybel: a Python wrapper for the OpenBabel cheminformatics toolkit. Chem Cent J 2:5 . doi: 10.1186/1752-153X-2-5

14. O'Boyle N, Banck M, James CA, et al (2011) Open Babel: An open chemical toolbox. J Cheminf 3:33

15. NCI/CADD Chemical Identifier Resolver. https://cactus.nci.nih.gov/chemical/structure. Accessed 21 Jul 2017

16. CIRpy (version 1.0.2). http://cirpy.readthedocs.io/en/latest/index.html. Accessed 21 Jul 2017

17. ChemSpider | Search and share chemistry. http://www.chemspider.com/. Accessed 21 Jul 2017

18. ChemSpiPy (version 1.0.4). http://chemspipy.readthedocs.io/en/latest/guide/intro.html. Accessed 21 Jul 2017

19. Kim S, Thiessen PA, Bolton EE, et al (2016) PubChem Substance and Compound databases. Nucleic Acids Res 44:D1202–D1213 . doi: 10.1093/nar/gkv951

20. The PubChem Project. http://pubchem.ncbi.nlm.nih.gov/. Accessed 24 Nov 2011

21. PubChemPy (version 1.0.3). http://pubchempy.readthedocs.io/en/latest/guide/introduction.html. Accessed 21 Jul 2017

22. Chemicalize - Instant Cheminformatics Solutions. https://chemicalize.com/welcome. Accessed 24 Jul 2017

23. CDK Depict: SMILES Depictor. http://cdkdepict-openchem.rhcloud.com/depict.html

24. Berman HM, Westbrook J, Feng Z, et al (2000) The Protein Data Bank. Nucleic Acids Res 28:235–242 . doi: 10.1093/nar/28.1.235

25. RCSB Protein Data Bank - RCSB PDB. http://www.rcsb.org/pdb/home/home.do. Accessed 1 May 2016

26. CSD Python API (version 1.0.0). Quick primer to using the CSD Python API. https://downloads.ccdc.cam.ac.uk/documentation/API/descriptive_docs/primer.html. Accessed 24 Jul 2017

27. Steed J Introduction | Z'. http://zprime.co.uk/. Accessed 25 Jul 2017

28. Steed J (2003) Should solid-state molecular packing have to obey the rules of crystallographic symmetry? CrystEngComm 5:169–179 . doi: 10.1039/B304631A

29. Unidecode (version 0.04.19). https://pypi.python.org/pypi/Unidecode. Accessed 24 Jul 2017

30. Macrae CF, Bruno IJ, Chisholm JA, et al (2008) Mercury CSD 2.0 – new features for the visualization and investigation of crystal structures. J Appl Cryst, J Appl Crystallogr 41:466–470 . doi: 10.1107/S0021889807067908

31. Zencirci N, Griesser UJ, Gelbrich T, et al (2014) Crystal Polymorphs of Barbital: News about a Classic Polymorphic System. Mol Pharmaceutics 11:338–350 . doi: 10.1021/mp400515f

32. Abu Bakar MR, Nagy ZK, Rielly CD, Dann SE (2011) Investigation of the riddle of sulfathiazole polymorphism. International Journal of Pharmaceutics 414:86–103 . doi: 10.1016/j.ijpharm.2011.05.004

33. CSD Python API (version 1.3.0). Quick primer to using the CSD Python API. https://downloads.ccdc.cam.ac.uk/documentation/API/descriptive_docs/primer.html. Accessed 24 Jul 2017

34. McDonagh JL, Palmer DS, Mourik T van, Mitchell JBO (2016) Are the Sublimation Thermodynamics of Organic Molecules Predictable? J Chem Inf Model 56:2162–2179 . doi: 10.1021/acs.jcim.6b00033

35. BIOVIA Materials Studio 2017 (17.1.0.48). http://accelrys.com/products/collaborative-science/biovia-materials-studio/. Accessed 25 Jul 2017

36. Hall SR, Allen FH, Brown ID (1991) The crystallographic information file (CIF): a new standard archive file for crystallography. Acta Crystallographica Section A Foundations of Crystallography 47:655–685 . doi: 10.1107/S010876739101067X

37. Weininger D (1988) SMILES, a chemical language and information system. 1. Introduction to methodology and encoding rules. J Chem Inf Comput Sci 28:31–36 . doi: 10.1021/ci00057a005

38. Sun H (1998) COMPASS: An ab Initio Force-Field Optimized for Condensed-Phase ApplicationsOverview with Details on Alkane and Benzene Compounds. J Phys Chem B 102:7338–7364 . doi: 10.1021/jp980939v

39. Sun H, Ren P, Fried JR (1998) The COMPASS force field: parameterization and validation for phosphazenes. Computational and Theoretical Polymer Science 8:229–246 . doi: 10.1016/S1089-3156(98)00042-7

40. Rigby D, Sun H, Eichinger BE (1997) Computer simulations of poly(ethylene oxide): force field, pvt diagram and cyclization behaviour. Polym Int 44:311–330 . doi: 10.1002/(SICI)1097-0126(199711)44:3<311::AID-PI880>3.0.CO;2-H

41. RDKit (version 2016.03.1). http://www.rdkit.org/. Accessed 25 Jul 2017

42. standardiser (version 0.1.7). https://pypi.python.org/pypi/standardiser. Accessed 25 Jul 2017

43. Carrió P, López O, Sanz F, Pastor M (2015) eTOXlab, an open source modeling framework for implementing predictive models in production environments. Journal of Cheminformatics 7:8 . doi: 10.1186/s13321-015-0058-6

44. standardiser module description. https://wwwdev.ebi.ac.uk/chembl/extra/francis/standardiser/. Accessed 25 Jul 2017

45. rules: apply structure-normalisation transformations. https://wwwdev.ebi.ac.uk/chembl/extra/francis/standardiser/03_rules.html. Accessed 25 Jul 2017

46. Dalby A, Nourse JG, Hounshell WD, et al (1992) Description of several chemical structure file formats used by computer programs developed at Molecular Design Limited. Journal of Chemical Information and Computer Sciences 32:244–255 . doi: 10.1021/ci00007a012

47. Percepta Desktop Program (ACD/Labs 2016 release, Build 2911. 12 Jul 2016). http://www.acdlabs.com/products/percepta/. Accessed 26 Jul 2017

48. HiT-QSAR Software (version 4.1.2.270). http://www.qsar4u.com/pages/sirms.php. Accessed 26 Jul 2017

49. Abraham MH (1993) Scales of solute hydrogen-bonding: their construction and application to physicochemical and biochemical processes. Chem Soc Rev 22:73–83 . doi: 10.1039/CS9932200073

50. Abraham MH, McGowan JC (1987) The use of characteristic volumes to measure cavity terms in reversed phase liquid chromatography. Chromatographia 23:243–246 . doi: 10.1007/BF02311772

51. Abraham MH, Le J (1999) The correlation and prediction of the solubility of compounds in water using an amended solvation energy relationship. Journal of Pharmaceutical Sciences 88:868–880 . doi: 10.1021/js9901007

52. Landrum G Feature Definitions Used in the Morgan Fingerprints. http://www.rdkit.org/docs/GettingStartedInPython.html#feature-definitions-used-in-the-morgan-fingerprints. Accessed 3 May 2017

53. List of Available Descriptors. http://www.rdkit.org/docs/GettingStartedInPython.html#list-of-available-descriptors. Accessed 26 Jul 2017

54. Kolumbin OG, Ognichenko LN, Artemenko AG, et al (2013) Nonexperimental screening of the water solubility, lipophilicity, bioavailability, mutagenicity and toxicity of various pesticides with QSAR models aid. Chemistry Journal of Moldova 8:95–100 . doi: 10.19261/cjm.2013.08(1).12

55. Kuz'min VE, Artemenko AG, Lozitsky VP, et al (2002) The analysis of structure-anticancer and antiviral activity relationships for macrocyclic pyridinophanes and their analogues on the basis of 4D QSAR models (simplex representation of molecular structure). Acta Biochim Pol 49:157–168

56. Kuz'min VE, Artemenko AG, Polischuk PG, et al (2005) Hierarchic system of QSAR models (1D–4D) on the base of simplex representation of molecular structure. J Mol Model 11:457–467 . doi: 10.1007/s00894-005-0237-x

57. Rappe AK, Casewit CJ, Colwell KS, et al (1992) UFF, a full periodic table force field for molecular mechanics and molecular dynamics simulations. J Am Chem Soc 114:10024–10035 . doi: 10.1021/ja00051a040

58. (2018) Mordred: a molecular descriptor calculator (version 1.0.0). https://github.com/mordred-descriptor/mordred

59. Low Y, Uehara T, Minowa Y, et al (2011) Predicting Drug-Induced Hepatotoxicity Using QSAR and Toxicogenomics Approaches. Chem Res Toxicol 24:1251–1262 . doi: 10.1021/tx200148a

60. Hawkins DM (2004) The Problem of Overfitting. Journal of Chemical Information and Computer Sciences 44:1–12 . doi: 10.1021/ci0342472

61. Jensen F (2007) Chapter 17: Statistics and QSAR. In: Introduction to Computational Chemistry, 2nd ed. John Wiley & Sons Ltd, pp 547–561

62. Dearden JC, Cronin MTD, Kaiser KLE (2009) How not to develop a quantitative structure–activity or structure–property relationship (QSAR/QSPR). SAR QSAR Environ Res 20:241–266 . doi: 10.1080/10629360902949567

63. Ferreira AJ, Figueiredo MAT (2012) Efficient feature selection filters for high-dimensional data. Pattern Recognition Letters 33:1794–1804 . doi: 10.1016/j.patrec.2012.05.019

64. M.A. Hall (1999) Correlation-based Feature Selection for Machine Learning, PhD Thesis. The University of Waikato

65. Guha R, Willighagen E (2012) A Survey of Quantitative Descriptions of Molecular Structure. Current Topics in Medicinal Chemistry 12:1946–1956 . doi: 10.2174/156802661121218000２

66. Guyon I, Elisseeff A (2003) An Introduction to Variable and Feature Selection. Journal of Machine Learning Research 3:1157–1182

67. SciPy (version 0.19.0) Reference Guide: scipy.cluster.hierarchy.linkage Documentation. https://docs.scipy.org/doc/scipy-0.19.0/reference/generated/scipy.cluster.hierarchy.linkage.html#scipy.cluster.hierarchy.linkage. Accessed 6 Sep 2017

68. Müllner D (2011) Modern hierarchical, agglomerative clustering algorithms. arXiv:11092378 [cs, stat]

69. Kraskov A, Stögbauer H, Grassberger P (2004) Estimating mutual information. Phys Rev E 69:66138 . doi: 10.1103/PhysRevE.69.066138

70. Ross BC (2014) Mutual Information between Discrete and Continuous Data Sets. PLOS ONE 9:e87357 . doi: 10.1371/journal.pone.0087357

71. Pedregosa F, Varoquaux G, Gramfort A, et al (2011) Scikit-learn: Machine Learning in Python. J Mach Learn Res 12:2825−2830

72. SciKit-Learn (version 0.18.1). http://scikit-learn.org. Accessed 27 Jul 2017

73. NumPy (version 1.11.3). http://www.numpy.org/. Accessed 8 Aug 2017

74. pandas (version 0.20.1). http://pandas.pydata.org/. Accessed 8 Aug 2017

75. Jones E, Oliphant E, Peterson P, et al. SciPy (version 0.19.0). https://scipy.org/. Accessed 27 Jul 2017

76. Kraskov A, Stögbauer H, Andrzejak RG, Grassberger P (2003) Hierarchical Clustering Based on Mutual Information. arXiv:q-bio/0311039

77. Cover TM, Thomas JA (1991) Elements of Information Theory. John Wiley and Sons, New York, NY, USA

78. D. J.C. MacKay (2003). In: Information Theory, Inference and Learning Algorithms. Cambridge University Press, p p.479

79. Svetnik V, Liaw A, Tong C, et al (2003) Random Forest: A Classification and Regression Tool for Compound Classification and QSAR Modeling. J Chem Inf Comput Sci 43:1947–1958 . doi: 10.1021/ci034160g

80. Breiman L (2001) Random Forests. Mach Learn 45:5–32 . doi: 10.1023/A:1010933404324

81. Liaw A The R randomForest Package (version 4.6-12). https://CRAN.R-project.org/package=randomForest. Accessed 27 Jul 2017

82. Liaw A, Wiener M (2002) Classification and Regression by randomForest. R News 2/3:18–22

83. The R Stats Package (version 3.3.2). https://stat.ethz.ch/R-manual/R-devel/library/stats/html/00Index.html. Accessed 27 Jul 2017

84. Kuhn M Building Predictive Models in R Using the caret Package. Journal of Statistical Software 28:

85. The R caret Package (version 6.0-73). https://cran.r-project.org/package=caret. Accessed 27 Jul 2017

86. Nichols TE, Holmes AP (2002) Nonparametric permutation tests for functional neuroimaging: A primer with examples. Hum Brain Mapp 15:1–25 . doi: 10.1002/hbm.1058

87. McDonagh JL, Nath N, De Ferrari L, et al (2014) Uniting Cheminformatics and Chemical Theory To Predict the Intrinsic Aqueous Solubility of Crystalline Druglike Molecules. J Chem Inf Model 54:844–856 . doi: 10.1021/ci4005805

88. Menke J, Martinez TR (2004) Using permutations instead of student's t distribution for p-values in paired-difference algorithm comparisons. In: 2004 IEEE International Joint Conference on Neural Networks (IEEE Cat. No.04CH37541). pp 1331–1335 vol.2

89. Dudoit S, Popper Shaffer J, Boldrick JC (2003) Multiple Hypothesis Testing in Microarray Experiments. Statist Sci 18:71–103

90. Benjamini Y, Yekutieli D (2001) The control of the false discovery rate in multiple testing under dependency. The Annals of Statistics 29:1165–1188 . doi: 10.1214/aos/1013699998

91. Breiman L (2001) Random Forests. Mach Learn 45:5–32 . doi: 10.1023/A:1010933404324

92. Bengio Y, Grandvalet Y (2004) No Unbiased Estimator of the Variance of K-Fold Cross-Validation. J Mach Learn Res 5:1089–1105

93. Nadeau C, Bengio Y (2003) Inference for the Generalization Error. Mach Learn 52:239–281

94. statsmodels (version 0.6.1). http://www.statsmodels.org/devel/index.html. Accessed 18 Oct 2017

95. RDKit (version 2017.03.1). http://www.rdkit.org/. Accessed 25 Jul 2017

96. Riniker S, Landrum GA (2015) Better Informed Distance Geometry: Using What We Know To Improve Conformation Generation. J Chem Inf Model 55:2562–2574 . doi: 10.1021/acs.jcim.5b00654

97. Miles DW, Urry DW (1968) Reciprocal Relations and Proximity of Bases in Flavin-Adenine Dinucleotide. Biochemistry 7:2791–2799

98. Sarkar L, Roy MN (2009) Studies on liquid–liquid interactions of some ternary mixtures by density, viscosity, ultrasonic speed and refractive index measurements. Thermochimica Acta 496:124–128 . doi: 10.1016/j.tca.2009.07.011

99. Wildman SA, Crippen GM (1999) Prediction of Physicochemical Parameters by Atomic Contributions. J Chem Inf Comput Sci 39:868–873 . doi: 10.1021/ci990307l

100. Hall LH, Kier LB (1991) The Molecular Connectivity Chi Indexes and Kappa Shape Indexes in Structure-Property Modeling. In: Lipkowitz KB, Boyd DB (eds) Reviews in Computational Chemistry. John Wiley & Sons, Inc., pp 367–422

101. QuaSAR-Descriptor: Documentation for Descriptors Calculated by the MOE Software Program. https://www.chemcomp.com/journal/descr.htm#KH. Accessed 6 Sep 2017

102. Ertl P, Rohde B, Selzer P (2000) Fast Calculation of Molecular Polar Surface Area as a Sum of Fragment-Based Contributions and Its Application to the Prediction of Drug Transport Properties. J Med Chem 43:3714–3717 . doi: 10.1021/jm000942e
