# Supplementary figures and images for: The influence of solid state information and descriptor selection on statistical models of temperature dependent aqueous solubility (part 1 of 2)
Source: J Cheminform. 2018 Aug 29;10:44. doi: 10.1186/s13321-018-0298-3 (PMC6115327; doi:10.1186/s13321-018-0298-3)

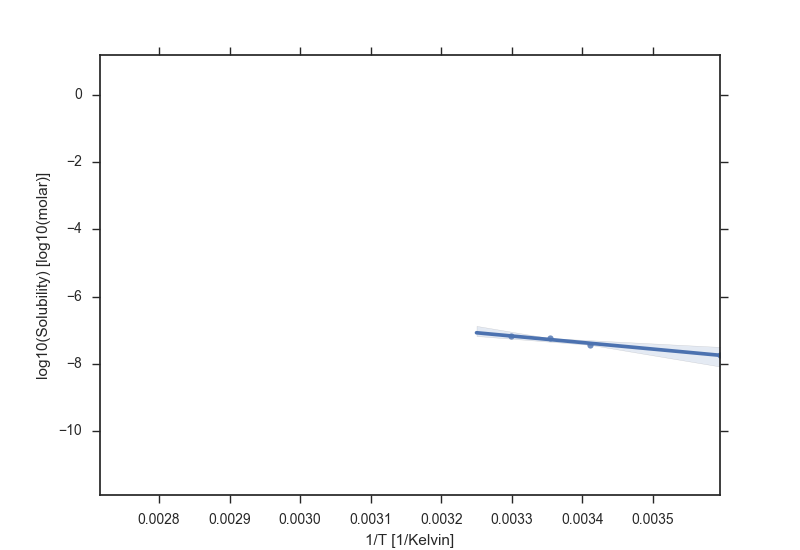

Supplement: Supplementary file 3 — Additional file 3. Additional results files, in electronic format. These additional results are (a) SUB-48 calculated lattice energies for the complete set and filtered set of 27 crystal structures, (b) results from analysis of the correspondence between our temperature dependent solubility data and (1/T), (c) Excel workbooks documenting all R2 and RMSE values obtained from cross-validation, their mean values and the p values (both raw and adjusted) obtained from pairwise comparisons of corresponding models, (d) comparison of melting point data used for the melting point descriptor and retrieved from the CSD for linked refcodes. [file 13321_2018_298_MOESM3_ESM.zip › additional_results/vantHoff_assessment/vh_plots/Klimenko_QSPRin_CD_False_MD_IntegSub.SiRMSSub.Absolv.Ind.Rdk_readyForR_vh.csv_100.tiff]

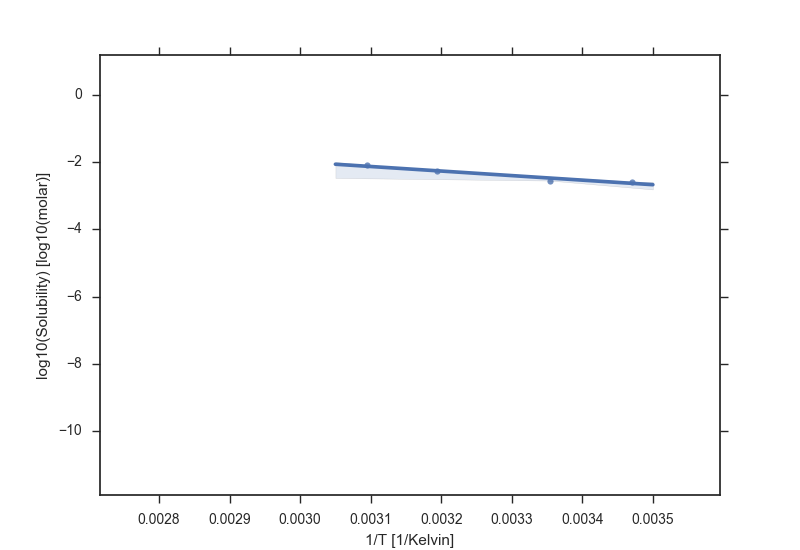

Supplement: Supplementary file 3 — Additional file 3. Additional results files, in electronic format. These additional results are (a) SUB-48 calculated lattice energies for the complete set and filtered set of 27 crystal structures, (b) results from analysis of the correspondence between our temperature dependent solubility data and (1/T), (c) Excel workbooks documenting all R2 and RMSE values obtained from cross-validation, their mean values and the p values (both raw and adjusted) obtained from pairwise comparisons of corresponding models, (d) comparison of melting point data used for the melting point descriptor and retrieved from the CSD for linked refcodes. [file 13321_2018_298_MOESM3_ESM.zip › additional_results/vantHoff_assessment/vh_plots/Klimenko_QSPRin_CD_False_MD_IntegSub.SiRMSSub.Absolv.Ind.Rdk_readyForR_vh.csv_101.tiff]

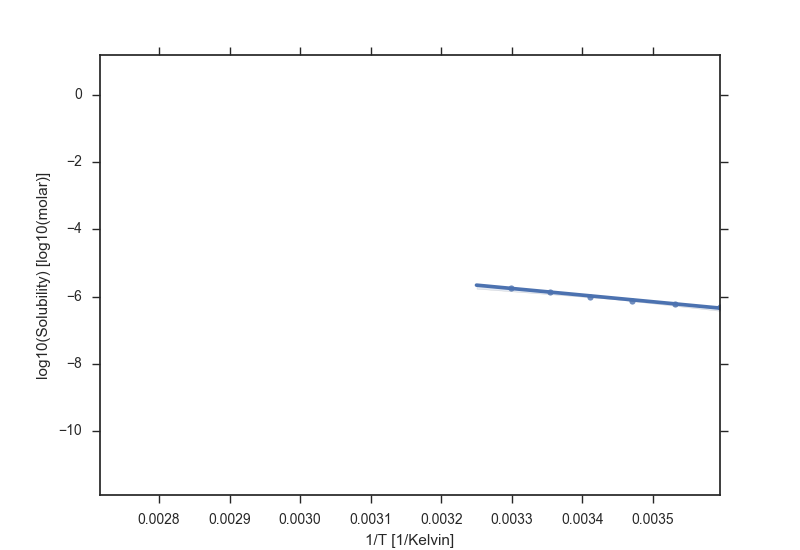

Supplement: Supplementary file 3 — Additional file 3. Additional results files, in electronic format. These additional results are (a) SUB-48 calculated lattice energies for the complete set and filtered set of 27 crystal structures, (b) results from analysis of the correspondence between our temperature dependent solubility data and (1/T), (c) Excel workbooks documenting all R2 and RMSE values obtained from cross-validation, their mean values and the p values (both raw and adjusted) obtained from pairwise comparisons of corresponding models, (d) comparison of melting point data used for the melting point descriptor and retrieved from the CSD for linked refcodes. [file 13321_2018_298_MOESM3_ESM.zip › additional_results/vantHoff_assessment/vh_plots/Klimenko_QSPRin_CD_False_MD_IntegSub.SiRMSSub.Absolv.Ind.Rdk_readyForR_vh.csv_104.tiff]

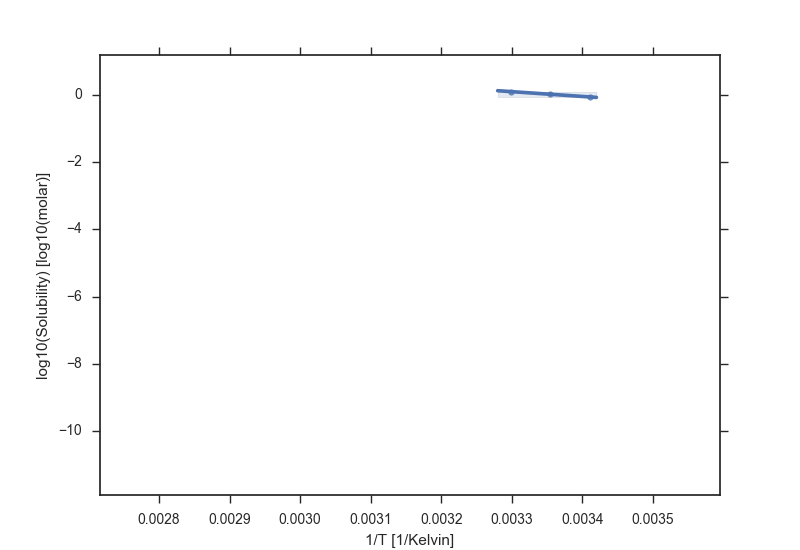

Supplement: Supplementary file 3 — Additional file 3. Additional results files, in electronic format. These additional results are (a) SUB-48 calculated lattice energies for the complete set and filtered set of 27 crystal structures, (b) results from analysis of the correspondence between our temperature dependent solubility data and (1/T), (c) Excel workbooks documenting all R2 and RMSE values obtained from cross-validation, their mean values and the p values (both raw and adjusted) obtained from pairwise comparisons of corresponding models, (d) comparison of melting point data used for the melting point descriptor and retrieved from the CSD for linked refcodes. [file 13321_2018_298_MOESM3_ESM.zip › additional_results/vantHoff_assessment/vh_plots/Klimenko_QSPRin_CD_False_MD_IntegSub.SiRMSSub.Absolv.Ind.Rdk_readyForR_vh.csv_114.tiff]

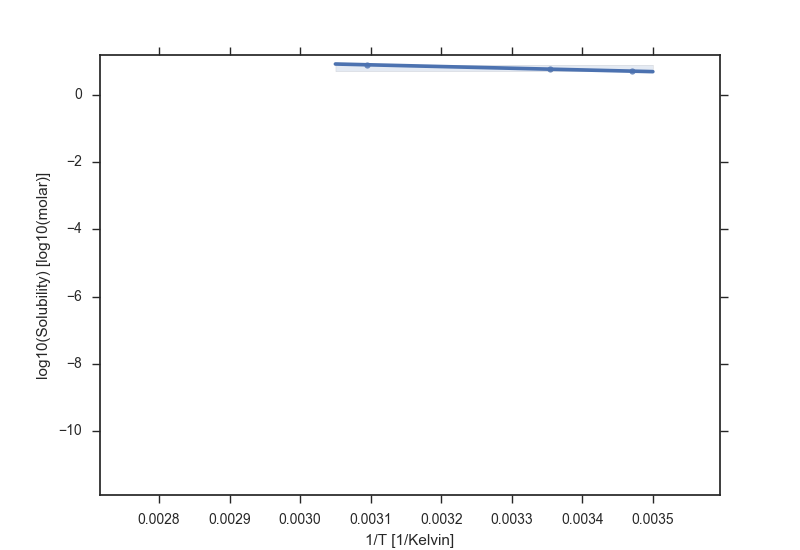

Supplement: Supplementary file 3 — Additional file 3. Additional results files, in electronic format. These additional results are (a) SUB-48 calculated lattice energies for the complete set and filtered set of 27 crystal structures, (b) results from analysis of the correspondence between our temperature dependent solubility data and (1/T), (c) Excel workbooks documenting all R2 and RMSE values obtained from cross-validation, their mean values and the p values (both raw and adjusted) obtained from pairwise comparisons of corresponding models, (d) comparison of melting point data used for the melting point descriptor and retrieved from the CSD for linked refcodes. [file 13321_2018_298_MOESM3_ESM.zip › additional_results/vantHoff_assessment/vh_plots/Klimenko_QSPRin_CD_False_MD_IntegSub.SiRMSSub.Absolv.Ind.Rdk_readyForR_vh.csv_115.tiff]

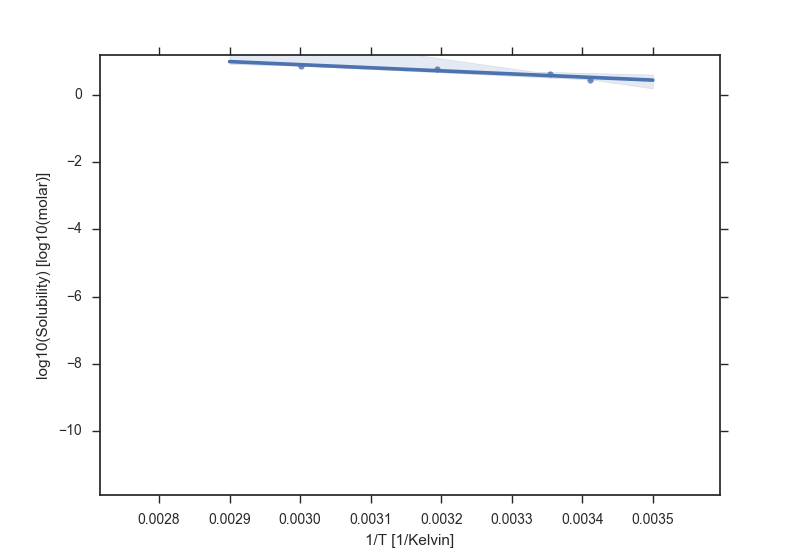

Supplement: Supplementary file 3 — Additional file 3. Additional results files, in electronic format. These additional results are (a) SUB-48 calculated lattice energies for the complete set and filtered set of 27 crystal structures, (b) results from analysis of the correspondence between our temperature dependent solubility data and (1/T), (c) Excel workbooks documenting all R2 and RMSE values obtained from cross-validation, their mean values and the p values (both raw and adjusted) obtained from pairwise comparisons of corresponding models, (d) comparison of melting point data used for the melting point descriptor and retrieved from the CSD for linked refcodes. [file 13321_2018_298_MOESM3_ESM.zip › additional_results/vantHoff_assessment/vh_plots/Klimenko_QSPRin_CD_False_MD_IntegSub.SiRMSSub.Absolv.Ind.Rdk_readyForR_vh.csv_119.tiff]

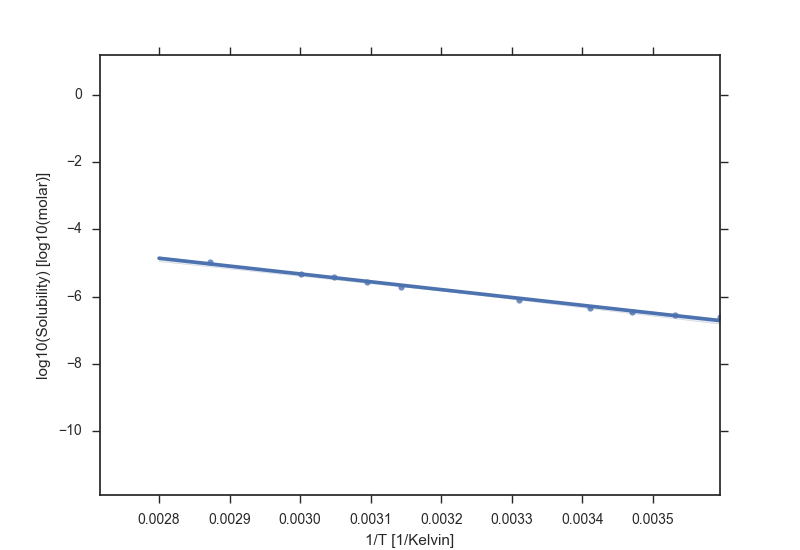

Supplement: Supplementary file 3 — Additional file 3. Additional results files, in electronic format. These additional results are (a) SUB-48 calculated lattice energies for the complete set and filtered set of 27 crystal structures, (b) results from analysis of the correspondence between our temperature dependent solubility data and (1/T), (c) Excel workbooks documenting all R2 and RMSE values obtained from cross-validation, their mean values and the p values (both raw and adjusted) obtained from pairwise comparisons of corresponding models, (d) comparison of melting point data used for the melting point descriptor and retrieved from the CSD for linked refcodes. [file 13321_2018_298_MOESM3_ESM.zip › additional_results/vantHoff_assessment/vh_plots/Klimenko_QSPRin_CD_False_MD_IntegSub.SiRMSSub.Absolv.Ind.Rdk_readyForR_vh.csv_123.tiff]

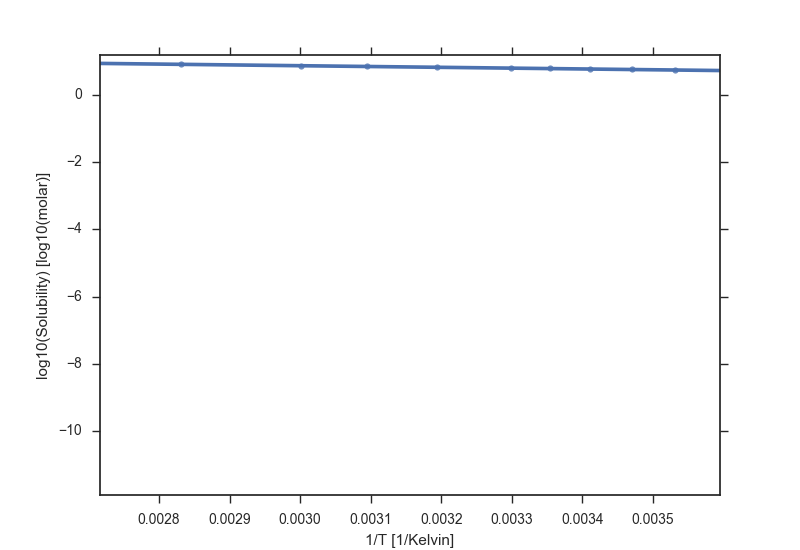

Supplement: Supplementary file 3 — Additional file 3. Additional results files, in electronic format. These additional results are (a) SUB-48 calculated lattice energies for the complete set and filtered set of 27 crystal structures, (b) results from analysis of the correspondence between our temperature dependent solubility data and (1/T), (c) Excel workbooks documenting all R2 and RMSE values obtained from cross-validation, their mean values and the p values (both raw and adjusted) obtained from pairwise comparisons of corresponding models, (d) comparison of melting point data used for the melting point descriptor and retrieved from the CSD for linked refcodes. [file 13321_2018_298_MOESM3_ESM.zip › additional_results/vantHoff_assessment/vh_plots/Klimenko_QSPRin_CD_False_MD_IntegSub.SiRMSSub.Absolv.Ind.Rdk_readyForR_vh.csv_126.tiff]

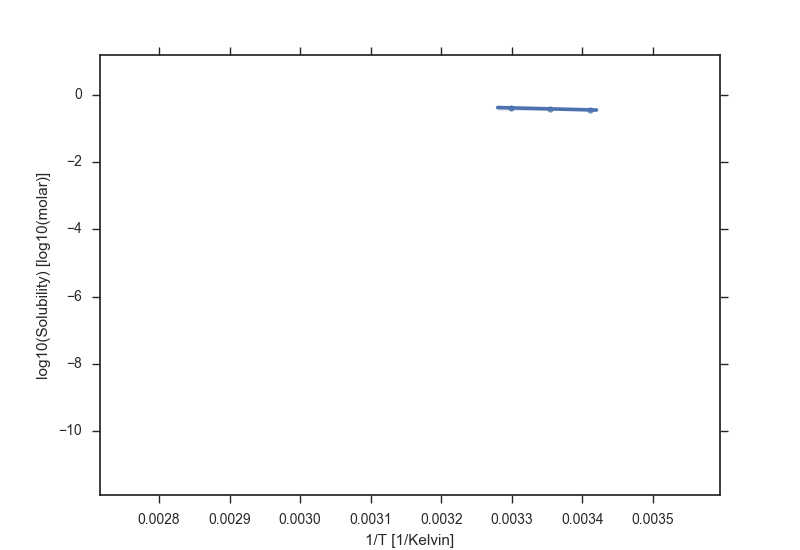

Supplement: Supplementary file 3 — Additional file 3. Additional results files, in electronic format. These additional results are (a) SUB-48 calculated lattice energies for the complete set and filtered set of 27 crystal structures, (b) results from analysis of the correspondence between our temperature dependent solubility data and (1/T), (c) Excel workbooks documenting all R2 and RMSE values obtained from cross-validation, their mean values and the p values (both raw and adjusted) obtained from pairwise comparisons of corresponding models, (d) comparison of melting point data used for the melting point descriptor and retrieved from the CSD for linked refcodes. [file 13321_2018_298_MOESM3_ESM.zip › additional_results/vantHoff_assessment/vh_plots/Klimenko_QSPRin_CD_False_MD_IntegSub.SiRMSSub.Absolv.Ind.Rdk_readyForR_vh.csv_129.tiff]

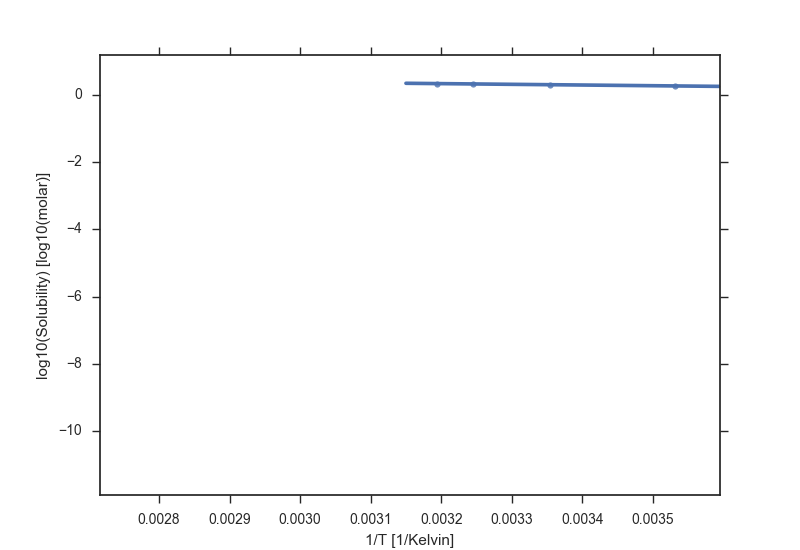

Supplement: Supplementary file 3 — Additional file 3. Additional results files, in electronic format. These additional results are (a) SUB-48 calculated lattice energies for the complete set and filtered set of 27 crystal structures, (b) results from analysis of the correspondence between our temperature dependent solubility data and (1/T), (c) Excel workbooks documenting all R2 and RMSE values obtained from cross-validation, their mean values and the p values (both raw and adjusted) obtained from pairwise comparisons of corresponding models, (d) comparison of melting point data used for the melting point descriptor and retrieved from the CSD for linked refcodes. [file 13321_2018_298_MOESM3_ESM.zip › additional_results/vantHoff_assessment/vh_plots/Klimenko_QSPRin_CD_False_MD_IntegSub.SiRMSSub.Absolv.Ind.Rdk_readyForR_vh.csv_13.tiff]

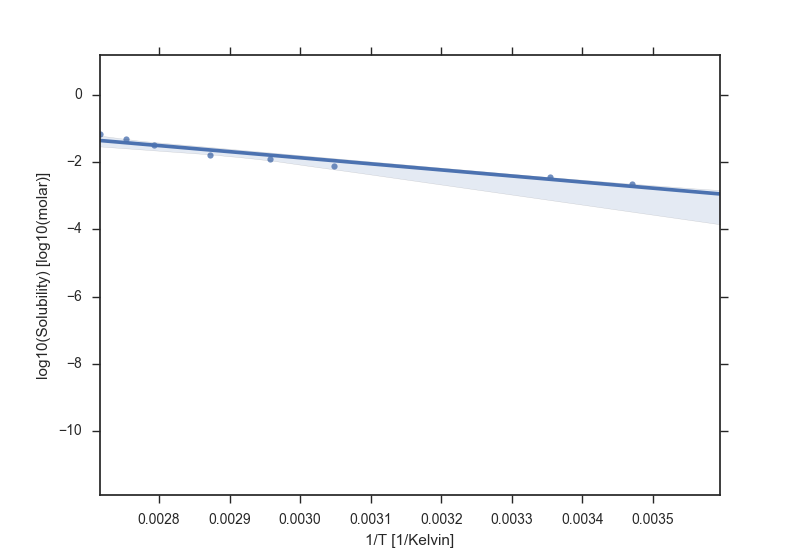

Supplement: Supplementary file 3 — Additional file 3. Additional results files, in electronic format. These additional results are (a) SUB-48 calculated lattice energies for the complete set and filtered set of 27 crystal structures, (b) results from analysis of the correspondence between our temperature dependent solubility data and (1/T), (c) Excel workbooks documenting all R2 and RMSE values obtained from cross-validation, their mean values and the p values (both raw and adjusted) obtained from pairwise comparisons of corresponding models, (d) comparison of melting point data used for the melting point descriptor and retrieved from the CSD for linked refcodes. [file 13321_2018_298_MOESM3_ESM.zip › additional_results/vantHoff_assessment/vh_plots/Klimenko_QSPRin_CD_False_MD_IntegSub.SiRMSSub.Absolv.Ind.Rdk_readyForR_vh.csv_130.tiff]

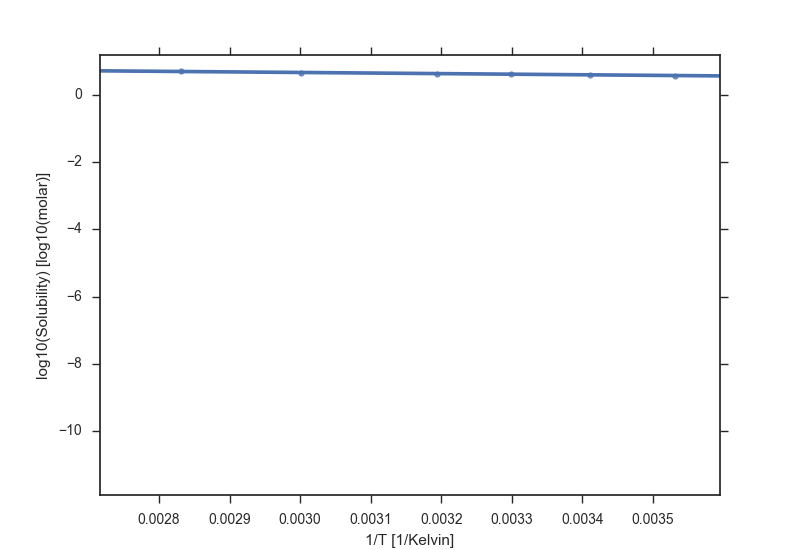

Supplement: Supplementary file 3 — Additional file 3. Additional results files, in electronic format. These additional results are (a) SUB-48 calculated lattice energies for the complete set and filtered set of 27 crystal structures, (b) results from analysis of the correspondence between our temperature dependent solubility data and (1/T), (c) Excel workbooks documenting all R2 and RMSE values obtained from cross-validation, their mean values and the p values (both raw and adjusted) obtained from pairwise comparisons of corresponding models, (d) comparison of melting point data used for the melting point descriptor and retrieved from the CSD for linked refcodes. [file 13321_2018_298_MOESM3_ESM.zip › additional_results/vantHoff_assessment/vh_plots/Klimenko_QSPRin_CD_False_MD_IntegSub.SiRMSSub.Absolv.Ind.Rdk_readyForR_vh.csv_133.tiff]

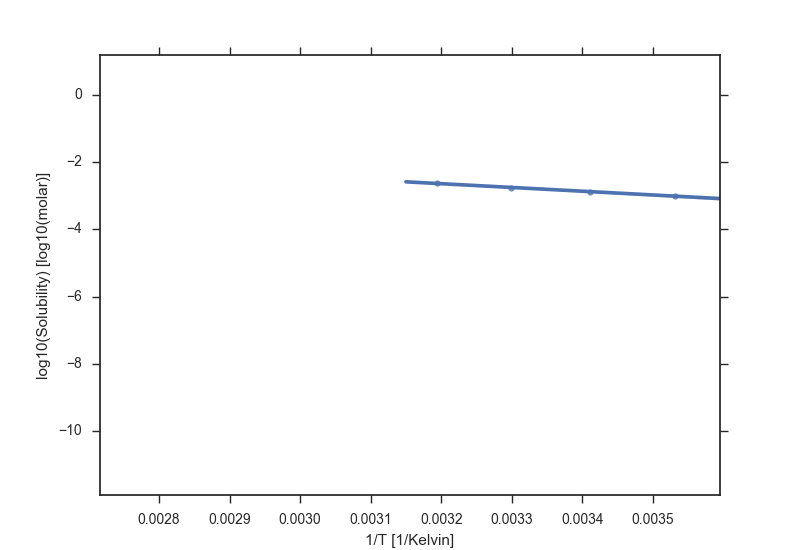

Supplement: Supplementary file 3 — Additional file 3. Additional results files, in electronic format. These additional results are (a) SUB-48 calculated lattice energies for the complete set and filtered set of 27 crystal structures, (b) results from analysis of the correspondence between our temperature dependent solubility data and (1/T), (c) Excel workbooks documenting all R2 and RMSE values obtained from cross-validation, their mean values and the p values (both raw and adjusted) obtained from pairwise comparisons of corresponding models, (d) comparison of melting point data used for the melting point descriptor and retrieved from the CSD for linked refcodes. [file 13321_2018_298_MOESM3_ESM.zip › additional_results/vantHoff_assessment/vh_plots/Klimenko_QSPRin_CD_False_MD_IntegSub.SiRMSSub.Absolv.Ind.Rdk_readyForR_vh.csv_140.tiff]

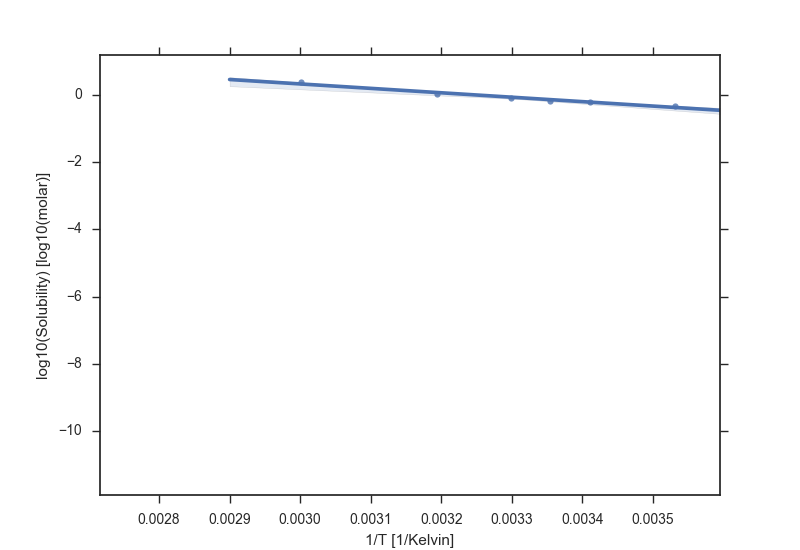

Supplement: Supplementary file 3 — Additional file 3. Additional results files, in electronic format. These additional results are (a) SUB-48 calculated lattice energies for the complete set and filtered set of 27 crystal structures, (b) results from analysis of the correspondence between our temperature dependent solubility data and (1/T), (c) Excel workbooks documenting all R2 and RMSE values obtained from cross-validation, their mean values and the p values (both raw and adjusted) obtained from pairwise comparisons of corresponding models, (d) comparison of melting point data used for the melting point descriptor and retrieved from the CSD for linked refcodes. [file 13321_2018_298_MOESM3_ESM.zip › additional_results/vantHoff_assessment/vh_plots/Klimenko_QSPRin_CD_False_MD_IntegSub.SiRMSSub.Absolv.Ind.Rdk_readyForR_vh.csv_141.tiff]

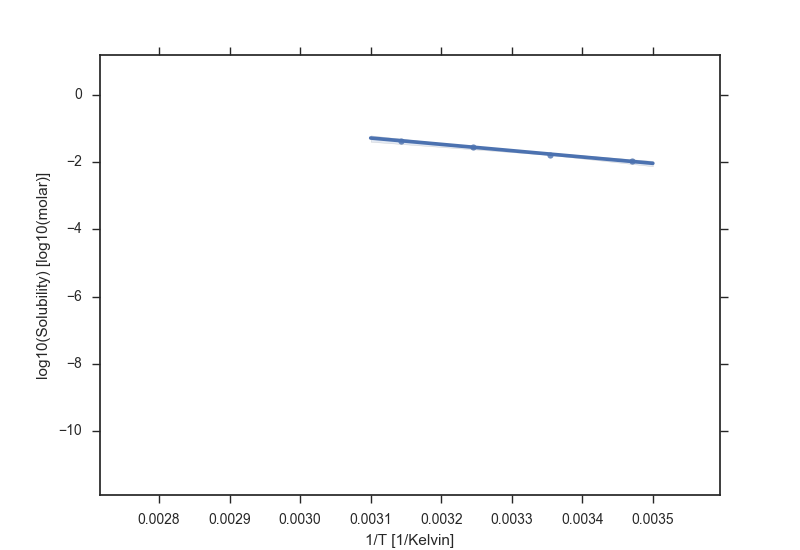

Supplement: Supplementary file 3 — Additional file 3. Additional results files, in electronic format. These additional results are (a) SUB-48 calculated lattice energies for the complete set and filtered set of 27 crystal structures, (b) results from analysis of the correspondence between our temperature dependent solubility data and (1/T), (c) Excel workbooks documenting all R2 and RMSE values obtained from cross-validation, their mean values and the p values (both raw and adjusted) obtained from pairwise comparisons of corresponding models, (d) comparison of melting point data used for the melting point descriptor and retrieved from the CSD for linked refcodes. [file 13321_2018_298_MOESM3_ESM.zip › additional_results/vantHoff_assessment/vh_plots/Klimenko_QSPRin_CD_False_MD_IntegSub.SiRMSSub.Absolv.Ind.Rdk_readyForR_vh.csv_142.tiff]

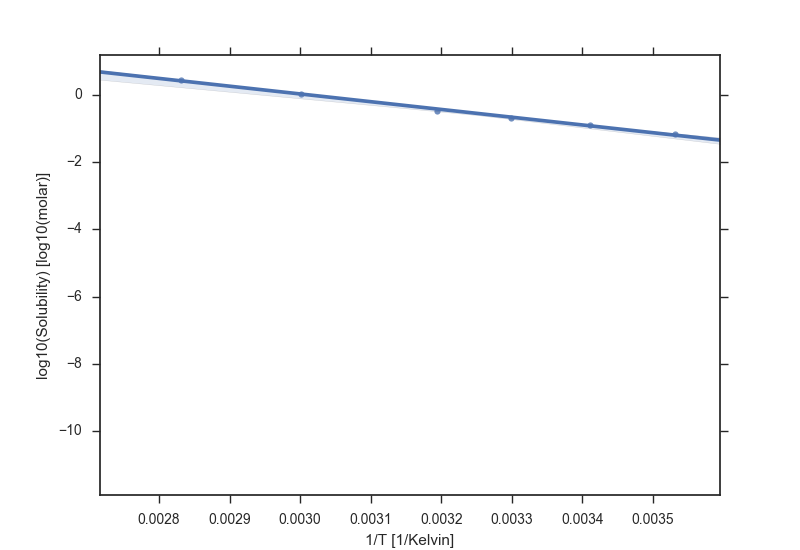

Supplement: Supplementary file 3 — Additional file 3. Additional results files, in electronic format. These additional results are (a) SUB-48 calculated lattice energies for the complete set and filtered set of 27 crystal structures, (b) results from analysis of the correspondence between our temperature dependent solubility data and (1/T), (c) Excel workbooks documenting all R2 and RMSE values obtained from cross-validation, their mean values and the p values (both raw and adjusted) obtained from pairwise comparisons of corresponding models, (d) comparison of melting point data used for the melting point descriptor and retrieved from the CSD for linked refcodes. [file 13321_2018_298_MOESM3_ESM.zip › additional_results/vantHoff_assessment/vh_plots/Klimenko_QSPRin_CD_False_MD_IntegSub.SiRMSSub.Absolv.Ind.Rdk_readyForR_vh.csv_143.tiff]

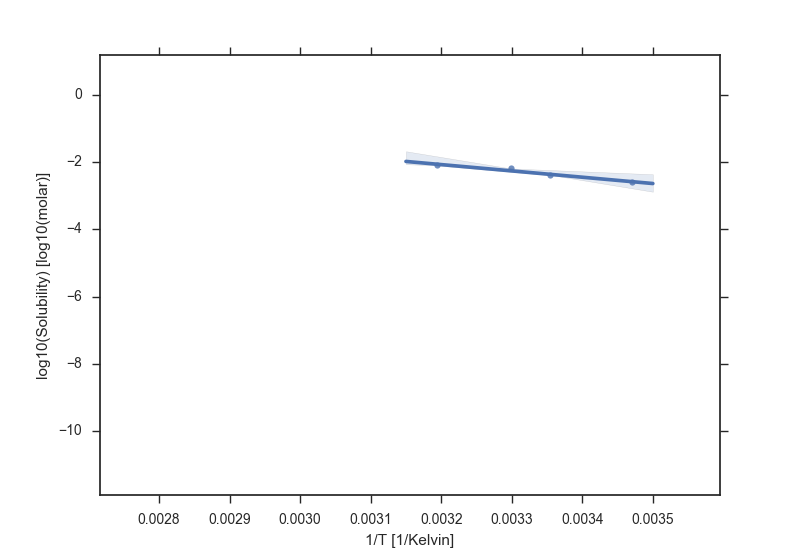

Supplement: Supplementary file 3 — Additional file 3. Additional results files, in electronic format. These additional results are (a) SUB-48 calculated lattice energies for the complete set and filtered set of 27 crystal structures, (b) results from analysis of the correspondence between our temperature dependent solubility data and (1/T), (c) Excel workbooks documenting all R2 and RMSE values obtained from cross-validation, their mean values and the p values (both raw and adjusted) obtained from pairwise comparisons of corresponding models, (d) comparison of melting point data used for the melting point descriptor and retrieved from the CSD for linked refcodes. [file 13321_2018_298_MOESM3_ESM.zip › additional_results/vantHoff_assessment/vh_plots/Klimenko_QSPRin_CD_False_MD_IntegSub.SiRMSSub.Absolv.Ind.Rdk_readyForR_vh.csv_145.tiff]

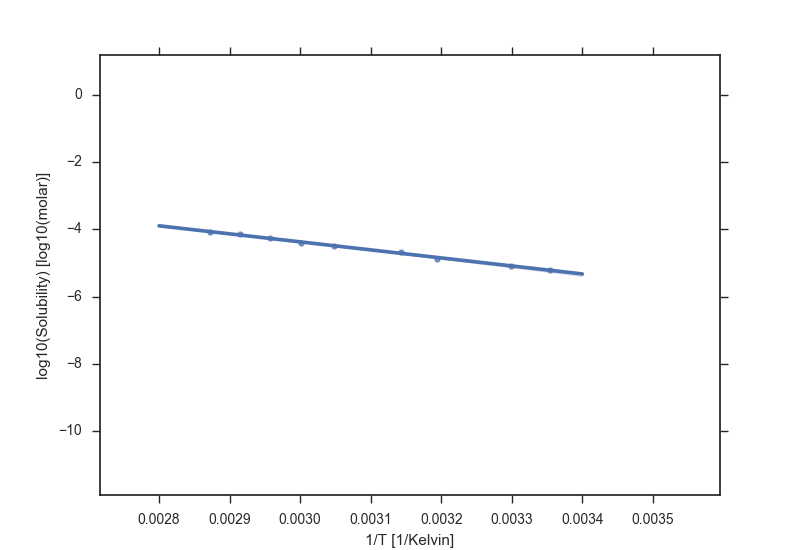

Supplement: Supplementary file 3 — Additional file 3. Additional results files, in electronic format. These additional results are (a) SUB-48 calculated lattice energies for the complete set and filtered set of 27 crystal structures, (b) results from analysis of the correspondence between our temperature dependent solubility data and (1/T), (c) Excel workbooks documenting all R2 and RMSE values obtained from cross-validation, their mean values and the p values (both raw and adjusted) obtained from pairwise comparisons of corresponding models, (d) comparison of melting point data used for the melting point descriptor and retrieved from the CSD for linked refcodes. [file 13321_2018_298_MOESM3_ESM.zip › additional_results/vantHoff_assessment/vh_plots/Klimenko_QSPRin_CD_False_MD_IntegSub.SiRMSSub.Absolv.Ind.Rdk_readyForR_vh.csv_148.tiff]

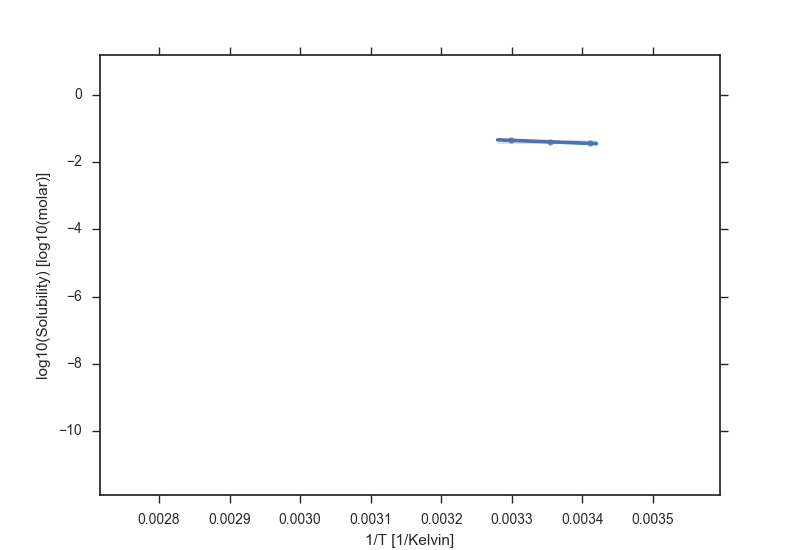

Supplement: Supplementary file 3 — Additional file 3. Additional results files, in electronic format. These additional results are (a) SUB-48 calculated lattice energies for the complete set and filtered set of 27 crystal structures, (b) results from analysis of the correspondence between our temperature dependent solubility data and (1/T), (c) Excel workbooks documenting all R2 and RMSE values obtained from cross-validation, their mean values and the p values (both raw and adjusted) obtained from pairwise comparisons of corresponding models, (d) comparison of melting point data used for the melting point descriptor and retrieved from the CSD for linked refcodes. [file 13321_2018_298_MOESM3_ESM.zip › additional_results/vantHoff_assessment/vh_plots/Klimenko_QSPRin_CD_False_MD_IntegSub.SiRMSSub.Absolv.Ind.Rdk_readyForR_vh.csv_149.tiff]

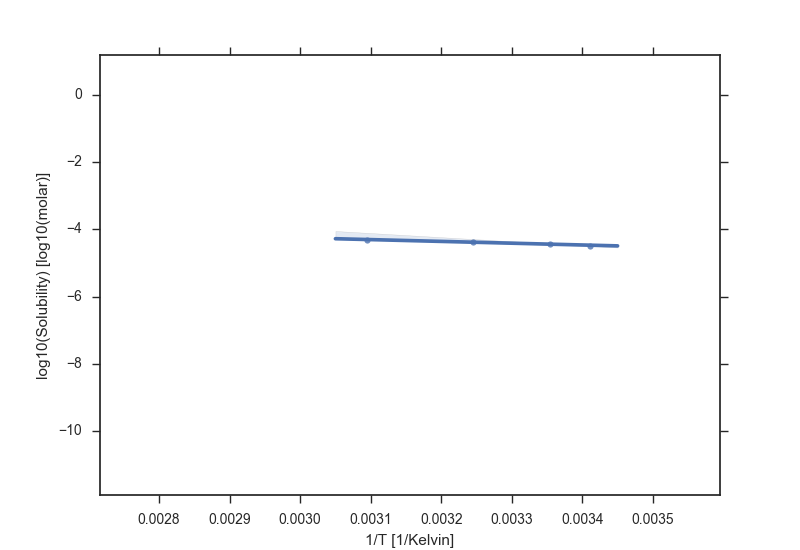

Supplement: Supplementary file 3 — Additional file 3. Additional results files, in electronic format. These additional results are (a) SUB-48 calculated lattice energies for the complete set and filtered set of 27 crystal structures, (b) results from analysis of the correspondence between our temperature dependent solubility data and (1/T), (c) Excel workbooks documenting all R2 and RMSE values obtained from cross-validation, their mean values and the p values (both raw and adjusted) obtained from pairwise comparisons of corresponding models, (d) comparison of melting point data used for the melting point descriptor and retrieved from the CSD for linked refcodes. [file 13321_2018_298_MOESM3_ESM.zip › additional_results/vantHoff_assessment/vh_plots/Klimenko_QSPRin_CD_False_MD_IntegSub.SiRMSSub.Absolv.Ind.Rdk_readyForR_vh.csv_151.tiff]

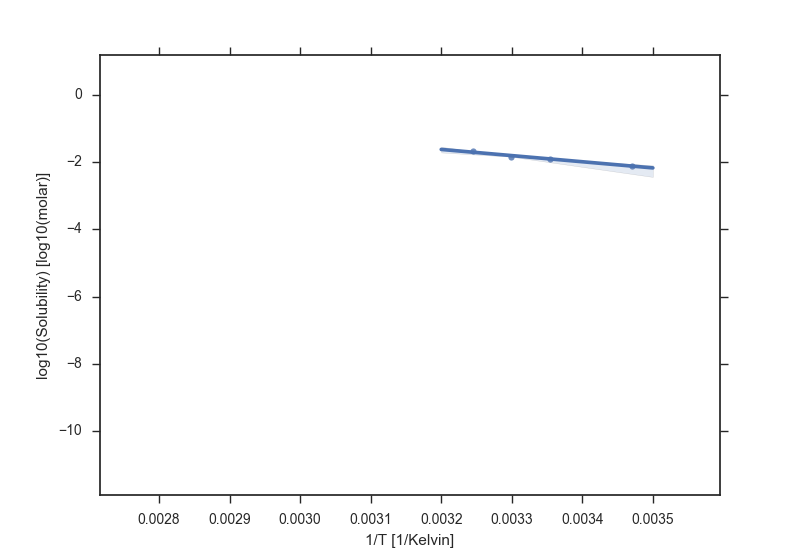

Supplement: Supplementary file 3 — Additional file 3. Additional results files, in electronic format. These additional results are (a) SUB-48 calculated lattice energies for the complete set and filtered set of 27 crystal structures, (b) results from analysis of the correspondence between our temperature dependent solubility data and (1/T), (c) Excel workbooks documenting all R2 and RMSE values obtained from cross-validation, their mean values and the p values (both raw and adjusted) obtained from pairwise comparisons of corresponding models, (d) comparison of melting point data used for the melting point descriptor and retrieved from the CSD for linked refcodes. [file 13321_2018_298_MOESM3_ESM.zip › additional_results/vantHoff_assessment/vh_plots/Klimenko_QSPRin_CD_False_MD_IntegSub.SiRMSSub.Absolv.Ind.Rdk_readyForR_vh.csv_153.tiff]

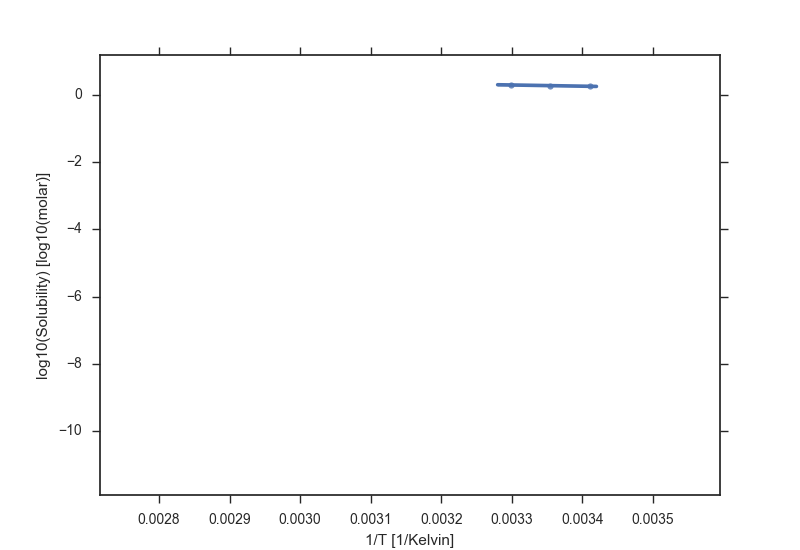

Supplement: Supplementary file 3 — Additional file 3. Additional results files, in electronic format. These additional results are (a) SUB-48 calculated lattice energies for the complete set and filtered set of 27 crystal structures, (b) results from analysis of the correspondence between our temperature dependent solubility data and (1/T), (c) Excel workbooks documenting all R2 and RMSE values obtained from cross-validation, their mean values and the p values (both raw and adjusted) obtained from pairwise comparisons of corresponding models, (d) comparison of melting point data used for the melting point descriptor and retrieved from the CSD for linked refcodes. [file 13321_2018_298_MOESM3_ESM.zip › additional_results/vantHoff_assessment/vh_plots/Klimenko_QSPRin_CD_False_MD_IntegSub.SiRMSSub.Absolv.Ind.Rdk_readyForR_vh.csv_155.tiff]

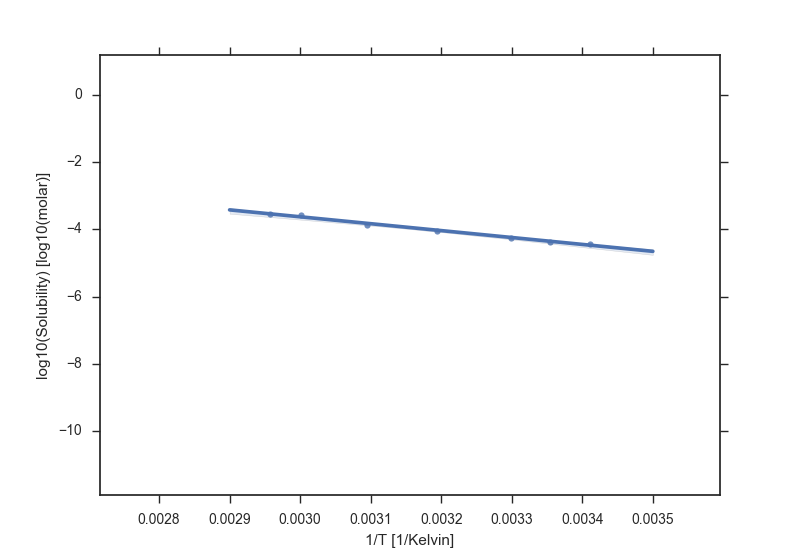

Supplement: Supplementary file 3 — Additional file 3. Additional results files, in electronic format. These additional results are (a) SUB-48 calculated lattice energies for the complete set and filtered set of 27 crystal structures, (b) results from analysis of the correspondence between our temperature dependent solubility data and (1/T), (c) Excel workbooks documenting all R2 and RMSE values obtained from cross-validation, their mean values and the p values (both raw and adjusted) obtained from pairwise comparisons of corresponding models, (d) comparison of melting point data used for the melting point descriptor and retrieved from the CSD for linked refcodes. [file 13321_2018_298_MOESM3_ESM.zip › additional_results/vantHoff_assessment/vh_plots/Klimenko_QSPRin_CD_False_MD_IntegSub.SiRMSSub.Absolv.Ind.Rdk_readyForR_vh.csv_156.tiff]

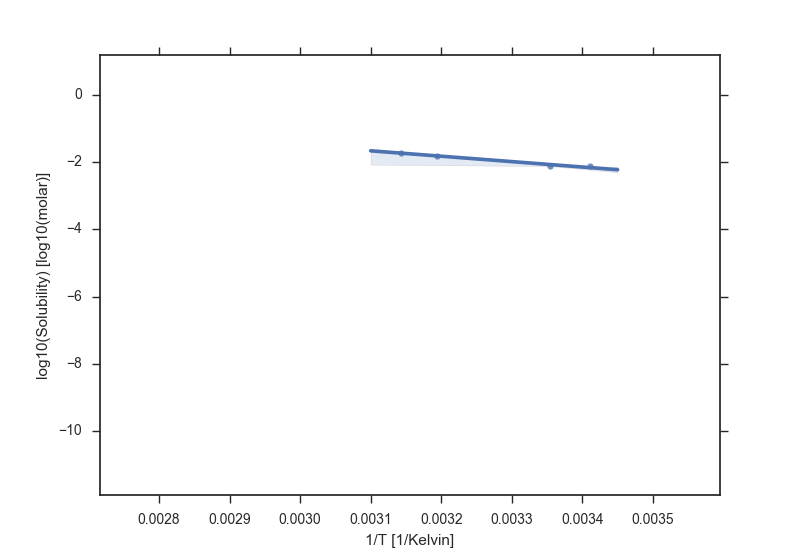

Supplement: Supplementary file 3 — Additional file 3. Additional results files, in electronic format. These additional results are (a) SUB-48 calculated lattice energies for the complete set and filtered set of 27 crystal structures, (b) results from analysis of the correspondence between our temperature dependent solubility data and (1/T), (c) Excel workbooks documenting all R2 and RMSE values obtained from cross-validation, their mean values and the p values (both raw and adjusted) obtained from pairwise comparisons of corresponding models, (d) comparison of melting point data used for the melting point descriptor and retrieved from the CSD for linked refcodes. [file 13321_2018_298_MOESM3_ESM.zip › additional_results/vantHoff_assessment/vh_plots/Klimenko_QSPRin_CD_False_MD_IntegSub.SiRMSSub.Absolv.Ind.Rdk_readyForR_vh.csv_158.tiff]

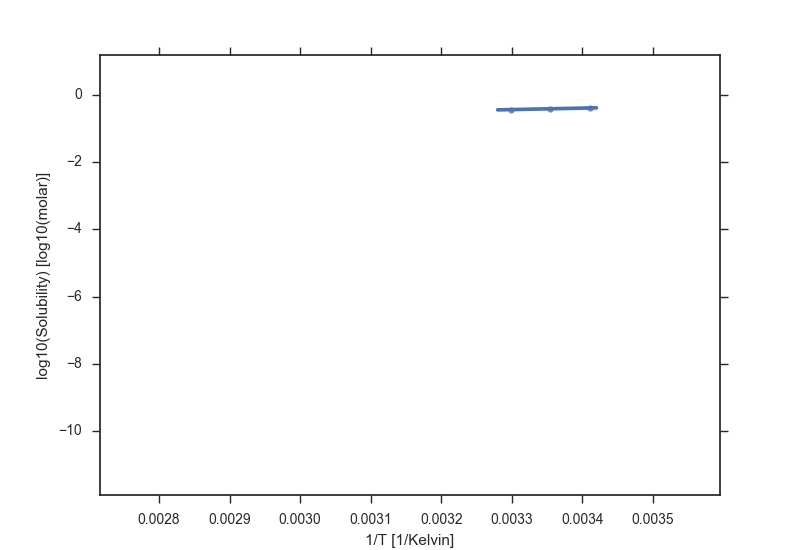

Supplement: Supplementary file 3 — Additional file 3. Additional results files, in electronic format. These additional results are (a) SUB-48 calculated lattice energies for the complete set and filtered set of 27 crystal structures, (b) results from analysis of the correspondence between our temperature dependent solubility data and (1/T), (c) Excel workbooks documenting all R2 and RMSE values obtained from cross-validation, their mean values and the p values (both raw and adjusted) obtained from pairwise comparisons of corresponding models, (d) comparison of melting point data used for the melting point descriptor and retrieved from the CSD for linked refcodes. [file 13321_2018_298_MOESM3_ESM.zip › additional_results/vantHoff_assessment/vh_plots/Klimenko_QSPRin_CD_False_MD_IntegSub.SiRMSSub.Absolv.Ind.Rdk_readyForR_vh.csv_162.tiff]

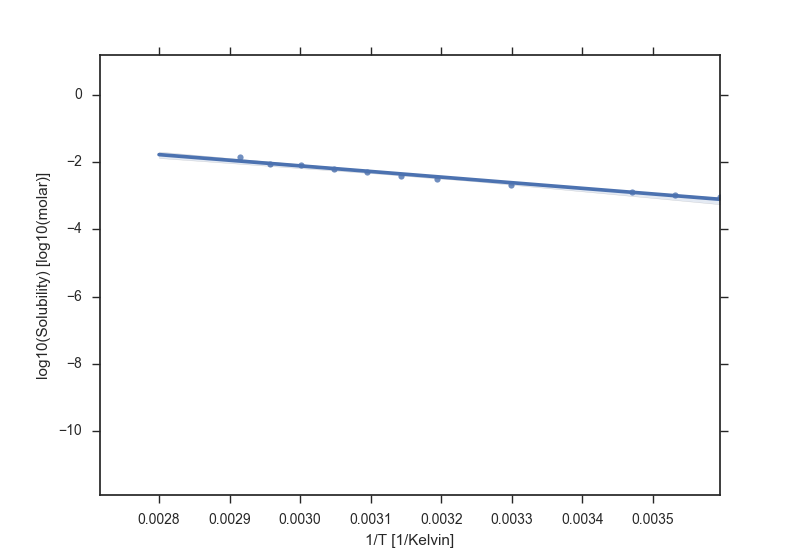

Supplement: Supplementary file 3 — Additional file 3. Additional results files, in electronic format. These additional results are (a) SUB-48 calculated lattice energies for the complete set and filtered set of 27 crystal structures, (b) results from analysis of the correspondence between our temperature dependent solubility data and (1/T), (c) Excel workbooks documenting all R2 and RMSE values obtained from cross-validation, their mean values and the p values (both raw and adjusted) obtained from pairwise comparisons of corresponding models, (d) comparison of melting point data used for the melting point descriptor and retrieved from the CSD for linked refcodes. [file 13321_2018_298_MOESM3_ESM.zip › additional_results/vantHoff_assessment/vh_plots/Klimenko_QSPRin_CD_False_MD_IntegSub.SiRMSSub.Absolv.Ind.Rdk_readyForR_vh.csv_163.tiff]

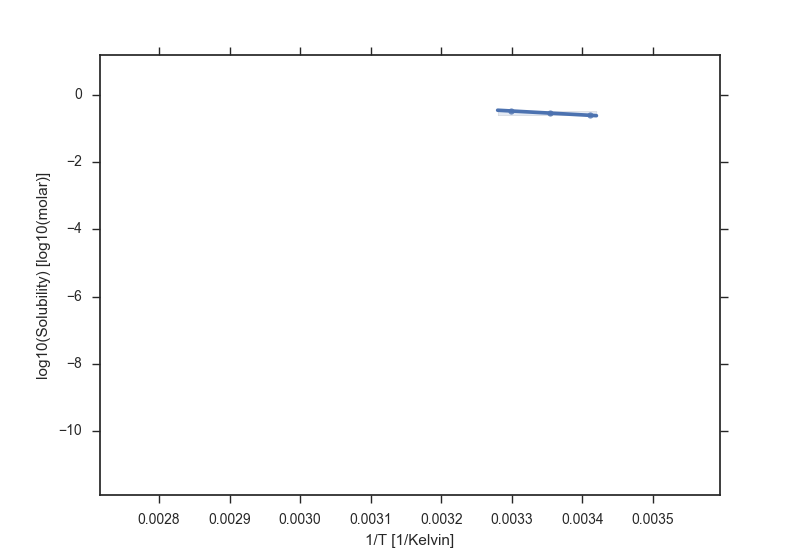

Supplement: Supplementary file 3 — Additional file 3. Additional results files, in electronic format. These additional results are (a) SUB-48 calculated lattice energies for the complete set and filtered set of 27 crystal structures, (b) results from analysis of the correspondence between our temperature dependent solubility data and (1/T), (c) Excel workbooks documenting all R2 and RMSE values obtained from cross-validation, their mean values and the p values (both raw and adjusted) obtained from pairwise comparisons of corresponding models, (d) comparison of melting point data used for the melting point descriptor and retrieved from the CSD for linked refcodes. [file 13321_2018_298_MOESM3_ESM.zip › additional_results/vantHoff_assessment/vh_plots/Klimenko_QSPRin_CD_False_MD_IntegSub.SiRMSSub.Absolv.Ind.Rdk_readyForR_vh.csv_166.tiff]

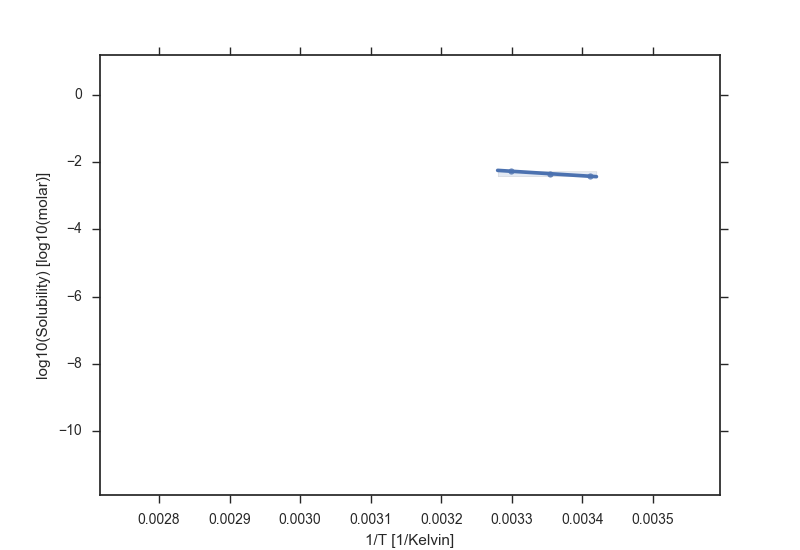

Supplement: Supplementary file 3 — Additional file 3. Additional results files, in electronic format. These additional results are (a) SUB-48 calculated lattice energies for the complete set and filtered set of 27 crystal structures, (b) results from analysis of the correspondence between our temperature dependent solubility data and (1/T), (c) Excel workbooks documenting all R2 and RMSE values obtained from cross-validation, their mean values and the p values (both raw and adjusted) obtained from pairwise comparisons of corresponding models, (d) comparison of melting point data used for the melting point descriptor and retrieved from the CSD for linked refcodes. [file 13321_2018_298_MOESM3_ESM.zip › additional_results/vantHoff_assessment/vh_plots/Klimenko_QSPRin_CD_False_MD_IntegSub.SiRMSSub.Absolv.Ind.Rdk_readyForR_vh.csv_168.tiff]

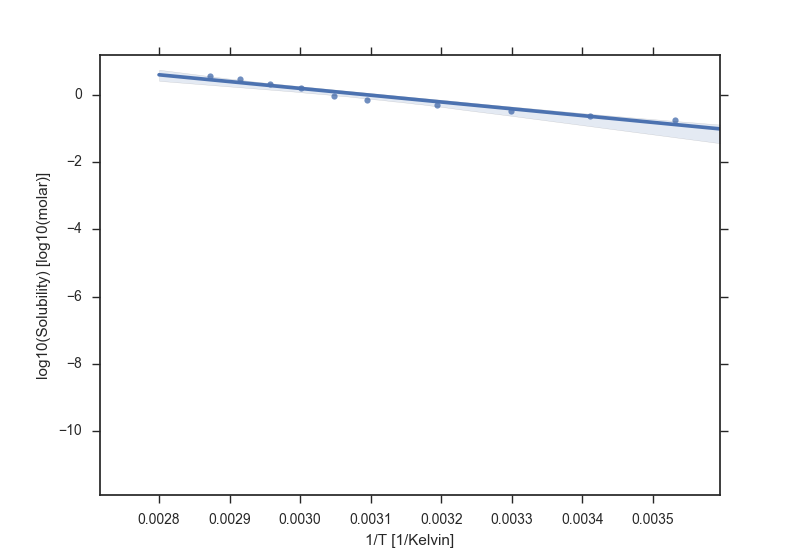

Supplement: Supplementary file 3 — Additional file 3. Additional results files, in electronic format. These additional results are (a) SUB-48 calculated lattice energies for the complete set and filtered set of 27 crystal structures, (b) results from analysis of the correspondence between our temperature dependent solubility data and (1/T), (c) Excel workbooks documenting all R2 and RMSE values obtained from cross-validation, their mean values and the p values (both raw and adjusted) obtained from pairwise comparisons of corresponding models, (d) comparison of melting point data used for the melting point descriptor and retrieved from the CSD for linked refcodes. [file 13321_2018_298_MOESM3_ESM.zip › additional_results/vantHoff_assessment/vh_plots/Klimenko_QSPRin_CD_False_MD_IntegSub.SiRMSSub.Absolv.Ind.Rdk_readyForR_vh.csv_169.tiff]

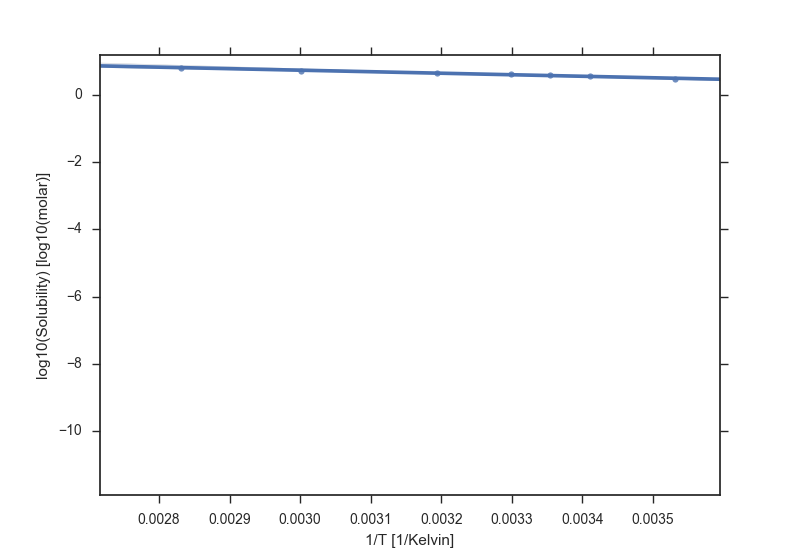

Supplement: Supplementary file 3 — Additional file 3. Additional results files, in electronic format. These additional results are (a) SUB-48 calculated lattice energies for the complete set and filtered set of 27 crystal structures, (b) results from analysis of the correspondence between our temperature dependent solubility data and (1/T), (c) Excel workbooks documenting all R2 and RMSE values obtained from cross-validation, their mean values and the p values (both raw and adjusted) obtained from pairwise comparisons of corresponding models, (d) comparison of melting point data used for the melting point descriptor and retrieved from the CSD for linked refcodes. [file 13321_2018_298_MOESM3_ESM.zip › additional_results/vantHoff_assessment/vh_plots/Klimenko_QSPRin_CD_False_MD_IntegSub.SiRMSSub.Absolv.Ind.Rdk_readyForR_vh.csv_17.tiff]

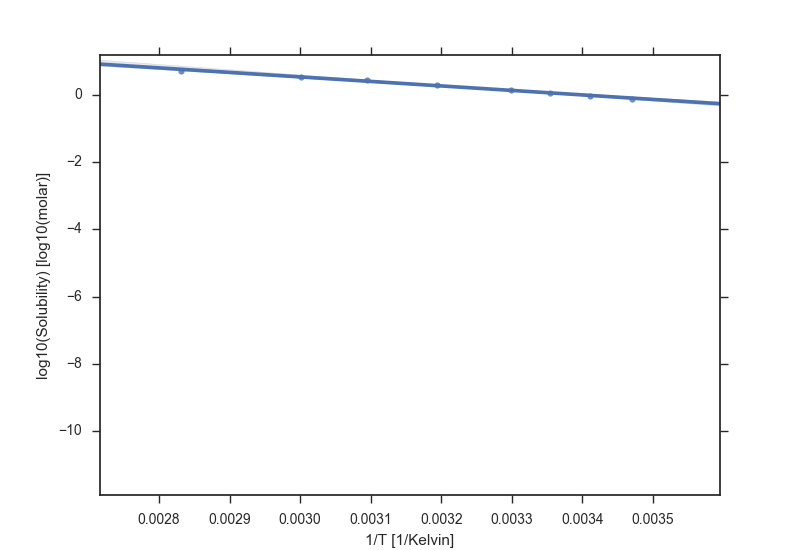

Supplement: Supplementary file 3 — Additional file 3. Additional results files, in electronic format. These additional results are (a) SUB-48 calculated lattice energies for the complete set and filtered set of 27 crystal structures, (b) results from analysis of the correspondence between our temperature dependent solubility data and (1/T), (c) Excel workbooks documenting all R2 and RMSE values obtained from cross-validation, their mean values and the p values (both raw and adjusted) obtained from pairwise comparisons of corresponding models, (d) comparison of melting point data used for the melting point descriptor and retrieved from the CSD for linked refcodes. [file 13321_2018_298_MOESM3_ESM.zip › additional_results/vantHoff_assessment/vh_plots/Klimenko_QSPRin_CD_False_MD_IntegSub.SiRMSSub.Absolv.Ind.Rdk_readyForR_vh.csv_170.tiff]

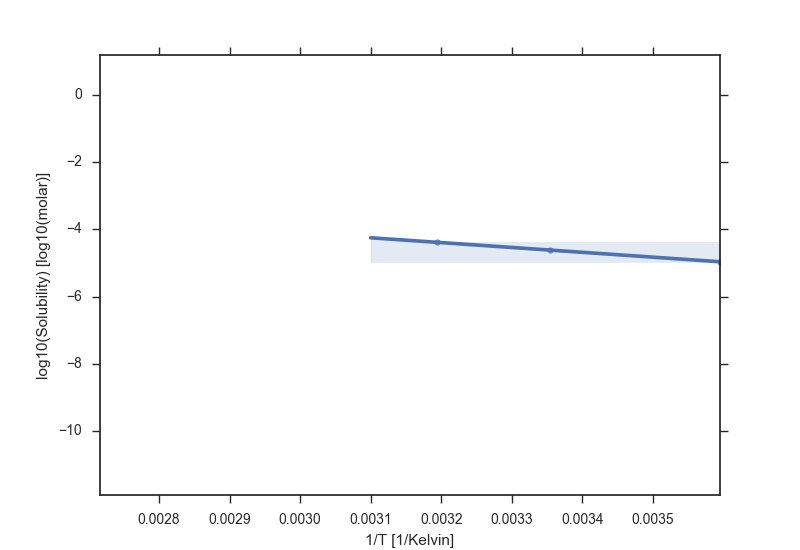

Supplement: Supplementary file 3 — Additional file 3. Additional results files, in electronic format. These additional results are (a) SUB-48 calculated lattice energies for the complete set and filtered set of 27 crystal structures, (b) results from analysis of the correspondence between our temperature dependent solubility data and (1/T), (c) Excel workbooks documenting all R2 and RMSE values obtained from cross-validation, their mean values and the p values (both raw and adjusted) obtained from pairwise comparisons of corresponding models, (d) comparison of melting point data used for the melting point descriptor and retrieved from the CSD for linked refcodes. [file 13321_2018_298_MOESM3_ESM.zip › additional_results/vantHoff_assessment/vh_plots/Klimenko_QSPRin_CD_False_MD_IntegSub.SiRMSSub.Absolv.Ind.Rdk_readyForR_vh.csv_174.tiff]

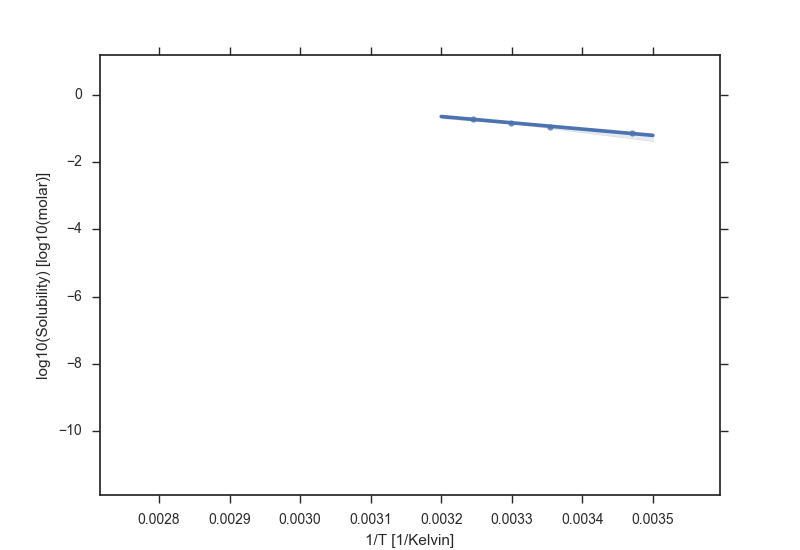

Supplement: Supplementary file 3 — Additional file 3. Additional results files, in electronic format. These additional results are (a) SUB-48 calculated lattice energies for the complete set and filtered set of 27 crystal structures, (b) results from analysis of the correspondence between our temperature dependent solubility data and (1/T), (c) Excel workbooks documenting all R2 and RMSE values obtained from cross-validation, their mean values and the p values (both raw and adjusted) obtained from pairwise comparisons of corresponding models, (d) comparison of melting point data used for the melting point descriptor and retrieved from the CSD for linked refcodes. [file 13321_2018_298_MOESM3_ESM.zip › additional_results/vantHoff_assessment/vh_plots/Klimenko_QSPRin_CD_False_MD_IntegSub.SiRMSSub.Absolv.Ind.Rdk_readyForR_vh.csv_178.tiff]

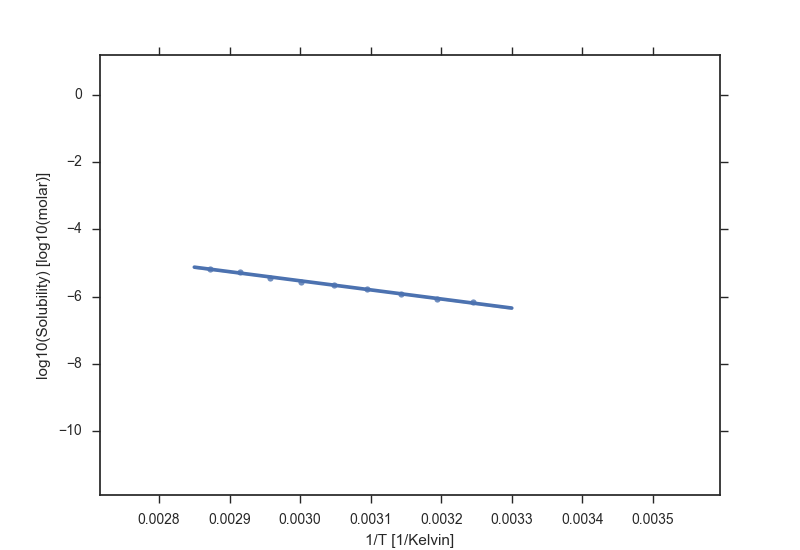

Supplement: Supplementary file 3 — Additional file 3. Additional results files, in electronic format. These additional results are (a) SUB-48 calculated lattice energies for the complete set and filtered set of 27 crystal structures, (b) results from analysis of the correspondence between our temperature dependent solubility data and (1/T), (c) Excel workbooks documenting all R2 and RMSE values obtained from cross-validation, their mean values and the p values (both raw and adjusted) obtained from pairwise comparisons of corresponding models, (d) comparison of melting point data used for the melting point descriptor and retrieved from the CSD for linked refcodes. [file 13321_2018_298_MOESM3_ESM.zip › additional_results/vantHoff_assessment/vh_plots/Klimenko_QSPRin_CD_False_MD_IntegSub.SiRMSSub.Absolv.Ind.Rdk_readyForR_vh.csv_180.tiff]

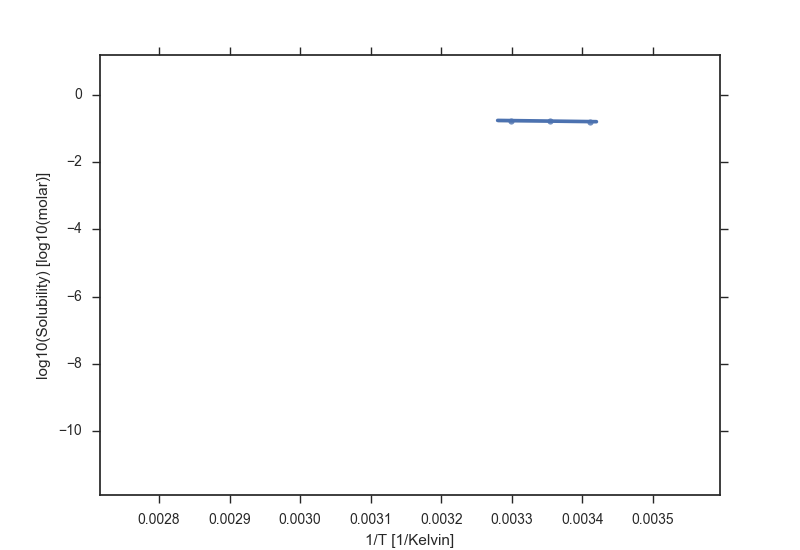

Supplement: Supplementary file 3 — Additional file 3. Additional results files, in electronic format. These additional results are (a) SUB-48 calculated lattice energies for the complete set and filtered set of 27 crystal structures, (b) results from analysis of the correspondence between our temperature dependent solubility data and (1/T), (c) Excel workbooks documenting all R2 and RMSE values obtained from cross-validation, their mean values and the p values (both raw and adjusted) obtained from pairwise comparisons of corresponding models, (d) comparison of melting point data used for the melting point descriptor and retrieved from the CSD for linked refcodes. [file 13321_2018_298_MOESM3_ESM.zip › additional_results/vantHoff_assessment/vh_plots/Klimenko_QSPRin_CD_False_MD_IntegSub.SiRMSSub.Absolv.Ind.Rdk_readyForR_vh.csv_181.tiff]

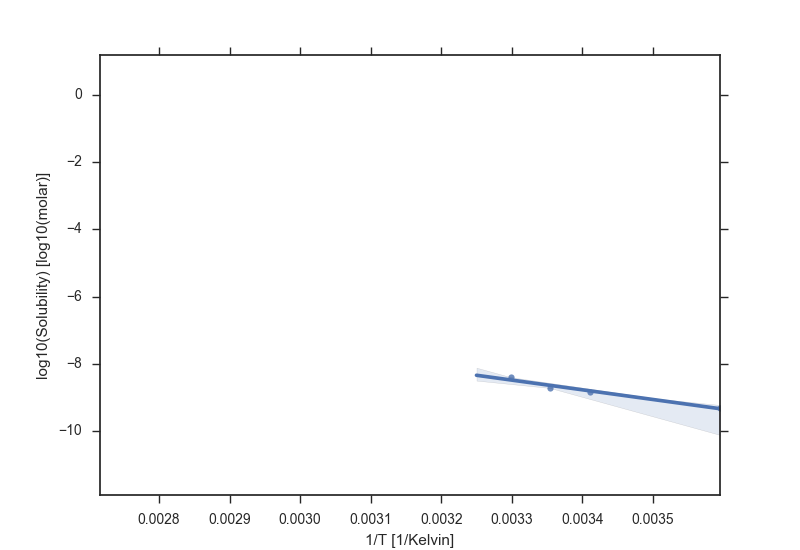

Supplement: Supplementary file 3 — Additional file 3. Additional results files, in electronic format. These additional results are (a) SUB-48 calculated lattice energies for the complete set and filtered set of 27 crystal structures, (b) results from analysis of the correspondence between our temperature dependent solubility data and (1/T), (c) Excel workbooks documenting all R2 and RMSE values obtained from cross-validation, their mean values and the p values (both raw and adjusted) obtained from pairwise comparisons of corresponding models, (d) comparison of melting point data used for the melting point descriptor and retrieved from the CSD for linked refcodes. [file 13321_2018_298_MOESM3_ESM.zip › additional_results/vantHoff_assessment/vh_plots/Klimenko_QSPRin_CD_False_MD_IntegSub.SiRMSSub.Absolv.Ind.Rdk_readyForR_vh.csv_182.tiff]

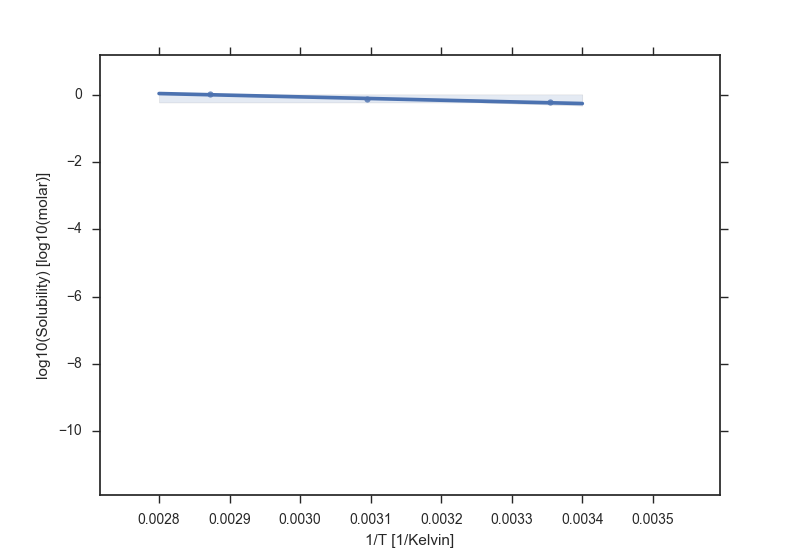

Supplement: Supplementary file 3 — Additional file 3. Additional results files, in electronic format. These additional results are (a) SUB-48 calculated lattice energies for the complete set and filtered set of 27 crystal structures, (b) results from analysis of the correspondence between our temperature dependent solubility data and (1/T), (c) Excel workbooks documenting all R2 and RMSE values obtained from cross-validation, their mean values and the p values (both raw and adjusted) obtained from pairwise comparisons of corresponding models, (d) comparison of melting point data used for the melting point descriptor and retrieved from the CSD for linked refcodes. [file 13321_2018_298_MOESM3_ESM.zip › additional_results/vantHoff_assessment/vh_plots/Klimenko_QSPRin_CD_False_MD_IntegSub.SiRMSSub.Absolv.Ind.Rdk_readyForR_vh.csv_184.tiff]

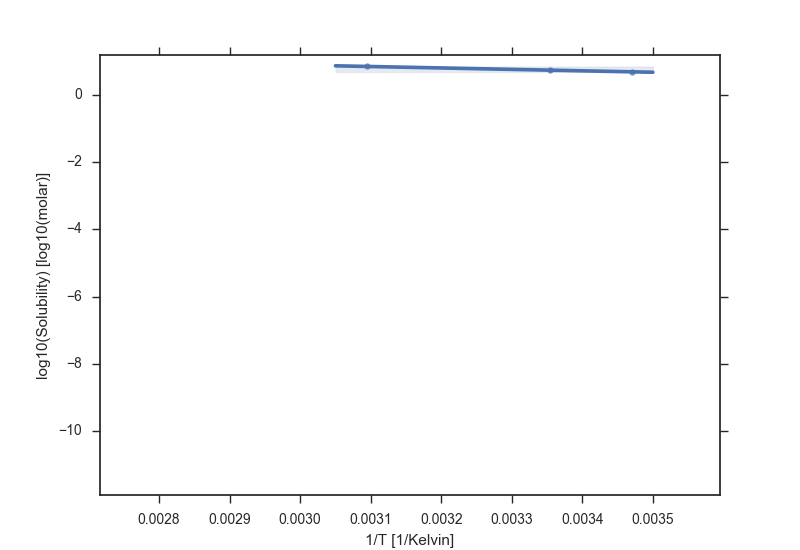

Supplement: Supplementary file 3 — Additional file 3. Additional results files, in electronic format. These additional results are (a) SUB-48 calculated lattice energies for the complete set and filtered set of 27 crystal structures, (b) results from analysis of the correspondence between our temperature dependent solubility data and (1/T), (c) Excel workbooks documenting all R2 and RMSE values obtained from cross-validation, their mean values and the p values (both raw and adjusted) obtained from pairwise comparisons of corresponding models, (d) comparison of melting point data used for the melting point descriptor and retrieved from the CSD for linked refcodes. [file 13321_2018_298_MOESM3_ESM.zip › additional_results/vantHoff_assessment/vh_plots/Klimenko_QSPRin_CD_False_MD_IntegSub.SiRMSSub.Absolv.Ind.Rdk_readyForR_vh.csv_186.tiff]

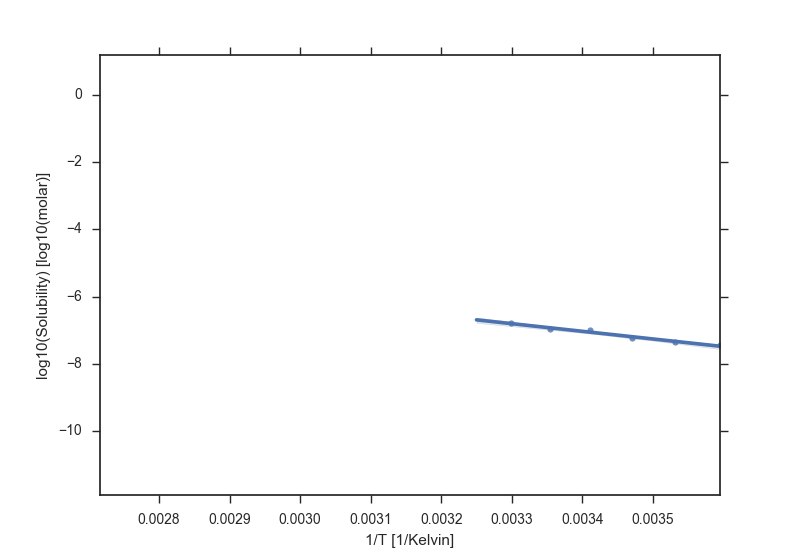

Supplement: Supplementary file 3 — Additional file 3. Additional results files, in electronic format. These additional results are (a) SUB-48 calculated lattice energies for the complete set and filtered set of 27 crystal structures, (b) results from analysis of the correspondence between our temperature dependent solubility data and (1/T), (c) Excel workbooks documenting all R2 and RMSE values obtained from cross-validation, their mean values and the p values (both raw and adjusted) obtained from pairwise comparisons of corresponding models, (d) comparison of melting point data used for the melting point descriptor and retrieved from the CSD for linked refcodes. [file 13321_2018_298_MOESM3_ESM.zip › additional_results/vantHoff_assessment/vh_plots/Klimenko_QSPRin_CD_False_MD_IntegSub.SiRMSSub.Absolv.Ind.Rdk_readyForR_vh.csv_189.tiff]

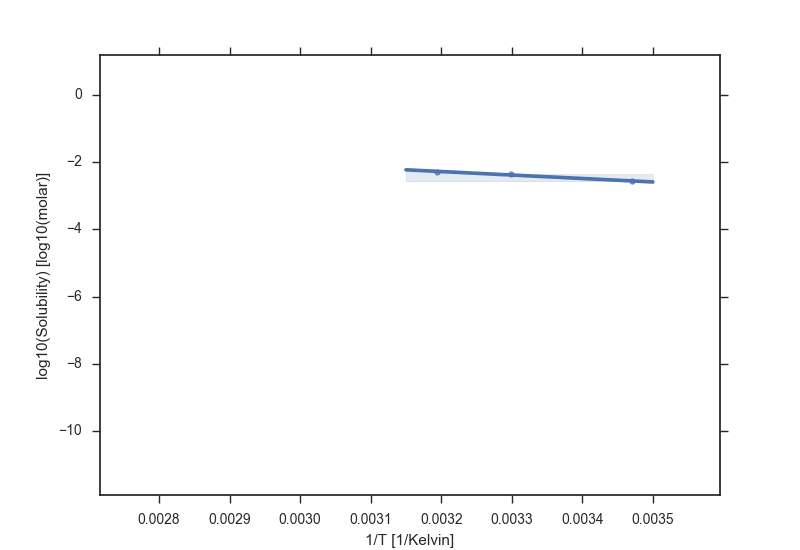

Supplement: Supplementary file 3 — Additional file 3. Additional results files, in electronic format. These additional results are (a) SUB-48 calculated lattice energies for the complete set and filtered set of 27 crystal structures, (b) results from analysis of the correspondence between our temperature dependent solubility data and (1/T), (c) Excel workbooks documenting all R2 and RMSE values obtained from cross-validation, their mean values and the p values (both raw and adjusted) obtained from pairwise comparisons of corresponding models, (d) comparison of melting point data used for the melting point descriptor and retrieved from the CSD for linked refcodes. [file 13321_2018_298_MOESM3_ESM.zip › additional_results/vantHoff_assessment/vh_plots/Klimenko_QSPRin_CD_False_MD_IntegSub.SiRMSSub.Absolv.Ind.Rdk_readyForR_vh.csv_191.tiff]

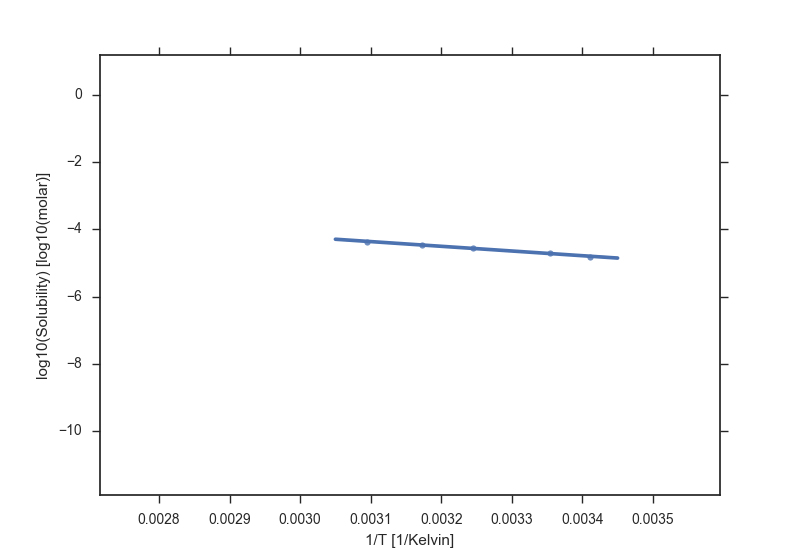

Supplement: Supplementary file 3 — Additional file 3. Additional results files, in electronic format. These additional results are (a) SUB-48 calculated lattice energies for the complete set and filtered set of 27 crystal structures, (b) results from analysis of the correspondence between our temperature dependent solubility data and (1/T), (c) Excel workbooks documenting all R2 and RMSE values obtained from cross-validation, their mean values and the p values (both raw and adjusted) obtained from pairwise comparisons of corresponding models, (d) comparison of melting point data used for the melting point descriptor and retrieved from the CSD for linked refcodes. [file 13321_2018_298_MOESM3_ESM.zip › additional_results/vantHoff_assessment/vh_plots/Klimenko_QSPRin_CD_False_MD_IntegSub.SiRMSSub.Absolv.Ind.Rdk_readyForR_vh.csv_192.tiff]

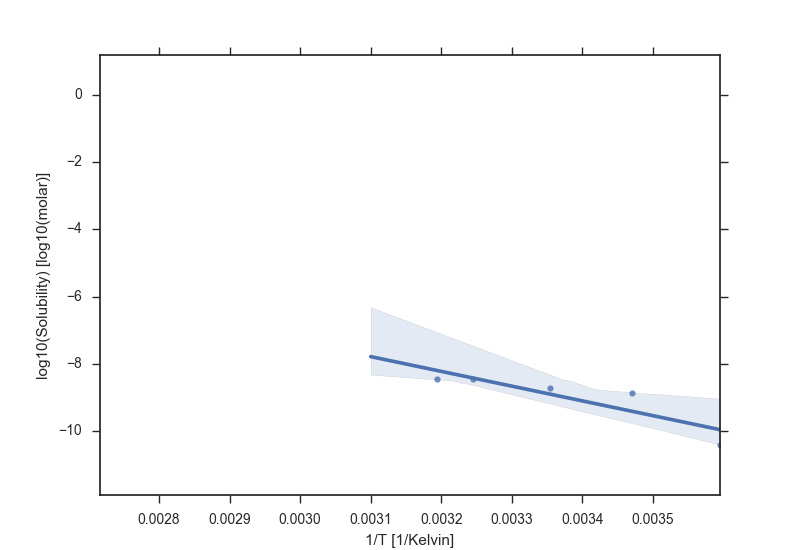

Supplement: Supplementary file 3 — Additional file 3. Additional results files, in electronic format. These additional results are (a) SUB-48 calculated lattice energies for the complete set and filtered set of 27 crystal structures, (b) results from analysis of the correspondence between our temperature dependent solubility data and (1/T), (c) Excel workbooks documenting all R2 and RMSE values obtained from cross-validation, their mean values and the p values (both raw and adjusted) obtained from pairwise comparisons of corresponding models, (d) comparison of melting point data used for the melting point descriptor and retrieved from the CSD for linked refcodes. [file 13321_2018_298_MOESM3_ESM.zip › additional_results/vantHoff_assessment/vh_plots/Klimenko_QSPRin_CD_False_MD_IntegSub.SiRMSSub.Absolv.Ind.Rdk_readyForR_vh.csv_194.tiff]

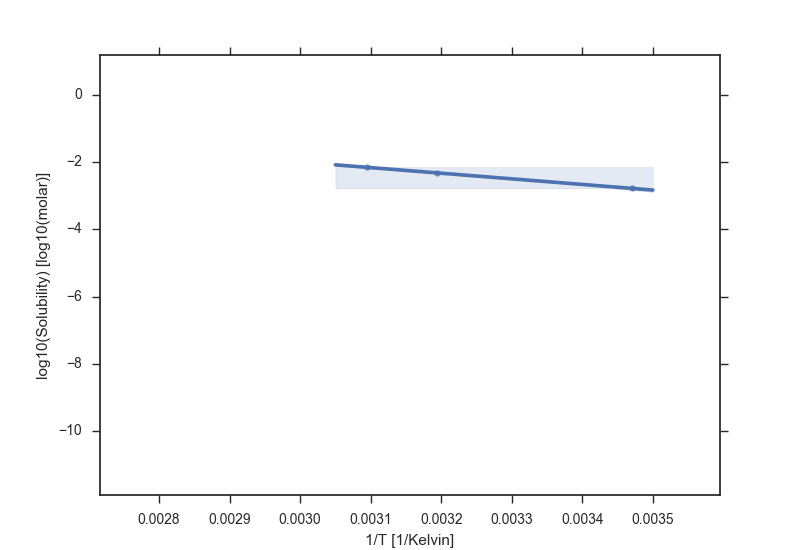

Supplement: Supplementary file 3 — Additional file 3. Additional results files, in electronic format. These additional results are (a) SUB-48 calculated lattice energies for the complete set and filtered set of 27 crystal structures, (b) results from analysis of the correspondence between our temperature dependent solubility data and (1/T), (c) Excel workbooks documenting all R2 and RMSE values obtained from cross-validation, their mean values and the p values (both raw and adjusted) obtained from pairwise comparisons of corresponding models, (d) comparison of melting point data used for the melting point descriptor and retrieved from the CSD for linked refcodes. [file 13321_2018_298_MOESM3_ESM.zip › additional_results/vantHoff_assessment/vh_plots/Klimenko_QSPRin_CD_False_MD_IntegSub.SiRMSSub.Absolv.Ind.Rdk_readyForR_vh.csv_201.tiff]

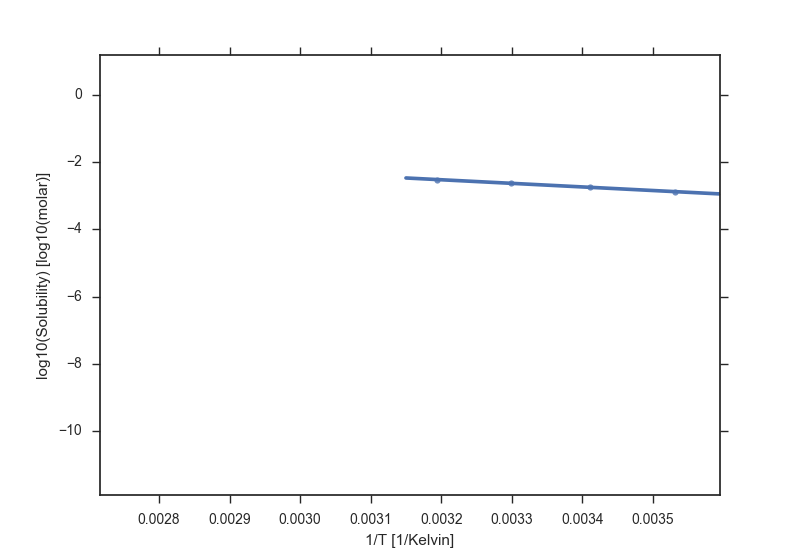

Supplement: Supplementary file 3 — Additional file 3. Additional results files, in electronic format. These additional results are (a) SUB-48 calculated lattice energies for the complete set and filtered set of 27 crystal structures, (b) results from analysis of the correspondence between our temperature dependent solubility data and (1/T), (c) Excel workbooks documenting all R2 and RMSE values obtained from cross-validation, their mean values and the p values (both raw and adjusted) obtained from pairwise comparisons of corresponding models, (d) comparison of melting point data used for the melting point descriptor and retrieved from the CSD for linked refcodes. [file 13321_2018_298_MOESM3_ESM.zip › additional_results/vantHoff_assessment/vh_plots/Klimenko_QSPRin_CD_False_MD_IntegSub.SiRMSSub.Absolv.Ind.Rdk_readyForR_vh.csv_202.tiff]

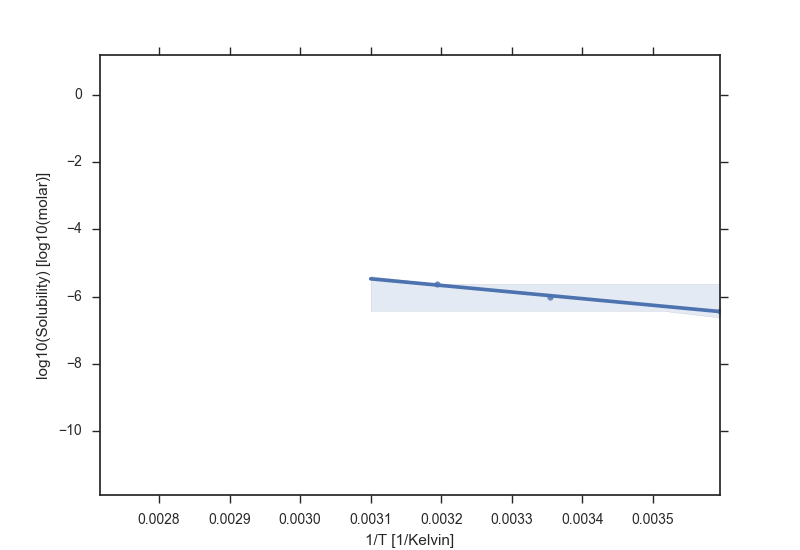

Supplement: Supplementary file 3 — Additional file 3. Additional results files, in electronic format. These additional results are (a) SUB-48 calculated lattice energies for the complete set and filtered set of 27 crystal structures, (b) results from analysis of the correspondence between our temperature dependent solubility data and (1/T), (c) Excel workbooks documenting all R2 and RMSE values obtained from cross-validation, their mean values and the p values (both raw and adjusted) obtained from pairwise comparisons of corresponding models, (d) comparison of melting point data used for the melting point descriptor and retrieved from the CSD for linked refcodes. [file 13321_2018_298_MOESM3_ESM.zip › additional_results/vantHoff_assessment/vh_plots/Klimenko_QSPRin_CD_False_MD_IntegSub.SiRMSSub.Absolv.Ind.Rdk_readyForR_vh.csv_206.tiff]

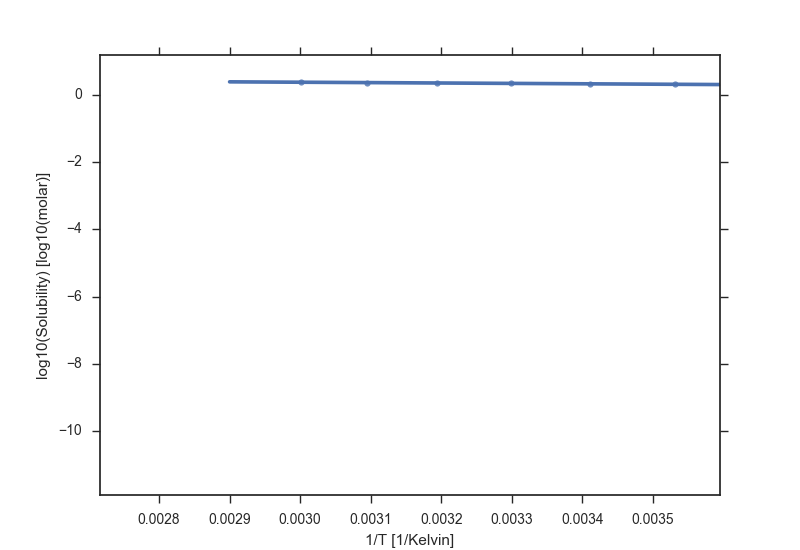

Supplement: Supplementary file 3 — Additional file 3. Additional results files, in electronic format. These additional results are (a) SUB-48 calculated lattice energies for the complete set and filtered set of 27 crystal structures, (b) results from analysis of the correspondence between our temperature dependent solubility data and (1/T), (c) Excel workbooks documenting all R2 and RMSE values obtained from cross-validation, their mean values and the p values (both raw and adjusted) obtained from pairwise comparisons of corresponding models, (d) comparison of melting point data used for the melting point descriptor and retrieved from the CSD for linked refcodes. [file 13321_2018_298_MOESM3_ESM.zip › additional_results/vantHoff_assessment/vh_plots/Klimenko_QSPRin_CD_False_MD_IntegSub.SiRMSSub.Absolv.Ind.Rdk_readyForR_vh.csv_207.tiff]

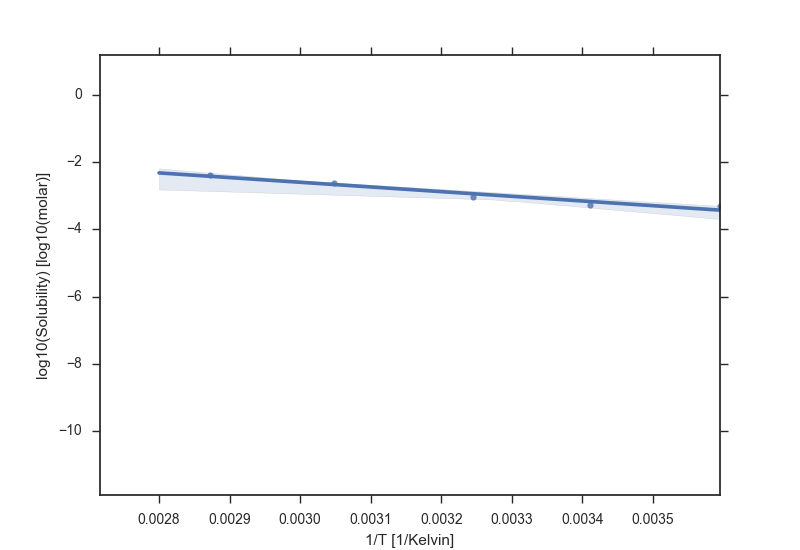

Supplement: Supplementary file 3 — Additional file 3. Additional results files, in electronic format. These additional results are (a) SUB-48 calculated lattice energies for the complete set and filtered set of 27 crystal structures, (b) results from analysis of the correspondence between our temperature dependent solubility data and (1/T), (c) Excel workbooks documenting all R2 and RMSE values obtained from cross-validation, their mean values and the p values (both raw and adjusted) obtained from pairwise comparisons of corresponding models, (d) comparison of melting point data used for the melting point descriptor and retrieved from the CSD for linked refcodes. [file 13321_2018_298_MOESM3_ESM.zip › additional_results/vantHoff_assessment/vh_plots/Klimenko_QSPRin_CD_False_MD_IntegSub.SiRMSSub.Absolv.Ind.Rdk_readyForR_vh.csv_208.tiff]

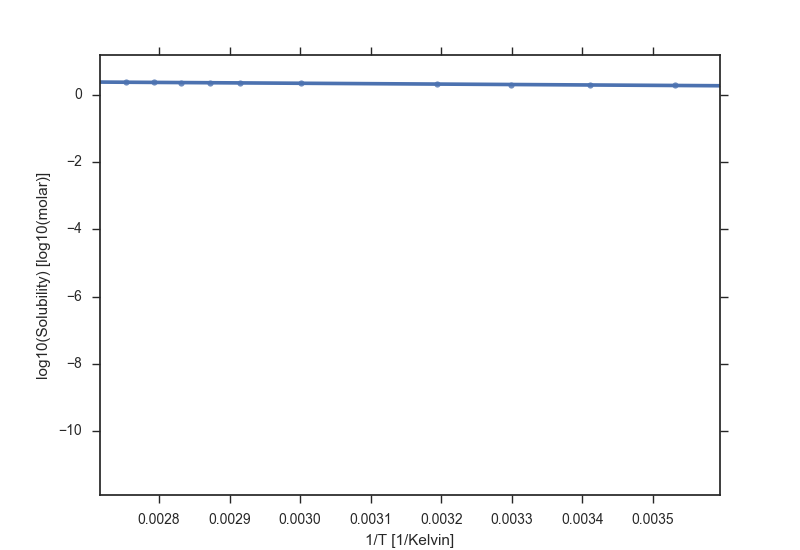

Supplement: Supplementary file 3 — Additional file 3. Additional results files, in electronic format. These additional results are (a) SUB-48 calculated lattice energies for the complete set and filtered set of 27 crystal structures, (b) results from analysis of the correspondence between our temperature dependent solubility data and (1/T), (c) Excel workbooks documenting all R2 and RMSE values obtained from cross-validation, their mean values and the p values (both raw and adjusted) obtained from pairwise comparisons of corresponding models, (d) comparison of melting point data used for the melting point descriptor and retrieved from the CSD for linked refcodes. [file 13321_2018_298_MOESM3_ESM.zip › additional_results/vantHoff_assessment/vh_plots/Klimenko_QSPRin_CD_False_MD_IntegSub.SiRMSSub.Absolv.Ind.Rdk_readyForR_vh.csv_214.tiff]

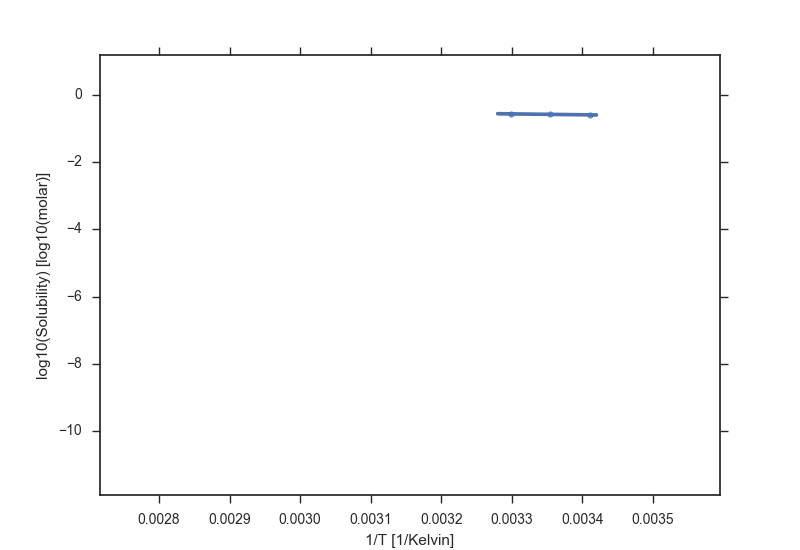

Supplement: Supplementary file 3 — Additional file 3. Additional results files, in electronic format. These additional results are (a) SUB-48 calculated lattice energies for the complete set and filtered set of 27 crystal structures, (b) results from analysis of the correspondence between our temperature dependent solubility data and (1/T), (c) Excel workbooks documenting all R2 and RMSE values obtained from cross-validation, their mean values and the p values (both raw and adjusted) obtained from pairwise comparisons of corresponding models, (d) comparison of melting point data used for the melting point descriptor and retrieved from the CSD for linked refcodes. [file 13321_2018_298_MOESM3_ESM.zip › additional_results/vantHoff_assessment/vh_plots/Klimenko_QSPRin_CD_False_MD_IntegSub.SiRMSSub.Absolv.Ind.Rdk_readyForR_vh.csv_215.tiff]

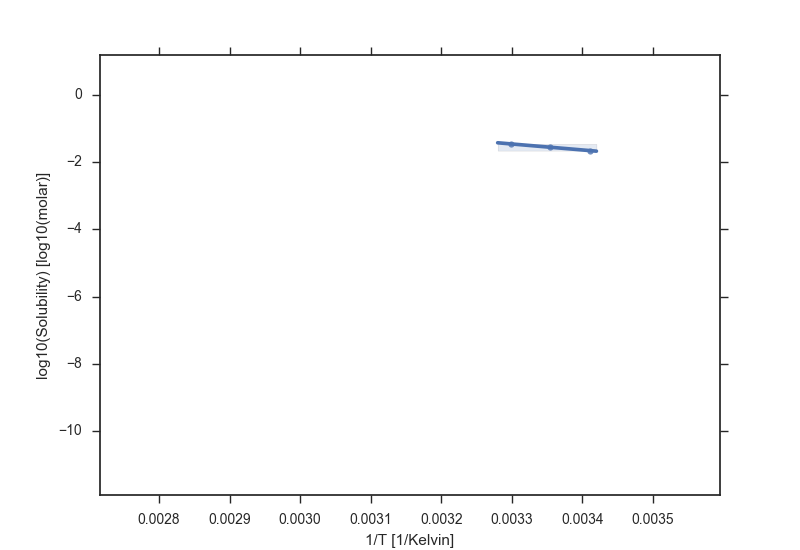

Supplement: Supplementary file 3 — Additional file 3. Additional results files, in electronic format. These additional results are (a) SUB-48 calculated lattice energies for the complete set and filtered set of 27 crystal structures, (b) results from analysis of the correspondence between our temperature dependent solubility data and (1/T), (c) Excel workbooks documenting all R2 and RMSE values obtained from cross-validation, their mean values and the p values (both raw and adjusted) obtained from pairwise comparisons of corresponding models, (d) comparison of melting point data used for the melting point descriptor and retrieved from the CSD for linked refcodes. [file 13321_2018_298_MOESM3_ESM.zip › additional_results/vantHoff_assessment/vh_plots/Klimenko_QSPRin_CD_False_MD_IntegSub.SiRMSSub.Absolv.Ind.Rdk_readyForR_vh.csv_218.tiff]

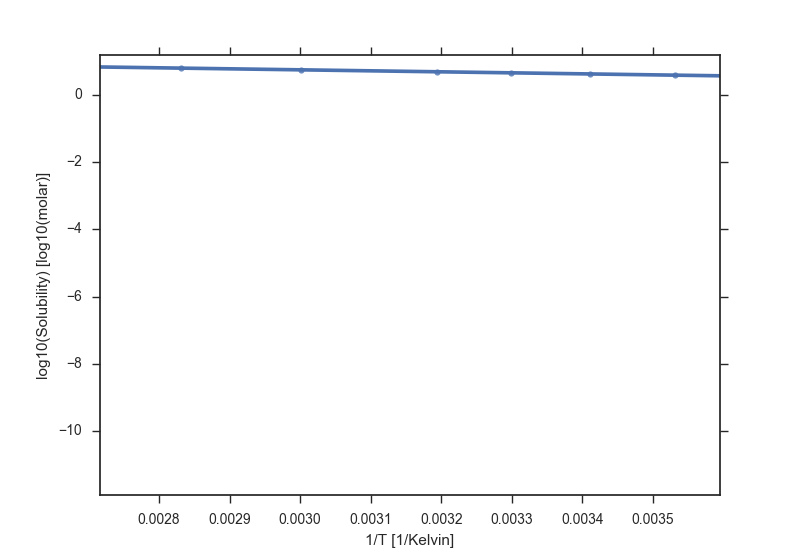

Supplement: Supplementary file 3 — Additional file 3. Additional results files, in electronic format. These additional results are (a) SUB-48 calculated lattice energies for the complete set and filtered set of 27 crystal structures, (b) results from analysis of the correspondence between our temperature dependent solubility data and (1/T), (c) Excel workbooks documenting all R2 and RMSE values obtained from cross-validation, their mean values and the p values (both raw and adjusted) obtained from pairwise comparisons of corresponding models, (d) comparison of melting point data used for the melting point descriptor and retrieved from the CSD for linked refcodes. [file 13321_2018_298_MOESM3_ESM.zip › additional_results/vantHoff_assessment/vh_plots/Klimenko_QSPRin_CD_False_MD_IntegSub.SiRMSSub.Absolv.Ind.Rdk_readyForR_vh.csv_22.tiff]

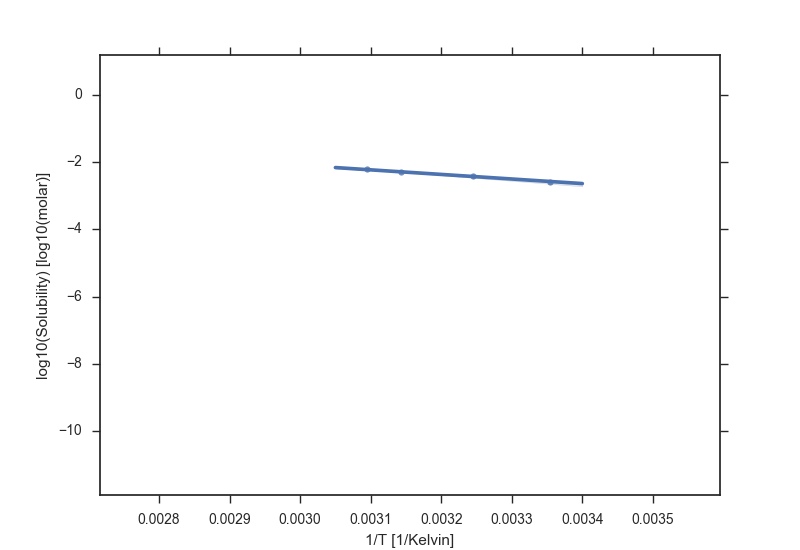

Supplement: Supplementary file 3 — Additional file 3. Additional results files, in electronic format. These additional results are (a) SUB-48 calculated lattice energies for the complete set and filtered set of 27 crystal structures, (b) results from analysis of the correspondence between our temperature dependent solubility data and (1/T), (c) Excel workbooks documenting all R2 and RMSE values obtained from cross-validation, their mean values and the p values (both raw and adjusted) obtained from pairwise comparisons of corresponding models, (d) comparison of melting point data used for the melting point descriptor and retrieved from the CSD for linked refcodes. [file 13321_2018_298_MOESM3_ESM.zip › additional_results/vantHoff_assessment/vh_plots/Klimenko_QSPRin_CD_False_MD_IntegSub.SiRMSSub.Absolv.Ind.Rdk_readyForR_vh.csv_223.tiff]

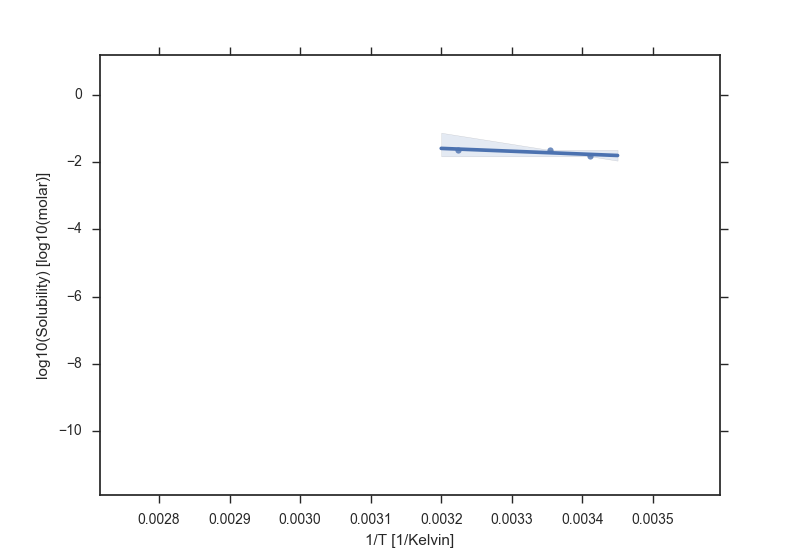

Supplement: Supplementary file 3 — Additional file 3. Additional results files, in electronic format. These additional results are (a) SUB-48 calculated lattice energies for the complete set and filtered set of 27 crystal structures, (b) results from analysis of the correspondence between our temperature dependent solubility data and (1/T), (c) Excel workbooks documenting all R2 and RMSE values obtained from cross-validation, their mean values and the p values (both raw and adjusted) obtained from pairwise comparisons of corresponding models, (d) comparison of melting point data used for the melting point descriptor and retrieved from the CSD for linked refcodes. [file 13321_2018_298_MOESM3_ESM.zip › additional_results/vantHoff_assessment/vh_plots/Klimenko_QSPRin_CD_False_MD_IntegSub.SiRMSSub.Absolv.Ind.Rdk_readyForR_vh.csv_224.tiff]

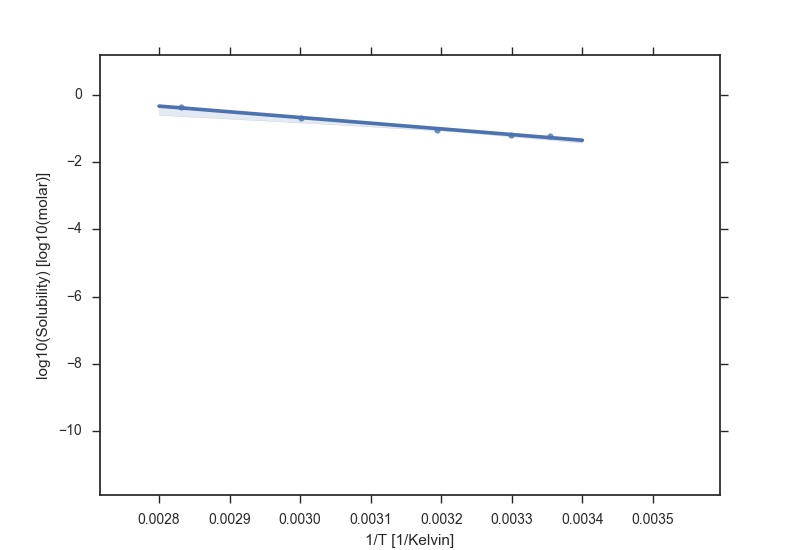

Supplement: Supplementary file 3 — Additional file 3. Additional results files, in electronic format. These additional results are (a) SUB-48 calculated lattice energies for the complete set and filtered set of 27 crystal structures, (b) results from analysis of the correspondence between our temperature dependent solubility data and (1/T), (c) Excel workbooks documenting all R2 and RMSE values obtained from cross-validation, their mean values and the p values (both raw and adjusted) obtained from pairwise comparisons of corresponding models, (d) comparison of melting point data used for the melting point descriptor and retrieved from the CSD for linked refcodes. [file 13321_2018_298_MOESM3_ESM.zip › additional_results/vantHoff_assessment/vh_plots/Klimenko_QSPRin_CD_False_MD_IntegSub.SiRMSSub.Absolv.Ind.Rdk_readyForR_vh.csv_228.tiff]

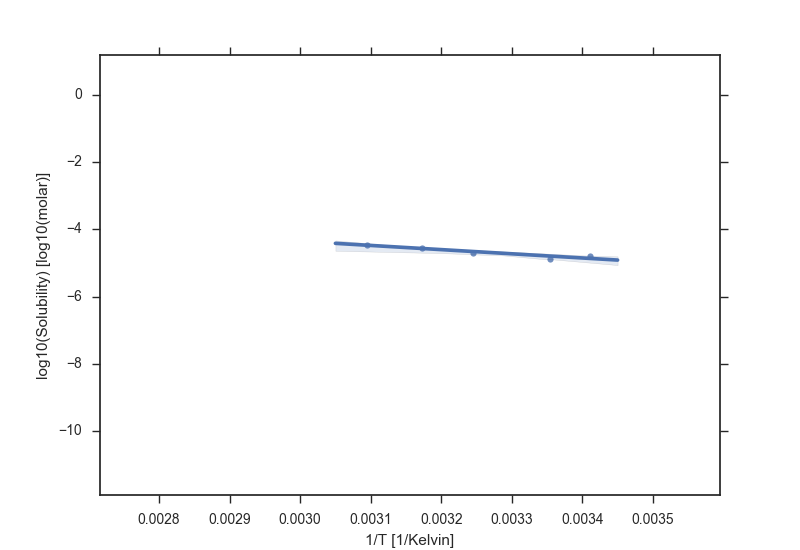

Supplement: Supplementary file 3 — Additional file 3. Additional results files, in electronic format. These additional results are (a) SUB-48 calculated lattice energies for the complete set and filtered set of 27 crystal structures, (b) results from analysis of the correspondence between our temperature dependent solubility data and (1/T), (c) Excel workbooks documenting all R2 and RMSE values obtained from cross-validation, their mean values and the p values (both raw and adjusted) obtained from pairwise comparisons of corresponding models, (d) comparison of melting point data used for the melting point descriptor and retrieved from the CSD for linked refcodes. [file 13321_2018_298_MOESM3_ESM.zip › additional_results/vantHoff_assessment/vh_plots/Klimenko_QSPRin_CD_False_MD_IntegSub.SiRMSSub.Absolv.Ind.Rdk_readyForR_vh.csv_231.tiff]

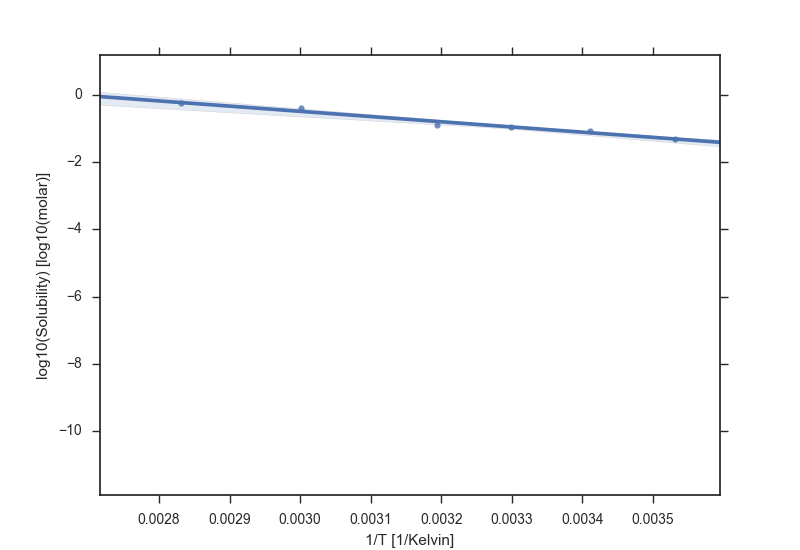

Supplement: Supplementary file 3 — Additional file 3. Additional results files, in electronic format. These additional results are (a) SUB-48 calculated lattice energies for the complete set and filtered set of 27 crystal structures, (b) results from analysis of the correspondence between our temperature dependent solubility data and (1/T), (c) Excel workbooks documenting all R2 and RMSE values obtained from cross-validation, their mean values and the p values (both raw and adjusted) obtained from pairwise comparisons of corresponding models, (d) comparison of melting point data used for the melting point descriptor and retrieved from the CSD for linked refcodes. [file 13321_2018_298_MOESM3_ESM.zip › additional_results/vantHoff_assessment/vh_plots/Klimenko_QSPRin_CD_False_MD_IntegSub.SiRMSSub.Absolv.Ind.Rdk_readyForR_vh.csv_238.tiff]

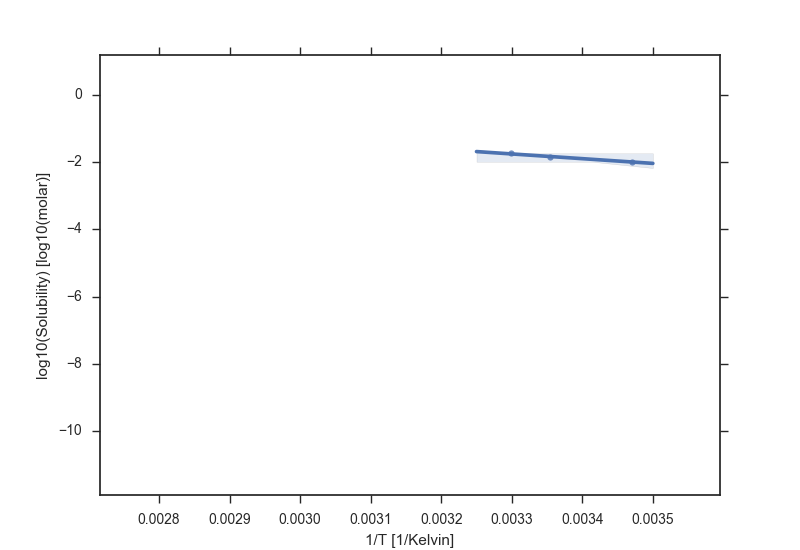

Supplement: Supplementary file 3 — Additional file 3. Additional results files, in electronic format. These additional results are (a) SUB-48 calculated lattice energies for the complete set and filtered set of 27 crystal structures, (b) results from analysis of the correspondence between our temperature dependent solubility data and (1/T), (c) Excel workbooks documenting all R2 and RMSE values obtained from cross-validation, their mean values and the p values (both raw and adjusted) obtained from pairwise comparisons of corresponding models, (d) comparison of melting point data used for the melting point descriptor and retrieved from the CSD for linked refcodes. [file 13321_2018_298_MOESM3_ESM.zip › additional_results/vantHoff_assessment/vh_plots/Klimenko_QSPRin_CD_False_MD_IntegSub.SiRMSSub.Absolv.Ind.Rdk_readyForR_vh.csv_239.tiff]

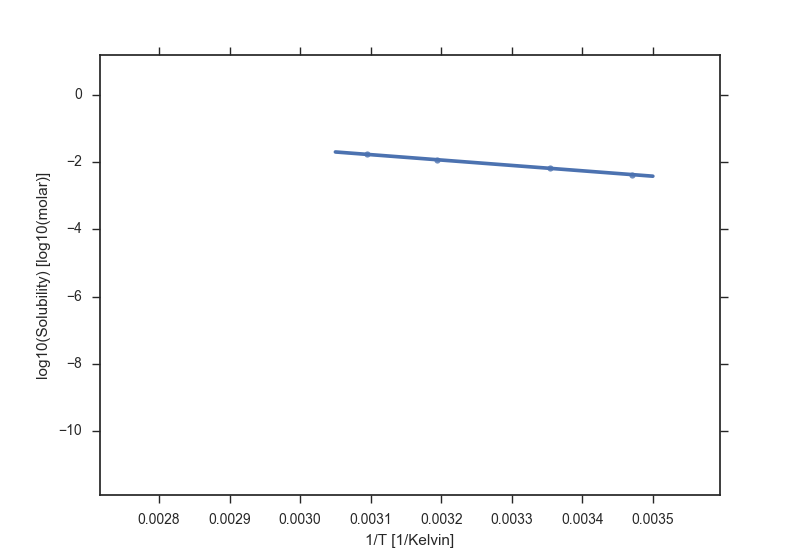

Supplement: Supplementary file 3 — Additional file 3. Additional results files, in electronic format. These additional results are (a) SUB-48 calculated lattice energies for the complete set and filtered set of 27 crystal structures, (b) results from analysis of the correspondence between our temperature dependent solubility data and (1/T), (c) Excel workbooks documenting all R2 and RMSE values obtained from cross-validation, their mean values and the p values (both raw and adjusted) obtained from pairwise comparisons of corresponding models, (d) comparison of melting point data used for the melting point descriptor and retrieved from the CSD for linked refcodes. [file 13321_2018_298_MOESM3_ESM.zip › additional_results/vantHoff_assessment/vh_plots/Klimenko_QSPRin_CD_False_MD_IntegSub.SiRMSSub.Absolv.Ind.Rdk_readyForR_vh.csv_24.tiff]

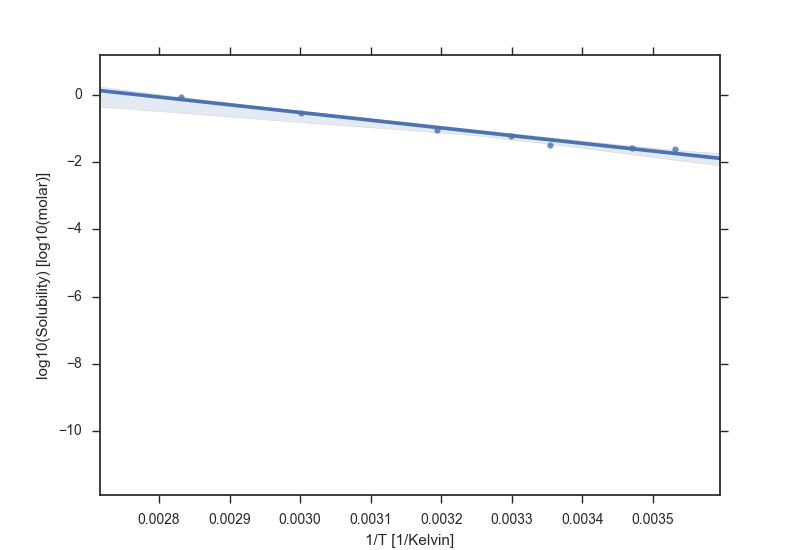

Supplement: Supplementary file 3 — Additional file 3. Additional results files, in electronic format. These additional results are (a) SUB-48 calculated lattice energies for the complete set and filtered set of 27 crystal structures, (b) results from analysis of the correspondence between our temperature dependent solubility data and (1/T), (c) Excel workbooks documenting all R2 and RMSE values obtained from cross-validation, their mean values and the p values (both raw and adjusted) obtained from pairwise comparisons of corresponding models, (d) comparison of melting point data used for the melting point descriptor and retrieved from the CSD for linked refcodes. [file 13321_2018_298_MOESM3_ESM.zip › additional_results/vantHoff_assessment/vh_plots/Klimenko_QSPRin_CD_False_MD_IntegSub.SiRMSSub.Absolv.Ind.Rdk_readyForR_vh.csv_241.tiff]

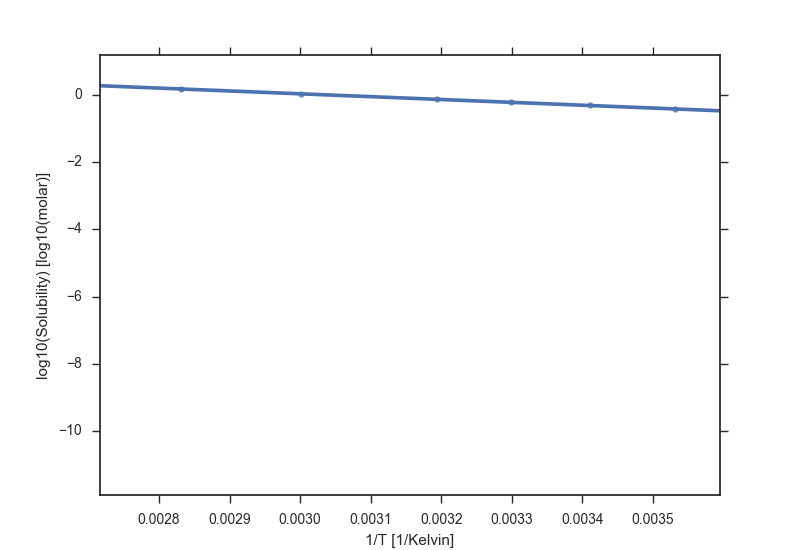

Supplement: Supplementary file 3 — Additional file 3. Additional results files, in electronic format. These additional results are (a) SUB-48 calculated lattice energies for the complete set and filtered set of 27 crystal structures, (b) results from analysis of the correspondence between our temperature dependent solubility data and (1/T), (c) Excel workbooks documenting all R2 and RMSE values obtained from cross-validation, their mean values and the p values (both raw and adjusted) obtained from pairwise comparisons of corresponding models, (d) comparison of melting point data used for the melting point descriptor and retrieved from the CSD for linked refcodes. [file 13321_2018_298_MOESM3_ESM.zip › additional_results/vantHoff_assessment/vh_plots/Klimenko_QSPRin_CD_False_MD_IntegSub.SiRMSSub.Absolv.Ind.Rdk_readyForR_vh.csv_243.tiff]

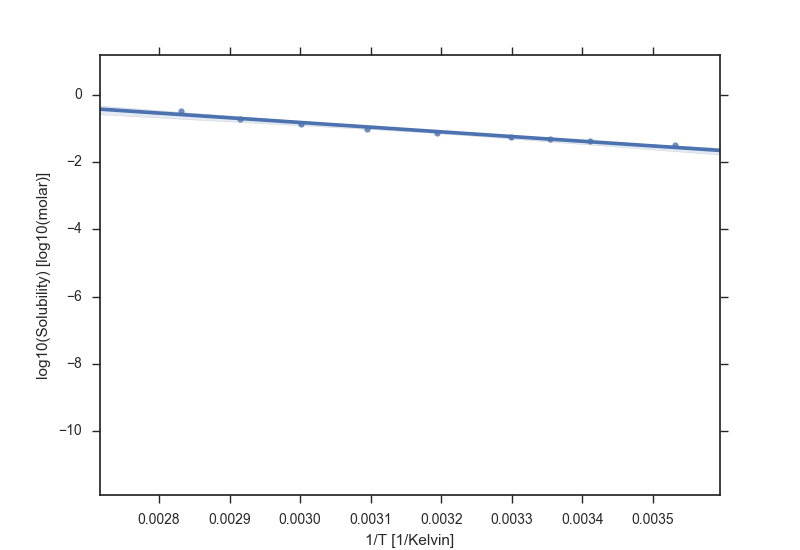

Supplement: Supplementary file 3 — Additional file 3. Additional results files, in electronic format. These additional results are (a) SUB-48 calculated lattice energies for the complete set and filtered set of 27 crystal structures, (b) results from analysis of the correspondence between our temperature dependent solubility data and (1/T), (c) Excel workbooks documenting all R2 and RMSE values obtained from cross-validation, their mean values and the p values (both raw and adjusted) obtained from pairwise comparisons of corresponding models, (d) comparison of melting point data used for the melting point descriptor and retrieved from the CSD for linked refcodes. [file 13321_2018_298_MOESM3_ESM.zip › additional_results/vantHoff_assessment/vh_plots/Klimenko_QSPRin_CD_False_MD_IntegSub.SiRMSSub.Absolv.Ind.Rdk_readyForR_vh.csv_246.tiff]

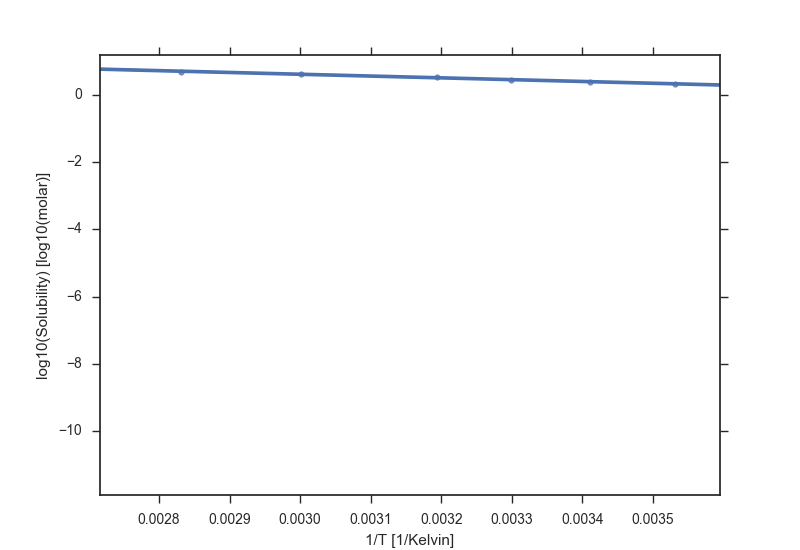

Supplement: Supplementary file 3 — Additional file 3. Additional results files, in electronic format. These additional results are (a) SUB-48 calculated lattice energies for the complete set and filtered set of 27 crystal structures, (b) results from analysis of the correspondence between our temperature dependent solubility data and (1/T), (c) Excel workbooks documenting all R2 and RMSE values obtained from cross-validation, their mean values and the p values (both raw and adjusted) obtained from pairwise comparisons of corresponding models, (d) comparison of melting point data used for the melting point descriptor and retrieved from the CSD for linked refcodes. [file 13321_2018_298_MOESM3_ESM.zip › additional_results/vantHoff_assessment/vh_plots/Klimenko_QSPRin_CD_False_MD_IntegSub.SiRMSSub.Absolv.Ind.Rdk_readyForR_vh.csv_247.tiff]

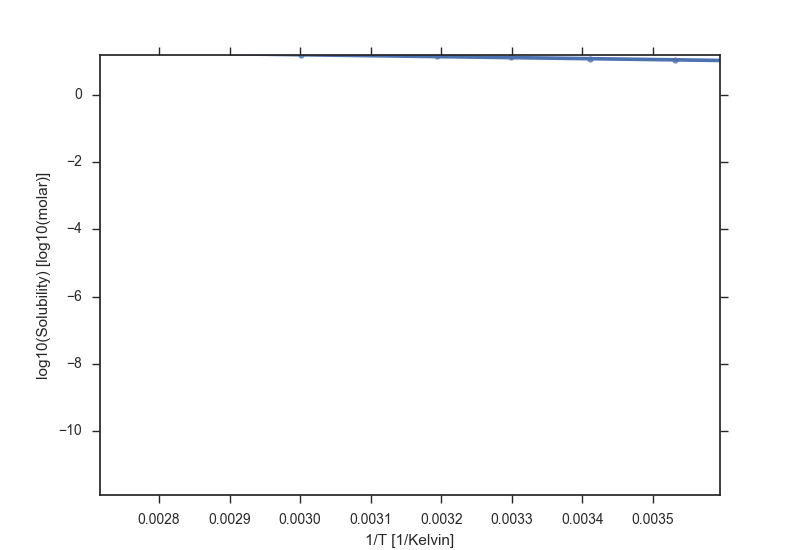

Supplement: Supplementary file 3 — Additional file 3. Additional results files, in electronic format. These additional results are (a) SUB-48 calculated lattice energies for the complete set and filtered set of 27 crystal structures, (b) results from analysis of the correspondence between our temperature dependent solubility data and (1/T), (c) Excel workbooks documenting all R2 and RMSE values obtained from cross-validation, their mean values and the p values (both raw and adjusted) obtained from pairwise comparisons of corresponding models, (d) comparison of melting point data used for the melting point descriptor and retrieved from the CSD for linked refcodes. [file 13321_2018_298_MOESM3_ESM.zip › additional_results/vantHoff_assessment/vh_plots/Klimenko_QSPRin_CD_False_MD_IntegSub.SiRMSSub.Absolv.Ind.Rdk_readyForR_vh.csv_249.tiff]

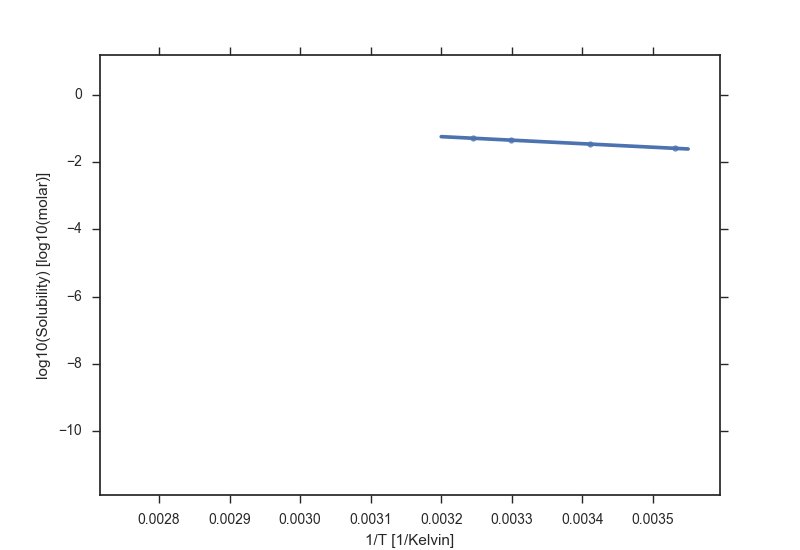

Supplement: Supplementary file 3 — Additional file 3. Additional results files, in electronic format. These additional results are (a) SUB-48 calculated lattice energies for the complete set and filtered set of 27 crystal structures, (b) results from analysis of the correspondence between our temperature dependent solubility data and (1/T), (c) Excel workbooks documenting all R2 and RMSE values obtained from cross-validation, their mean values and the p values (both raw and adjusted) obtained from pairwise comparisons of corresponding models, (d) comparison of melting point data used for the melting point descriptor and retrieved from the CSD for linked refcodes. [file 13321_2018_298_MOESM3_ESM.zip › additional_results/vantHoff_assessment/vh_plots/Klimenko_QSPRin_CD_False_MD_IntegSub.SiRMSSub.Absolv.Ind.Rdk_readyForR_vh.csv_251.tiff]

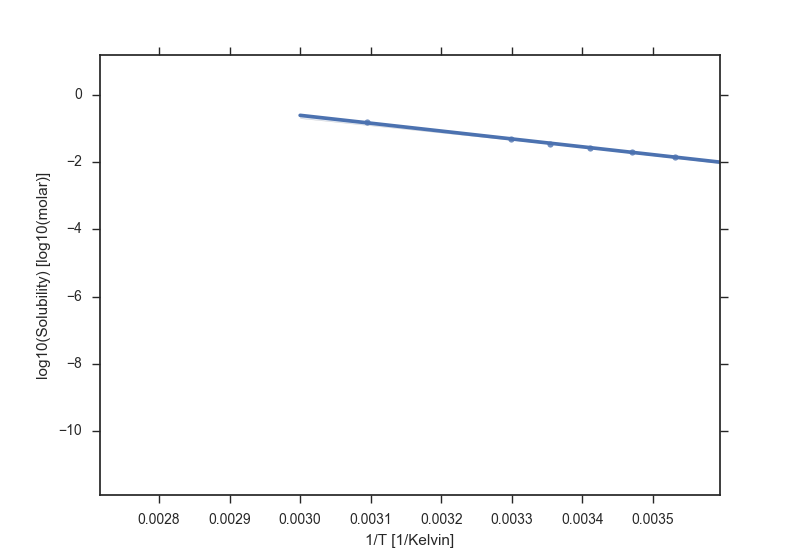

Supplement: Supplementary file 3 — Additional file 3. Additional results files, in electronic format. These additional results are (a) SUB-48 calculated lattice energies for the complete set and filtered set of 27 crystal structures, (b) results from analysis of the correspondence between our temperature dependent solubility data and (1/T), (c) Excel workbooks documenting all R2 and RMSE values obtained from cross-validation, their mean values and the p values (both raw and adjusted) obtained from pairwise comparisons of corresponding models, (d) comparison of melting point data used for the melting point descriptor and retrieved from the CSD for linked refcodes. [file 13321_2018_298_MOESM3_ESM.zip › additional_results/vantHoff_assessment/vh_plots/Klimenko_QSPRin_CD_False_MD_IntegSub.SiRMSSub.Absolv.Ind.Rdk_readyForR_vh.csv_253.tiff]

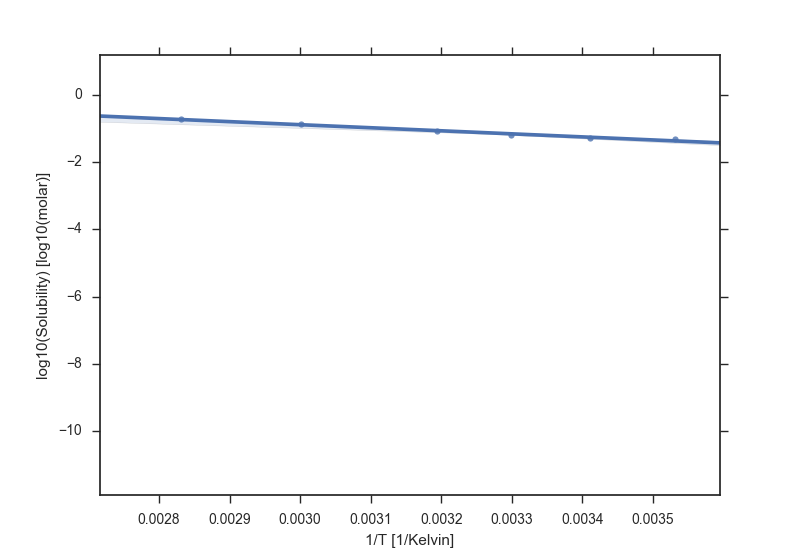

Supplement: Supplementary file 3 — Additional file 3. Additional results files, in electronic format. These additional results are (a) SUB-48 calculated lattice energies for the complete set and filtered set of 27 crystal structures, (b) results from analysis of the correspondence between our temperature dependent solubility data and (1/T), (c) Excel workbooks documenting all R2 and RMSE values obtained from cross-validation, their mean values and the p values (both raw and adjusted) obtained from pairwise comparisons of corresponding models, (d) comparison of melting point data used for the melting point descriptor and retrieved from the CSD for linked refcodes. [file 13321_2018_298_MOESM3_ESM.zip › additional_results/vantHoff_assessment/vh_plots/Klimenko_QSPRin_CD_False_MD_IntegSub.SiRMSSub.Absolv.Ind.Rdk_readyForR_vh.csv_254.tiff]

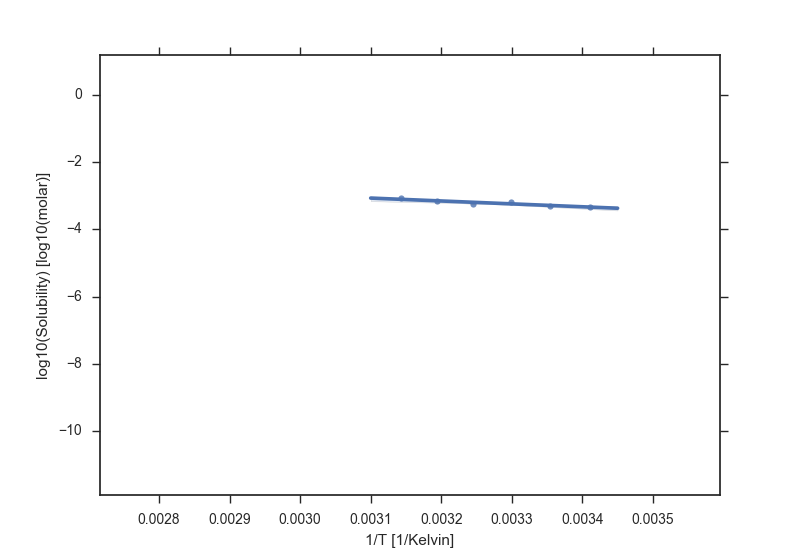

Supplement: Supplementary file 3 — Additional file 3. Additional results files, in electronic format. These additional results are (a) SUB-48 calculated lattice energies for the complete set and filtered set of 27 crystal structures, (b) results from analysis of the correspondence between our temperature dependent solubility data and (1/T), (c) Excel workbooks documenting all R2 and RMSE values obtained from cross-validation, their mean values and the p values (both raw and adjusted) obtained from pairwise comparisons of corresponding models, (d) comparison of melting point data used for the melting point descriptor and retrieved from the CSD for linked refcodes. [file 13321_2018_298_MOESM3_ESM.zip › additional_results/vantHoff_assessment/vh_plots/Klimenko_QSPRin_CD_False_MD_IntegSub.SiRMSSub.Absolv.Ind.Rdk_readyForR_vh.csv_257.tiff]

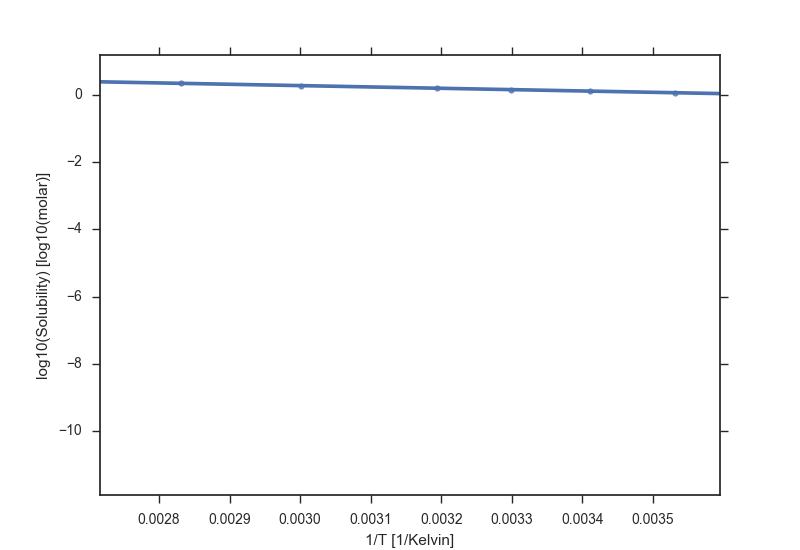

Supplement: Supplementary file 3 — Additional file 3. Additional results files, in electronic format. These additional results are (a) SUB-48 calculated lattice energies for the complete set and filtered set of 27 crystal structures, (b) results from analysis of the correspondence between our temperature dependent solubility data and (1/T), (c) Excel workbooks documenting all R2 and RMSE values obtained from cross-validation, their mean values and the p values (both raw and adjusted) obtained from pairwise comparisons of corresponding models, (d) comparison of melting point data used for the melting point descriptor and retrieved from the CSD for linked refcodes. [file 13321_2018_298_MOESM3_ESM.zip › additional_results/vantHoff_assessment/vh_plots/Klimenko_QSPRin_CD_False_MD_IntegSub.SiRMSSub.Absolv.Ind.Rdk_readyForR_vh.csv_261.tiff]

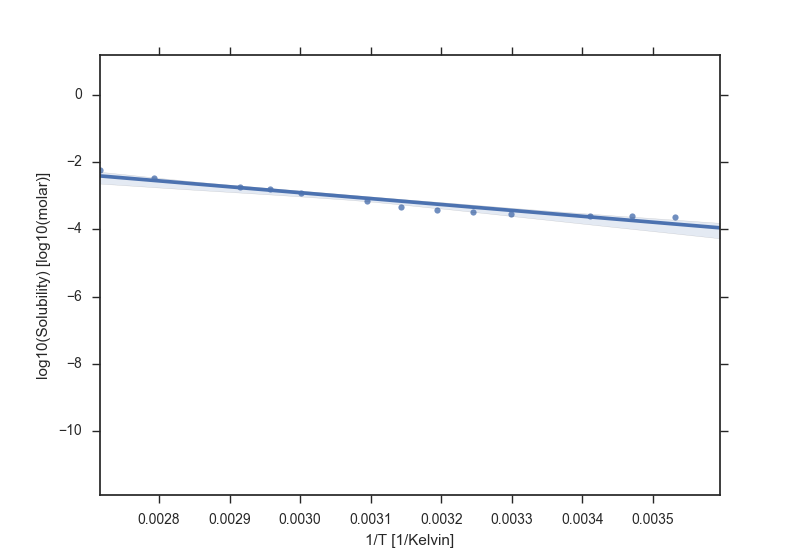

Supplement: Supplementary file 3 — Additional file 3. Additional results files, in electronic format. These additional results are (a) SUB-48 calculated lattice energies for the complete set and filtered set of 27 crystal structures, (b) results from analysis of the correspondence between our temperature dependent solubility data and (1/T), (c) Excel workbooks documenting all R2 and RMSE values obtained from cross-validation, their mean values and the p values (both raw and adjusted) obtained from pairwise comparisons of corresponding models, (d) comparison of melting point data used for the melting point descriptor and retrieved from the CSD for linked refcodes. [file 13321_2018_298_MOESM3_ESM.zip › additional_results/vantHoff_assessment/vh_plots/Klimenko_QSPRin_CD_False_MD_IntegSub.SiRMSSub.Absolv.Ind.Rdk_readyForR_vh.csv_262.tiff]

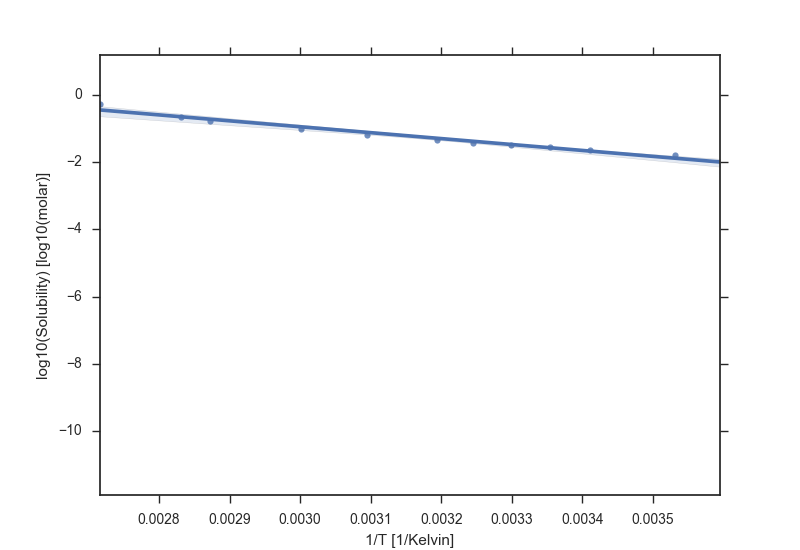

Supplement: Supplementary file 3 — Additional file 3. Additional results files, in electronic format. These additional results are (a) SUB-48 calculated lattice energies for the complete set and filtered set of 27 crystal structures, (b) results from analysis of the correspondence between our temperature dependent solubility data and (1/T), (c) Excel workbooks documenting all R2 and RMSE values obtained from cross-validation, their mean values and the p values (both raw and adjusted) obtained from pairwise comparisons of corresponding models, (d) comparison of melting point data used for the melting point descriptor and retrieved from the CSD for linked refcodes. [file 13321_2018_298_MOESM3_ESM.zip › additional_results/vantHoff_assessment/vh_plots/Klimenko_QSPRin_CD_False_MD_IntegSub.SiRMSSub.Absolv.Ind.Rdk_readyForR_vh.csv_265.tiff]

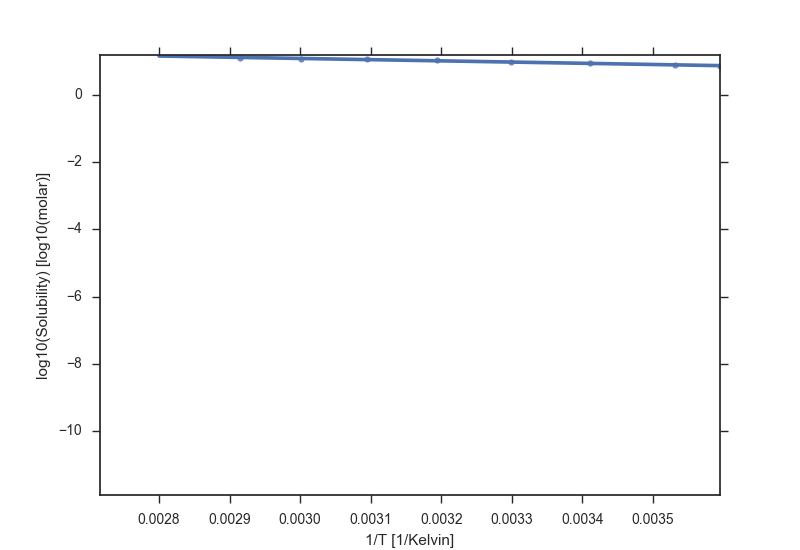

Supplement: Supplementary file 3 — Additional file 3. Additional results files, in electronic format. These additional results are (a) SUB-48 calculated lattice energies for the complete set and filtered set of 27 crystal structures, (b) results from analysis of the correspondence between our temperature dependent solubility data and (1/T), (c) Excel workbooks documenting all R2 and RMSE values obtained from cross-validation, their mean values and the p values (both raw and adjusted) obtained from pairwise comparisons of corresponding models, (d) comparison of melting point data used for the melting point descriptor and retrieved from the CSD for linked refcodes. [file 13321_2018_298_MOESM3_ESM.zip › additional_results/vantHoff_assessment/vh_plots/Klimenko_QSPRin_CD_False_MD_IntegSub.SiRMSSub.Absolv.Ind.Rdk_readyForR_vh.csv_266.tiff]

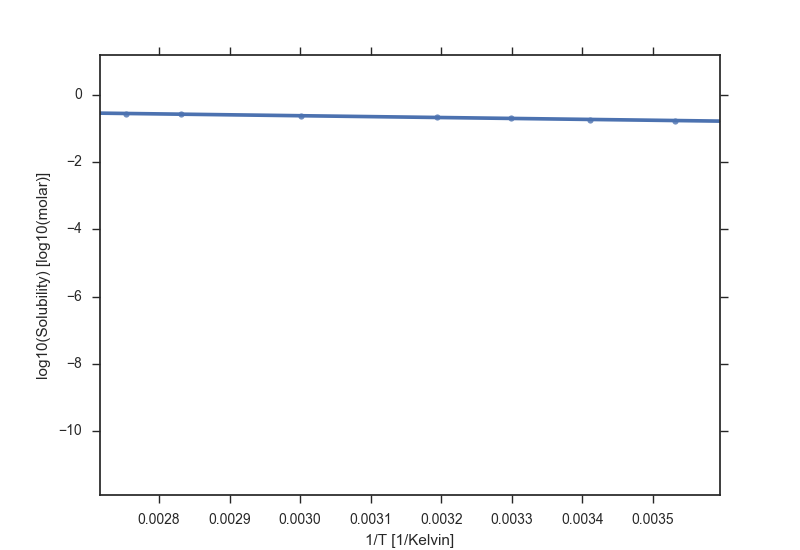

Supplement: Supplementary file 3 — Additional file 3. Additional results files, in electronic format. These additional results are (a) SUB-48 calculated lattice energies for the complete set and filtered set of 27 crystal structures, (b) results from analysis of the correspondence between our temperature dependent solubility data and (1/T), (c) Excel workbooks documenting all R2 and RMSE values obtained from cross-validation, their mean values and the p values (both raw and adjusted) obtained from pairwise comparisons of corresponding models, (d) comparison of melting point data used for the melting point descriptor and retrieved from the CSD for linked refcodes. [file 13321_2018_298_MOESM3_ESM.zip › additional_results/vantHoff_assessment/vh_plots/Klimenko_QSPRin_CD_False_MD_IntegSub.SiRMSSub.Absolv.Ind.Rdk_readyForR_vh.csv_267.tiff]

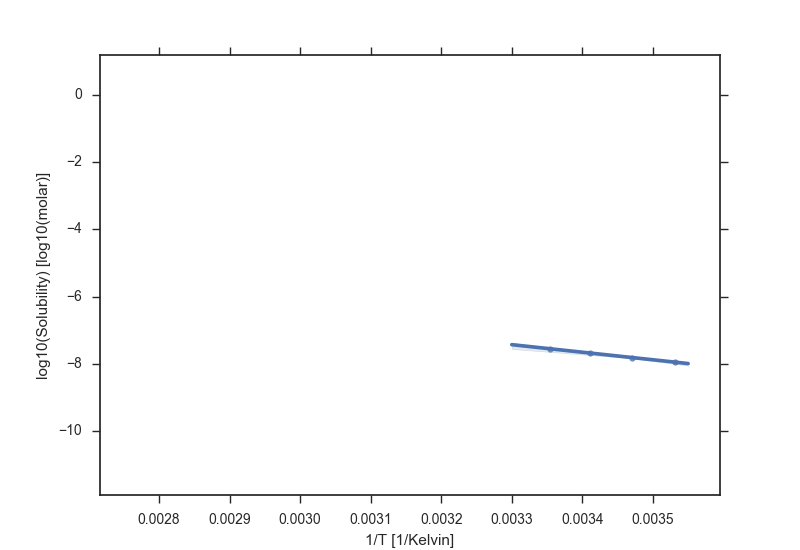

Supplement: Supplementary file 3 — Additional file 3. Additional results files, in electronic format. These additional results are (a) SUB-48 calculated lattice energies for the complete set and filtered set of 27 crystal structures, (b) results from analysis of the correspondence between our temperature dependent solubility data and (1/T), (c) Excel workbooks documenting all R2 and RMSE values obtained from cross-validation, their mean values and the p values (both raw and adjusted) obtained from pairwise comparisons of corresponding models, (d) comparison of melting point data used for the melting point descriptor and retrieved from the CSD for linked refcodes. [file 13321_2018_298_MOESM3_ESM.zip › additional_results/vantHoff_assessment/vh_plots/Klimenko_QSPRin_CD_False_MD_IntegSub.SiRMSSub.Absolv.Ind.Rdk_readyForR_vh.csv_268.tiff]

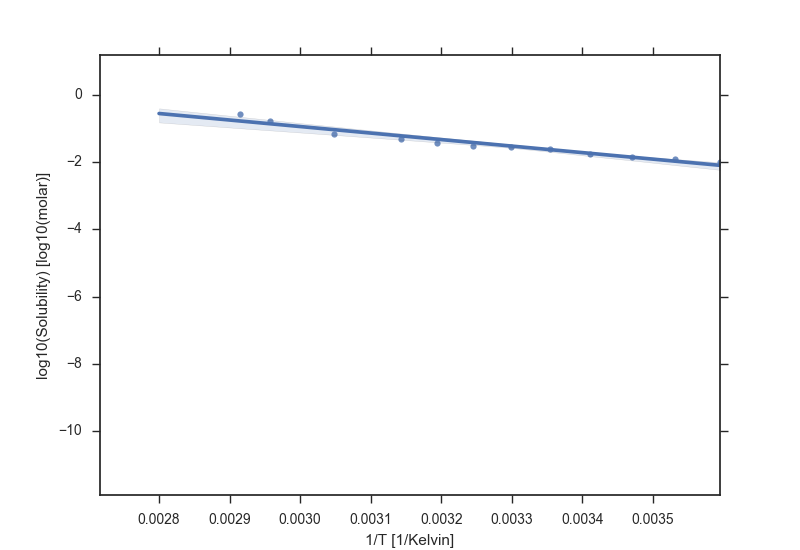

Supplement: Supplementary file 3 — Additional file 3. Additional results files, in electronic format. These additional results are (a) SUB-48 calculated lattice energies for the complete set and filtered set of 27 crystal structures, (b) results from analysis of the correspondence between our temperature dependent solubility data and (1/T), (c) Excel workbooks documenting all R2 and RMSE values obtained from cross-validation, their mean values and the p values (both raw and adjusted) obtained from pairwise comparisons of corresponding models, (d) comparison of melting point data used for the melting point descriptor and retrieved from the CSD for linked refcodes. [file 13321_2018_298_MOESM3_ESM.zip › additional_results/vantHoff_assessment/vh_plots/Klimenko_QSPRin_CD_False_MD_IntegSub.SiRMSSub.Absolv.Ind.Rdk_readyForR_vh.csv_27.tiff]

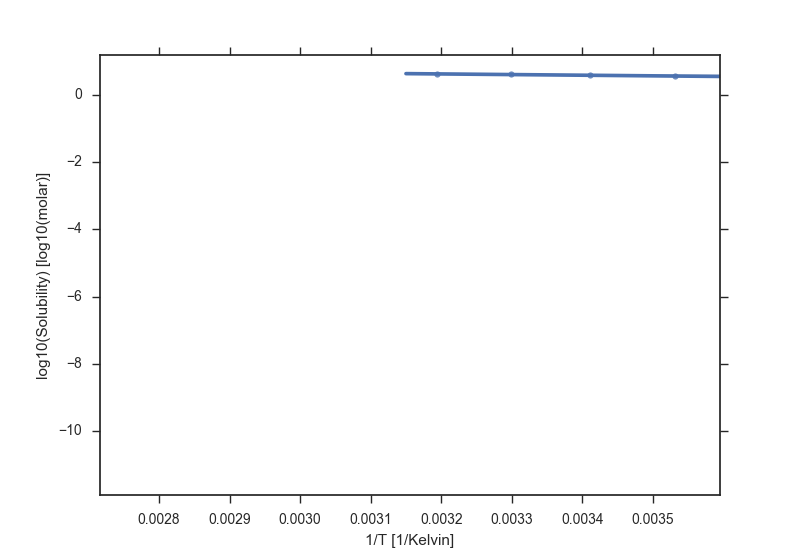

Supplement: Supplementary file 3 — Additional file 3. Additional results files, in electronic format. These additional results are (a) SUB-48 calculated lattice energies for the complete set and filtered set of 27 crystal structures, (b) results from analysis of the correspondence between our temperature dependent solubility data and (1/T), (c) Excel workbooks documenting all R2 and RMSE values obtained from cross-validation, their mean values and the p values (both raw and adjusted) obtained from pairwise comparisons of corresponding models, (d) comparison of melting point data used for the melting point descriptor and retrieved from the CSD for linked refcodes. [file 13321_2018_298_MOESM3_ESM.zip › additional_results/vantHoff_assessment/vh_plots/Klimenko_QSPRin_CD_False_MD_IntegSub.SiRMSSub.Absolv.Ind.Rdk_readyForR_vh.csv_270.tiff]

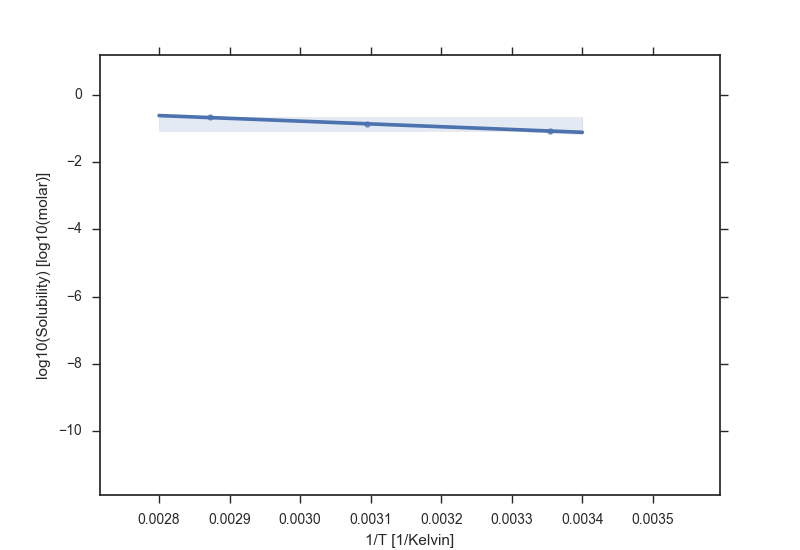

Supplement: Supplementary file 3 — Additional file 3. Additional results files, in electronic format. These additional results are (a) SUB-48 calculated lattice energies for the complete set and filtered set of 27 crystal structures, (b) results from analysis of the correspondence between our temperature dependent solubility data and (1/T), (c) Excel workbooks documenting all R2 and RMSE values obtained from cross-validation, their mean values and the p values (both raw and adjusted) obtained from pairwise comparisons of corresponding models, (d) comparison of melting point data used for the melting point descriptor and retrieved from the CSD for linked refcodes. [file 13321_2018_298_MOESM3_ESM.zip › additional_results/vantHoff_assessment/vh_plots/Klimenko_QSPRin_CD_False_MD_IntegSub.SiRMSSub.Absolv.Ind.Rdk_readyForR_vh.csv_272.tiff]

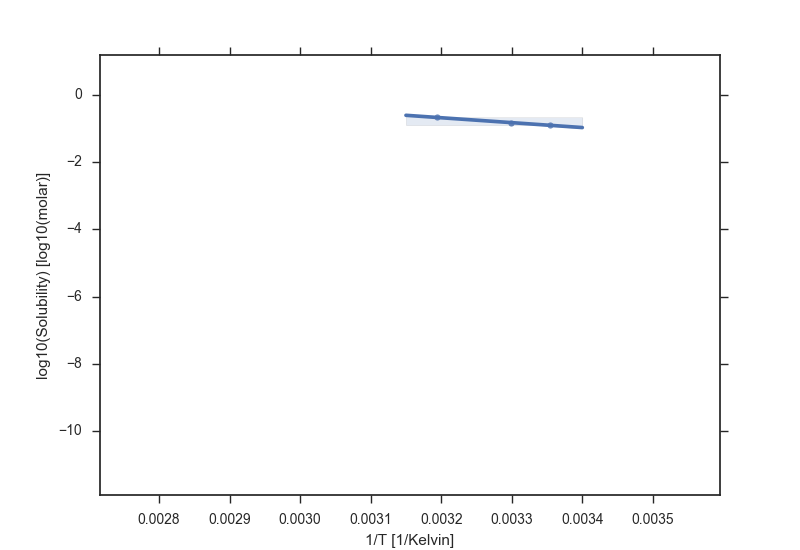

Supplement: Supplementary file 3 — Additional file 3. Additional results files, in electronic format. These additional results are (a) SUB-48 calculated lattice energies for the complete set and filtered set of 27 crystal structures, (b) results from analysis of the correspondence between our temperature dependent solubility data and (1/T), (c) Excel workbooks documenting all R2 and RMSE values obtained from cross-validation, their mean values and the p values (both raw and adjusted) obtained from pairwise comparisons of corresponding models, (d) comparison of melting point data used for the melting point descriptor and retrieved from the CSD for linked refcodes. [file 13321_2018_298_MOESM3_ESM.zip › additional_results/vantHoff_assessment/vh_plots/Klimenko_QSPRin_CD_False_MD_IntegSub.SiRMSSub.Absolv.Ind.Rdk_readyForR_vh.csv_275.tiff]

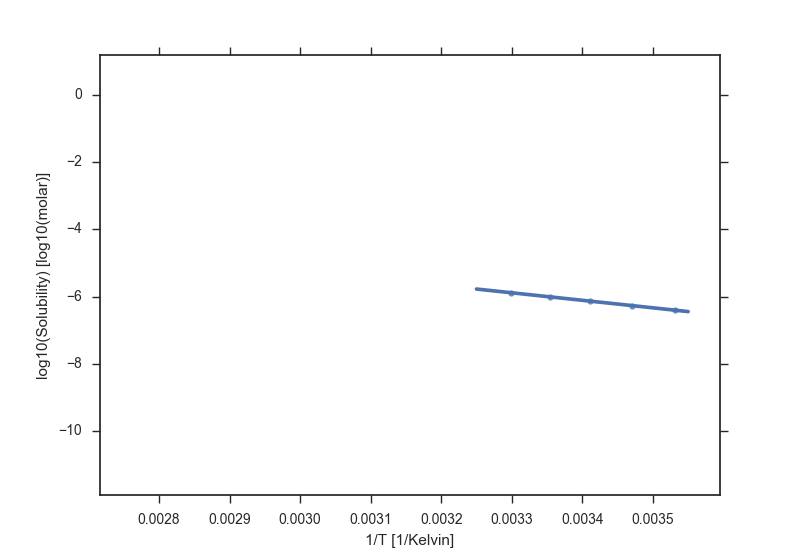

Supplement: Supplementary file 3 — Additional file 3. Additional results files, in electronic format. These additional results are (a) SUB-48 calculated lattice energies for the complete set and filtered set of 27 crystal structures, (b) results from analysis of the correspondence between our temperature dependent solubility data and (1/T), (c) Excel workbooks documenting all R2 and RMSE values obtained from cross-validation, their mean values and the p values (both raw and adjusted) obtained from pairwise comparisons of corresponding models, (d) comparison of melting point data used for the melting point descriptor and retrieved from the CSD for linked refcodes. [file 13321_2018_298_MOESM3_ESM.zip › additional_results/vantHoff_assessment/vh_plots/Klimenko_QSPRin_CD_False_MD_IntegSub.SiRMSSub.Absolv.Ind.Rdk_readyForR_vh.csv_276.tiff]

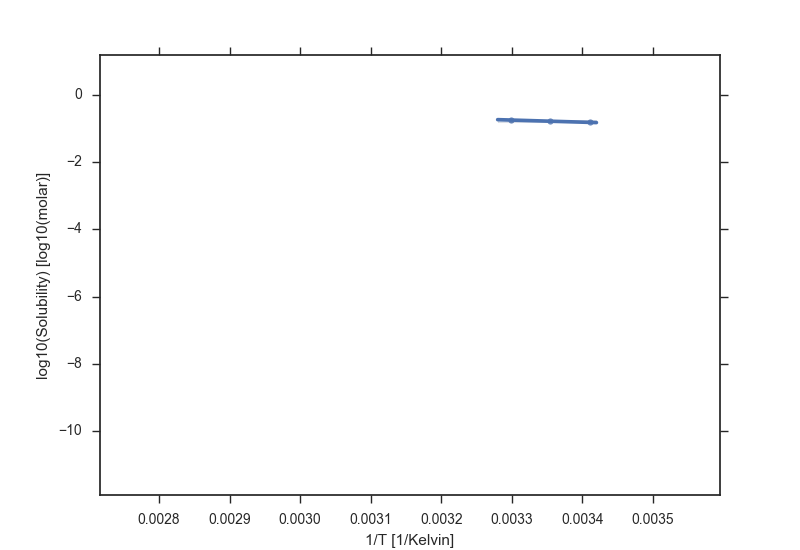

Supplement: Supplementary file 3 — Additional file 3. Additional results files, in electronic format. These additional results are (a) SUB-48 calculated lattice energies for the complete set and filtered set of 27 crystal structures, (b) results from analysis of the correspondence between our temperature dependent solubility data and (1/T), (c) Excel workbooks documenting all R2 and RMSE values obtained from cross-validation, their mean values and the p values (both raw and adjusted) obtained from pairwise comparisons of corresponding models, (d) comparison of melting point data used for the melting point descriptor and retrieved from the CSD for linked refcodes. [file 13321_2018_298_MOESM3_ESM.zip › additional_results/vantHoff_assessment/vh_plots/Klimenko_QSPRin_CD_False_MD_IntegSub.SiRMSSub.Absolv.Ind.Rdk_readyForR_vh.csv_278.tiff]

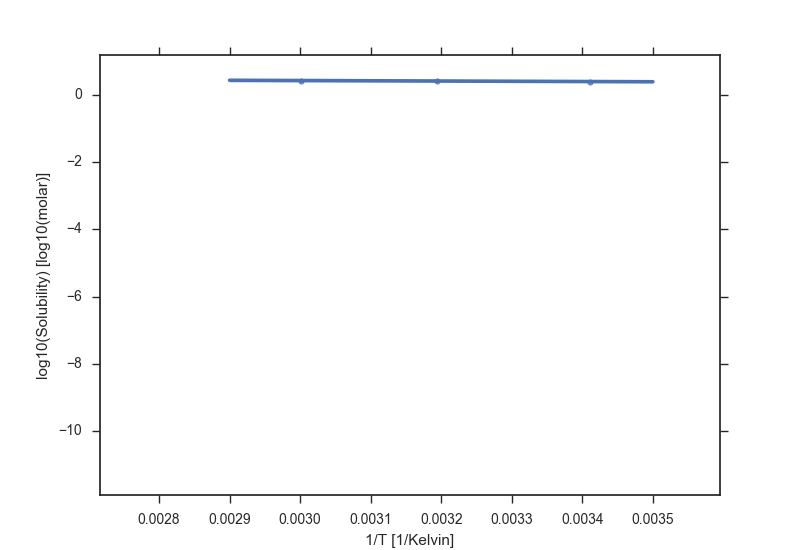

Supplement: Supplementary file 3 — Additional file 3. Additional results files, in electronic format. These additional results are (a) SUB-48 calculated lattice energies for the complete set and filtered set of 27 crystal structures, (b) results from analysis of the correspondence between our temperature dependent solubility data and (1/T), (c) Excel workbooks documenting all R2 and RMSE values obtained from cross-validation, their mean values and the p values (both raw and adjusted) obtained from pairwise comparisons of corresponding models, (d) comparison of melting point data used for the melting point descriptor and retrieved from the CSD for linked refcodes. [file 13321_2018_298_MOESM3_ESM.zip › additional_results/vantHoff_assessment/vh_plots/Klimenko_QSPRin_CD_False_MD_IntegSub.SiRMSSub.Absolv.Ind.Rdk_readyForR_vh.csv_280.tiff]

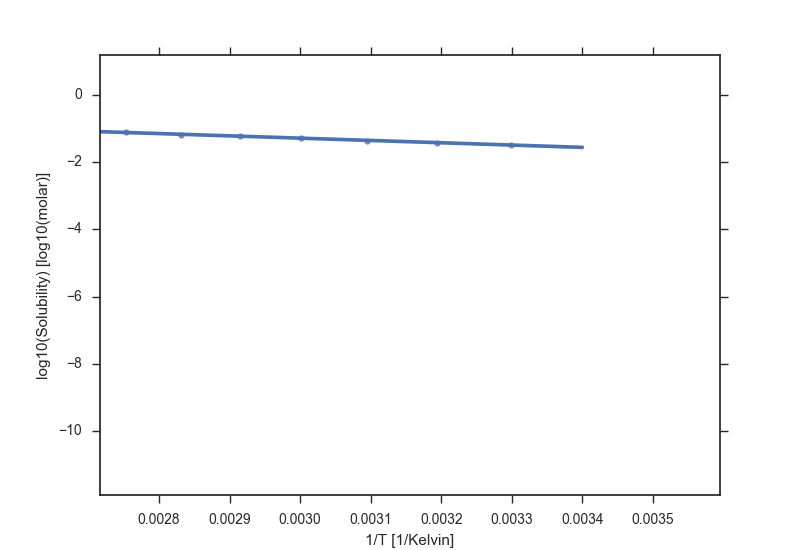

Supplement: Supplementary file 3 — Additional file 3. Additional results files, in electronic format. These additional results are (a) SUB-48 calculated lattice energies for the complete set and filtered set of 27 crystal structures, (b) results from analysis of the correspondence between our temperature dependent solubility data and (1/T), (c) Excel workbooks documenting all R2 and RMSE values obtained from cross-validation, their mean values and the p values (both raw and adjusted) obtained from pairwise comparisons of corresponding models, (d) comparison of melting point data used for the melting point descriptor and retrieved from the CSD for linked refcodes. [file 13321_2018_298_MOESM3_ESM.zip › additional_results/vantHoff_assessment/vh_plots/Klimenko_QSPRin_CD_False_MD_IntegSub.SiRMSSub.Absolv.Ind.Rdk_readyForR_vh.csv_281.tiff]

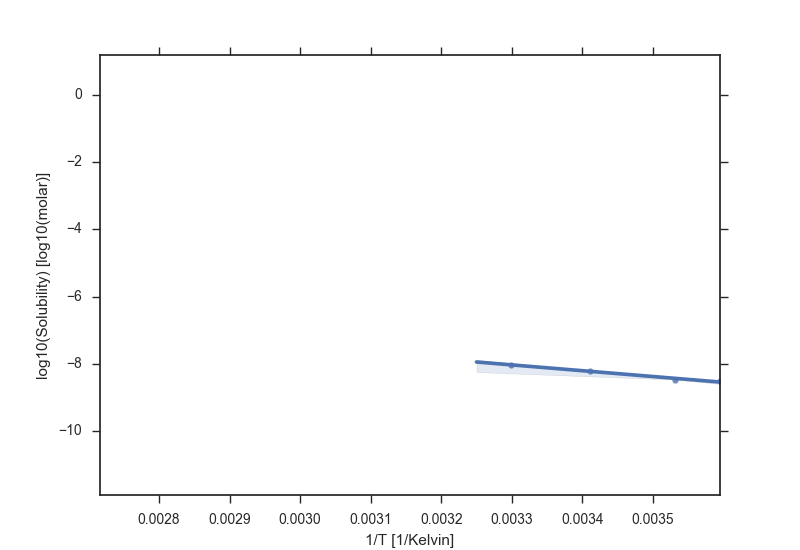

Supplement: Supplementary file 3 — Additional file 3. Additional results files, in electronic format. These additional results are (a) SUB-48 calculated lattice energies for the complete set and filtered set of 27 crystal structures, (b) results from analysis of the correspondence between our temperature dependent solubility data and (1/T), (c) Excel workbooks documenting all R2 and RMSE values obtained from cross-validation, their mean values and the p values (both raw and adjusted) obtained from pairwise comparisons of corresponding models, (d) comparison of melting point data used for the melting point descriptor and retrieved from the CSD for linked refcodes. [file 13321_2018_298_MOESM3_ESM.zip › additional_results/vantHoff_assessment/vh_plots/Klimenko_QSPRin_CD_False_MD_IntegSub.SiRMSSub.Absolv.Ind.Rdk_readyForR_vh.csv_284.tiff]

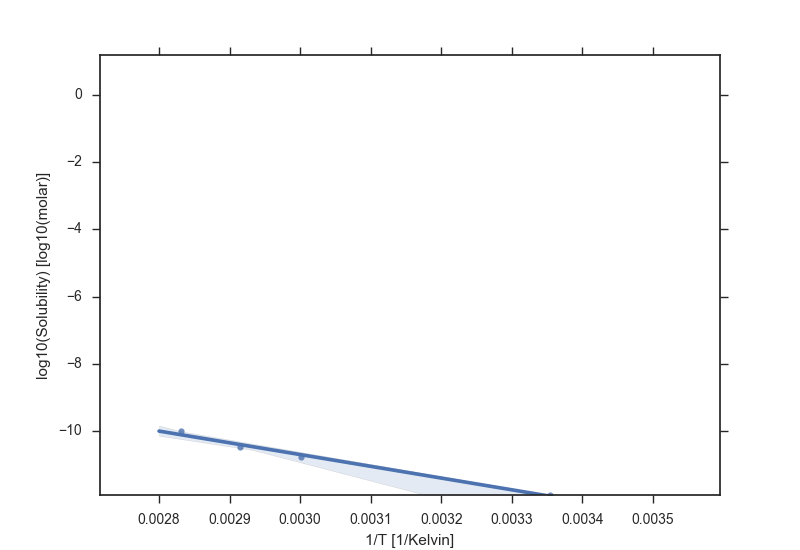

Supplement: Supplementary file 3 — Additional file 3. Additional results files, in electronic format. These additional results are (a) SUB-48 calculated lattice energies for the complete set and filtered set of 27 crystal structures, (b) results from analysis of the correspondence between our temperature dependent solubility data and (1/T), (c) Excel workbooks documenting all R2 and RMSE values obtained from cross-validation, their mean values and the p values (both raw and adjusted) obtained from pairwise comparisons of corresponding models, (d) comparison of melting point data used for the melting point descriptor and retrieved from the CSD for linked refcodes. [file 13321_2018_298_MOESM3_ESM.zip › additional_results/vantHoff_assessment/vh_plots/Klimenko_QSPRin_CD_False_MD_IntegSub.SiRMSSub.Absolv.Ind.Rdk_readyForR_vh.csv_285.tiff]

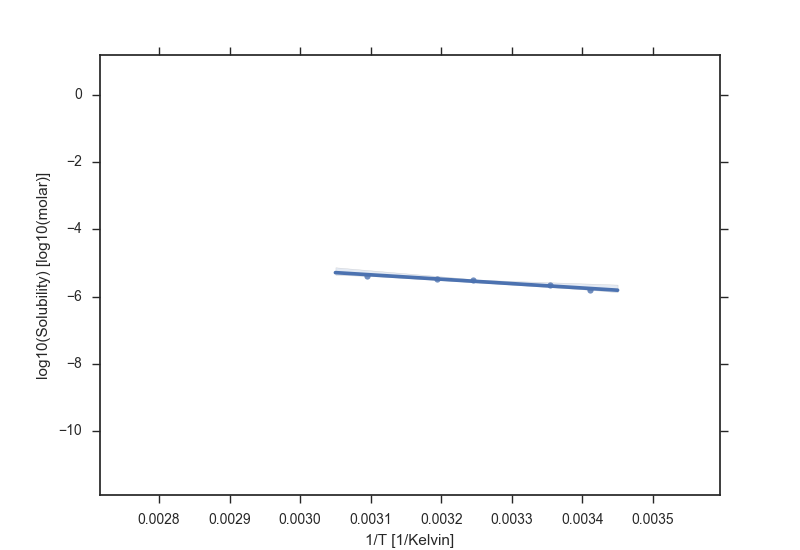

Supplement: Supplementary file 3 — Additional file 3. Additional results files, in electronic format. These additional results are (a) SUB-48 calculated lattice energies for the complete set and filtered set of 27 crystal structures, (b) results from analysis of the correspondence between our temperature dependent solubility data and (1/T), (c) Excel workbooks documenting all R2 and RMSE values obtained from cross-validation, their mean values and the p values (both raw and adjusted) obtained from pairwise comparisons of corresponding models, (d) comparison of melting point data used for the melting point descriptor and retrieved from the CSD for linked refcodes. [file 13321_2018_298_MOESM3_ESM.zip › additional_results/vantHoff_assessment/vh_plots/Klimenko_QSPRin_CD_False_MD_IntegSub.SiRMSSub.Absolv.Ind.Rdk_readyForR_vh.csv_287.tiff]

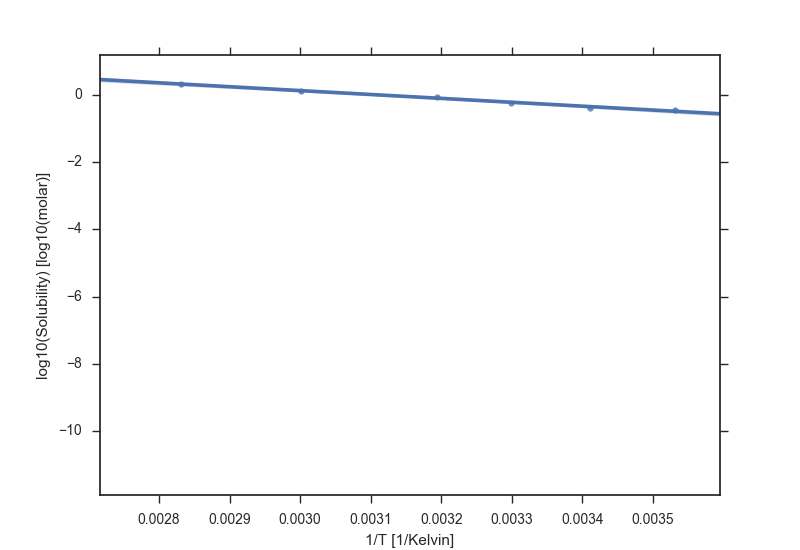

Supplement: Supplementary file 3 — Additional file 3. Additional results files, in electronic format. These additional results are (a) SUB-48 calculated lattice energies for the complete set and filtered set of 27 crystal structures, (b) results from analysis of the correspondence between our temperature dependent solubility data and (1/T), (c) Excel workbooks documenting all R2 and RMSE values obtained from cross-validation, their mean values and the p values (both raw and adjusted) obtained from pairwise comparisons of corresponding models, (d) comparison of melting point data used for the melting point descriptor and retrieved from the CSD for linked refcodes. [file 13321_2018_298_MOESM3_ESM.zip › additional_results/vantHoff_assessment/vh_plots/Klimenko_QSPRin_CD_False_MD_IntegSub.SiRMSSub.Absolv.Ind.Rdk_readyForR_vh.csv_289.tiff]

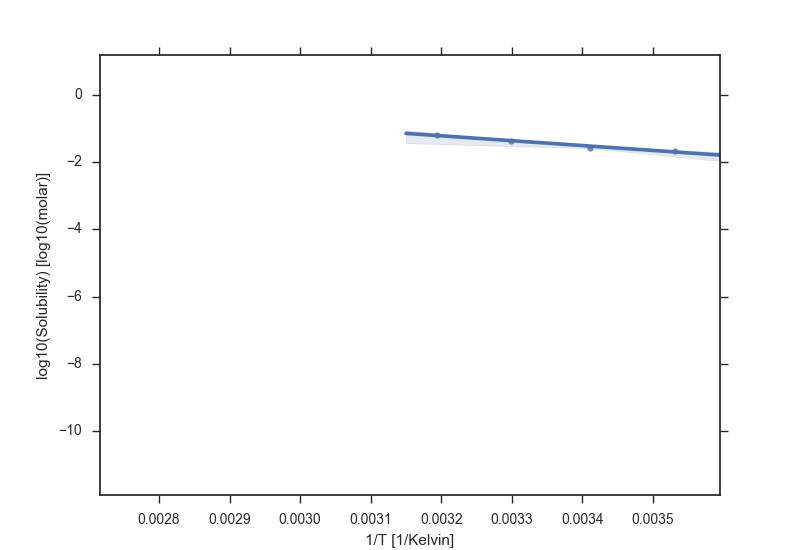

Supplement: Supplementary file 3 — Additional file 3. Additional results files, in electronic format. These additional results are (a) SUB-48 calculated lattice energies for the complete set and filtered set of 27 crystal structures, (b) results from analysis of the correspondence between our temperature dependent solubility data and (1/T), (c) Excel workbooks documenting all R2 and RMSE values obtained from cross-validation, their mean values and the p values (both raw and adjusted) obtained from pairwise comparisons of corresponding models, (d) comparison of melting point data used for the melting point descriptor and retrieved from the CSD for linked refcodes. [file 13321_2018_298_MOESM3_ESM.zip › additional_results/vantHoff_assessment/vh_plots/Klimenko_QSPRin_CD_False_MD_IntegSub.SiRMSSub.Absolv.Ind.Rdk_readyForR_vh.csv_29.tiff]

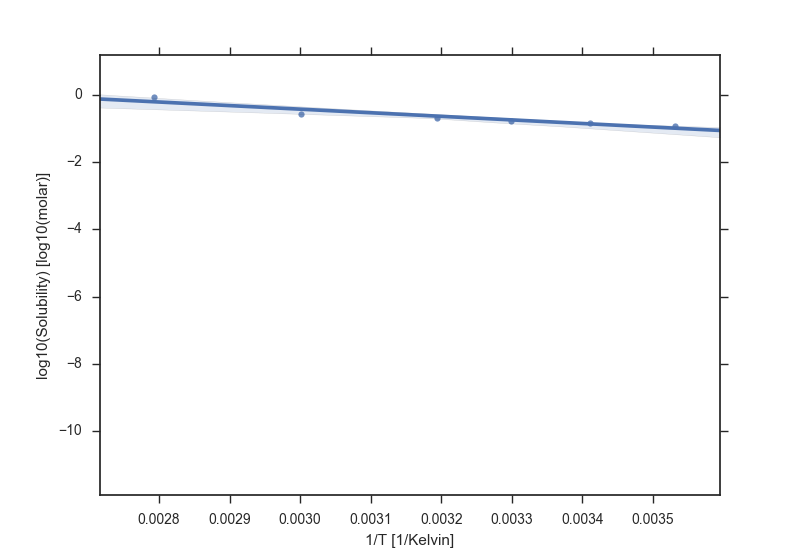

Supplement: Supplementary file 3 — Additional file 3. Additional results files, in electronic format. These additional results are (a) SUB-48 calculated lattice energies for the complete set and filtered set of 27 crystal structures, (b) results from analysis of the correspondence between our temperature dependent solubility data and (1/T), (c) Excel workbooks documenting all R2 and RMSE values obtained from cross-validation, their mean values and the p values (both raw and adjusted) obtained from pairwise comparisons of corresponding models, (d) comparison of melting point data used for the melting point descriptor and retrieved from the CSD for linked refcodes. [file 13321_2018_298_MOESM3_ESM.zip › additional_results/vantHoff_assessment/vh_plots/Klimenko_QSPRin_CD_False_MD_IntegSub.SiRMSSub.Absolv.Ind.Rdk_readyForR_vh.csv_290.tiff]

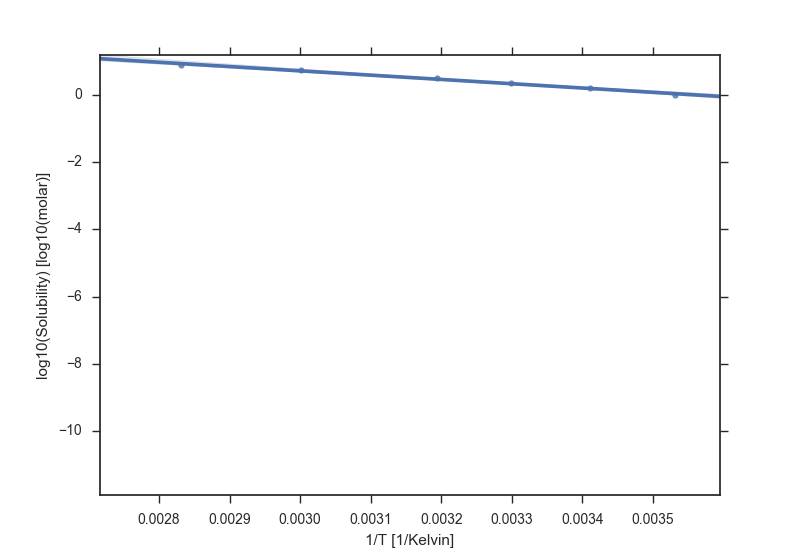

Supplement: Supplementary file 3 — Additional file 3. Additional results files, in electronic format. These additional results are (a) SUB-48 calculated lattice energies for the complete set and filtered set of 27 crystal structures, (b) results from analysis of the correspondence between our temperature dependent solubility data and (1/T), (c) Excel workbooks documenting all R2 and RMSE values obtained from cross-validation, their mean values and the p values (both raw and adjusted) obtained from pairwise comparisons of corresponding models, (d) comparison of melting point data used for the melting point descriptor and retrieved from the CSD for linked refcodes. [file 13321_2018_298_MOESM3_ESM.zip › additional_results/vantHoff_assessment/vh_plots/Klimenko_QSPRin_CD_False_MD_IntegSub.SiRMSSub.Absolv.Ind.Rdk_readyForR_vh.csv_292.tiff]

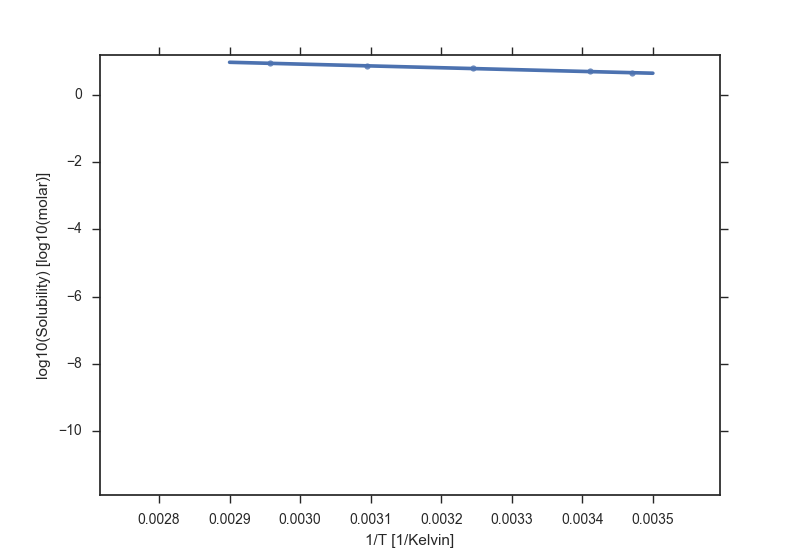

Supplement: Supplementary file 3 — Additional file 3. Additional results files, in electronic format. These additional results are (a) SUB-48 calculated lattice energies for the complete set and filtered set of 27 crystal structures, (b) results from analysis of the correspondence between our temperature dependent solubility data and (1/T), (c) Excel workbooks documenting all R2 and RMSE values obtained from cross-validation, their mean values and the p values (both raw and adjusted) obtained from pairwise comparisons of corresponding models, (d) comparison of melting point data used for the melting point descriptor and retrieved from the CSD for linked refcodes. [file 13321_2018_298_MOESM3_ESM.zip › additional_results/vantHoff_assessment/vh_plots/Klimenko_QSPRin_CD_False_MD_IntegSub.SiRMSSub.Absolv.Ind.Rdk_readyForR_vh.csv_294.tiff]

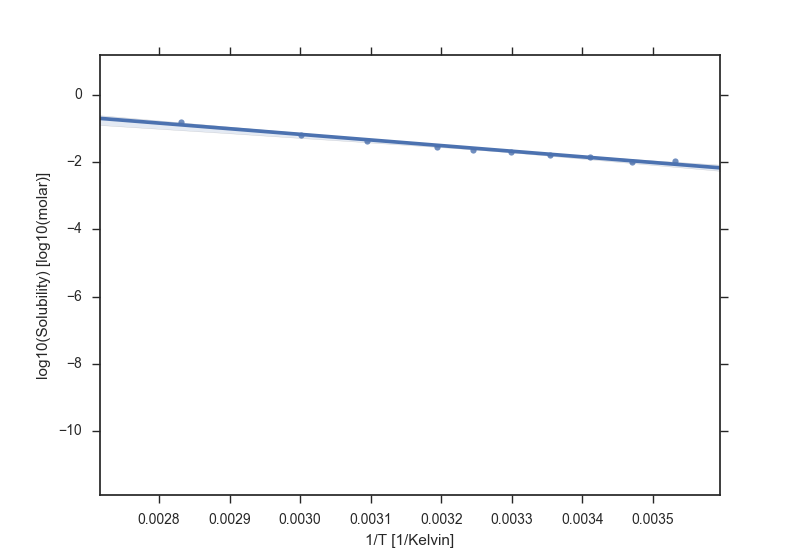

Supplement: Supplementary file 3 — Additional file 3. Additional results files, in electronic format. These additional results are (a) SUB-48 calculated lattice energies for the complete set and filtered set of 27 crystal structures, (b) results from analysis of the correspondence between our temperature dependent solubility data and (1/T), (c) Excel workbooks documenting all R2 and RMSE values obtained from cross-validation, their mean values and the p values (both raw and adjusted) obtained from pairwise comparisons of corresponding models, (d) comparison of melting point data used for the melting point descriptor and retrieved from the CSD for linked refcodes. [file 13321_2018_298_MOESM3_ESM.zip › additional_results/vantHoff_assessment/vh_plots/Klimenko_QSPRin_CD_False_MD_IntegSub.SiRMSSub.Absolv.Ind.Rdk_readyForR_vh.csv_296.tiff]

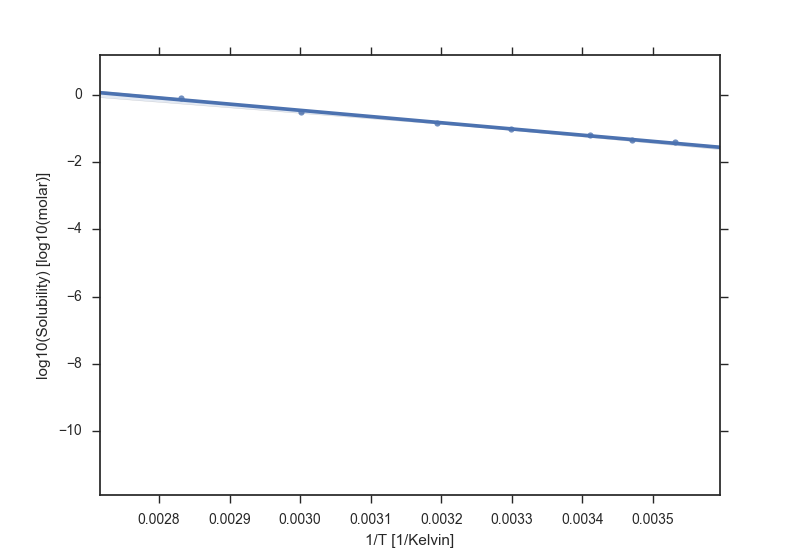

Supplement: Supplementary file 3 — Additional file 3. Additional results files, in electronic format. These additional results are (a) SUB-48 calculated lattice energies for the complete set and filtered set of 27 crystal structures, (b) results from analysis of the correspondence between our temperature dependent solubility data and (1/T), (c) Excel workbooks documenting all R2 and RMSE values obtained from cross-validation, their mean values and the p values (both raw and adjusted) obtained from pairwise comparisons of corresponding models, (d) comparison of melting point data used for the melting point descriptor and retrieved from the CSD for linked refcodes. [file 13321_2018_298_MOESM3_ESM.zip › additional_results/vantHoff_assessment/vh_plots/Klimenko_QSPRin_CD_False_MD_IntegSub.SiRMSSub.Absolv.Ind.Rdk_readyForR_vh.csv_3.tiff]

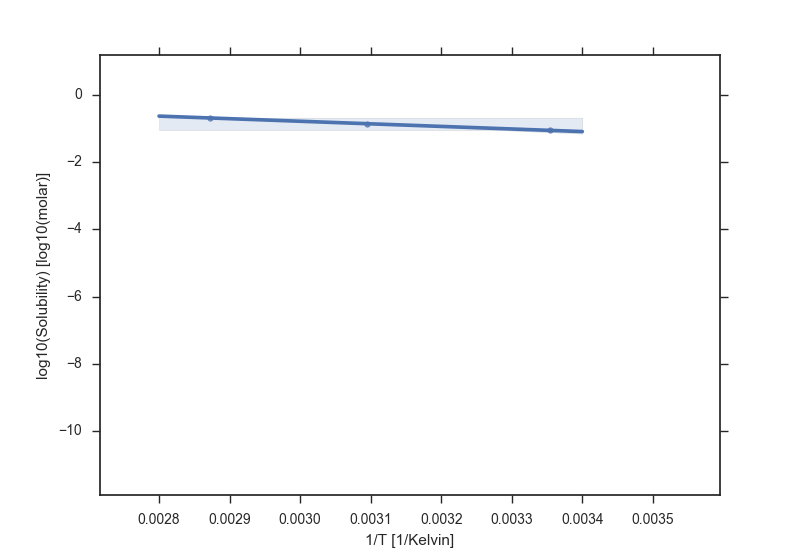

Supplement: Supplementary file 3 — Additional file 3. Additional results files, in electronic format. These additional results are (a) SUB-48 calculated lattice energies for the complete set and filtered set of 27 crystal structures, (b) results from analysis of the correspondence between our temperature dependent solubility data and (1/T), (c) Excel workbooks documenting all R2 and RMSE values obtained from cross-validation, their mean values and the p values (both raw and adjusted) obtained from pairwise comparisons of corresponding models, (d) comparison of melting point data used for the melting point descriptor and retrieved from the CSD for linked refcodes. [file 13321_2018_298_MOESM3_ESM.zip › additional_results/vantHoff_assessment/vh_plots/Klimenko_QSPRin_CD_False_MD_IntegSub.SiRMSSub.Absolv.Ind.Rdk_readyForR_vh.csv_304.tiff]

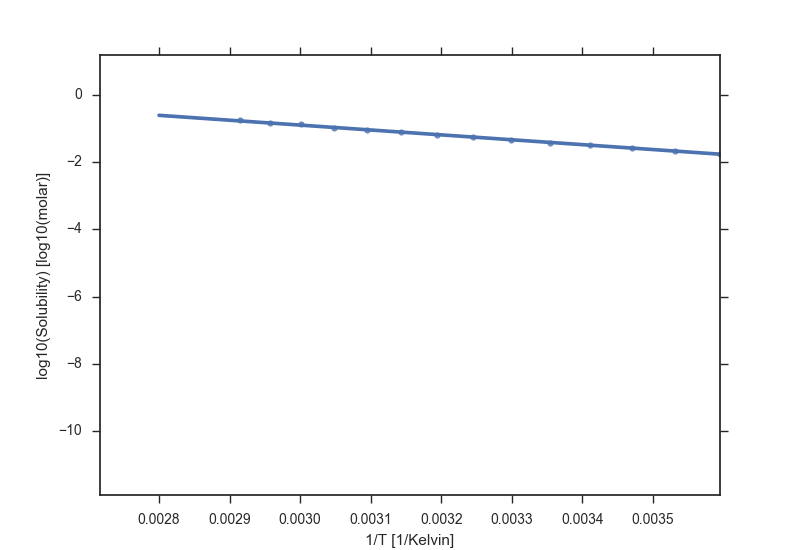

Supplement: Supplementary file 3 — Additional file 3. Additional results files, in electronic format. These additional results are (a) SUB-48 calculated lattice energies for the complete set and filtered set of 27 crystal structures, (b) results from analysis of the correspondence between our temperature dependent solubility data and (1/T), (c) Excel workbooks documenting all R2 and RMSE values obtained from cross-validation, their mean values and the p values (both raw and adjusted) obtained from pairwise comparisons of corresponding models, (d) comparison of melting point data used for the melting point descriptor and retrieved from the CSD for linked refcodes. [file 13321_2018_298_MOESM3_ESM.zip › additional_results/vantHoff_assessment/vh_plots/Klimenko_QSPRin_CD_False_MD_IntegSub.SiRMSSub.Absolv.Ind.Rdk_readyForR_vh.csv_308.tiff]

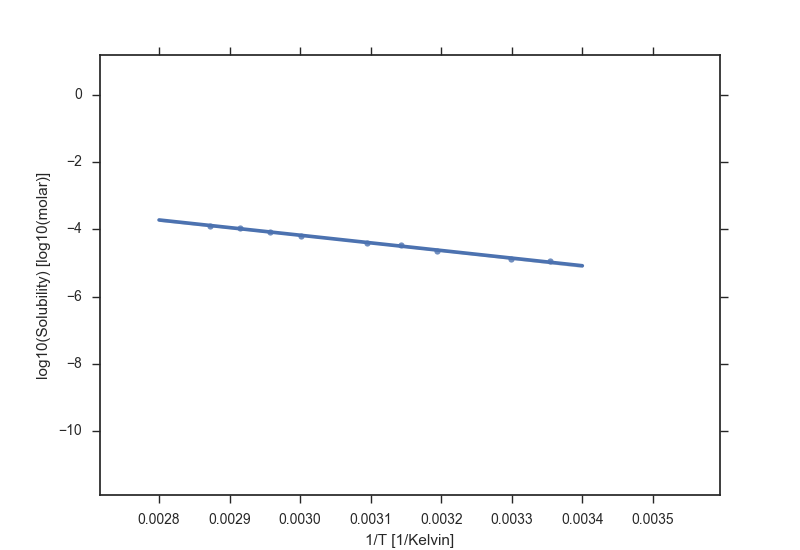

Supplement: Supplementary file 3 — Additional file 3. Additional results files, in electronic format. These additional results are (a) SUB-48 calculated lattice energies for the complete set and filtered set of 27 crystal structures, (b) results from analysis of the correspondence between our temperature dependent solubility data and (1/T), (c) Excel workbooks documenting all R2 and RMSE values obtained from cross-validation, their mean values and the p values (both raw and adjusted) obtained from pairwise comparisons of corresponding models, (d) comparison of melting point data used for the melting point descriptor and retrieved from the CSD for linked refcodes. [file 13321_2018_298_MOESM3_ESM.zip › additional_results/vantHoff_assessment/vh_plots/Klimenko_QSPRin_CD_False_MD_IntegSub.SiRMSSub.Absolv.Ind.Rdk_readyForR_vh.csv_309.tiff]

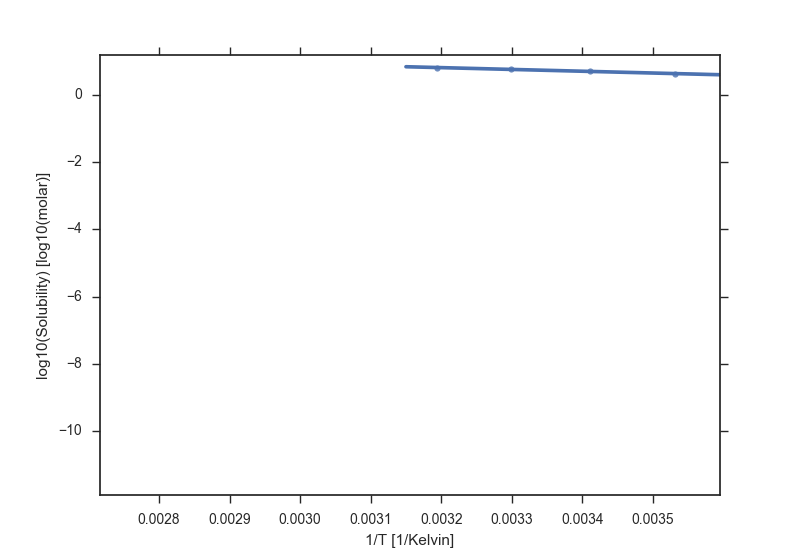

Supplement: Supplementary file 3 — Additional file 3. Additional results files, in electronic format. These additional results are (a) SUB-48 calculated lattice energies for the complete set and filtered set of 27 crystal structures, (b) results from analysis of the correspondence between our temperature dependent solubility data and (1/T), (c) Excel workbooks documenting all R2 and RMSE values obtained from cross-validation, their mean values and the p values (both raw and adjusted) obtained from pairwise comparisons of corresponding models, (d) comparison of melting point data used for the melting point descriptor and retrieved from the CSD for linked refcodes. [file 13321_2018_298_MOESM3_ESM.zip › additional_results/vantHoff_assessment/vh_plots/Klimenko_QSPRin_CD_False_MD_IntegSub.SiRMSSub.Absolv.Ind.Rdk_readyForR_vh.csv_33.tiff]

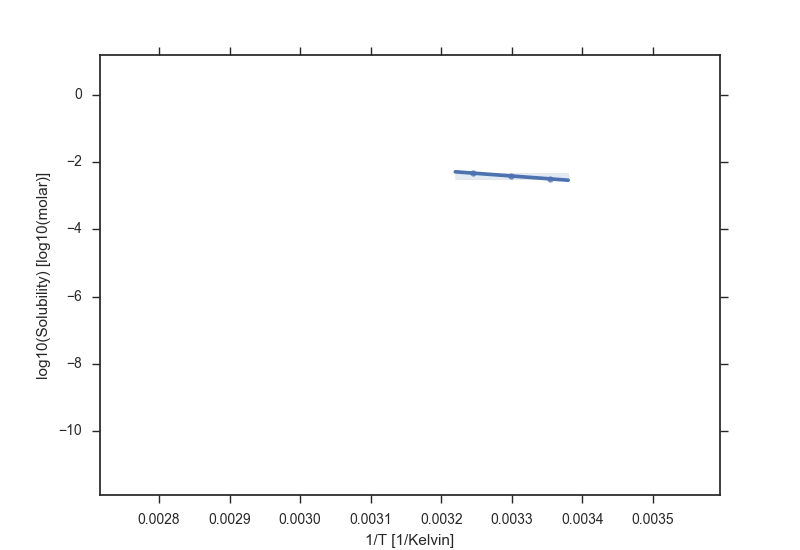

Supplement: Supplementary file 3 — Additional file 3. Additional results files, in electronic format. These additional results are (a) SUB-48 calculated lattice energies for the complete set and filtered set of 27 crystal structures, (b) results from analysis of the correspondence between our temperature dependent solubility data and (1/T), (c) Excel workbooks documenting all R2 and RMSE values obtained from cross-validation, their mean values and the p values (both raw and adjusted) obtained from pairwise comparisons of corresponding models, (d) comparison of melting point data used for the melting point descriptor and retrieved from the CSD for linked refcodes. [file 13321_2018_298_MOESM3_ESM.zip › additional_results/vantHoff_assessment/vh_plots/Klimenko_QSPRin_CD_False_MD_IntegSub.SiRMSSub.Absolv.Ind.Rdk_readyForR_vh.csv_36.tiff]

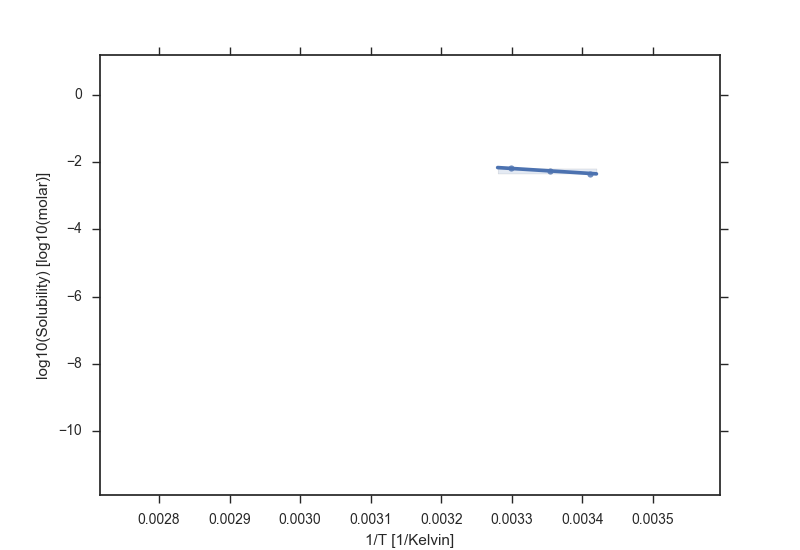

Supplement: Supplementary file 3 — Additional file 3. Additional results files, in electronic format. These additional results are (a) SUB-48 calculated lattice energies for the complete set and filtered set of 27 crystal structures, (b) results from analysis of the correspondence between our temperature dependent solubility data and (1/T), (c) Excel workbooks documenting all R2 and RMSE values obtained from cross-validation, their mean values and the p values (both raw and adjusted) obtained from pairwise comparisons of corresponding models, (d) comparison of melting point data used for the melting point descriptor and retrieved from the CSD for linked refcodes. [file 13321_2018_298_MOESM3_ESM.zip › additional_results/vantHoff_assessment/vh_plots/Klimenko_QSPRin_CD_False_MD_IntegSub.SiRMSSub.Absolv.Ind.Rdk_readyForR_vh.csv_38.tiff]

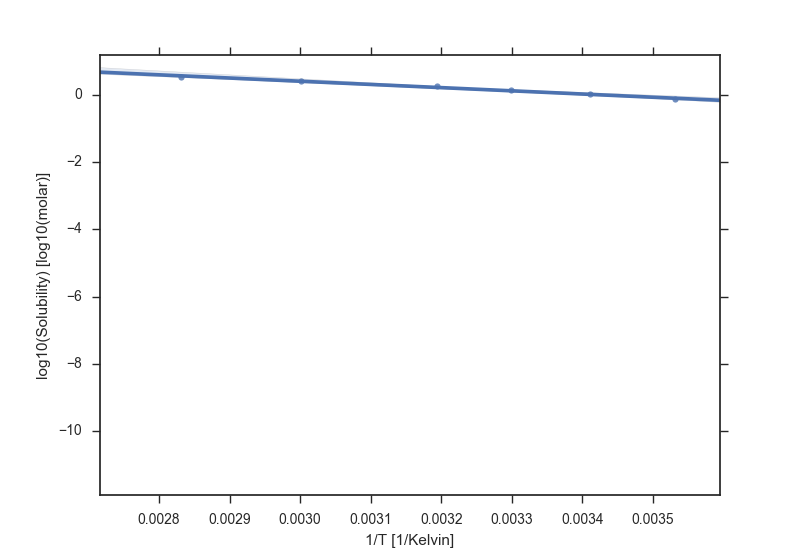

Supplement: Supplementary file 3 — Additional file 3. Additional results files, in electronic format. These additional results are (a) SUB-48 calculated lattice energies for the complete set and filtered set of 27 crystal structures, (b) results from analysis of the correspondence between our temperature dependent solubility data and (1/T), (c) Excel workbooks documenting all R2 and RMSE values obtained from cross-validation, their mean values and the p values (both raw and adjusted) obtained from pairwise comparisons of corresponding models, (d) comparison of melting point data used for the melting point descriptor and retrieved from the CSD for linked refcodes. [file 13321_2018_298_MOESM3_ESM.zip › additional_results/vantHoff_assessment/vh_plots/Klimenko_QSPRin_CD_False_MD_IntegSub.SiRMSSub.Absolv.Ind.Rdk_readyForR_vh.csv_4.tiff]

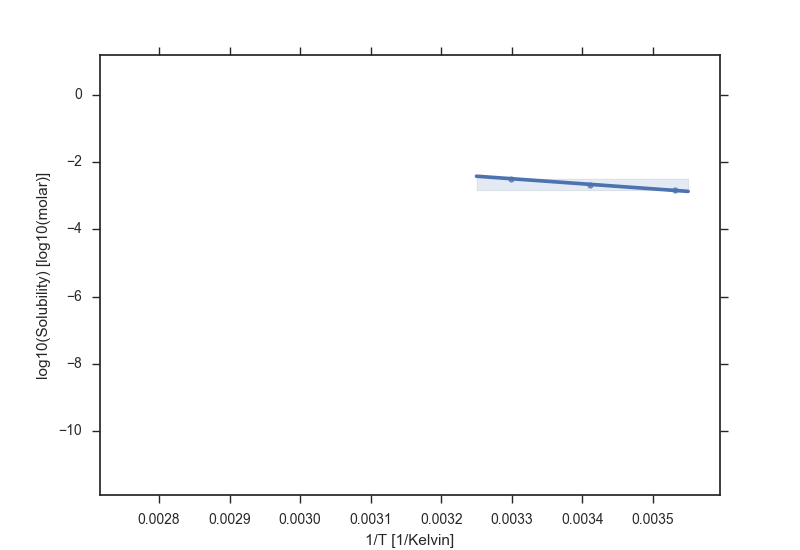

Supplement: Supplementary file 3 — Additional file 3. Additional results files, in electronic format. These additional results are (a) SUB-48 calculated lattice energies for the complete set and filtered set of 27 crystal structures, (b) results from analysis of the correspondence between our temperature dependent solubility data and (1/T), (c) Excel workbooks documenting all R2 and RMSE values obtained from cross-validation, their mean values and the p values (both raw and adjusted) obtained from pairwise comparisons of corresponding models, (d) comparison of melting point data used for the melting point descriptor and retrieved from the CSD for linked refcodes. [file 13321_2018_298_MOESM3_ESM.zip › additional_results/vantHoff_assessment/vh_plots/Klimenko_QSPRin_CD_False_MD_IntegSub.SiRMSSub.Absolv.Ind.Rdk_readyForR_vh.csv_46.tiff]

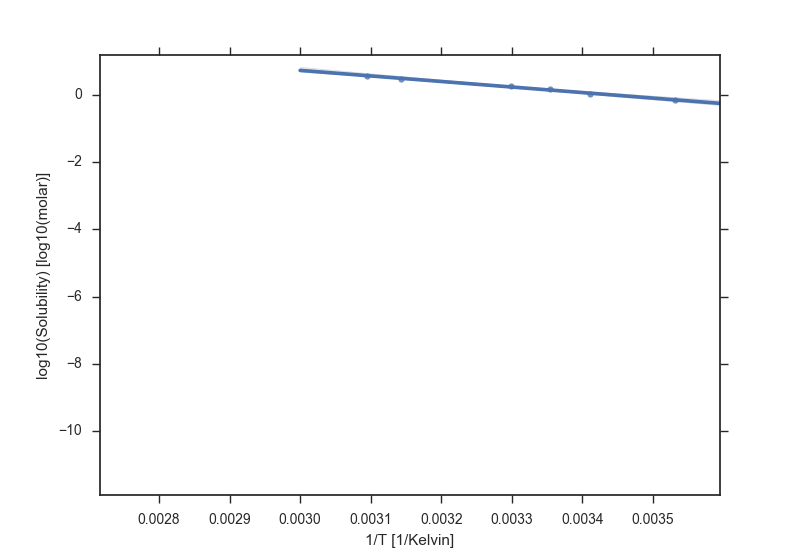

Supplement: Supplementary file 3 — Additional file 3. Additional results files, in electronic format. These additional results are (a) SUB-48 calculated lattice energies for the complete set and filtered set of 27 crystal structures, (b) results from analysis of the correspondence between our temperature dependent solubility data and (1/T), (c) Excel workbooks documenting all R2 and RMSE values obtained from cross-validation, their mean values and the p values (both raw and adjusted) obtained from pairwise comparisons of corresponding models, (d) comparison of melting point data used for the melting point descriptor and retrieved from the CSD for linked refcodes. [file 13321_2018_298_MOESM3_ESM.zip › additional_results/vantHoff_assessment/vh_plots/Klimenko_QSPRin_CD_False_MD_IntegSub.SiRMSSub.Absolv.Ind.Rdk_readyForR_vh.csv_48.tiff]
